# Iridium‐Catalyzed Reductive Deoxygenation of Esters for the Synthesis of Sterically Hindered Ethers

**DOI:** 10.1002/anie.202508301

**Published:** 2025-08-26

**Authors:** Yaseen A. Almehmadi, Anna J. Passmore, Pablo Gabriel, Darren J. Dixon

**Affiliations:** ^1^ Department of Chemistry Chemistry Research Laboratory University of Oxford 12 Mansfield Road Oxford OX1 3TA UK; ^2^ Department of Chemistry Rabigh College of Science and Arts King Abdulaziz University Jeddah PO Box 344 Saudi Arabia

**Keywords:** Ester reduction, Ethers, Hindered ether synthesis, Iridium catalysis, Silanes

## Abstract

The synthesis of sterically hindered α‐tertiary and β‐quaternary (neopentylic) ethers has long been constrained by the limitations of traditional S_N_2 and related S_N_1 approaches, namely low or inexistent reactivity arising from severe steric hindrance or competitive elimination/rearrangement pathways diverting the reaction outcome. Herein, we describe a general solution to the synthesis of sterically hindered ethers via an iridium‐catalyzed reductive deoxygenation reaction of readily available ester and lactone starting materials. Employing commercially available, bench‐stable IrCl(CO)(P[OCH(CF_3_)_2_]_3_)_2_ as a precatalyst at 1 mol% loading with 4 equivalents of tetramethyldisiloxane (TMDS) as the terminal reductant at room temperature, this practical synthetic approach to hindered ethers features a simple, mix‐and‐stir, single‐vessel protocol under ambient conditions and produces a diverse range of both acyclic and cyclic ether products in good to excellent yields. Control experiments demonstrated that the IrCl(CO)(P[OCH(CF_3_)_2_]_3_)_2_/TMDS catalytic system could not only rapidly hydrosilylate esters to mixed silyl/alkyl hemiacetal intermediates but also catalyze the reduction of acetals directly to ethers, revealing the Lewis acidic and hydridic properties required for this deoxygenative transformation.

## Graphical abstract

A general method for accessing sterically hindered ethers via reductive deoxygenation of readily prepared ester starting materials is reported. Employing commercially available and bench‐stable IrCl(CO)(P[OCH(CF_3_)_2_]_3_)_2_ (1–2 mol%) and tetramethyldisiloxane (TMDS) as reductant, this single‐vessel approach operates under mild conditions and provides a range of alkyl, aryl, and benzyl ethers—both acyclic and cyclic—in good to excellent yields.

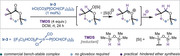

## Introduction

**Scheme 1:**
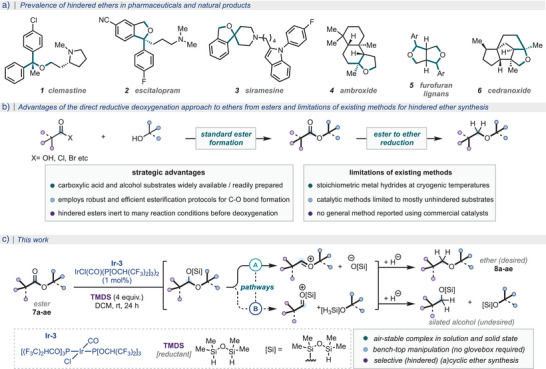
a) Prevalence of hindered ethers in pharmaceuticals and natural products; b) Direct reduction of ester to ether approach and limitations of existing methods; c) This work.

Sterically hindered ethers, such as those possessing *α*‐tertiary or *β*‐quaternary (neopentylic) carbon atoms, are commonplace structural motifs found across biologically relevant molecules, such as natural products and pharmaceutical compounds (Scheme 1a).^[^ ^1^, ^2^, ^3^ ^]^ Despite their abundance and importance, there are numerous challenges associated with the synthesis of these motifs. Textbook synthetic approaches, such as the venerable Williamson reaction or the Mitsunobu reaction, can often deliver unhindered ethers with relative ease but are rarely applicable to the synthesis of more sterically encumbered variants.^[^ ^4^, ^5^, ^6^ ^]^ For example, attempted bimolecular union of sterically demanding alkoxide nucleophiles with secondary, tertiary, and neopentylic electrophilic coupling partners typically fails due to severe steric repulsions hindering the desired S_N_2 pathway. This results in low or inexistent reactivity, or competitive elimination/rearrangement pathways dominating the reaction outcome.^[^ ^7^, ^8^, ^9^, ^10^ ^]^ An alternative approach to the synthesis of *α*‐tertiary ethers relies on weak alcohol nucleophiles intercepting tertiary carbocationic intermediates, formed via acid‐mediated reactions (solvolysis) or by protonation of a suitable alkene substrate. However, acid sensitivity of some substrates, poor reactivity, and competitive rearrangement pathways limit the applicability of this approach.^[^ ^11^ ^]^ To address these challenges, in recent years, significant efforts have been made to improve the synthetic accessibility of such hindered ethers.^[^ ^3^ ^]^ Productive lines of enquiry have focused on single‐electron reaction manifolds using both electrochemical and photochemical activation approaches, with notable contributions from the group of Baran,^[^ ^12^, ^13^ ^]^ Ohmiya,^[^ ^14^, ^15^, ^16^ ^]^ Knowles,^[^ ^17^ ^]^ Doyle,^[^ ^18^, ^19^ ^]^ Glorius,^[^ ^20^, ^21^ ^]^ as well as our own.^[^ ^22^, ^23^ ^]^ Despite these advances, major synthetic hurdles still remain in achieving their synthesis, especially for *α*‐tertiary or *β*‐quaternary (neopentylic) ethers, underscoring the need for novel chemical methodologies to address this ongoing challenge.^[^ ^3^ ^]^

As a general and practical solution to hindered ether synthesis, we were drawn to the conceptually simple yet strategically powerful ester reductive deoxygenation approach. This would enable the direct synthesis of hindered ethers from naturally occurring or commercially available esters or lactone starting materials or, in two steps, from abundant feedstock carboxylic acids (or derivatives) and alcohols using well‐established and robust esterification methods, which tolerate steric hindrance on both acyl and alkoxy sides of the ester moiety.^[^ ^24^ ^]^ Not unsurprisingly, this reductive deoxygenative approach toward the synthesis of sterically hindered (and unhindered) ethers has been investigated previously using stoichiometric amounts of strong metal hydride reagents, such as lithium aluminum hydride (LiAlH_4_) or diisobutylaluminum hydride (DIBAL), followed by an in situ acylation or silylation of the metalated hemiacetal intermediate. In a subsequent step, a Lewis acidic activator, such as boron trifluoride etherate (BF_3_·Et_2_O) in the presence of a silane terminal reductant, provides access to the ether product from the hemiacetal intermediate.^[^ ^25^, ^26^, ^27^, ^28^, ^29^ ^]^ However, the use of stoichiometric metal hydride reagents at carefully controlled cryogenic temperatures, the two‐step nature of the protocols, and the relatively harsh reaction conditions somewhat limit this approach in both academic and industrial settings.

More recently, catalytic variants of the reductive deoxygenation process for accessing ether functionality directly from relatively simple and mostly unhindered ester substrates have been reported using various catalytic systems based on organometallic complexes of Ru,^[^ ^30^, ^31^ ^]^ Fe,^[^ ^32^ ^]^ Mn,^[^ ^33^, ^34^ ^]^ Ti,^[^ ^35^, ^36^, ^37^ ^]^ Rh,^[^ ^38^ ^]^ Pd,^[^ ^39^ ^]^ Ir,^[^ ^40^, ^41^, ^42^, ^43^, ^44^, ^45^, ^46^ ^]^ and main group metal salts such as GaBr_3_,^[^ ^47^ ^]^ InBr_3_,^[^ ^48^, ^49^, ^50^ ^]^ and fluorinated borate salts or boranes^[^ ^51^, ^52^, ^53^ ^]^ in conjunction with excess silanes as the terminal reductant. However, for sterically demanding ester substrates, i.e., those possessing fully substituted carbon atoms on both sides of the carboxyl group, a general and practical catalytic reductive deoxygenation methodology to access the sterically hindered dialkyl ether products remains undeveloped.

Inspired by these pioneering studies and building on our own^[^ ^54^, ^55^, ^56^, ^57^, ^58^, ^59^, ^60^, ^61^, ^62^, ^63^, ^64^, ^65^, ^66^, ^67^, ^68^, ^69^ ^]^ and others’^[^ ^70^, ^71^, ^72^, ^73^, ^74^, ^75^, ^76^, ^77^, ^78^, ^79^, ^80^, ^81^, ^82^, ^83^, ^84^, ^85^, ^86^, ^87^ ^]^ findings in the field of amide reductive functionalization using Vaska's complex IrCl(CO)(PPh_3_)_2_ **Ir‐1** with TMDS and the work of Brookhart on the hydrosilylation of esters to access aldehydes,^[^ ^43^ ^]^ we reasoned that a selective catalytic reductive deoxygenation of hindered esters to hindered ethers could indeed be developed if a more powerful derivative of Vaska's complex in conjunction with a suitable silane terminal reductant could be identified. Previous work by our group had revealed that with less electron‐rich anilide or hydrazide substrates, significantly enhanced carbonyl hydrosilylation reactivity could be achieved using electron‐deficient triphenyl phosphite ligands in lieu of triphenyl phosphine on Vaska's complex.^[^ ^59^, ^88^ ^]^ Extending these observations to ester substrates, we envisioned that the necessary reactivity that we sought could indeed come from such phosphite analogues of Vaska's complex (Scheme 1c), and herein we present our findings.

## Results and Discussion

Isopropyl 4‐fluorobenzoate **7a** was selected as a model ester substrate to explore the catalytic reductive deoxygenation reaction. Initially, we reconfirmed many previous observations that subjecting ester **7a** to standard hydrosilylation reaction conditions using Vaska's complex **Ir‐1** (1.0 mol%) and 2.0 equivalents of 1,1,3,3‐tetramethyldisiloxane (TMDS)^[^ ^89^ ^]^ as the hydride source in toluene (0.1 M) at room temperature resulted in zero reactivity. Increasing the amount of TMDS to 5 equivalents, the concentration to 0.5 M in toluene, and the reaction time to 24 h also resulted in no conversion and full recovery of the starting material (Table 1, entry 1). We then turned our attention to investigating the performance of derivatives of Vaska's complex **Ir‐2** and **Ir‐3**, possessing triphenyl phosphite and tris(1,1,1,3,3,3‐hexafluoro‐2‐propyl) phosphite P[OCH(CF_3_)_2_]_3_ ligands, respectively.^[^ ^59^, ^77^ ^]^

**Table 1:** Precatalyst screen and reaction optimization.

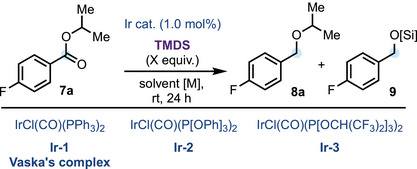							
						Ratio (%)	
Entry	Solvent	TMDS (X equiv.)	Conc. [M]	Ir cat.	Conv. %	**8a**	**9**
1	toluene	5.0	0.5	Ir‐1	0	0	0
2	toluene	5.0	0.5	Ir‐2	8	60	40
3	toluene	5.0	0.5	Ir‐3	100	90	10
4	DCM	5.0	0.5	Ir‐3	100	94	6
5	Et_2_O	5.0	0.5	Ir‐3	86	90	10
6	THF	5.0	0.5	Ir‐3	85	89	11
7	(TMDS)	5.0	neat	Ir‐3	100	>95	5
8	DCM	5.0	1.0	Ir‐3	100	>95	5
**9**	**DCM**	**4.0**	**1.0**	**Ir‐3**	**100**	**>95**	**5**
10	DCM	3.0	1.0	Ir‐3	88	>95	5

Encouragingly, use of IrCl(CO)(P[OPh]_3_)_2_ **Ir‐2** resulted in an 8% conversion of ester **7a** to the desired isopropyl ether **8a** and the undesired silylated alcohol **9a** in a 60:40 ratio, respectively (Table 1, entry 2). However, IrCl(CO)(P[OCH(CF_3_)_2_]_3_)_2_ **Ir‐3**, an air‐stable, bright yellow crystalline solid,^[^ ^77^, ^90^ ^]^ under the same reaction conditions (Table 1, entry 3), resulted impressively in complete conversion of ester (**7a**) to the corresponding ether **8a** and silylated alcohol **9** in a 90:10 ratio, respectively. This brief precatalyst investigation identified that **Ir‐3** complex was ideally suited for this reductive deoxygenative transformation, providing the necessary hydrosilylation reactivity and the Lewis acidity to form the putative oxocarbenium ion from the mixed silyl acetal intermediate prior to terminal hydride addition. Upon examining different reaction solvents such as dichloromethane (DCM), diethyl ether (Et_2_O), and tetrahydrofuran (THF), DCM was identified as the most suitable solvent for this reaction, increasing the ratio of **8a**:**9** to 94:6, respectively (Table 1, entries 4–6). Further optimization of the concentration of the reaction and the equivalents of TMDS (Table 1, entries 7–10) led to the optimized reaction conditions (1.0 M in DCM, 4 equivalents of TMDS), which provided the desired isopropyl ether **8a** in 95% yield and in excellent (>95:5) product selectivity (Table 1, entry 9).

**Scheme 2:**
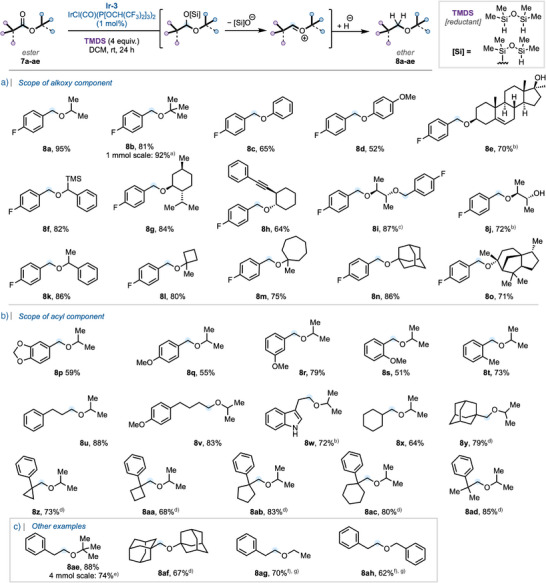
Substrate scope of the reduction of (hindered) acyclic esters to ethers; all yields are isolated yields. ^a)^0.5 mol% of **Ir‐3** was used in neat TMDS. ^b)^5.0 equivalents of TMDS were used. ^c)^2 mol% of **Ir‐3** and 8.0 equivalents of TMDS were used. ^d)^2 mol% of **Ir‐3** was used in neat TMDS (4.0 equiv.), and TMSOTf (1.0 equiv.) was added at rt after 24 h. ^e)^0.25 mol% of **Ir‐3** was used in neat TMDS. ^f)^The reaction was carried out in 0.1 M in toluene, and the reduction was complete within 1 h at rt instead of 24 h. ^g^TMSOTf (1.0 equiv.) was added at rt after 1 h.

Having established a practically simple and highly efficient protocol for the reductive deoxygenation of model substrate **7a**, we then turned our attention to the scope of the reaction. Initially we explored its generality with respect to the alkoxy group of 4‐fluorobenzoyl esters (Scheme 2a). Under the optimized conditions, increasing the bulk of the alkoxy component from isopropyl (**7a**) to *tert*‐butyl (**7b**) gratifyingly resulted in an excellent yield of alpha *tert*‐butyl ether **8b**. The 4‐fluorobenzoic ester of phenol (**7c**) and *para*‐methoxy phenol (**7d**) were also tolerated under our reductive protocol, providing a good yield of their respective ethers **8c**–**d**. Furthermore, esters derived from methandriol (**7e**), trimethylsilyl‐containing alcohol (**7f**), menthol (**7g**), and alkyne‐containing alcohol (**7h**) were also amenable to this methodology. Pleasingly, double reductive deoxygenation of diester **7i** proceeded in high yield (87%), as did the reductive deoxygenation of an ester carbonyl in the presence of an unprotected secondary alcohol (**7j**), although an additional equivalent of TMDS was required for transient in situ alcohol silylation. 4‐Fluorobenzoyl esters of other more sterically hindered alcohols (**7k–o**) were smoothly reduced to their corresponding hindered ethers **8k–o** in good yields (71%–86%). Remarkably, the reduction of chiral esters **7e**, **7g–j**, and **7o** proceeded without observable racemization, delivering the corresponding hindered ethers **8e**, **8g–j**, and **8o** in good to excellent yields.

Next, keeping the isopropoxy group as the model alcohol component, the scope with respect to the acyl group was then investigated (Scheme 2b). Pleasingly, switching the 4‐fluorobenzoyl group to 3,4‐methylenedioxybenzoyl, 4‐methoxybenzoyl, 3‐methoxybenzoyl, 2‐methoxybenzoyl, and 2‐methylbenzoyl gave acceptable yields of the desired ether products, **8p–t**, respectively. Importantly, isopropyl esters derived from linear aliphatic carboxylic acids such as **7u**, **7v**, and **7w**, featuring an unprotected indole, were excellent substrates and resulted in the formation of their respective ethers **8u**–**w** in good to excellent yields. Similarly, isopropyl cyclohexyl carboxylate **7x**, when also subjected to the optimal reaction conditions, resulted in the formation of ether **8x** in good yield. Furthermore, isopropyl *α*‐tertiary carboxylates **7y**–**ad** were successfully reduced to the corresponding ethers **8y–ad**; however, in the cases of such sterically hindered *α*‐tertiary alkyl esters, solvent‐free conditions and 2 mol % of **Ir‐3** for the initial hydrosilyation step, followed by direct addition of TMSOTf (1 equiv.) as a Lewis acid to promote ionization of the mixed silyl hemiacetals prior to hydride addition, were required.

Other synthetically relevant examples are shown in Scheme 2c. *tert*‐Butyl 2‐phenylacetate **7ae** was reduced in excellent yield to the corresponding ether **8ae** under the standard conditions. This example is particularly noteworthy owing to the lack of feasibility of accessing such a compound using an S_N_2 approach with *tert*‐butoxide as the nucleophile. 1‐Adamantyl adamantane‐1‐carboxylate **7af** was successfully reduced to the corresponding ether **8af** in acceptable 67% yield using the protocol described above for other α‐tertiary alkyl esters. Gaining access to such hindered neopentylic ether products using alternative synthetic approaches would be otherwise difficult. Pleasingly, a slight adjustment of the reaction conditions also enabled the reduction of less hindered esters **7ag** and **7ah** to the corresponding ethers **8ag** and **8ah** in 70% and 62% yield, respectively. Although the main scope was routinely carried out on 0.2 mmol scale, we successfully demonstrated that it could be carried out on a 1 mmol (**7b**) and 4 mmol scale (**7ae**) using 0.5 mol% and 0.25 mol% loading of **Ir‐3**, respectively, while maintaining reaction efficiency (see Supporting Information). Using **7ae** as a model substrate, a Glorius robustness screen^[^ ^91^ ^]^ was carried out to test the procedure's compatibility with different additives (see Supporting Information). The reaction tolerated aryl bromides, chlorides, and iodides, and terminal alkenes with no reduction in yield or conversion of the additive. Conversely, nitrile and nitro groups were found to be incompatible, resulting in a decreased yield and consumption of the additive. Interestingly, with pyridine and secondary carbamate additives, the reactivity was strongly diminished, albeit with no loss of the additive, suggesting these functionalities were poisoning the active catalyst.

**Scheme 3:**
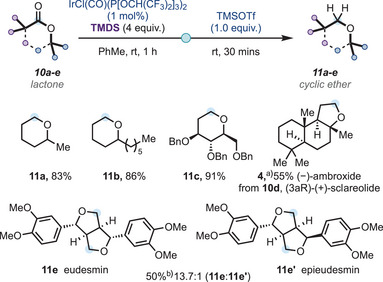
Reductive deoxygenation of lactones to cyclic ethers: scope and application in natural product synthesis; all yields are isolated yields. ^a)^20 mol% of TMSOTf was used. ^b)^TMSOTf was added at −20 °C.

Having successfully explored the scope of the reductive deoxygenation reaction for acyclic (hindered) esters to (hindered) ethers, we then turned our attention to cyclic systems (Scheme 3). Six‐membered lactones **10a**–**c** were accommodated in good‐to‐excellent yields to access cyclic ethers **11a**–**c**, although TMSOTf (1.0 equiv.) was necessary to trigger the second stage of the reductive deoxygenation reaction. Lowering the amount of TMSOTf to 20 mol% was also successfully demonstrated in the case of natural product (3aR)‐(+)‐sclareolide **10d**, which was successfully reduced to (−)‐ambroxide **4** in a good yield in a single vessel. Furthermore, natural product eudesmin **11e** was synthesized from the corresponding bicyclic bislactone **10e** in good yield and excellent diastereoselectivity; in this particular example, TMSOTf was added at −20 °C to avoid possible epimerization.^[^ ^92^ ^]^

**Scheme 4:**
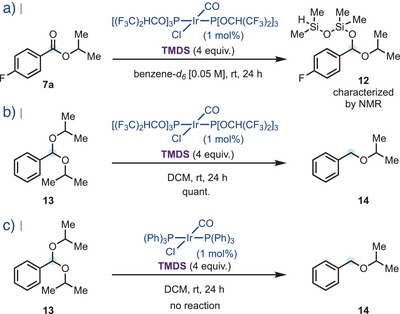
Mechanistic studies into carbonyl hydrosilylation and for acetal activation by the **Ir‐3**/TMDS precatalyst system; all yields are isolated yields.

To probe the proposed two‐stage mechanistic course of the reaction, the standard reduction conditions were applied to model substrate **7a**; however, with higher dilution in deuterated benzene for NMR monitoring purposes (Scheme 4a). Pleasingly, after 24 h at room temperature, ^1^H NMR analysis revealed the presence of silylated mixed acetal **12**, the direct product of hydrosilylation of **7a**. Furthermore, as full deoxygenation was occurring without added TMSOTf in the cases of benzoate ester substrates, **7a**–**7o**, as well as aroyl esters **8p–t** and linear aliphatic carboxylates **7u–w**, **7x**, and **7ae**, this indicated that in the presence of TMDS, **Ir‐3** acts not only as a strong hydrosilylating agent but also as a competent Lewis acid. To probe this hypothesis experimentally, hindered diisopropyl benzyl acetal **13** was synthesized and subjected to the optimized reaction conditions using **Ir‐3** (1 mol%) and TMDS (4 eq). As shown in Scheme 4b, after 24 h acetal **13** was indeed smoothly converted to the corresponding ether **14**, which was subsequently isolated in excellent yield, whereas the analogous reaction using Vaska's complex **Ir‐1** resulted in no reaction (Scheme 4c). These data are consistent with the hypothesis that in conjunction with TMDS, **Ir‐3** forms catalytic intermediates sufficiently Lewis acidic to generate putative oxo‐carbenium ions that are subsequently reduced by hydridic species present in the medium.

## Conclusion

In conclusion, an efficient and broad‐in‐scope iridium(I)‐catalyzed reductive deoxygenation of hindered esters to ethers has been successfully developed. The practically simple, mix‐and‐stir, single‐vessel, ambient‐temperature reaction protocol relies on the enhanced carbonyl reducing power of bench‐stable and commercially available^[^ ^90^ ^]^ iridium complex IrCl(CO)(P[OCH(CF_3_)_2_]_3_)_2_ **Ir‐3**, relative to that of Vaska's complex. Control experiments revealed that complex **Ir‐3** in the presence of TMDS engenders Lewis acidity, which, with the more activated substrates or bulky alkoxy esters derived from linear aliphatic carboxylic acids, enables overall deoxygenation without assistance from added Lewis acids. Experiments to further explore and apply the reactivity of **Ir‐3** and related complexes are actively under investigation in our group, and the results will be reported in due course.

## Conflict of Interests

D.J.D. is a co‐founder, director, and major shareholder of Cortex Organics Ltd.

## Supporting information



Supporting Information

## Data Availability

The data that support the findings of this study are available in the Supporting Information of this article.

## References

[1] S. D. Roughley , A. M. Jordan , J. Med. Chem. 2011, 54, 3451–3479. 21504168 10.1021/jm200187y

[2] P. D. de María , R. W. van Gemert , A. J. Straathof , U. Hanefeld , Nat. Prod. Rep. 2010, 27, 370–392. 20179877 10.1039/b809416k

[3] B. D. Shennan , D. Berheci , J. L. Crompton , T. A. Davidson , J. L. Field , B. A. Williams , D. J. Dixon , Chem. Soc. Rev. 2022, 51, 5878–5929. 35770619 10.1039/d1cs00669j

[4] A. Williamson , Justus Liebigs Annalen der Chemie. 1851, 77, 37–49.

[5] L. Kurti , B. Czakó , Strategic Applications of Named Reactions in Organic Synthesis, Elsevier, Academic Press, Amsterdam 2005.

[6] K. K. Swamy , N. B. Kumar , E. Balaraman , K. P. Kumar , Chem. Rev. 2009, 109, 2551–2651. 19382806 10.1021/cr800278z

[7] F. C. Whitmore , H. S. Rothrock , J. Am. Chem. Soc. 1932, 54, 3431–3435.

[8] I. Dostrovsky , E. Hughes , J. Chem. Soc. (Resumed) 1946, 171–173.

[9] P. De la Mare , L. Fowden , E. Hughes , C. Ingold , J. Mackie , Chem. Soc. 1955, 3200.

[10] P. R. Rablen , B. D. McLarney , B. J. Karlow , J. E. Schneider , J. Org. Chem. 2014, 79, 867–879. 24437451 10.1021/jo4026644

[11] H. Beyerman , G. Heiszwolf , Recueil des Travaux Chimiques des Pays‐Bas. 1965, 84, 203–212.

[12] J. Xiang , M. Shang , Y. Kawamata , H. Lundberg , S. H. Reisberg , M. Chen , P. Mykhailiuk , G. Beutner , M. R. Collins , A. Davies , M. Del Bel , G. M. Gallego , J. E. Spangler , J. Starr , S. Yang , D. G. Blackmond , P. S. Baran , Nature 2019, 573, 398–402. 31501569 10.1038/s41586-019-1539-y PMC6996793

[13] H.‐J. Zhang , L. Chen , M. S. Oderinde , J. T. Edwards , Y. Kawamata , P. S. Baran , Angew. Chem. Int. Ed. 2021, 60, 20700–20705. 10.1002/anie.202107820 PMC8429144 34288303

[14] M. Nakagawa , Y. Matsuki , K. Nagao , H. Ohmiya , J. Am. Chem. Soc. 2022, 144, 7953–7959. 35476545 10.1021/jacs.2c00527

[15] S. Shibutani , T. Kodo , M. Takeda , K. Nagao , N. Tokunaga , Y. Sasaki , H. Ohmiya , J. Am. Chem. Soc. 2020, 142, 1211–1216. 31898903 10.1021/jacs.9b12335

[16] S. Shibutani , K. Nagao , H. Ohmiya , Org. Lett. 2021, 23, 1798–1803. 33569947 10.1021/acs.orglett.1c00211

[17] Q. Zhu , E. C. Gentry , R. R. Knowles , Angew. Chem. Int. Ed. 2016, 55, 9969–9973. 10.1002/anie.201604619 PMC5102159 27403637

[18] E. W. Webb , J. B. Park , E. L. Cole , D. J. Donnelly , S. J. Bonacorsi , W. R. Ewing , A. G. Doyle , J. Am. Chem. Soc. 2020, 142, 9493–9500. 32378889 10.1021/jacs.0c03125

[19] K. M. Arendt , A. G. Doyle , Angew. Chem. Int. Ed. 2015, 54, 9876–9880. 10.1002/anie.201503936 PMC4629785 26219537

[20] L. Pitzer , F. Sandfort , F. Strieth‐Kalthoff , F. Glorius , J. Am. Chem. Soc. 2017, 139, 13652–13655. 28918623 10.1021/jacs.7b08086

[21] A. Tlahuext‐Aca , R. A. Garza‐Sanchez , F. Glorius , Angew. Chem. Int. Ed. 2017, 56, 3708–3711. 10.1002/anie.201700049 28221000

[22] J. A. Leitch , T. Rossolini , T. Rogova , D. J. Dixon , ACS Catal. 2020, 10, 11430–11437.

[23] T. Rossolini , B. Ferko , D. J. Dixon , Org. Lett. 2019, 21, 6668–6673. 31397159 10.1021/acs.orglett.9b02273

[24] J. Otera , J. Nishikido , Esterification: Methods, Reactions, and Applications, John Wiley & Sons, Hoboken 2009.

[25] G. Pettit , T. Kasturi , J. Org. Chem. 1960, 25, 875–876.

[26] G. R. Pettit , D. M. Piatak , J. Org. Chem. 1962, 27, 2127–2130.

[27] G. A. Kraus , K. A. Frazier , B. D. Roth , M. J. Taschner , K. Neuenschwander , J. Org. Chem. 1981, 46, 2417–2419.

[28] A. Hart , S. A. Kelley , T. Harless , J. A. Hood , M. Tagert , J. A. Pigza , Tetrahedron Lett. 2017, 58, 3024–3027.

[29] D. J. Kopecky , S. D. Rychnovsky , J. Org. Chem. 2000, 65, 191–198. 10813915 10.1021/jo9914521

[30] K. Matsubara , T. Iura , T. Maki , H. Nagashima , J. Org. Chem. 2002, 67, 4985–4988. 12098320 10.1021/jo025726n

[31] A. Tahara , Y. Sunada , T. Takeshita , R. Inoue , H. Nagashima , Chem. Commun. 2018, 54, 11192–11195. 10.1039/c8cc04780d 30229241

[32] S. Das , Y. Li , K. Junge , M. Beller , Chem. Commun. 2012, 48, 10742–10744. 10.1039/c2cc32142d 23024977

[33] Z. Mao , B. T. Gregg , A. R. Cutler , J. Am. Chem. Soc. 1995, 117, 10139–10140.

[34] O. Martínez‐Ferraté , B. Chatterjee , C. Werlé , W. Leitner , Catal. Sci. Technol. 2019, 9, 6370–6378.

[35] M. C. Hansen , X. Verdaguer , S. L. Buchwald , J. Org. Chem. 1998, 63, 2360–2361.

[36] M. Yato , K. Homma , A. Ishida , Tetrahedron 2001, 57, 5353–5359.

[37] P. V. Ramachandran , A. A. Alawaed , H. J. Hamann , Org. Lett. 2023, 25, 6902–6906. 37690034 10.1021/acs.orglett.3c02643

[38] S. Xu , J. S. Boschen , A. Biswas , T. Kobayashi , M. Pruski , T. L. Windus , A. D. Sadow , Dalton Trans. 2015, 44, 15897–15904. 26278517 10.1039/c5dt02844b

[39] S. Hosokawa , M. Toya , A. Noda , M. Morita , T. Ogawa , Y. Motoyama , ChemistrySelect 2018, 3, 2958–2961.

[40] G. S. Lu , Z. L. Ruan , Y. Wang , J. F. Lü , J. L. Ye , P. Q. Huang , Angew. Chem. Int. Ed. 2025, 64, e202422742. 10.1002/anie.202422742 39655429

[41] S. Park , M. Brookhart , Organometallics 2010, 29, 6057–6064. 21572562 10.1021/om100818y PMC3092162

[42] S. Park , D. Bézier , M. Brookhart , J. Am. Chem. Soc. 2012, 134, 11404–11407. 22765847 10.1021/ja305318c

[43] C. Cheng , M. Brookhart , Angew. Chem. Int. Ed. 2012, 51, 9422–9424. 10.1002/anie.201205154 22907673

[44] T. T. Metsänen , P. Hrobárik , H. F. Klare , M. Kaupp , M. Oestreich , J. Am. Chem. Soc. 2014, 136, 6912–6915. 24784900 10.1021/ja503254f

[45] K. Stęsik , A. Franczyk , A. Czapik , I. Kownacki , J. Walkowiak , ChemCatChem 2023, 15, e202201510.

[46] Y. Corre , V. Rysak , X. Trivelli , F. Agbossou‐Niedercorn , C. Michon , Eur. J. Org. Chem. 2017, 2017, 4820–4826.

[47] U. Biermann , J. O. Metzger , ChemSusChem 2014, 7, 644–649. 24488681 10.1002/cssc.201300627

[48] N. Sakai , T. Moriya , T. Konakahara , J. Org. Chem. 2007, 72, 5920–5922. 17602594 10.1021/jo070814z

[49] N. Sakai , T. Moriya , K. Fujii , T. Konakahara , Synthesis 2008, 2008, 3533–3536.

[50] N. Sakai , Y. Usui , R. Ikeda , T. Konakahara , Adv. Synth. Catal. 2011, 353, 3397–3401.

[51] V. Rysak , R. Dixit , X. Trivelli , N. Merle , F. Agbossou‐ Niedercorn , K. Vanka , C. Michon , Catal. Sci. Technol. 2020, 10, 4586–4592.

[52] Á. Dudás , Á. Gyömöre , B. B. Mészáros , S. Gondár , R. Adamik , D. Fegyverneki , D. Papp , K. B. Otte , S. Ayala, Jr. , J. Daru , J. Répási , T. Soós , J. Am. Chem. Soc. 2025, 147, 1112–1122. 39723648 10.1021/jacs.4c14596 PMC11726553

[53] B. B. Mészáros , Á. Dudás , A. Preszner , G. Sztanó , B. Csesztregi , C. Horváth , P. Szabó , J. Daru , T. Soós , ChemRxiv preprint 2025, 10.26434/chemrxiv-2025-lnvc3-v2. PMC13452557 42273760

[54] B. D. Shennan , S. Sánchez‐Alonso , G. Rossini , D. J. Dixon , J. Am. Chem. Soc. 2023, 145, 21745–21751. 37756523 10.1021/jacs.3c08466 PMC10571086

[55] P. Biallas , K. Yamazaki , D. J. Dixon , Org. Lett. 2022, 24, 2002–2007. 35258311 10.1021/acs.orglett.2c00438 PMC9082613

[56] K. Yamazaki , P. Gabriel , G. Di Carmine , J. Pedroni , M. Farizyan , T. A. Hamlin , D. J. Dixon , ACS Catal. 2021, 11, 7489–7497. 34306810 10.1021/acscatal.1c01589 PMC8291578

[57] D. Matheau‐Raven , D. J. Dixon , Angew. Chem. Int. Ed. 2021, 60, 19725–19729. 10.1002/anie.202107536 PMC8457168 34191400

[58] P. Gabriel , Y. A. Almehmadi , Z. R. Wong , D. J. Dixon , J. Am. Chem. Soc. 2021, 143, 10828–10835. 34254792 10.1021/jacs.1c04980 PMC8397322

[59] T. Rogova , P. Gabriel , S. Zavitsanou , J. A. Leitch , F. Duarte , D. J. Dixon , ACS Catal. 2020, 10, 11438–11447.

[60] D. Matheau‐Raven , P. Gabriel , J. A. Leitch , Y. A. Almehmadi , K. Yamazaki , D. J. Dixon , ACS Catal. 2020, 10, 8880–8897.

[61] L.‐G. Xie , J. Rogers , I. Anastasiou , J. A. Leitch , D. J. Dixon , Org. Lett. 2019, 21, 6663–6667. 31397155 10.1021/acs.orglett.9b02119

[62] L.‐G. Xie , D. J. Dixon , Nat. Commun. 2018, 9, 2841. 30026608 10.1038/s41467-018-05192-7 PMC6053461

[63] L.‐G. Xie , D. J. Dixon , Chem. Sci. 2017, 8, 7492–7497. 29163902 10.1039/c7sc03613b PMC5676097

[64] H. Shi , I. N. Michaelides , B. Darses , P. Jakubec , Q. N. N. Nguyen , R. S. Paton , D. J. Dixon , J. Am. Chem. Soc. 2017, 139, 17755–17758. 29120635 10.1021/jacs.7b10956

[65] Á. L. Fuentes de Arriba , E. Lenci , M. Sonawane , O. Formery , D. J. Dixon , Angew. Chem. Int. Ed. 2017, 56, 3655–3659. 10.1002/anie.201612367 28233919

[66] P. W. Tan , J. Seayad , D. J. Dixon , Angew. Chem. Int. Ed. 2016, 55, 13436–13440. 10.1002/anie.201605503 27659476

[67] A. W. Gregory , A. Chambers , A. Hawkins , P. Jakubec , D. J. Dixon , Chem. ‐ Eur. J. 2015, 21, 111–114. 25399919 10.1002/chem.201405256 PMC4730865

[68] B. Pijper , R. Martín , A. J. Huertas‐Alonso , M. L. Linares , E. López , J. Llaveria , Á. Díaz‐Ortiz , D. J. Dixon , A. de la Hoz , J. Alcázar , Org. Lett. 2024, 26, 2724–2728. 37219892 10.1021/acs.orglett.3c01390 PMC11020161

[69] P. Gabriel , A. W. Gregory , D. J. Dixon , Org. Lett. 2019, 21, 6658–6662. 31397160 10.1021/acs.orglett.9b02194

[70] S. Katahara , S. Kobayashi , K. Fujita , T. Matsumoto , T. Sato , N. Chida , J. Am. Chem. Soc. 2016, 138, 5246–5249. 27071479 10.1021/jacs.6b02324

[71] M. Yoritate , Y. Takahashi , H. Tajima , C. Ogihara , T. Yokoyama , Y. Soda , T. Oishi , T. Sato , N. Chida , J. Am. Chem. Soc. 2017, 139, 18386–18391. 29179540 10.1021/jacs.7b10944

[72] Z.‐P. Yang , Q. He , J.‐L. Ye , P.‐Q. Huang , Org. Lett. 2018, 20, 4200–4203. 29969900 10.1021/acs.orglett.8b01579

[73] W. Ou , G. S. Lu , D. An , F. Han , P. Q. Huang , Eur. J. Org. Chem. 2020, 2020, 52–56.

[74] S. Katahara , Y. Sugiyama , M. Yamane , Y. Komiya , T. Sato , N. Chida , Org. Lett. 2021, 23, 3058–3063. 33822636 10.1021/acs.orglett.1c00735

[75] Y. Motoyama , M. Aoki , N. Takaoka , R. Aoto , H. Nagashima , Chem. Commun. 2009, 1574–1576. 10.1039/b821317h 19277394

[76] M. Nakajima , T. Sato , N. Chida , Org. Lett. 2015, 17, 1696–1699. 25815605 10.1021/acs.orglett.5b00664

[77] A. Tahara , Y. Miyamoto , R. Aoto , K. Shigeta , Y. Une , Y. Sunada , Y. Motoyama , H. Nagashima , Organometallics 2015, 34, 4895–4907.

[78] Y. Une , A. Tahara , Y. Miyamoto , Y. Sunada , H. Nagashima , Organometallics 2019, 38, 852–862.

[79] Y. Nakayama , Y. Maeda , M. Kotatsu , R. Sekiya , M. Ichiki , T. Sato , N. Chida , Chem. ‐ Eur. J. 2016, 22, 3300–3303. 26756545 10.1002/chem.201600058

[80] S. Yamamoto , Y. Komiya , A. Kobayashi , R. Minamikawa , T. Oishi , T. Sato , N. Chida , Org. Lett. 2019, 21, 1868–1871. 30817163 10.1021/acs.orglett.9b00478

[81] Y. Takahashi , T. Sato , N. Chida , Chem. Lett. 2019, 48, 1138–1141.

[82] Y. Soda , Y. Sugiyama , M. Yoritate , H. Tajima , K. Shibuya , C. Ogihara , T. Oishi , T. Sato , N. Chida , Org. Lett. 2020, 22, 7502–7507. 32960064 10.1021/acs.orglett.0c02697

[83] P.‐Q. Huang , W. Ou , F. Han , Chem. Commun. 2016, 52, 11967–11970. 10.1039/c6cc05318a 27722243

[84] X.‐N. Hu , T.‐L. Shen , D.‐C. Cai , J.‐F. Zheng , P.‐Q. Huang , Org. Chem. Front. 2018, 5, 2051–2056.

[85] Z.‐P. Yang , G.‐S. Lu , J.‐L. Ye , P.‐Q. Huang , Tetrahedron 2019, 75, 1624–1631.

[86] C. L. Hugelshofer , V. Palani , R. Sarpong , J. Am. Chem. Soc. 2019, 141, 8431–8435. 31074980 10.1021/jacs.9b03576 PMC6750003

[87] C. L. Hugelshofer , V. Palani , R. Sarpong , J. Org. Chem. 2019, 84, 14069–14091. 31533427 10.1021/acs.joc.9b02223

[88] Y. A. Almehmadi , J. McGeehan , N. J. Guzman , K. E. Christensen , K. Yamazaki , D. J. Dixon , Nat. Synth. 2024, 3, 1168–1175.

[89] J. Pesti , G. L. Larson , Org. Process Res. Dev. 2016, 20, 1164–1181.

[90] IrCl(CO)(P[OCH(CF_3_)_2_]_3_)_2_ **Ir‐3** complex is available from Cortex Organics (www.cortexorganics.com).

[91] K. D. Collins , F. Glorius , Nat. Chem. 2013, 5, 597–601. 23787750 10.1038/nchem.1669

[92] A. Anfimov , S. Y. Erdyakov , M. Gurskii , Y. N. Bubnov , Russ. Chem. Bull. 2011, 60, 2336–2342.

