# Supplementary material for: Iridium‐Catalyzed Reductive Deoxygenation of Esters for the Synthesis of Sterically Hindered Ethers
Source: Angew Chem Int Ed Engl. 2025 Aug 26;64(40):e202508301. doi: 10.1002/anie.202508301 (PMC12462758; doi:10.1002/anie.202508301)
Supplement: Supplementary file 1 — Supporting Information [file ANIE-64-e202508301-s001.pdf]

Supporting Information

Iridium-Catalyzed Reductive Deoxygenation of Esters for the Synthesis of Sterically Hindered Ethers

Yaseen A. Almehmadi,[⊥‡] Anna J. Passmore,[⊥] Pablo Gabriel,[⊥] and Darren J. Dixon[⊥*]

[⊥] Department of Chemistry, University of Oxford, Chemistry Research Laboratory, 12 Mansfield Road, Oxford, OX1 3TA, UK

[‡] Department of Chemistry, Rabigh College of Arts and Sciences, King Abdulaziz University, Jeddah 21589, Saudi Arabia

Corresponding author email: darren.dixon@chem.ox.ac.uk

Table of Contents

1. General information 5

2. General procedures 6

General procedure A for the synthesis of esters 6

General procedure B for the synthesis of esters 6

General procedure C for the synthesis of esters 6

General procedure D for the synthesis of esters 7

General procedure E for the reduction of esters to ethers 7

General procedure F for the reduction of esters to ethers 7

General procedure G for the reduction of lactones to cyclic ethers 8

3. Synthesis of IrCl(CO)[P(HFIP)$_3$]$_2$ 8

Synthesis of IrCl(CO)[P(HFIP)$_3$]$_2$ from [IrCl(COE)$_2$]$_2$ 8

4. Optimisation 1

5. Synthesis of Esters 1

isopropyl 4-fluorobenzoate (7a) 1

tert-butyl 4-fluorobenzoate (7b) 1

phenyl 4-fluorobenzoate (7c) 2

4-methoxyphenyl 4-fluorobenzoate (7d) 2

(*3S,8R,9S,10R,13S,14S,17S*)-17-hydroxy-10,13,17-trimethyl-2,3,4,7,8,9,10,11,12,13,14,15,16,17-tetradecahydro-1H-cyclopenta[a]phenanthren-3-yl 4-fluorobenzoate (7e) 3

phenyl(trimethylsilyl)methyl 4-fluorobenzoate (7f) 3

(*1S,2R,5S*)-2-isopropyl-5-methylcyclohexyl 4-fluorobenzoate (7g) 4

1-fluoro-4-(((2-(phenylethynyl)cyclohexyl)oxy)methyl)benzene (7h) 5

butane-2,3-diyl bis(4-fluorobenzoate) (7i) 5

3-hydroxybutan-2-yl 4-fluorobenzoate (7j) 6

1-phenylethyl 4-fluorobenzoate (7k) 6

1-methylcyclobutyl 4-fluorobenzoate (7l) 7

1-methylcyclooctyl 4-fluorobenzoate (7m) 8

(3s,5s,7s)-adamantan-1-yl 4-fluorobenzoate (7n) 8

(3R,3aS,6R,7R,8aS)-6-((1-(4-fluorophenyl)vinyl)oxy)-3,6,8,8-tetramethyloctahydro-1H-3a,7-methanoazulene (7o) 9

isopropyl benzo[d][1,3]dioxole-5-carboxylate (7p) 9

isopropyl 4-methoxybenzoate (7q) 10

isopropyl 3-methoxybenzoate (7r) 10

isopropyl 2-methoxybenzoate (7s) 11

isopropyl 2-methylbenzoate (7t) 11

isopropyl 3-phenylpropanoate (7u) 11

isopropyl 4-(4-methoxyphenyl)butanoate (7v) 12

isopropyl 2-(1H-indol-3-yl)acetate (7w) 12

isopropyl cyclohexanecarboxylate (7x) 13

isopropyl (3r,5r,7r)-adamantane-1-carboxylate (7y) 13

isopropyl 1-phenylcyclopropane-1-carboxylate (7z) 14

isopropyl 1-phenylcyclobutane-1-carboxylate (7aa) 14

isopropyl 1-phenylcyclopentane-1-carboxylate (7ab) 15

isopropyl 1-phenylcyclohexane-1-carboxylate (7ac) 15

isopropyl 2-methyl-2-phenylpropcanoate (7ad) 16

tert-butyl 2-phenylacetate (7ae) 16

adamantan-1-yl adamantane-1-carboxylate (7af) 17

6. Synthesis of Ethers 18

1-fluoro-4-(isopropoxymethyl)benzene (8a) 18

1-(tert-butoxymethyl)-4-fluorobenzene (8b) 18

1-fluoro-4-(phenoxymethyl)benzene (8c) 19

1-fluoro-4-((4-methoxyphenoxy)methyl)benzene (8d) 19

(*3S,8R,9S,10R,13S,14S,17S*)-3-((4-fluorobenzyl)oxy)-10,13,17-trimethyl-2,3,4,7,8,9,10,11,12,13,14,15,16,17-tetradecahydro-1H-cyclopenta[a]phenanthren-17-ol (8e) 20

(((4-fluorobenzyl)oxy)(phenyl)methyl)trimethylsilane (8f) 20

1-fluoro-4-((((*1S,2R,5S*)-2-isopropyl-5-methylcyclohexyl)oxy)methyl)benzene (8g) 21

1-fluoro-4-(((2-(phenylethynyl)cyclohexyl)oxy)methyl)benzene (8h) 22

4,4'-((butane-2,3-diylbis(oxy))bis(methylene))bis(fluorobenzene) (8i) 22

3-((4-fluorobenzyl)oxy)butan-2-ol (8j) 23

1-fluoro-4-((1-phenylethoxy)methyl)benzene (8k) 24

1-fluoro-4-((1-methylcyclobutoxy)methyl)benzene(8l) 24

1-((4-fluorobenzyl)oxy)-1-methylcyclooctane (8m) 25

1-((4-fluorobenzyl)oxy)adamantane (8n) 25

(*3R,3aS,6R,7R,8aS*)-6-((4-fluorobenzyl)oxy)-3,6,8,8-tetramethyloctahydro-1H-3a,7-methanoazulene (8o) 26

5-(isopropoxymethyl)benzo[d][1,3]dioxole (8p) 27

1-(isopropoxymethyl)-4-methoxybenzene (8q) 27

1-(isopropoxymethyl)-3-methoxybenzene (8r) 28

1-(isopropoxymethyl)-2-methoxybenzene (8s) 28

1-(isopropoxymethyl)-2-methylbenzene (8t) 29

(3-isopropoxypropyl)benzene (8u) 29

1-(4-isopropoxybutyl)-4-methoxybenzene (8v) 29

3-(2-isopropoxyethyl)-1H-indole (8w) 30
(isopropoxymethyl)cyclohexane (8x) 30
1-(isopropoxymethyl)adamantane (8y) 31
(1-(isopropoxymethyl)cyclopropyl)benzene (8z) 31
(1-(isopropoxymethyl)cyclobutyl)benzene (8aa) 32
(1-(isopropoxymethyl)cyclopentyl)benzene (8ab) 32
(1-(isopropoxymethyl)cyclohexyl)benzene (8ac) 33
(1-isopropoxy-2-methylpropan-2-yl)benzene (8ad) 34
(2-(tert-butoxy)ethyl)benzene (8ae) 34
1-((adamantan-1-yl)methoxy)adamantane (8af) 35
2-ethoxyethyl)benzene (8ag) 35
(2-(benzyloxy)ethyl)benzene (8ah) 35
2-methyltetrahydro-2H-pyran (11a) 36
2-hexyltetrahydro-2H-pyran (11b) 36
(2S,3R,4S)-3,4-bis(benzyloxy)-2-((benzyloxy)methyl)tetrahydro-2H-pyran (11c) 37
Ambrox (4) 37
Eudesmin (11e) and Epieudesmin (11e') 38
7. Mechanistic experiments 39
8. Robustness Screen 40
9. Scale Up 41
10. References 42
11. NMR Spectra 42

1. General information

All reactions were performed using reagents/chemicals purchased from Sigma-Aldrich, Acros Organics, Alfa Aesar, STREM or Fluorochem without further purification unless otherwise stated. All water was purified through a Merck Millipore reverse osmosis purification system prior to use. Dichloromethane and diethyl ether were dried by filtration through activated alumina (powder ~150 mesh, pore size 58 Å, basic, Sigma-Aldrich) columns and stored under an atmosphere of N_2 prior to use. Anhydrous toluene was used as supplied from Acros Organics (99.7+%, Extra Dry over Molecular Sieve, AcroSeal®) and were sparged with N_2 prior to use. Dichloromethane was used as supplied. Deuterated solvents were used as supplied. Reactions were performed under a nitrogen atmosphere, unless otherwise stated. Temperatures quoted are external. Solvents were removed under reduced pressure using Büchi Rotavapor apparatus. NMR Spectra were measured on 400 MHz (^{1}H NMR at 400 MHz, ^{13}C NMR at 101 MHz, and ^{19}F NMR at 376 MHz) or Bruker 500 MHz (^{1}H NMR at 500 MHz, ^{13}C NMR at 126 MHz). Chemical shifts for ^{1}H NMR and ^{13}C were referenced based on the used deuterated solvent at: ^{1}H, 7.26 ppm, ^{13}C 77.16 ($CDCl_3$). Unless otherwise stated, 13C spectra are 1H decoupled and reported coupling constants for 13C spectra correspond to 19F–13C heteronuclear coupling NMR data are presented in the following format: chemical shift (δ) (multiplicity [app = apparent, br = broad, d = doublet, t = triplet, q = quartet, dd = doublet of doublets, dt = doublet of triplets, dq = doublet of quartets, ddt = doublet of doublet of triplets, ddd = doublet of doublet of doublets, m = multiplet], coupling constant [in Hz], number of equivalent nuclei by integration, assignment). The numbering of the compounds for assignment was made based on a synthetic point of view and do not follow the IUPAC nomenclature. High-resolution mass spectra (ESI) were recorded on Bruker μTOF mass spectrometer. Infrared spectra were recorded on a Bruker Tensor 27 FT-IR spectrometer as a thin film. Only selected maximum absorbances are reported (in *ν*max (cm^{-1})). Melting points were obtained on a Leica Galen III Hot-stage melting point apparatus and microscope and on a Kofler hot block and are reported uncorrected. Analytical thin-layer chromatography (TLC) was performed on Merck silica gel 60 F254 plates and visualised with UV light (254), and/or a vanillin stain or a $KMnO_4$ solution. Silica gel column chromatography was performed using 60 Å silica gel 40-63 μm purchased from VWR.

2. General procedures

General procedure A for the synthesis of esters

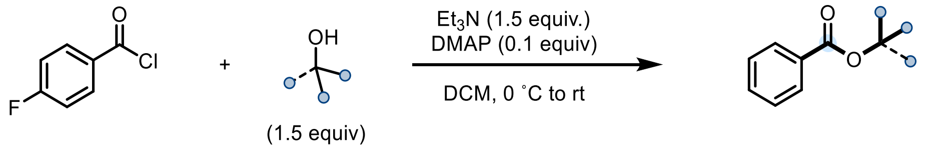

To a solution of the corresponding alcohol (1.5 equiv.), DMAP (0.1 equiv.), and triethylamine (1.5 equiv.) in CH_2Cl_2 (1.0 M), was slowly added *p*-fluorobenzoyl chloride (1 equiv.) at 0 °C. The reaction mixture was allowed to warm to rt and stirred until completion. The reaction mixture was diluted with water, extracted with CH_2Cl_2 three times and the combined organic phase was dried over $MgSO_4$. The organic phase was concentrated under reduced pressure, and the resulting crude material was purified by column chromatography to afford the desired ester.

General procedure B for the synthesis of esters

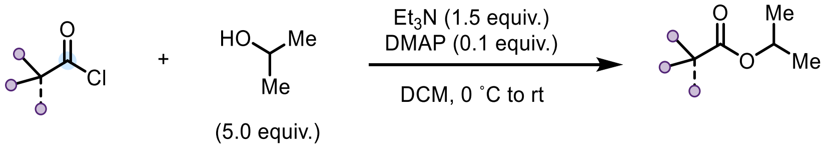

To a solution of isopropanol (5.0 equiv.), DMAP (0.1 equiv.), and triethylamine (1.5 equiv.) in CH_2Cl_2 (1.0 M), was slowly added the corresponding acid chloride (1 equiv.) at 0 °C. The reaction mixture was allowed to warm to rt and stirred until completion. The reaction mixture was diluted with water, extracted with CH_2Cl_2 three times and the combined organic phase was dried over $MgSO_4$. The organic phase was concentrated under reduced pressure, and the resulting crude material was purified by column chromatography to afford the desired ester.

General procedure C for the synthesis of esters

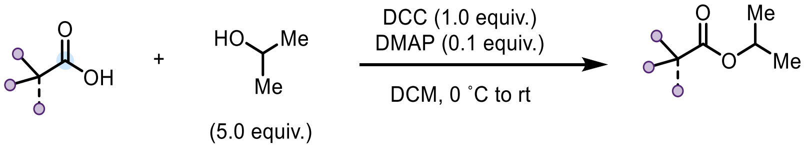

To a solution of carboxylic acid, DMAP (0.1 equiv.), and isopropanol (5.0 equiv.) in CH_2Cl_2 (1.0 M), was added DCC (1 equiv.) at 0 °C. The reaction mixture was allowed to warm to rt and stirred until completion. The reaction mixture was filtrated, and the filtrate was washed three times with

saturated aqueous $NaHCO_3$. The organic layer was dried over $MgSO_4$ before concentration under reduced pressure and purification by column chromatography to afford the desired ester.

General procedure D for the synthesis of esters

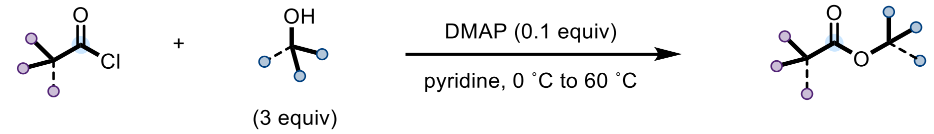

To a solution of isopropanol (3.0 equiv.) and DMAP (0.1 equiv.) in pyridine (1.0 M), was slowly added the corresponding acid chloride (1.0 equiv.) at 0 °C. The reaction mixture was allowed to warm to rt and stirred until completion. The reaction mixture was diluted with water, extracted with CH_2Cl_2 three times and the combined organic phase was dried over $MgSO_4$. The organic phase was concentrated under reduced pressure, and the resulting crude material was purified by column chromatography to afford the desired ester.

General procedure E for the reduction of esters to ethers

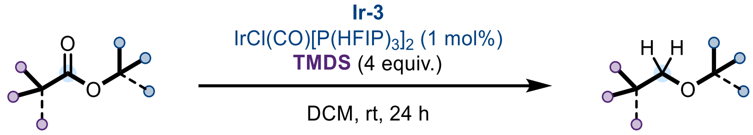

To a stirred solution of the relevant ester (0.20 mmol) and $IrCl(CO)[P(HFIP)_3]_2$ complex (2.60 mg, 1 mol%) in dry CH_2Cl_2 (1.0 M, 0.20 mL) in a 1.75 mL vial was added TMDS (0.80 mmol, 4 equiv.) at room temperature, which resulted in a gentle effervescence. The vial was purged with hydrogen gas before being capped and left to stir at rt for 24 h. The solvent was then evaporated in vacuo and the crude reaction mixture was purified by chromatography on silica gel (9:1 pentane/CH_2Cl_2).

General procedure F for the reduction of esters to ethers

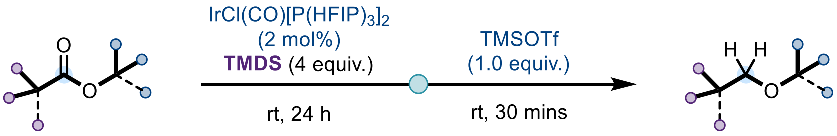

To a stirred solution of the relevant ester (0.20 mmol) and $IrCl(CO)[P(HFIP)_3]_2$ complex (5.20 mg, 2 mol%) in a 1.75 mL vial was added TMDS (0.80 mmol, 4 equiv.) at room temperature, which resulted in a gentle effervescence. The vial was purged with hydrogen gas before being capped and left to stir at rt for 24 h. TMSOTf (36.2 µL, 0.20 mmol, 1 equiv.) was then added at rt and the reaction stirred for a further 30 mins, after which the crude mixture was quenched with saturated aqueous $NaHCO_3$, extracted with diethyl ether, concentrated, and purified by column chromatography on silica gel (9:1 pentane/CH_2Cl_2).

General procedure G for the reduction of lactones to cyclic ethers

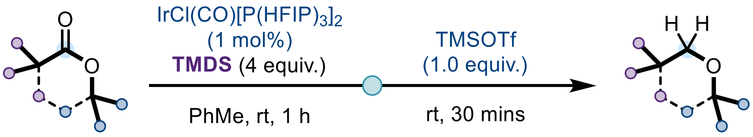

To a stirred solution of the relevant ester (0.20 mmol) and $IrCl(CO)[P(HFIP)_3]_2$ complex (2.60 mg, 1 mol%) in dry toluene (2 mL, 0.1 M) in a 1.75 mL vial was added TMDS (0.80 mmol, 4 equiv.) which resulted in a gentle effervescence. The vial was purged with hydrogen gas before being capped and left to stir at rt for 24 h. TMSOTf (36.2 μL, 0.20 mmol, 1 equiv.) was then added at rt and the reaction stirred for a further 30 mins, after which the crude mixture was quenched with saturated aqueous $NaHCO_3$, extracted with diethyl ether, concentrated, and purified by column chromatography on silica gel (9:1 pentane/CH_2Cl_2).

3. Synthesis of $IrCl(CO)[P(HFIP)_3]_2$

Synthesis of $IrCl(CO)[P(HFIP)_3]_2$ from $[IrCl(COE)_2]_2$

WARNING: this reaction must be performed with appropriate supervision, and a carbon monoxide alarm must be used at all times.

According to a modified literature procedure,[1] in a dry two-necked flask charged with $[IrCl(COE)_2]_2$ (179.2 mg, 0.20 mmol, 1 equiv.) was CH_3CN (100 mL, 0.002 M) added under a nitrogen atmosphere. The suspension was allowed to stir at rt for 5 minutes, after which carbon monoxide gas was introduced using a balloon and stirred for another 5 minutes, resulting in a homogeneous yellow solution. To this solution, tris(1,1,1,3,3,3-hexafluoro-2-propyl) phosphite (126 μL, 0.40 mmol, 4.0 equiv.) was added before an additional 15 minutes of stirring. The reaction mixture was concentrated to dryness, and the resulting yellow solid was washed with toluene (10 mL) to give $IrCl(CO)[P(HFIP)_3]_2$ as a yellow solid (242 mg, 0.186 mmol, 93 %). NMR spectra and physical properties are consistent with the literature.[2]

^{1}H NMR ($CDCl_3$, 400 MHz) δ_H: 5.8 – 5.4 (m, 6H)

^{19}F NMR ($CDCl_3$, 376 MHz) δ_F: -73.54.

^{31}P NMR ($CDCl_3$, 162 MHz) δ_F: 112.9.

4. Optimisation

Table S1: optimization of silane.

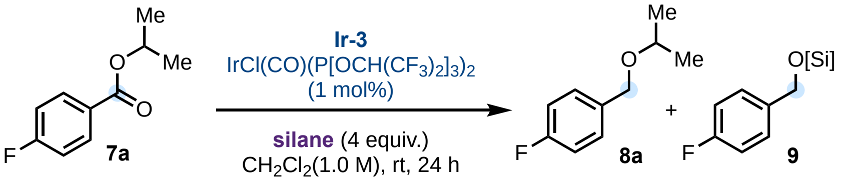

entry	silane (5 equiv.)	conv. %	ratio (%) 8a	ratio (%) 9
**1**	**TMDS**	**100**	**>95**	**5**
2	Et_3SiH	0	0	0
3	Ph_3SiH	0	0	0
4	PMHS	0	0	0
5	Et_2SiH_2	14	85	15
6	$(EtO)_3SiH$	<5	90	10

To a stirred solution of the relevant ester **7a** (0.20 mmol) and $IrCl(CO)[P(HFIP)_3]_2$ complex (2.60 mg, 1 mol%) in dry CH_2Cl_2 (1.0 M, 0.20 mL) in a 1.75 mL vial was added silane (0.80 mmol, 4 equiv.) at room temperature. The vial was purged with hydrogen gas before being capped and left to stir at rt for 24 h. The solvent was then evaporated in vacuo and the crude reaction mixture was purified by chromatography on silica gel (9:1 pentane/CH_2Cl_2).

5. Synthesis of Esters

isopropyl 4-fluorobenzoate (7a)

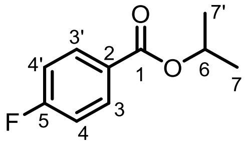

Prepared according to **General procedure A**. Purification via FCC (8 : 2 pentane/Et_2O) gave **7a** as a colourless liquid (783 mg, 4.30 mmol, 86%). The NMR spectra and physical properties are consistent with the literature.[3]

^{1}H NMR ($CDCl_3$, 400 MHz) δ_H: 8.09 – 8.00 (m, 2H, C^3H, $C^{3'}H$), 7.14 – 7.04 (m, 2H, C^4H, $C^{4'}H$), 5.24 (hept, *J* = 6.3 Hz, 1H, C^6H), 1.36 (d, *J* = 6.3 Hz, 7H, C^7H, $C^{7'}H$).

^{13}C NMR ($CDCl_3$, 101 MHz) δ_C: 165.8 (d, *J* = 253.2 Hz, C^5, $C^{5'}$), 165.3 (C^1), 132.1 (d, *J* = 9.1 Hz, C^3, $C^{3'}$), 127.3 (d, *J* = 3.1 Hz, C^2), 115.4 (d, *J* = 21.8 Hz, C^4, $C^{4'}$), 68.7 (C^6), 22.1 (C^7, $C^{7'}$).
^{19}F NMR ($CDCl_3$, 376 MHz) δ_F: -106.3 (tt, *J* = 8.5, 5.5 Hz).

tert-butyl 4-fluorobenzoate (7b)

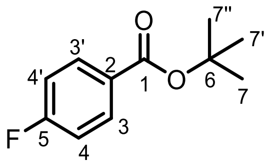

Prepared according to **General procedure A**. Purification via FCC (8 : 2 pentane/Et_2O) gave **7b** as a colourless liquid (833 mg, 4.25 mmol, 85%). The NMR spectra and physical properties are consistent with the literature.[4]

^{1}H NMR ($CDCl_3$, 400 MHz) δ_H: 8.04 – 7.95 (m, 2H, C^3H, $C^{3'}H$), 7.12 – 7.02 (m, 2H C^4H, $C^{4'}H$), 1.59 (s, 9H, C^7H, $C^{7'}H$, $C^{7''}H$).

^{13}C NMR ($CDCl_3$, 101 MHz) δ_C: 165.5 (d, *J* = 252.8 Hz, C^5, $C^{5'}$), 164.8 (C^1), 131.9 (d, *J* = 9.2 Hz, C^3, $C^{3'}$), 128.2 (d, *J* = 3.0 Hz, C^2), 115.2 (d, *J* = 21.9 Hz, C^4, $C^{4'}$), 81.2 (C^6), 28.2 (C^7, $C^{7'}$).
^{19}F NMR ($CDCl_3$, 376 MHz) δ_F: -106.9 (tt, *J* = 8.5, 5.5 Hz).

phenyl 4-fluorobenzoate (7c)

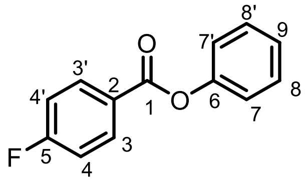

Prepared according to **General procedure D**. Purification via FCC (8 : 2 pentane/Et_2O) gave **7c** as a white solid (885 mg, 4.10 mmol, 82%). The NMR spectra and physical properties are consistent with the literature.[5]

^{1}H NMR ($CDCl_3$, 400 MHz) δ_H: 8.33 – 8.13 (m, 2H, C^{3}H, C$^{3'}$H), 7.50 – 7.38 (m, 2H, C^{4}H, C$^{4'}$H), 7.35 – 7.25 (m, 1H, C^{9}H), 7.22 (s, 4H, C^{7}H, C$^{7'}$H, C^{8}H, C$^{8'}$H).

^{13}C NMR ($CDCl_3$, 101 MHz) δ_C: 166.3 (d, *J* = 254.9 Hz, C^5, C$^{5'}$), 164.3 (C^1), 151.0 (C^6), 132.9 (d, *J* = 9.5 Hz, C^3, C$^{3'}$), 129.7 (C^7, C$^{7'}$), 126.1 (C^9), 126.0 (d, *J* = 3.0 Hz, C^2), 121.8 (C^8, C$^{8'}$), 115.9 (d, *J* = 22.1 Hz, C^4, C$^{4'}$).

^{19}F NMR ($CDCl_3$, 376 MHz) δ_F: -104.5 (tt, *J* = 8.4, 5.3 Hz)

4-methoxyphenyl 4-fluorobenzoate (7d)

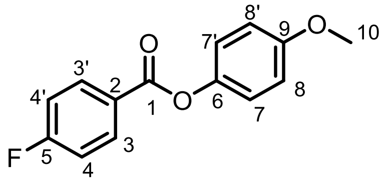

Prepared according to **General procedure D**. Purification via FCC (8 : 2 pentane/Et_2O) gave **7d** as a white solid (1.10 g, 4.50 mmol, 90%). The NMR spectra and physical properties are consistent with the literature.[6]

^{1}H NMR ($CDCl_3$, 400 MHz) δ_H: 8.26 – 8.17 (m, 2H, C^{3}H, C$^{3'}$H), 7.23 – 7.08 (m, 4H, C^{4}H, C$^{4'}$H, C^{7}H, C$^{7'}$H), 6.99 – 6.90 (m, 2H, C^{8}H, C$^{8'}$H), 3.83 (s, 3H, C^{10}H).

^{13}C NMR ($CDCl_3$, 101 MHz) δ_C: 166.2 (d, *J* = 254.9 Hz, C$^{5'}$), 164.7 (C^1), 157.5 (C^6), 144.4 (C^9), 132.9 (d, *J* = 9.4 Hz, C^3, C$^{3'}$), 126.0 (d, *J* = 3.1 Hz, C^2), 122.5 (C^7, C$^{7'}$), 115.9 (d, *J* = 21.9 Hz, C^4, C$^{4'}$), 114.7 (C^8, C$^{8'}$), 55.7 (C^{10}).

^{19}F NMR ($CDCl_3$, 376 MHz) δ_F: -104.6 (tt, *J* = 8.6, 2.7 Hz)

(*3S,8R,9S,10R,13S,14S,17S*)-17-hydroxy-10,13,17-trimethyl-2,3,4,7,8,9,10,11,12,13,14,15,16,17-tetradecahydro-1H-cyclopenta[a]phenanthren-3-yl 4-fluorobenzoate (7e)

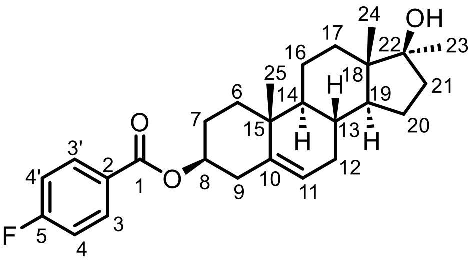

Prepared according to **General procedure A**. Purification via FCC (8 : 2 pentane/Et_2O) gave **7e** as a white solid (639 mg, 1.50 mmol, 30%).

IR 3375, 2979, 1722 (C=O), 1504, 1277, 1026.

^{1}H NMR ($CDCl_3$, 400 MHz) δ_H: 8.08 – 8.01 (m, 2H, $C^{3}H$, $C^{3'}H$), 7.10 (t, *J* = 8.7 Hz, 2H, $C^{4}H$, $C^{4'}H$), 5.42 (d, *J* = 4.7 Hz, 1H, $C^{11}H$), 4.90 – 4.79 (m, 1H, $C^{8}H$), 2.46 (br d, *J* = 7.8 Hz, 2H, $C^{9}H$), 2.09 – 1.96 (m, 2H, $C^{6}H$, $C^{12}H$), 1.93 (dt, *J* = 13.5, 3.6 Hz, 1H, $C^{21}H$), 1.89 – 1.80 (m, 1H, $C^{7}H$), 1.79 – 1.71 (m, 2H, $C^{6}H$, $C^{7}H$), 1.67 – 1.41 (m, 6H, $C^{12}H$, $C^{13}H$, $C^{16}H$, $C^{17}H$, $C^{20}H$, $C^{20'}H$), 1.36 – 1.17 (m, 8H, $C^{14}H$, $C^{16'}H$, $C^{17'}H$, $C^{21'}H$, $C^{23}H$), 1.09 (s, 3H, $C^{25}H$), 0.99 (ddd, *J* = 12.3, 10.4, 5.0 Hz, 1H, $C^{19}H$), 0.88 (s, 3H, $C^{24}H$).

^{13}C NMR ($CDCl_3$, 101 MHz) δ_C: 165.7 (d, *J* = 253.4 Hz, C^{5}), 165.1 (C^{1}), 139.7 (C^{10}), 132.1 (d, *J* = 9.1 Hz, C^{3}, $C^{3'}$), 127.0 (d, *J* = 2.9 Hz, C^{2}), 122.6 (C^{11}), 115.4 (d, *J* = 21.9 Hz, C^{4}, $C^{4'}$), 81.8 (C^{22}), 74.7 (C^{8}), 51.0 (C^{14}), 50.1 (C^{19}), 45.3 (C^{18}), 39.0 (C^{7}), 38.2 (C^{9}), 37.1 (C^{21}), 36.8 (C^{15}), 32.8 (C^{13}), 31.7 (C^{12}), 31.5 (C^{17}), 27.9 (C^{6}), 25.8 (C^{23}), 23.4 (C^{16}), 20.7 (C^{20}), 19.4 (C^{25}), 13.8 (C^{24}).

^{19}F NMR{^{1}H} ($CDCl_3$, 376 MHz) δ_F: -106.1.

HRMS (ES^+) exact mass calculated for $[M+H]^+$ ($C_{27}H_{36}FO_3$) requires **m/z** 427.2643, found **m/z** 427.2631.

Melting Point 201-202 °C.

phenyl(trimethylsilyl)methyl 4-fluorobenzoate (7f)

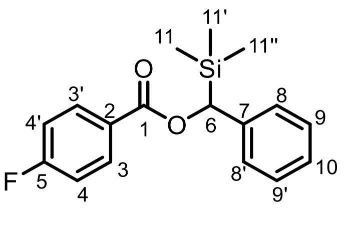

Prepared according to **General procedure A**. Purification via FCC (8 : 2 pentane/Et_2O) gave **7f** as a colourless liquid (453 mg, 1.50 mmol, 30%).

IR 2960, 2876, 1725 (C=O), 1507, 1256, 1109.

^{1}H NMR ($CDCl_3$, 400 MHz) δ_H: 8.18 – 8.06 (m, 2H, C^3H, $C^{3'}$H), 7.30 (td, *J* = 7.4, 1.4 Hz, 2H, C^4H, $C^{4'}$H), 7.24 – 7.06 (m, 5H, C^8H, $C^{8'}$H, C^9H, $C^{9'}$H, C^{10}H), 5.90 (s, 1H, C^6H), 0.09 (s, 9H, C^{11}H, $C^{11'}$H, $C^{11''}$H).

^{13}C NMR ($CDCl_3$, 101 MHz) δ_C: 167.2 (d, *J* = 244.5 Hz, C^5), 165.5 (C^1), 140.1 (C^7), 132.2 (d, *J* = 9.2 Hz, C^3, $C^{3'}$), 128.5 (C^9, $C^{9'}$), 127.1 (d, *J* = 3.3 Hz, C^2), 126.4 (C^{10}), 125.3 (C^8, $C^{8'}$), 115.7 (d, *J* = 22.1 Hz, C^4, $C^{4'}$), 72.5 (C^6), -3.6 (C^{11}, $C^{11'}$, $C^{11''}$).
^{19}F NMR ($CDCl_3$, 376 MHz) δ_F: -105.85 (tt, *J* = 8.5, 5.5 Hz)
HRMS (ES^+) exact mass calculated for $[M+Na]^+$ ($C_{17}H_{19}FO_2SiNa$) requires **m/z** 325.1031, found **m/z** 325.1029.

(*1S,2R,5S*)-2-isopropyl-5-methylcyclohexyl 4-fluorobenzoate (7g)

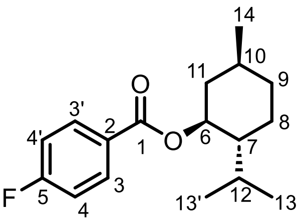

Prepared according to **General procedure A**. Purification via FCC (8 : 2 pentane/Et_2O) gave **7g** as a colourless liquid (1.17 g, 4.20 mmol, 84%). The NMR spectra and physical properties are consistent with the literature.[7]

^{1}H NMR ($CDCl_3$, 400 MHz) δ_H: 8.10 – 8.02 (m, 2H, C^3H, $C^{3'}$H), 7.14 – 7.06 (m, 2H, C^4H, $C^{4'}$H), 4.92 (td, *J* = 10.9, 4.4 Hz, 1H, C^6H), 2.16 – 2.08 (m, 1H, C^{11}H), 1.94 (heptd, *J* = 7.0, 2.8 Hz, 1H, C^{12}H), 1.77 – 1.68 (m, 2H, C^8H, C^9H), 1.62 – 1.49 (m, 2H, C^7H, C^{10}H), 1.19 – 1.03 (m, 2H, C^8H, $C^{11'}$H), 0.99 – 0.86 (m, 7H, $C^{9'}$H, C^{13}H, $C^{13'}$H), 0.79 (d, *J* = 6.9 Hz, 3H, C^{14}H).

^{13}C NMR ($CDCl_3$, 101 MHz) δ_C: 165.8 (d, *J* = 253.2 Hz, C^5), 165.3 (C^1), 132.2 (d, *J* = 9.4 Hz, C^3, $C^{3'}$), 127.2 (d, *J* = 3.1 Hz, C^2), 115.5 (d, *J* = 21.9 Hz, C^4, $C^{4'}$), 75.2 (C^6), 47.4 (C^7), 41.1 (C^{11}), 34.4 (C^9), 31.6 (C^{10}), 26.7 (C^{12}), 23.8 (C^8), 22.2 (C^{13}), 20.9 ($C^{13'}$), 16.7 (C^{14}).
^{19}F NMR{^{1}H} ($CDCl_3$, 376 MHz) δ_F: -106.3.

1-fluoro-4-(((2-(phenylethynyl)cyclohexyl)oxy)methyl)benzene (7h)

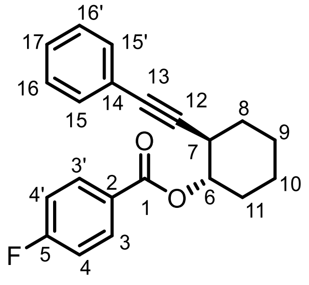

Prepared according to **General procedure A**. Purification via FCC (8 : 2 pentane/Et_2O) gave **7h** as a colourless liquid (1.53 g, 4.75 mmol, 95%).

IR 2941, 2863, 1717 (C=O), 1507, 1270, 1111.

^{1}H NMR ($CDCl_3$, 400 MHz) δ_H: 8.16 – 8.06 (m, 2H, C^3H, $C^{3'}$H), 7.34 – 7.27 (m, 2H, C^{16}H, $C^{16'}$H), 7.26 – 7.19 (m, 3H, C^{15}H, $C^{15'}$H, C^{17}H), 7.17 – 7.06 (m, 2H, C^4H, $C^{4'}$H), 5.13 (ddd, *J* = 8.4, 8.4, 3.7 Hz, 1H, C^6H), 2.89 (ddd, *J* = 9.3, 8.2, 4.0 Hz, 1H, C^7H), 2.26 – 2.07 (m, 2H, C^9H), 1.89 – 1.75 (m, 2H, C^{10}H, C^{11}H), 1.75 – 1.63 (m, 1H, C^8H), 1.63 – 1.46 (m, 2H, C^8H, $C^{11'}$H), 1.46 – 1.34 (m, 1H, $C^{10'}$H).

^{13}C NMR ($CDCl_3$, 101 MHz) δ_C: 165.9 (d, *J* = 253.5 Hz, C^5), 165.1 (C^1), 132.3 (d, *J* = 9.2 Hz, C^3, $C^{3'}$), 131.7 (C^{16}, $C^{16'}$), 128.3 (C^{17}, $C^{17'}$), 127.9 (C^{17}), 127.1 (d, *J* = 3.1 Hz, C^2), 123.6 (C^{14}), 115.6 (d, *J* = 22.0 Hz, C^4, $C^{4'}$), 90.2 (C^{12}), 82.4 (C^{13}), 75.2 (C^6), 35.2 (C^7), 30.3 (C^8, C^9), 24.0 (C^{10}), 23.4 (C^{11}).
^{19}F NMR ($CDCl_3$, 376 MHz) δ_F: -106.0 (tt, *J* = 8.5, 4.3 Hz).
HRMS (ES^+) exact mass calculated for $[M+Na]^+$ ($C_{21}H_{19}FO_2Na$) requires **m/z** 345.1261, found **m/z** 345.1269.

butane-2,3-diyl bis(4-fluorobenzoate) (7i)

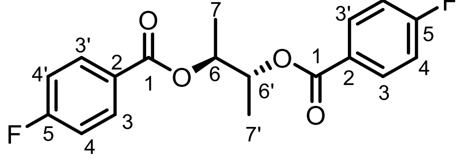

Prepared according to **General procedure A** using 1.0 equivalent of butane-2,3-diol and 2.0 equivalent of acid chloride. Purification via FCC (8 : 2 pentane/Et_2O) gave **7i** as a colourless oil (1.25 g, 3.75 mmol, 75%).

IR 3005, 2955, 1716 (2 x C=O), 1507, 1412, 1263, 1088.

^{1}H NMR ($CDCl_3$, 400 MHz) δ_H: 8.12 – 7.91 (m, 4H, C^3H, $C^{3'}$H), 7.18 – 6.97 (m, 4H, C^4H, $C^{4'}$H), 5.46 – 5.23 (m, 2H, C^6H, $C^{6'}$H), 1.46 – 1.35 (m, 6H, C^7H, $C^{7'}$H).

^{13}**C NMR** ($CDCl_3$, 101 MHz) δ_C: 165.8 (d, J = 254.2 Hz, C^5), 165.0 (C^1), 132.1 (d, J = 9.4 Hz, C^3, $C^{3'}$), 126.3 (d, J = 3.0 Hz, C^2), 115.5 (d, J = 22.1 Hz, C^4, $C^{4'}$), 72.5 (C^6, $C^{6'}$), 16.4 (C^7, $C^{7'}$).
^{19}**F NMR** ($CDCl_3$, 376 MHz) δ_F: -105.5 (tt, J = 8.5, 5.5 Hz)
HRMS (ES^+) exact mass calculated for $[M+Na]^+$ ($C_{18}H_{16}F_2O_4Na$) requires **m/z** 357.0909, found **m/z** 357.0910.

3-hydroxybutan-2-yl 4-fluorobenzoate (7j)

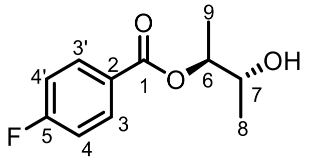

Prepared according to **General procedure A** using 2.0 equivalent of butane-2,3-diol and 1.0 equivalent of acid chloride. Purification via FCC (8 : 2 pentane/Et_2O) gave ***7J*** as a colourless liquid (721 mg, 3.40 mmol, 68%).

IR 3439, 2982, 1715 (C=O), 1508, 1449, 1272, 1091.

^{1}**H NMR** ($CDCl_3$, 400 MHz) δ_H: 8.09 – 7.99 (m, 2H, C^3H, $C^{3'}$H), 7.16 – 7.07 (m, 2H, C^4H, $C^{4'}$H), 5.02 (p, J = 6.3 Hz, 1H, C^6H), 3.90 (p, J = 6.3 Hz, 1H, C^7H), 2.03 (s, 1H, OH), 1.34 (d, J = 6.5 Hz, 3H, C^9H), 1.25 (d, J = 6.4 Hz, 3H, C^8H).

^{13}**C NMR** ($CDCl_3$, 101 MHz) δ_C: 166.0 (d, J = 254.2 Hz, C^5), 165.5 (C^1), 132.3 (d, J = 9.4 Hz, C^3, $C^{3'}$), 126.7 (d, J = 3.1 Hz, C^2), 115.7 (d, J = 22.0 Hz, C^4, $C^{4'}$), 75.7 (C^6), 70.3 (C^7), 19.2 (C^8), 16.4 (C^9).
^{19}**F NMR{^{1}H}** ($CDCl_3$, 376 MHz) δ_F: -105.5.
HRMS (ES^+) exact mass calculated for $[M+Na]^+$ ($C_{11}H_{13}FO_3Na$) requires **m/z** 235.0741, found **m/z** 235.0743.

1-phenylethyl 4-fluorobenzoate (7k)

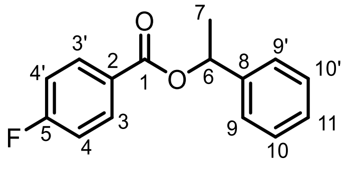

Prepared according to **General procedure A**. Purification via FCC (8 : 2 pentane/Et_2O) gave **7k** as a colourless liquid (1.01 g, 3.15 mmol, 83%).

IR 3035, 2986, 1719 (C=O), 1506, 1270.

^{1}H NMR ($CDCl_3$, 400 MHz) δ_H: 8.15 – 8.05 (m, 2H, $C^{10}H$, $C^{10'}H$), 7.48 – 7.42 (m, 2H, $C^{4}H$, $C^{4'}H$), 7.42 – 7.34 (m, 2H, $C^{3}H$, $C^{3'}H$), 7.34 – 7.27 (m, 1H, $C^{11}H$), 7.16 – 7.06 (m, 2H, $C^{9}H$, $C^{9'}H$), 6.13 (q, *J* = 6.6 Hz, 1H, $C^{6}H$), 1.68 (d, *J* = 6.6 Hz, 3H, $C^{7}H$).

^{13}C NMR ($CDCl_3$, 101 MHz) δ_C: 165.9 (d, *J* = 253.7 Hz, C^5), 165.0 (C^1), 141.8 (C^8), 132.3 (d, *J* = 9.2 Hz, C^3, $C^{3'}$), 128.7 (C^{10}, $C^{10'}$), 128.1 (C^{11}), 126.9 (d, *J* = 3.1 Hz, C^2), 126.2 (C^9, $C^{9'}$), 115.6 (d, *J* = 21.9 Hz, C^4, $C^{4'}$), 73.3 (C^6), 22.5 (C^7).
^{19}F NMR{^{1}H} ($CDCl_3$, 376 MHz) δ_F: -105.8.
HRMS (ES^+) exact mass calculated for $[M+Na]^+$ ($C_{15}H_{13}FO_2Na$) requires **m/z** 267.0792, found **m/z** 267.0787.

1-methylcyclobutyl 4-fluorobenzoate (71)

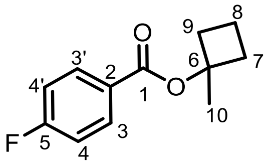

Prepared according to **General procedure A**. Purification via FCC (8 : 2 pentane/Et_2O) gave **71** as a colourless liquid (884 mg, 4.25 mmol, 85%).

IR 2991, 2949, 1718 (C=O), 1605, 1508, 1308.

^{1}H NMR ($CDCl_3$, 400 MHz) δ_H: 8.09 – 7.97 (m, 2H, $C^{3}H$, $C^{3'}H$), 7.14 – 7.04 (m, 2H, $C^{4}H$, $C^{4'}H$), 2.51 – 2.37 (m, 2H, $C^{7}H$, $C^{9}H$), 2.31 – 2.19 (m, 2H, $C^{7'}H$, $C^{9'}H$), 1.95 – 1.80 (m, 1H, $C^{8}H$), 1.80 – 1.68 (m, 1H, $C^{8'}H$), 1.65 (s, 3H, $C^{10}H$).

^{13}C NMR ($CDCl_3$, 101 MHz) δ_C: 165.7 (d, *J* = 252.9 Hz, C^5), 164.5 (C^1), 132.1 (d, *J* = 9.2 Hz, C^3, $C^{3'}$), 127.7 (d, *J* = 3.1 Hz, C^2), 115.5 (d, *J* = 21.9 Hz, C^4, $C^{4'}$), 80.8 (C^6), 35.7 (C^7, C^9), 23.4 (C^{10}), 14.0 (C^8).
^{19}F NMR ($CDCl_3$, 376 MHz) δ_F: -106.5 (tt, *J* = 8.4, 5.5 Hz).
HRMS (ES^+) exact mass calculated for $[M+H]^+$ ($C_{12}H_{14}F\ O_2$) requires **m/z** 209.0972, found **m/z** 209.0973.

1-methylcyclooctyl 4-fluorobenzoate (7m)

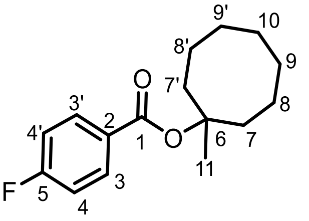

Prepared according to **General procedure A**. Purification via FCC (8 : 2 pentane/Et_2O) gave **7m** as a colourless liquid (1.10 g, 4.40 mmol, 88%).

IR 2928, 2857, 1715 (C=O), 1507, 1286, 1113.

^{1}H NMR ($CDCl_3$, 400 MHz) δ_H: 8.10 – 7.95 (m, 2H, C^{3}H, C$^{3'}$H), 7.12 – 7.03 (m, 2H, C^{4}H, C$^{4'}$H), 2.30 (ddd, *J* = 15.0, 9.1, 2.2 Hz, 2H, C^{7}H, C$^{7'}$H), 1.92 (ddd, *J* = 14.9, 9.0, 1.7 Hz, 2H, C^{7}H, C$^{7'}$H), 1.81 – 1.43 (m, 13H, C^{8}H, C$^{8'}$H, C^{9}H, C$^{9'}$H, C^{10}H, C^{11}H).

^{13}C NMR ($CDCl_3$, 101 MHz) δ_C: 165.6 (d, *J* = 252.8 Hz, C^{5}), 164.8 (C^{1}), 132.0 (d, *J* = 9.1 Hz, C^{3}, C$^{3'}$), 128.6 (d, *J* = 3.1 Hz, C^{2}), 115.4 (d, *J* = 21.9 Hz, C^{4}, C$^{4'}$), 87.2 (C^{6}), 35.4 (C^{7}, C$^{7'}$), 28.3 (C^{8}, C$^{8'}$), 26.2 (C^{11}), 25.1 (C^{10}), 22.3 (C^{9}, C$^{9'}$).

^{19}F NMR ($CDCl_3$, 376 MHz) δ_F: -107.0 (tt, *J* = 8.5, 5.5 Hz).

HRMS (ES^{+}) exact mass calculated for $[M+H]^+$ ($C_{15}H_{20}FO_2$) requires **m/z** 1251.1442, found **m/z** 251.1439.

(3s,5s,7s)-adamantan-1-yl 4-fluorobenzoate (7n)

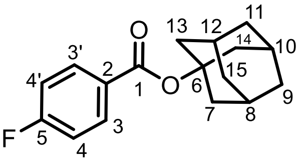

Prepared according to **General procedure D**. Purification via FCC (8 : 2 pentane/Et_2O) gave **7n** as a colourless liquid (1.04 g, 3.80 mmol, 76%). The NMR spectra and physical properties are consistent with the literature.[8]

^{1}H NMR ($CDCl_3$, 400 MHz) δ_H: 8.03 – 7.95 (m, 2H, C^{3}H, C$^{3'}$H), 7.11 – 7.03 (m, 2H, C^{4}H, C$^{4'}$H), 2.29 – 2.19 (m, 9H, C^{7}H, C^{8}H, C^{10}H, C^{12}H, C^{13}H, C^{15}H), 1.76 – 1.66 (m, 6H, C^{9}H, C^{11}H, C^{14}H).

^{13}C NMR ($CDCl_3$, 101 MHz) δ_C: 165.6 (d, *J* = 252.9 Hz, C^{5}), 164.6 (C^{1}), 132.0 (d, *J* = 9.2 Hz, C^{3}, C$^{3'}$), 128.5 (d, *J* = 3.0 Hz, C^{2}), 115.3 (d, *J* = 21.9 Hz, C^{4}, C$^{4'}$), 81.4 (C^{6}), 41.6 (C^{7}, C^{13}, C^{15}), 36.4 (C^{9}, C^{11}, C^{14}), 31.0 (C^{8}, C^{10}, C^{12}).

^{19}**F NMR{^{1}H}** ($CDCl_3$, 376 MHz) δ_F: -106.97.

(3R,3aS,6R,7R,8aS)-6-((1-(4-fluorophenyl)vinyl)oxy)-3,6,8,8-tetramethyloctahydro-1H-3a,7-methanoazulene (7o)

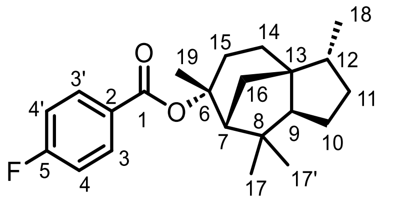

Prepared according to **General procedure D**. Purification via FCC (8 : 2 pentane/Et_2O) gave **7o** as a white solid (688 mg, 2.0 mmol, 40%).

IR 2961, 1701 (C=O), 1507,1457, 1292.

^{1}**H NMR** ($CDCl_3$, 400 MHz) δ_H: 8.04 – 7.96 (m, 2H, C^3H, $C^{3'}H$), 7.12 – 7.04 (m, 2H, C^4H, $C^{4'}H$), 2.62 (d, *J* = 5.2 Hz, 1H, C^7H), 2.20 – 2.08 (m, 2H, $C^{15}H$), 1.94 – 1.82 (m, 2H, C^9H, $C^{11}H$), 1.77 – 1.64 (m, 5H, $C^{12}H$, $C^{16}H$, $C^{19}H$), 1.60 – 1.45 (m, 3H, $C^{10}H$, $C^{14}H$), 1.45 – 1.36 (m, 2H, $C^{10'}H$, $C^{16'}H$), 1.35 – 1.25 (m, 1H, $C^{11'}H$), 1.13 (s, 3H, $C^{17}H$), 0.99 (s, 3H, $C^{17'}H$), 0.86 (d, *J* = 7.0 Hz, 3H, $C^{18}H$).

^{13}**C NMR** ($CDCl_3$, 101 MHz) δ_C: 165.4 (d, *J* = 252.6 Hz, C^5), 164.6 (C^1), 132.0 (d, *J* = 9.2 Hz, C^3, $C^{3'}$), 128.4 (d, *J* = 3.0 Hz, C^2), 115.2 (d, *J* = 21.8 Hz, C^4, $C^{4'}$), 87.5 (C^6), 56.8 (C^7), 56.8 (C^9), 54.0 (C^{13}), 43.5 (C^8), 41.3 (C^{12}), 41.1 (C^{16}), 37.0 (C^{15}), 33.4 (C^{11}), 31.3 (C^{14}), 28.5 (C^{19}), 27.3 (C^{17}), 26.2 (C^{10}), 25.3 ($C^{17'}$), 15.6 (C^{18}).

^{19}**F NMR** ($CDCl_3$, 376 MHz) δ_F: -107.1 (tt, *J* = 8.5, 5.5 Hz).

HRMS (ES^+) exact mass calculated for $[M+H]^+$ ($C_{22}H_{29}FO_2Na$) requires **m/z** 367.2044, found **m/z** 267.2038.

isopropyl benzo[d][1,3]dioxole-5-carboxylate (7p)

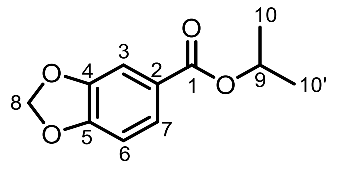

Prepared according to **General procedure C**. Purification via FCC (8 : 2 pentane/EtOAc) gave **7p** as a colourless oil (853 mg, 4.10 mmol, 82%). The NMR spectra and physical properties are consistent with the literature.[9]

^{1}**H NMR** ($CDCl_3$, 400 MHz) δ_H: 7.64 (dd, J = 8.2, 1.7 Hz, 1H, C^7H), 7.46 (d, J = 1.7 Hz, 1H, C^3H), 6.82 (d, J = 8.2 Hz, 1H, C^6H), 6.02 (s, 2H, C^8H), 5.21 (hept, J = 6.3 Hz, 1H, C^9H), 1.34 (d, J = 6.2 Hz, 7H, $C^{10}H$, $C^{10'}H$).

^{13}**C NMR** ($CDCl_3$, 101 MHz) δ_C: 165.6 (C^1), 151.5 (C^4), 147.8 (C^5), 125.3 (C^7), 125.2 (C^2), 109.6 (C^3), 108.0 (C^6), 101.8 (C^8), 68.4 (C^9), 22.1 (C^{10}, $C^{10'}$).

isopropyl 4-methoxybenzoate (7q)

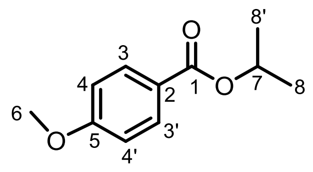

Prepared according to **General procedure C**. Purification via FCC (8 : 2 pentane/Et_2O) gave **7q** as a colourless liquid (786 mg, 4.05 mmol, 81%). The NMR spectra and physical properties are consistent with the literature.[10]

^{1}**H NMR** ($CDCl_3$, 400 MHz) δ_H: 8.04 – 7.94 (m, 2H, C^3H, $C^{3'}H$), 6.95 – 6.87 (m, 2H, C^4H, $C^{4'}H$), 5.22 (hept, J = 6.2 Hz, 1H, C^7H), 3.85 (s, 3H, C^6H), 1.35 (d, J = 6.3 Hz, 6H, C^8H, $C^{8'}H$).

^{13}**C NMR** ($CDCl_3$, 101 MHz) δ_C: 166.0 (C^1), 163.3 (C^5), 131.6 (C^3, $C^{3'}$), 123.6 (C^2), 113.6 (C^4, $C^{4'}$), 68.1 (C^7), 55.5 (C^6), 22.1 (C^8, $C^{8'}$).

isopropyl 3-methoxybenzoate (7r)

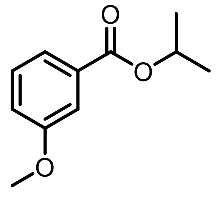

Prepared according to **General procedure C.** Purification via FCC (8 : 2 pentane/ Et_2O) gave **7r** as a colourless liquid (75.1 mg, 0.39 mmol, 39%). The NMR spectra and physical properties are consistent with the literature.[11]

^{1}**H NMR** (400 MHz, $CDCl_3$) δ 7.63 (ddd, J = 7.7, 1.5, 1.0 Hz, 1H), 7.56 (dd, J = 2.8, 1.5 Hz, 1H), 7.33 (t, J = 7.9 Hz, 1H), 7.08 (ddd, J = 8.3, 2.7, 1.0 Hz, 1H), 5.25 (hept, J = 6.3 Hz, 1H), 3.85 (s, 3H), 1.36 (d, J = 6.3 Hz, 6H).

^{13}**C NMR** (101 MHz, $CDCl_3$) δ 166.1, 159.6, 132.4, 129.4, 122.0, 119.2, 114.2, 68.6, 55.5, 22.0.

isopropyl 2-methoxybenzoate (7s)

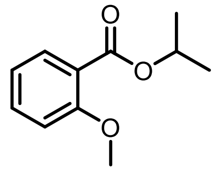

Prepared according to **General procedure C.** Purification via FCC (8 : 2 pentane/ Et_2O) gave **7s** as a colourless liquid (114.3 mg, 0.59 mmol, 59%). The NMR spectra and physical properties are consistent with the literature.[11]

^{1}H NMR (400 MHz, $CDCl_3$) δ 7.75 (dd, *J* = 7.8, 1.8 Hz, 1H), 7.45 (ddd, *J* = 8.4, 7.4, 1.8 Hz, 1H), 7.01 – 6.92 (m, 2H), 5.25 (hept, *J* = 6.5 Hz, 1H), 3.90 (s, 3H), 1.36 (d, *J* = 6.3 Hz, 6H).

^{13}C NMR (101 MHz, $CDCl_3$) δ 165.8, 159.2, 133.3, 131.4, 121.1, 120.2, 112.2, 68.2, 56.1, 22.1.

isopropyl 2-methylbenzoate (7t)

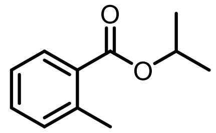

Prepared according to **General procedure C.** Purification via FCC (8 : 2 pentane/ Et_2O) gave **7t** as a colourless liquid (110.5 mg, 0.62 mmol, 62%). The NMR spectra and physical properties are consistent with the literature.[12]

^{1}H NMR (400 MHz, $CDCl_3$) δ 7.90 (dd, *J* = 8.2, 1.6 Hz, 1H), 7.39 (td, *J* = 7.4, 1.5 Hz, 1H), 7.30 – 7.21 (m, 2H), 5.27 (hept *J* = 6.1 Hz, 1H), 2.62 (s, 3H), 1.39 (d, *J* = 6.3 Hz, 6H).

^{13}C NMR (101 MHz, $CDCl_3$) δ 167.41, 139.84, 131.75, 131.68, 130.59, 130.48, 125.74, 68.21, 22.08, 21.78.

isopropyl 3-phenylpropanoate (7u)

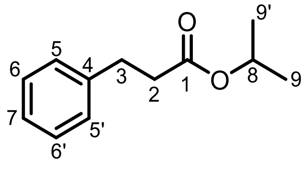

Prepared according to **General procedure C**. Purification via FCC (8 : 2 pentane/Et_2O) gave **7u** as a colourless liquid (768 mg, 4.00 mmol, 80%). The NMR spectra and physical properties are consistent with the literature.[13]

^{1}H NMR ($CDCl_3$, 400 MHz) δ_H: 7.34 – 7.25 (m, 2H, $C^{6}H$, $C^{6'}H$), 7.24 – 7.16 (m, 3H, $C^{5}H$, $C^{5'}H$, $C^{7}H$), 5.02 (hept, J = 6.3 Hz, 1H, $C^{8}H$), 2.96 (t, J = 7.8 Hz, 2H, $C^{2}H$), 2.61 (t, J = 7.2 Hz, 2H, $C^{3}H$), 1.22 (d, J = 6.2 Hz, 6H, $C^{9}H$, $C^{9'}H$).

^{13}C NMR ($CDCl_3$, 101 MHz) δ_C: 172.4 (C^{1}), 140.6 (C^{4}), 128.5 (C^{5}, $C^{5'}$), 128.3 (C^{6}, $C^{6'}$), 126.2 (C^{7}). 67.7 (C^{8}), 36.3 (C^{2}), 31.1 (C^{3}), 21.8 (C^{9}, $C^{9'}$).

isopropyl 4-(4-methoxyphenyl)butanoate (7v)

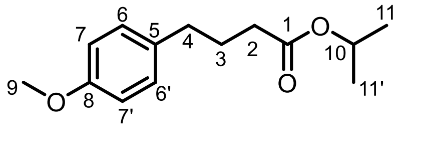

Prepared according to **General procedure C**. Purification via FCC (8 : 2 pentane/Et_2O) gave **7v** as a colourless liquid (1.05 g, 4.45 mmol, 89%).

IR 2970, 2934, 2858, 1512 (C=O), 1464, 1367.

^{1}H NMR ($CDCl_3$, 400 MHz) δ_H: 7.14 – 7.05 (m, 2H, $C^{7}H$, $C^{7'}H$), 6.87 – 6.79 (m, 2H, $C^{6}H$, $C^{6'}H$), 5.01 (hept, J = 6.3 Hz, 1H, $C^{10}H$), 3.79 (s, 3H, $C^{9}H$), 2.59 (t, J = 7.8 Hz, 2H, $C^{4}H$), 2.28 (t, J = 7.5 Hz, 2H, $C^{2}H$), 1.97 – 1.85 (m, 2H, $C^{3}H$), 1.23 (d, J = 6.3 Hz, 6H, $C^{11}H$, $C^{11'}H$).

^{13}C NMR ($CDCl_3$, 101 MHz) δ_C: 173.2 (C^{1}), 158.0 (C^{8}), 133.7 (C^{5}), 129.5 (C^{6}, $C^{6'}$), 113.9 (C^{7}, $C^{7'}$), 67.6 (C^{10}), 55.4 (C^{9}), 34.4 (C^{4}), 34.1 (C^{2}), 27.0 (C^{3}), 22.0 (C^{11}, $C^{11'}$).

HRMS (ES^{+}) exact mass calculated for $[M+Na]^{+}$ ($C_{14}H_{20}O_3Na$) requires **m/z** 259.1305, found **m/z** 259.1309.

Melting Point 195-193 °C.

isopropyl 2-(1H-indol-3-yl)acetate (7w)

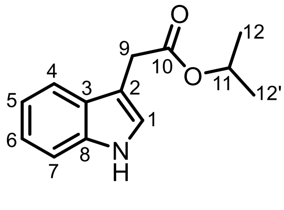

Prepared according to **General procedure C**. Purification via FCC (8 : 2 pentane/EtOAc) gave **7w** as a brown oil (955 mg, 4.40 mmol, 88%). The NMR spectra and physical properties are consistent with the literature.[14]

^{1}H NMR ($CDCl_3$, 400 MHz) δ_H: 8.09 (br s, 1H, NH), 7.64 (d, J = 0.7 Hz, 1H, C^4H), 7.34 (d, J = 0.9 Hz, 1H, C^7H), 7.20 (ddd, J = 8.2, 7.0, 1.3 Hz, 1H, C^5H), 7.17 – 7.11 (m, 2H, C^1H, C^6H), 5.06 (hept, J = 6.3 Hz, 1H, $C^{11}H$), 3.75 (s, 2H, C^9H), 1.25 (dd, J = 6.3, 1.0 Hz, 6H, $C^{12}H$, $C^{12'}H$).

^{13}C NMR ($CDCl_3$, 101 MHz) δ_C: 171.8 (C^{10}), 136.3 (C^8), 127.4 (C^3), 123.1 (C^1), 122.3 (C^5), 119.7 (C^6), 119.1 (C^4), 111.3 (C^7), 108.9 (C^2), 68.3 (C^{11}), 31.9 (C^9), 22.0 (C^{12}, $C^{12'}$).

isopropyl cyclohexanecarboxylate (7x)

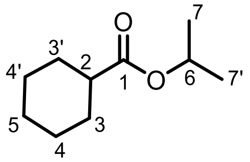

Prepared according to **General procedure B**. Purification via FCC (8 : 2 pentane/Et_2O) gave **7x** as a colourless liquid (774 mg, 4.55 mmol, 91%). The NMR spectra and physical properties are consistent with the literature.[15]

^{1}H NMR ($CDCl_3$, 400 MHz) δ_H: 4.98 (hept, J = 6.3 Hz, 1H, C^6H), 2.23 (tt, J = 11.2, 3.6 Hz, 1H, C^2H), 1.93 – 1.81 (m, 2H, C^3H, $C^{3'}H$), 1.80 – 1.67 (m, 2H, C^4H, $C^{4'}H$), 1.67 – 1.56 (m, 1H, C^5H), 1.50 – 1.34 (m, 2H, C^3H, $C^{3'}H$), 1.33 – 1.15 (m, 9H, C^5H, C^7H, $C^{7'}H$).

^{13}C NMR ($CDCl_3$, 101 MHz) δ_C: 175.7 (C^1), 67.0 (C^6), 43.4 (C^2), 29.0 (C^3, $C^{3'}$), 25.8 (C^5), 25.5 (C^4, $C^{4'}$), 21.8 (C^7, $C^{7'}$).

isopropyl (3r,5r,7r)-adamantane-1-carboxylate (7y)

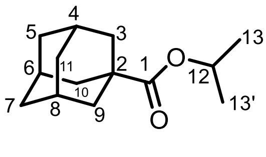

Prepared according to **General procedure B**. Purification via FCC (8 : 2 pentane/Et_2O) gave **7y** as a white solid (932 mg, 4.20 mmol, 84%). The NMR spectra and physical properties are consistent with the literature.[16]

^{1}H NMR ($CDCl_3$, 400 MHz) δ_H: 4.96 (p, J = 6.3 Hz, 1H, $C^{12}H$), 2.00 (p, J = 3.0 Hz, 3H, C^4H, C^6H, C^8H), 1.86 (d, J = 2.9 Hz, 6H, C^3H, C^9H, $C^{10}H$), 1.77 – 1.63 (m, 6H, C^5H, C^7H, $C^{11}H$), 1.20 (d, J = 6.3 Hz, 6H, $C^{13}H$, $C^{13'}H$).

^{13}C NMR ($CDCl_3$, 101 MHz) δ_C: 177.4 (C^1), 67.0 (C^{12}), 38.9 (C^3, C^9, C^{10}), 36.7 (C^5, C^7, C^{11}), 28.2 (C^3, C^9, C^{10}), 21.9 (C^{13}, $C^{13'}$).

isopropyl 1-phenylcyclopropane-1-carboxylate (7z)

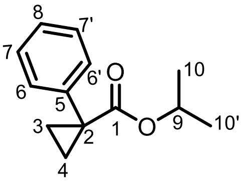

Prepared according to **General procedure C**. Purification via FCC (8 : 2 pentane/Et_2O) gave **7z** as a colourless liquid (847 mg, 4.15 mmol, 83%).

IR 2958, 2873, 1715 (C=O), 1505, 1288, 1153.

^{1}H NMR ($CDCl_3$, 400 MHz) δ_H: 7.40 – 7.12 (m, 5H, $C^{6}H$, $C^{6'}H$, $C^{7}H$, $C^{7'}H$, $C^{8}H$), 4.96 (hept, *J* = 6.2 Hz, 1H, $C^{9}H$), 1.58 (q, *J* = 3.9 Hz, 2H, $C^{2}H$, $C^{3}H$, $C^{4}H$), 1.24 – 1.09 (m, 8H, $C^{3}H$, $C^{4}H$, $C^{10}H$, $C^{10'}H$).

^{13}C NMR ($CDCl_3$, 101 MHz) δ_C: 174.1 (C^{1}), 140.0 (C^{5}), 130.6 (C^{7}, $C^{7'}$), 128.1 (C^{6}, $C^{6'}$), 127.1 (C^{8}), 68.3 (C^{9}), 29.4 (C^{2}), 21.8 (C^{3}, C^{4}), 16.3 (C^{10}).

HRMS (ES^{+}) exact mass calculated for $[M+Na]^{+}$ ($C_{13}H_{16}O_2Na$) requires **m/z** 227.1043, found **m/z** 227.1040.

isopropyl 1-phenylcyclobutane-1-carboxylate (7aa)

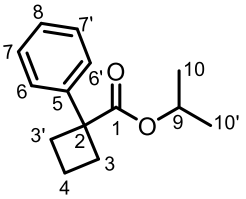

Prepared according to **General procedure C**. Purification via FCC (8 : 2 pentane/Et_2O) gave **7aa** as a colourless liquid (828 mg, 3.80 mmol, 76%).

IR 2980, 2937, 2863, 1723 (C=O), 1503, 1220, 1107.

^{1}H NMR ($CDCl_3$, 400 MHz) δ_H: 7.70 – 6.92 (m, 5H, $C^{6}H$, $C^{6'}H$, $C^{7}H$, $C^{7'}H$, $C^{8}H$), 4.91 (hept, *J* = 6.3 Hz, 1H, $C^{9}H$), 2.83 – 2.71 (m, 2H, $C^{3}H$, $C^{3'}H$), 2.52 – 2.39 (m, 2H, $C^{3}H$, $C^{3'}H$), 2.05 – 1.75 (m, 2H, $C^{4}H$), 1.10 (d, *J* = 6.2 Hz, 6H, $C^{10}H$, $C^{10'}H$).

^{13}C NMR ($CDCl_3$, 101 MHz) δ_C: 175.6 (C^{1}), 144.1 (C^{5}), 128.2 (C^{7}, $C^{7'}$), 126.5 (C^{8}), 126.3, (C^{6}, $C^{6'}$), 68.1 (C^{9}), 52.7 (C^{2}), 32.4 (C^{3}), 21.7 (C^{10}, $C^{10'}$), 16.7 (C^{4}).

HRMS (ES$^+$) exact mass calculated for $[M+Na]^+$ ($C_{14}H_{18}O_2Na$) requires **m/z** 241.1199, found **m/z** 241.1202.

isopropyl 1-phenylcyclopentane-1-carboxylate (7ab)

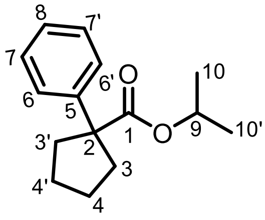

Prepared according to **General procedure C**. Purification via FCC (8 : 2 pentane/Et_2O) gave **7ab** as a colourless liquid (940 mg, 4.05 mmol, 81%).

IR 2979,2876, 1723 (C=O), 1503, 1259, 1110.

^{1}H NMR ($CDCl_3$, 400 MHz) δ_H: 7.39 – 7.34 (m, 2H, C^7H, $C^{7'}H$), 7.29 (ddd, *J* = 7.7, 6.8, 1.2 Hz, 2H, C^6H, $C^{6'}H$), 7.25 – 7.17 (m, 1H, C^8H), 4.93 (hept, *J* = 6.3 Hz, 1H, C^9H), 2.73 – 2.59 (m, 2H, C^3H, $C^{3'}H$), 1.95 – 1.80 (m, 2H, C^3H, $C^{3'}H$), 1.80 – 1.64 (m, 4H, C^4H, $C^{4'}H$), 1.12 (d, *J* = 6.3 Hz, 6H, $C^{10}H$, $C^{10'}H$).

^{13}C NMR ($CDCl_3$, 101 MHz) δ_C: 175.5 (C^1), 143.7 (C^5), 128.2 (C^7, $C^{7'}$), 127.0 (C^6, $C^{6'}$), 126.6 (C^8), 68.0 (C^9), 59.4 (C^2), 36.2 (C^3, $C^{3'}$), 23.7 (C^4, $C^{4'}$), 21.6 (C^{10}, $C^{10'}$).

HRMS (ES$^+$) exact mass calculated for $[M+Na]^+$ ($C_{15}H_{20}O_2Na$) requires **m/z** 255.1356, found **m/z** 255.1356.

isopropyl 1-phenylcyclohexane-1-carboxylate (7ac)

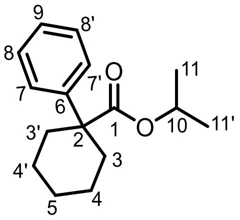

Prepared according to **General procedure C**. Purification via FCC (8 : 2 pentane/Et_2O) gave **7ac** as a colourless liquid (775 mg, 3.15 mmol, 63%).

IR 2937, 2863, 1723 (C=O), 1503, 1358, 1219.

^{1}H NMR ($CDCl_3$, 400 MHz) δ_H: 7.43 – 7.37 (m, 2H, C^7H, $C^{7'}H$), 7.35 – 7.28 (m, 2H, C^8H, $C^{8'}H$), 7.25 – 7.18 (m, 1H, C^9H), 5.00 (hept, *J* = 6.3 Hz, 1H, $C^{10}H$), 2.55 – 2.45 (m, 2H, C^3H, $C^{3'}H$), 1.77

– 1.57 (m, 5H, $C^{3}H$, $C^{3'}H$, $C^{4}H$, $C^{4'}H$, $C^{5}H$), 1.56 – 1.41 (m, 2H, $C^{4}H$, $C^{4'}H$), 1.35 – 1.20 (m, 1H, $C^{5'}H$), 1.15 (d, J = 6.3 Hz, 5H, $C^{11}H$, $C^{11'}H$).

^{13}C NMR ($CDCl_3$, 101 MHz) δ_C: 174.6 (C^{1}), 144.3 (C^{6}), 128.4 (C^{8}, $C^{8'}$), 126.7 (C^{9}), 126.0 (C, $C^{7'}$), 67.8 (C^{12}), 50.9 (C^{2}), 34.8 (C^{3}, $C^{3'}$), 25.8 (C^{5}), 23.9 (C^{4}, $C^{4'}$), 21.7 (C^{11}, $C^{11'}$).
HRMS (ES^{+}) exact mass calculated for $[M+Na]^{+}$ ($C_{16}H_{22}O_2Na$) requires **m/z** 269.1512, found **m/z** 269.1515.

isopropyl 2-methyl-2-phenylpropcanoate (7ad)

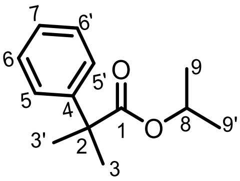

Prepared according to **General procedure C**. Purification via FCC (8 : 2 pentane/Et_2O) gave **7ad** as a colourless liquid (783 mg, 3.80 mmol, 76%).

IR 2983, 2877, 1724 (C=O), 1503, 1261, 1111.

^{1}H NMR ($CDCl_3$, 400 MHz) δ_H: 7.37 – 7.27 (m, 4H, $C^{5}H$, $C^{5'}H$, $C^{6}H$, $C^{6'}H$), 7.25 – 7.19 (m, 1H, $C^{7}H$), 5.00 (hept, J = 6.3 Hz, 1H, $C^{8}H$), 1.56 (s, 6H, $C^{3}H$, $C^{3'}H$), 1.16 (d, J = 6.3 Hz, 6H, $C^{9}H$, $C^{9'}H$).

^{13}C NMR ($CDCl_3$, 101 MHz) δ_C: 176.2 (C^{1}), 145.0 (C^{4}), 128.2 (C^{6}, $C^{6'}$), 126.5 (C^{7}), 125.6 (C^{5}, $C^{5'}$), 67.9 (C^{8}), 46.5 (C^{2}), 26.5 (C^{3}, $C^{3'}$), 21.5 (C^{9}, $C^{9'}$).
HRMS (ES^{+}) exact mass calculated for $[M+Na]^{+}$ ($C_{13}H_{18}O_2Na$) requires **m/z** 229.1199, found **m/z** 229.1193.

tert-butyl 2-phenylacetate (7ae)

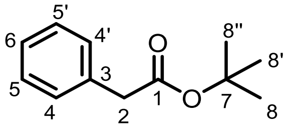

Prepared according to **General procedure B**. Purification via FCC (8 : 2 pentane/Et_2O) gave **7ae** as a colourless liquid (883 mg, 4.60 mmol, 92%).

IR 3032, 2980, 1734 (C=O), 1417, 1143.

^{1}**H NMR** ($CDCl_3$, 400 MHz) δ_H: 7.37 – 7.21 (m, 5H, C^4H, $C^{4'}H$, C^5H, $C^{5'}H$, C^6H), 3.53 (s, 2H, C^2H), 1.47 – 1.42 (m, 9H, C^8H, $C^{8'}H$, $C^{8''}H$).

^{13}**C NMR** ($CDCl_3$, 101 MHz) δ_C: 171.1 (C^1), 134.9 (C^3), 129.3 (C^5, $C^{5'}$), 128.6 (C^4, $C^{4'}$), 126.9 (C^6), 80.9 (C^7), 42.8 (C^2), 28.2 (C^8, C^8, $C^{8''}$).
HRMS (ES^+) exact mass calculated for $[M+Na]^+$ ($C_{12}H_{16}O_2Na$) requires **m/z** 215.1043, found **m/z** 215.1036.

adamantan-1-yl adamantane-1-carboxylate (7af)

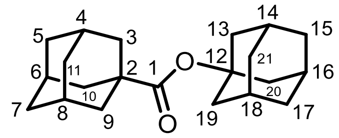

Prepared according to **General procedure D**. Purification via FCC (8 : 2 pentane/Et_2O) gave **7af** as a colourless liquid (1.04 g, 3.30 mmol, 66%).

IR 2910, 2851, 1723 (C=O), 1455, 1240.

^{1}**H NMR** ($CDCl_3$, 400 MHz) δ_H: 2.18 – 2.12 (m, 3H), 2.09 – 2.04 (m, 5H), 2.00 – 1.94 (m, 3H), 1.85 – 1.80 (m, 5H), 1.74 – 1.59 (m, 14H).

^{13}**C NMR** ($CDCl_3$, 101 MHz) δ_C: 177.1, 79.4, 41.4, 41.3, 39.0, 36.8, 36.4, 30.9, 28.2.
HRMS (ES^+) exact mass calculated for $[M+Na]^+$ ($C_{21}H_{30}O_2Na$) requires **m/z** 337.2138, found **m/z** 337.2136.
Melting Point 230-228 °C.

6. Synthesis of Ethers

1-fluoro-4-(isopropoxymethyl)benzene (8a)

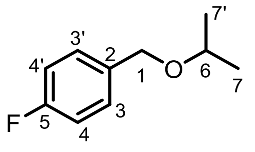

Prepared according to **General procedure E** from **7a**. Purification via FCC (8 : 2 pentane/CH_2Cl_2) gave **8a** as a colourless liquid (31.9 mg, 0.19 mmol, 95%). The NMR spectra and physical properties are consistent with the literature.[17]

^{1}H NMR ($CDCl_3$, 400 MHz) δ_H: 7.31 (m, 2H, C^3H, $C^{3'}H$), 7.02 (m, 2H, C^4H, $C^{4'}H$), 4.47 (s, 2H, C^1H), 3.68 (hept, *J* = 6.1 Hz, 1H, C^6H), 1.22 (d, *J* = 6.1 Hz, 7H, C^7H, $C^{7'}H$).

^{13}C NMR ($CDCl_3$, 101 MHz) δ_C: 162.2 (d, *J* = 244.9 Hz, C^5, $C^{5'}$), 134.8 (d, *J* = 3.1 Hz, C^2), 129.2 (d, *J* = 8.0 Hz, C^3, $C^{3'}$), 115.2 (d, *J* = 21.4 Hz, C^4, $C^{4'}$), 71.1 C^6 (C^6), 69.4 (C^1), 22.1 (C^7, $C^{7'}$).
^{19}F NMR ($CDCl_3$, 376 MHz) δ_F: -115.5 (tt, *J* = 8.9, 5.5 Hz)

1-(tert-butoxymethyl)-4-fluorobenzene (8b)

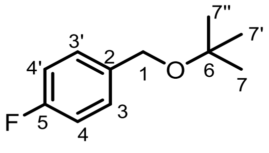

Prepared according to **General procedure E** from **7b**. Purification via FCC (8 : 2 pentane/CH_2Cl_2) gave **8b** as a colourless liquid (29.5 mg, 0.16 mmol, 81%). The NMR spectra and physical properties are consistent with the literature.[17]

^{1}H NMR ($CDCl_3$, 400 MHz) δ_H: δ 7.35 – 7.28 (m, 2H, C^3H, $C^{3'}H$), 7.01 (t, *J* = 8.7 Hz, 2H, C^4H, $C^{4'}H$), 4.41 (d, *J* = 0.8 Hz, 2H, C^1H), 1.29 (s, 9H, C^7H, $C^{7'}H$, $C^{7''}H$).

^{13}C NMR ($CDCl_3$, 101 MHz) δ_C: 162.2 (d, *J* = 244.5 Hz, C^5, $C^{5'}$), 135.7 (d, *J* = 3.2 Hz, , C^2), 129.2 (d, *J* = 8.0 Hz, C^3, $C^{3'}$), 115.2 (d, *J* = 21.4 Hz, C^4, $C^{4'}$), 73.6 (C^6), 63.6 (C^1), 27.8 (C^7, $C^{7'}$, $C^{7''}$).
^{19}F NMR ($CDCl_3$, 376 MHz) δ_F: -115.9 (tt, *J* = 9.1, 5.5 Hz)

1-fluoro-4-(phenoxymethyl)benzene (8c)

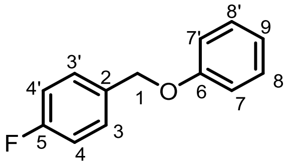

Prepared according to **General procedure E** from **7c**. Purification via FCC (8 : 2 pentane/CH_2Cl_2) gave **8c** as a colourless liquid (26.3 mg, 0.13 mmol, 65%). The NMR spectra and physical properties are consistent with the literature.[18]

^{1}H NMR ($CDCl_3$, 400 MHz) δ_H: δ 7.46 – 7.36 (m, 2H, C^{3}H, C$^{3'}$H), 7.35 – 7.25 (m, 2H, C^{7}H, C$^{7'}$H), 7.12 – 7.03 (m, 2H, C^{4}H, C$^{4'}$H), 7.01 – 6.89 (m, 3H, C^{8}H, C$^{8'}$H, C^{9}H), 5.03 (s, 2H, C^{1}H).

^{13}C NMR ($CDCl_3$, 101 MHz) δ_C: 162.7 (d, J = 246.3 Hz, C^5), 158.8 (C^6), 133.0 (d, J = 3.2 Hz, C^2), 129.7 (C, C^1), 129.5 (d, J = 8.2 Hz, C^3, C$^{3'}$), 121.2 (C^9), 115.6 (d, J = 21.7 Hz, (C^4, C$^{4'}$), 115.0 (C^8, C$^{8'}$), 69.4 (C^1).

^{19}F NMR ($CDCl_3$, 376 MHz) δ_F: -114.3 (tt, J = 9.8, 5.9 Hz).

1-fluoro-4-((4-methoxyphenoxy)methyl)benzene (8d)

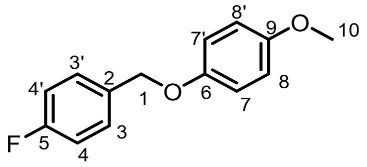

Prepared according to **General procedure E** from **7d**. Purification via FCC (8 : 2 pentane/CH_2Cl_2) gave **8d** as a colourless liquid (24.1 mg, 0.104 mmol, 52%).

IR 2980, 2836, 1508, 1294, 1128.

^{1}H NMR ($CDCl_3$, 400 MHz) δ_H: 7.43 – 7.36 (m, 2H, C^{3}H, C$^{3'}$H), 7.09 – 7.03 (m, 2H, C^{4}H, C$^{4'}$H), 6.92 – 6.87 (m, 2H, C^{8}H, C$^{8'}$H), 6.86 – 6.81 (m, 2H, C^{7}H, C$^{7'}$H), 4.97 (s, 2H, C^{1}H), 3.77 (s, 3H, C^{10}H).

^{13}C NMR ($CDCl_3$, 101 MHz) δ_C: 162.6 (d, J = 246.2 Hz, C$^{5'}$), 154.3 (C^{10}), 152.9 (C^6), 133.2 (d, J = 3.2 Hz, C^2), 129.5 (d, J = 8.2 Hz, C^3, C$^{3'}$), 116.1 (C^8, C$^{8'}$), 115.6 (d, J = 21.3 Hz, C^4, C$^{4'}$), 114.8 (C^7, C$^{7'}$), 70.3 (C^1), 55.9 (C^{10}).

^{19}F NMR ($CDCl_3$, 376 MHz) δ_F: -114.4 (tt, J = 9.2, 5.3 Hz)

HRMS (ES$^+$) exact mass calculated for $[M+H]^+$ ($C_{14}H_{14}FO$) requires **m/z** 233.0972, found **m/z** 233.0973.

(*3S,8R,9S,10R,13S,14S,17S*)-3-((4-fluorobenzyl)oxy)-10,13,17-trimethyl-2,3,4,7,8,9,10,11,12,13,14,15,16,17-tetradecahydro-1H-cyclopenta[a]phenanthren-17-ol (8e)

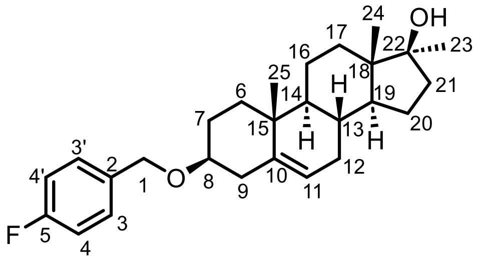

Prepared according to **General procedure E** from **7e**. Purification via FCC (8 : 2 pentane/CH_2Cl_2) gave **8e** as a white solid (57.7 mg, 0.14 mmol, 70%).

IR 3352, 2936, 2855, 1510, 1377, 1225, 1105.

^{1}H NMR ($CDCl_3$, 400 MHz) δ_H: 7.31 (dd, *J* = 8.4, 5.6 Hz, 2H, C^3H, $C^{3'}$H), 7.06 – 6.98 (m, 2H, C^4H, $C^{4'}$H), 5.35 (dt, *J* = 5.1, 1.9 Hz, 1H C^{11}H), 4.52 (s, 2H, C^1H), 3.26 (tt, *J* = 11.3, 4.5 Hz, 1H, C^8H), 2.41 (ddd, *J* = 13.2, 4.8, 2.4 Hz, 1H, C^9), 2.28 (ddd, *J* = 16.0, 11.5, 2.7 Hz, 1H, C^9H), 2.06 – 1.98 (m, 1H, C^{21}H), 1.98 – 1.92 (m, 1H, C^{12}H), 1.88 (dt, *J* = 13.3, 3.5 Hz, 1H, C^6H), 1.83 (ddd, *J* = 15.1, 11.9, 3.6 Hz, 1H, C^{16}H), 1.74 (ddd, *J* = 13.9, 9.6, 6.4 Hz, 1H, $C^{16'}$H), 1.65 – 1.42 (m, 7H, C^7H, $C^{12'}$H, C^{13}H, C^{17}H, C^{20}H, $C^{20'}$H, C^{21}H), 1.35 – 1.23 (m, 3H, $C^{7'}$H, $C^{17'}$H, OH), 1.24 – 1.12 (m, 4H, C^{14}H, C^{23}H), 1.04 (s, 4H, C^6H, C^{25}H), 0.96 – 0.89 (m, 1H, C^{19}H), 0.87 (s, 3H, C^{24}H).

^{13}C NMR ($CDCl_3$, 101 MHz) δ_C: 162.4 (d, *J* = 245.2 Hz, C^5), 141.1 (C^{10}), 134.9 (d, *J* = 3.2 Hz, C^2), 129.4 (d, *J* = 8.2 Hz, C^3, $C^{3'}$), 121.5 (C^{11}), 115.3 (d, *J* = 21.3 Hz, C^4, $C^{4'}$), 81.9 (C^{22}), 78.8 (C^8), 69.4 (C^1), 51.2 (C^{14}), 50.4 (C^{19}), 45.4 (C^{18}), 39.3 (C^9), 39.2 (C^{16}), 37.4 (C^6), 37.2 (C^{15}), 33.0 (C^{13}), 31.9 (C^{21}), 31.7 (C^7), 28.6 (C^{12}), 25.9 (C^{23}), 23.5 (C^{17}), 20.9 (C^{20}), 19.5 (C^{25}), 14.0 (C^{24}).

^{19}F NMR{^{1}H} ($CDCl_3$, 376 MHz) δ_F: -106.1.

HRMS (ES^+) exact mass calculated for $[M+H]^+$ ($C_{27}H_{38}FO_2$) requires **m/z** 413.2850, found **m/z** 413.2849.

Melting Point 190-188 °C.

(((4-fluorobenzyl)oxy)(phenyl)methyl)trimethylsilane (8f)

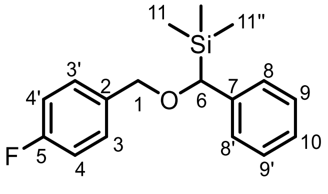

Prepared according to **General procedure E** from **7f**. Purification via FCC (8 : 2 pentane/CH_2Cl_2) gave **8f** as a colourless liquid (47.2 mg, 0.164 mmol, 82%).

IR 3025, 2959, 2898, 1510, 1450, 1247,1223, 1097.

^{1}H NMR ($CDCl_3$, 400 MHz) δ_H: 7.31 (t, *J* = 7.7 Hz, 2H, C^9H, $C^{9'}H$), 7.28 – 7.23 (m, 2H, C^3H, $C^{3'}H$), 7.20 – 7.15 (m, 3H, C^8H, $C^{8'}H$, $C^{10}H$), 7.05 – 6.98 (m, 2H, C^4H, $C^{4'}H$), 4.59 (d, *J* = 12.0 Hz, 1H, C^1H), 4.20 (d, *J* = 11.9 Hz, 1H, $C^{1'}H$), 4.10 (s, 1H, C^6H), -0.03 (s, 9H, $C^{11}H$, $C^{11'}H$, $C^{11''}H$).

^{13}C NMR ($CDCl_3$, 101 MHz) δ_C: 162.3 (d, *J* = 245.1 Hz, C^5), 141.3 (C^7), 134.9 (d, *J* = 3.2 Hz, (C^2), 129.5 (d, *J* = 7.8 Hz, C^3, $C^{3'}$), 128.3 (C^9, $C^{9'}$), 126.1 (C^8, $C^{8'}$), 126.0 (C^{10}), 115.2 (d, *J* = 21.3 Hz, C^4, $C^{4'}$), 77.4 (C^7), 71.5 (C^2), -3.8 (C^{11}, $C^{11'}$, $C^{11''}$).
^{19}F NMR ($CDCl_3$, 376 MHz) δ_F: -115.4 (tt, *J* = 9.4, 5.4 Hz).
HRMS (APCI) exact mass calculated for $[M]^+$ ($C_{17}H_{21}FO$) requires **m/z** 288.1340, found **m/z** 288.1344.

1-fluoro-4-((((*1S,2R,5S*)-2-isopropyl-5-methylcyclohexyl)oxy)methyl)benzene (8g)

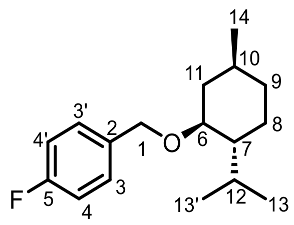

Prepared according to **General procedure E** from **7g**. Purification via FCC (8 : 2 pentane/CH_2Cl_2) gave **8g** as a colourless liquid (44.4 mg, 0.168 mmol, 84%).

IR 2956, 2924, 2870, 1510, 1224, 1108.

^{1}H NMR ($CDCl_3$, 400 MHz) δ_H: 7.34 – 7.28 (m, 2H, C^3H, $C^{3'}H$), 7.05 – 6.98 (m, 2H, C^4H, $C^{4'}H$), 4.62 (d, *J* = 11.3 Hz, 1H, C^1H), 4.36 (d, *J* = 11.3 Hz, 1H, $C^{1'}H$), 3.16 (td, *J* = 10.5, 4.1 Hz, 1H, C^6H), 2.27 (heptd, *J* = 7.0, 2.7 Hz, 1H, $C^{12}H$), 2.21 – 2.14 (m, 1H, $C^{11}H$), 1.70 – 1.59 (m, 2H, C^8H, C^9H), 1.41 – 1.31 (m, 1H, $C^{10}H$), 1.29 (ddt, *J* = 13.2, 10.4, 3.2 Hz, 1H, C^7H), 1.03 – 0.79 (m, 9H, $C^{8'}H$, $C^{9'}H$, $C^{11}H$, $C^{13}H$, $C^{13'}H$), 0.71 (d, *J* = 6.9 Hz, 3H, $C^{14}H$).

^{13}C NMR ($CDCl_3$, 101 MHz) δ_C: 162.4 (d, *J* = 245.0 Hz, C^5), 135.1 (d, *J* = 3.2 Hz, C^2), 129.7 (d, *J* = 8.2 Hz, C^3, $C^{3'}$), 115.3 (d, *J* = 21.5 Hz, C^4, $C^{4'}$), 78.9 (C^6), 69.9 (C^1), 48.5 (C^7), 40.5 (C^{11}), 34.7 (C^9), 31.7 (C^{10}), 25.7 (C^{12}), 23.4 (C^8), 22.5 (C^{13}), 21.2 ($C^{13'}$), 16.2 (C^{14}).
^{19}F NMR ($CDCl_3$, 376 MHz) δ_F: -115.4 (tt, *J* = 9.1, 5.4 Hz).

HRMS (EI) exact mass calculated for $[M]^+$ ($C_{17}H_{25}FO$) requires **m/z** 264.1884, found **m/z** 264.1889.

1-fluoro-4-(((2-(phenylethynyl)cyclohexyl)oxy)methyl)benzene (8h)

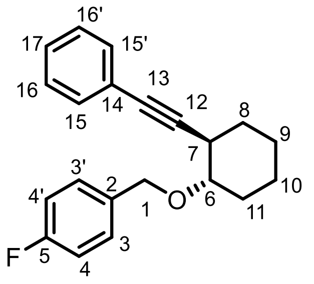

Prepared according to **General procedure E** from **7h**. Purification via FCC (8 : 2 pentane/CH_2Cl_2) gave **8h** as a colourless liquid (39.4 mg, 0.128 mmol, 64%).

IR 2937, 2859, 1510, 1448, 1224, 1150

^{1}H NMR ($CDCl_3$, 400 MHz) δ_H: 7.36 – 7.28 (m, 4H, C^3H, $C^{3'}$H, C^{15}H, $C^{15'}$H), 7.24 – 7.20 (m, 3H, C^{16}H, $C^{16'}$H, C^{17}H), 6.96 – 6.86 (m, 2H, C^4H, $C^{4'}$H), 4.60 (s, 2H, C^1H), 3.37 (td, *J* = 8.5, 3.7 Hz, 1H, C^6H), 2.62 (ddd, *J* = 9.7, 8.2, 4.0 Hz, 1H, C^7H), 2.06 – 1.94 (m, 2H, C^8H), 1.74 – 1.57 (m, 2H, C^{11}H), 1.53 – 1.40 (m, 1H, C^9H), 1.38 – 1.10 (m, 3H, $C^{9'}$H, C^{10}H, $C^{10'}$H).

^{13}C NMR ($CDCl_3$, 101 MHz) δ_C: 162.2 (d, *J* = 244.9 Hz, C^5), 134.7 (d, *J* = 3.2 Hz, C^2), 131.6 (C^{15}, $C^{15'}$), 129.4 (d, *J* = 8.1 Hz, C^2), 128.2 (C^{16}, $C^{16'}$), 127.6 (C^{17}), 124.0 (C^{14}), 115.1 (d, *J* = 21.3 Hz, C^4), 92.4 (C^{12}), 81.7 (C^{13}), 79.7 (C^6), 70.7 (C^1), 36.1 (C^{17}), 30.7 (C^7), 30.5 (C^8), 29.7 (C^{11}), 24.2 (C^9), 23.5 (C^{10}).

^{19}F NMR ($CDCl_3$, 376 MHz) δ_F: -115.36 (t, *J* = 6.3 Hz)

HRMS (ES^+) exact mass calculated for $[M+Na]^+$ ($C_{21}H_{21}FONa$) requires **m/z** 331.1469, found **m/z** 331.1472.

4,4'-((butane-2,3-diylbis(oxy))bis(methylene))bis(fluorobenzene) (8i)

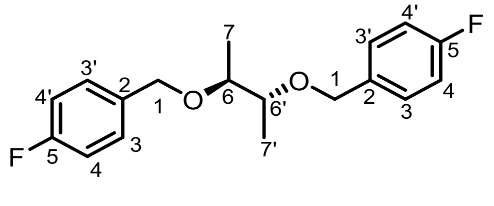

Prepared according to **General procedure E** from **7i** using 2.0 mol% of IrCl(CO)[P(HFIP)$_3$]$_2$ complex and 8.0 equivalent of TMDS. Purification via FCC (8 : 2 pentane/CH_2Cl_2) gave **8i** as a colourless liquid (53.3 mg, 0.174 mmol, 87%).

IR 2976, 2873, 1510, 1223, 1108.

^{1}H NMR ($CDCl_3$, 400 MHz) δ_H: 7.33 – 7.26 (m, 4H, C^{3}H, C$^{3'}$H), 7.01 (t, *J* = 8.7 Hz, 4H, C^{4}H, C$^{4'}$H), 4.58 (d, *J* = 11.7 Hz, 2H, C^{1}H, C$^{1'}$H), 4.50 (d, *J* = 11.7 Hz, 2H, C^{1}H, C$^{1'}$H), 3.60 – 3.53 (m, 2H, C^{6}H, C$^{6'}$H), 1.16 (d, *J* = 6.2 Hz, 6H, C^{7}H, C$^{7'}$H).

^{13}C NMR ($CDCl_3$, 101 MHz) δ_C: 162.4 (d, *J* = 245.2 Hz, C^5), 134.8 (d, *J* = 3.0 Hz, C^2), 129.4 (d, *J* = 7.9 Hz, C^3, C$^{3'}$), 115.3 (d, *J* = 21.3 Hz, C^4, C$^{4'}$), 77.5 (C^6, C$^{6'}$), 70.8 (C^1, C$^{1'}$), 15.2 (C^7, C$^{7'}$).
^{19}F NMR ($CDCl_3$, 376 MHz) δ_F: -115.2 (tt, *J* = 9.4, 5.4 Hz).
HRMS (ES$^+$) exact mass calculated for $[M+Na]^+$ ($C_{18}H_{20}F_2O_2Na$) requires **m/z** 329.1324, found **m/z** 329.1319.

3-((4-fluorobenzyl)oxy)butan-2-ol (8j)

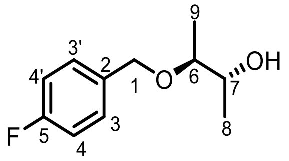

Prepared according to **General procedure E** from **7j**. using 5.0 equivalent of TMDS Purification via FCC (8 : 2 pentane/CH_2Cl_2) gave **8j** as a colourless liquid (28.5 mg, 0.144 mmol, 72%).

IR 3438, 2978, 2879, 1510, 1378, 1223, 1085.

^{1}H NMR ($CDCl_3$, 400 MHz) δ_H: 7.34 – 7.28 (m, 2H, C^{3}H, C$^{3'}$H), 7.07 – 7.00 (m, 2H, C^{4}H, C$^{4'}$H), 4.63 (d, *J* = 11.4 Hz, 1H, C^{1}H), 4.41 (d, *J* = 11.3 Hz, 1H, C$^{1'}$H), 3.61 (pd, *J* = 6.5, 2.4 Hz, 1H, C^{7}H), 3.34 – 3.26 (m, 1H, C^{6}H), 2.63 (br d, *J* = 2.8 Hz, 1H, OH), 1.18 – 1.15 (m, 6H, C^{8}H, C^{9}H).

^{13}C NMR ($CDCl_3$, 101 MHz) δ_C: 162.4 (d, *J* = 246.0 Hz, C^5), 134.1 (d, *J* = 3.2 Hz, C^2), 129.5 (d, *J* = 8.1 Hz, C^3, C$^{3'}$), 115.4 (d, *J* = 21.3 Hz, C^4, C$^{4'}$), 80.2 (C^6), 71.2 (C^7), 70.4 (C^1), 18.6 (C^9), 15.4 (C^8).
^{19}F NMR (CDCl3, 376 MHz) δ_F: -114.7 (tt, *J* = 8.7, 5.3 Hz).
HRMS (ES$^+$) exact mass calculated for $[M+Na]^+$ ($C_{11}H_{15}FO_2Na$) requires **m/z** 221.0948, found **m/z** 221.0952.

1-fluoro-4-((1-phenylethoxy)methyl)benzene (8k)

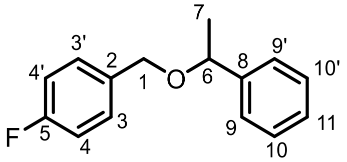

Prepared according to **General procedure E** from **7k**. Purification via FCC (8 : 2 pentane/CH_2Cl_2) gave **8k** as a colourless liquid (39.5 mg, 0.172 mmol, 86%).

IR 3035, 2986, 1506, 1270.

^{1}H NMR ($CDCl_3$, 500 MHz) δ_H: 7.43 – 7.36 (m, 4H, $C^{10}H$, $C^{10'}H$, C^4H, $C^{4'}H$), 7.36 – 7.27 (m, 3H, C^3H, $C^{3'}H$, $C^{11}H$), 7.09 – 7.01 (m, 2H, C^9H, $C^{9'}H$), 4.52 (q, *J* = 6.5 Hz, 1H, C^6H), 4.42 (d, *J* = 11.6 Hz, 1H, C^1H), 4.30 (d, *J* = 11.6 Hz, 1H, $C^{1'}H$), 1.51 (d, *J* = 6.5 Hz, 3H, C^7H).

^{13}C NMR ($CDCl_3$, 126 MHz) δ_C: 162.4 (d, *J* = 245.3 Hz, C^5), 143.7 (C^8), 134.5 (d, *J* = 3.2 Hz, C^2), 129.6 (d, *J* = 8.1 Hz, C^3, $C^{3'}$), 128.7 (C^{10}, $C^{10'}$), 127.7 (C^{11}), 126.5 (C^9, $C^{9'}$), 115.2 (d, *J* = 21.6 Hz, C^4, $C^{4'}$), 77.5 (C^6), 69.8 (C^1), 24.3 (C^7).

^{19}F NMR ($CDCl_3$, 471 MHz) δ_F: -115.2 (tt, *J* = 8.8, 5.2 Hz).

HRMS (ES^+) exact mass calculated for $[M+Na]^+$ ($C_{15}H_{15}FONa$) requires **m/z** 253.0999, found **m/z** 253.0990.

1-fluoro-4-((1-methylcyclobutoxy)methyl)benzene(8l)

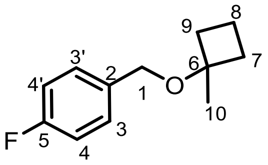

Prepared according to **General procedure E** from **7l**. Purification via FCC (8 : 2 pentane/CH_2Cl_2) gave **8l** as a colourless liquid (31.0 mg, 0.16 mmol, 80%).

IR 2967, 2869, 1511, 1222, 1154.

^{1}H NMR ($CDCl_3$, 400 MHz) δ_H: 7.36 – 7.28 (m, 2H, C^3H, $C^{3'}H$), 7.06 – 6.97 (m, 2H C^3H, $C^{3'}H$), 4.34 (s, 2H, C^1H), 2.25 – 2.16 (m, 2H, C^7H, C^9H), 1.96 – 1.87 (m, 2H, $C^{7'}H$, $C^{9'}H$), 1.82 – 1.71 (m, 1H, C^8H), 1.68 – 1.55 (m, 1H, $C^{8'}H$), 1.42 (s, 3H, $C^{10}H$).

^{13}C NMR ($CDCl_3$, 101 MHz) δ_C: 162.3 (d, *J* = 244.8 Hz, C^5), 135.2 (d, *J* = 3.1 Hz, C^2), 129.3 (d, *J* = 8.1 Hz, C^3, $C^{3'}$), 115.3 (d, *J* = 21.3 Hz, C^4, $C^{4'}$), 77.5 (C^6), 64.0 (C^1), 34.1 (C^7, C^9), 23.4 (C^{10}), 12.5 (C^8).

^{19}F NMR ($CDCl_3$, 376 MHz) δ_F: -115.67 (tt, J = 9.3, 5.4 Hz).
HRMS (ES^+) exact mass calculated for $[M+H]^+$ ($C_{12}H_{15}FONa$) requires **m/z** 217.0999, found **m/z** 217.0996.

1-((4-fluorobenzyl)oxy)-1-methylcyclooctane (8m)

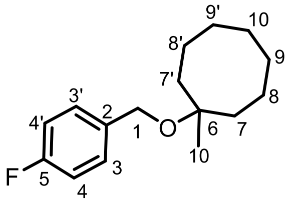

Prepared according to **General procedure E** from **7m**. Purification via FCC (8 : 2 pentane/CH_2Cl_2) gave **8m** as a colourless liquid (35.4 mg, 0.15 mmol, 75%).

IR 2922, 2856, 1510, 1222, 1123.

^{1}H NMR ($CDCl_3$, 400 MHz) δ_H: 7.35 – 7.27 (m, 2H, C^3H, $C^{3'}$H), 7.04 – 6.96 (m, 2H, C^4H, $C^{4'}$H), 4.37 (s, 2H, C^1H), 1.92 (ddd, J = 15.0, 9.6, 2.1 Hz, 2H, C^7H, $C^{7'}$H), 1.78 – 1.66 (m, 2H, C^7H, $C^{7'}$H), 1.65 – 1.39 (m, 10H, C^8H, $C^{8'}$H, C^9H, $C^{9'}$H, C^{10}H), 1.21 (s, 3H, C^{10}H).

^{13}C NMR ($CDCl_3$, 101 MHz) δ_C: 162.0 (d, J = 244.3 Hz, C^6), 135.8 (d, J = 3.2 Hz, C^2), 128.9 (d, J = 8.1 Hz, C^3, $C^{3'}$), 115.0 (d, J = 21.3 Hz, C^4, $C^{4'}$), 77.9 (C^6), 62.4 (C^1), 34.7 (C^7, $C^{7'}$), 28.4 (C^8, $C^{8'}$), 25.2 (C^{11}), 24.8 (C^{10}), 22.1 (C^9, $C^{9'}$).
^{19}F NMR ($CDCl_3$, 376 MHz) δ_F: -116.12 (tt, J = 8.5, 5.5 Hz).
HRMS (ES^+) exact mass calculated for $[M+H]^+$ ($C_{16}H_{23}FONa$) requires **m/z** 273.1625, found **m/z** 273.1628.

1-((4-fluorobenzyl)oxy)adamantane (8n)

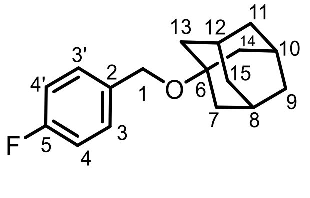

Prepared according to **General procedure E** from **7n**. Purification via FCC (8 : 2 pentane/CH_2Cl_2) gave **8n** as a colourless liquid (44.7 mg, 0.172 mmol, 86%).

IR 2908, 2853, 1510, 1222, 1116.

^{1}H NMR ($CDCl_3$, 400 MHz) δ_H: 7.34 – 7.28 (m, 2H, C^3H, $C^{3'}H$), 7.03 – 6.97 (m, 2H, C^4H, $C^{4'}H$), 4.47 (s, 2H, C^1H), 2.22 – 2.14 (m, 3H, C^8H, $C^{10}H$, $C^{12}H$), 1.84 (br d, *J* = 2.9 Hz, 6H, C^7H, $C^{13}H$, $C^{15}H$), 1.70 – 1.60 (m, 6H, C^9H, $C^{11}H$, $C^{14}H$).

^{13}C NMR ($CDCl_3$, 101 MHz) δ_C: 162.2 (d, *J* = 244.3 Hz, C^5), 136.0 (d, *J* = 3.2 Hz, C^2), 129.3 (d, *J* = 8.1 Hz, C^3, $C^{3'}$), 115.2 (d, *J* = 21.3 Hz, C^4, $C^{4'}$), 73.0 (C^6), 61.8 (C^1), 41.9 (C^7, C^{13}, C^{15}), 36.6 (C^9, C^{11}, C^{14}), 30.7 (C^8, C^{10}, C^{12}).

^{19}F NMR ($CDCl_3$, 376 MHz) δ_F: -116.0 (tt, *J* = 8.9, 5.5 Hz)

HRMS (ES^+) exact mass calculated for $[M+H]^+$ ($C_{17}H_{22}FO$) requires **m/z** 261.1649, found **m/z** 261.1650.

(*3R,3aS,6R,7R,8aS*)-6-((4-fluorobenzyl)oxy)-3,6,8,8-tetramethyloctahydro-1H-3a,7-methanoazulene (8o)

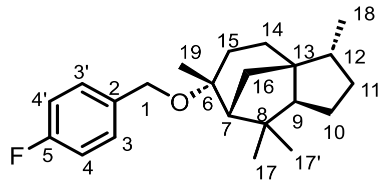

Prepared according to **General procedure E** from **7o**. Purification via FCC (8 : 2 pentane/CH_2Cl_2) gave **8o** as a colourless liquid (46.8 mg, 0.142 mmol, 71%).

IR 2973, 2872, 1510, 1463, 1414, 1224, 1128.

^{1}H NMR ($CDCl_3$, 400 MHz) δ_H: 7.34 – 7.28 (m, 2H, C^3H, $C^{3'}H$), 7.02 – 6.97 (m, 2H, C^4H, $C^{4'}H$), 4.49 – 4.34 (d, *J* = 4.0 Hz, 2H, C^1H), 2.00 – 1.93 (m, 1H, $C^{11}H$), 1.91 (dd, *J* = 5.2, 1.6 Hz, 1H, C^7H), 1.90 – 1.85 (m, 1H, $C^{15}H$), 1.83 – 1.79 (m, 1H, C^9H), 1.78 – 1.72 (m, 1H, $C^{11}H$), 1.71 – 1.61 (m, 2H, $C^{12}H$, $C^{16}H$), 1.54 – 1.50 (m, 1H, $C^{10}H$), 1.50 – 1.44 (m, 1H, $C^{14}H$), 1.44 – 1.37 (m, 2H, $C^{10}H$, $C^{14}H$), 1.37 – 1.23 (m, 8H, $C^{15'}H$, $C^{16'}H$, $C^{17}H$, $C^{17'}H$), 1.00 (s, 3H, $C^{19}H$), 0.86 (d, *J* = 7.1 Hz, 3H, $C^{18}H$).

^{13}C NMR ($CDCl_3$, 101 MHz) δ_C: 162.1 (d, *J* = 244.2 Hz, C^5), 135.7 (d, *J* = 3.0 Hz, C^2), 129.2 (d, *J* = 7.9 Hz, C^3, $C^{3'}$), 115.1 (d, *J* = 21.3 Hz, C^4, $C^{4'}$), 79.3 (C^6), 62.3 (C^1), 57.1 (C^9), 56.6 (C^7), 54.1 (C^{13}), 43.5 (C^8), 41.6 (C^{12}), 41.5 (C^{16}), 37.2 (C^{15}), 33.3 (C^{11}), 31.5 (C^{14}), 29.0 (C^{19}), 27.4 (C^{17}), 25.5 (C^{10}), 25.2 ($C^{17'}$), 15.7 (C^{18}).

^{19}F NMR ($CDCl_3$, 376 MHz) δ_F: -116.2 (tt, *J* = 8.5, 5.3 Hz)

HRMS (ES^+) exact mass calculated for $[M+H]^+$ ($C_{22}H_{31}FO$) requires **m/z** 330.2354, found **m/z** 330.2359.

5-(isopropoxymethyl)benzo[d][1,3]dioxole (8p)

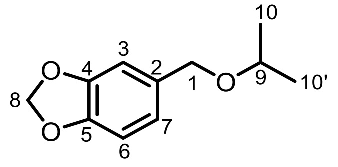

Prepared according to **General procedure E** from **7p**. Purification via FCC (8 : 2 pentane/CH_2Cl_2) gave **8p** as a colourless liquid (22.9 mg, 0.118 mmol, 59%).

IR 2973, 2877, 1731, 1504, 1253, 1127.

^{1}H NMR ($CDCl_3$, 400 MHz) δ_H: 6.86 (br d, *J* = 1.5 Hz, 1H, C^6H), 6.82 – 6.73 (m, 2H, C^3H, C^7H), 5.94 (s, 2H, C^8H), 4.40 (s, 2H, C^1H), 3.66 (hept, *J* = 6.1 Hz, 1H, C^9H), 1.20 (d, *J* = 6.1 Hz, 6H, $C^{10}H$, $C^{10'}H$).

^{13}C NMR ($CDCl_3$, 101 MHz) δ_C: 147.8 (C^4), 147.0 (C^5), 133.1 (C^2), 121.1 (C^7), 108.5 (C^3), 108.1 (C^6), 101.0 (C^8), 70.8 (C^9), 70.0 (C^1), 22.3 (C^{10}, $C^{10'}$).

HRMS (ES^+) exact mass calculated for $[M+Na]^+$ ($C_{11}H_{14}O_3Na$) requires **m/z** 217.0835, found **m/z** 217.0829.

1-(isopropoxymethyl)-4-methoxybenzene (8q)

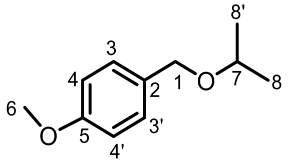

Prepared according to **General procedure E** from **7q**. Purification via FCC (8 : 2 pentane/CH_2Cl_2) gave **8q** as a colourless liquid (19.8 mg, 0.11 mmol, 55%). The NMR spectra and physical properties are consistent with the literature.[19]

^{1}H NMR ($CDCl_3$, 400 MHz) δ_H: 7.26 (m, 2H, C^3H, $C^{3'}H$), 6.90 – 6.84 (m, 2H, C^4H, $C^{4'}H$), 4.44 (s, 2H, C^1H), 3.80 (s, 3H, C^6H), 3.67 (hept, *J* = 6.1 Hz, 1H, C^7H), 1.20 (d, *J* = 6.1, Hz, 6H, C^8H, $C^{8'}H$).

^{13}C NMR ($CDCl_3$, 101 MHz) δ_C: 159.2 (C^5), 131.4 (C^2), 129.2 (C^3, $C^{3'}$), 113.9 (C^4, $C^{4'}$), 70.7 (C^7), 69.8 (C^1), 55.4 (C^6), 22.3 (C^8, $C^{8'}$).

1-(isopropoxymethyl)-3-methoxybenzene (8r)

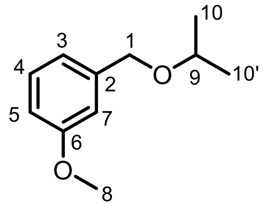

Prepared according to **General procedure E** from **7r**. Purification via FCC (8 : 2 pentane/CH_2Cl_2) gave **8r** as a colourless liquid (28.4 mg, 0.16 mmol, 79%).

^{1}H NMR (400 MHz, $CDCl_3$) δ 7.29 – 7.20 (m, 1H, C^4H), 6.93 (m, 2H, C^3H, C^7H), 6.85 – 6.78 (m, 1H, C^5H), 4.53 – 4.48 (m, 2H, C^1H), 3.82 (s, 3H, C^8H), 3.69 (hept *J* = 6.1 Hz, 1H, C^9H), 1.23 (d, *J* = 6.1 Hz, 6H, C^{10}H, $C^{10'}$H).

^{13}C NMR (101 MHz, $CDCl_3$) δ 159.8 (C^6), 140.9 (C^2), 129.4 (C^4), 119.9 (C^3), 113.1 (C^7), 113.0 (C^5), 71.0 (C^9), 70.0 (C^1), 55.3 (C^8), 22.2 (C^{10}, $C^{10'}$).

HRMS (ES^+) exact mass calculated for $[M+Na]^+$ ($C_{11}H_{16}O_2Na$) requires **m/z** 203.1043, found **m/z** 203.1034.

1-(isopropoxymethyl)-2-methoxybenzene (8s)

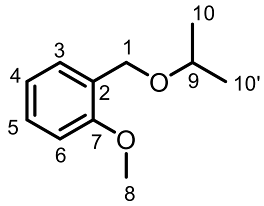

Prepared according to **General procedure E** from **7s**. Purification via FCC (8 : 2 pentane/CH_2Cl_2) gave **8s** as a colourless liquid (18.5 mg, 0.10 mmol, 51%).

^{1}H NMR (400 MHz, $CDCl_3$) δ 7.42 (ddt, *J* = 7.4, 1.8, 0.9 Hz, 1H, C^3H), 7.29 – 7.19 (m, 1H, C^4H), 6.95 (td, *J* = 7.4, 1.1 Hz, 1H, C^6H), 6.85 (dd, *J* = 8.2, 1.1 Hz, 1H, C^5H), 4.56 (s, 2H, C^1H), 3.83 (s, 3H, C^8H), 3.77 – 3.64 (hept, *J* = 6.2 Hz, 1H, C^9H), 1.23 (d, *J* = 6.1 Hz, 6H, C^{10}H, $C^{10'}$H).
^{13}C NMR (101 MHz, $CDCl_3$) δ 157.0 (C^7), 128.7 (C^5), 128.3 (C^6), 127.8 (C^2), 120.6 (C^4), 110.2 (C^3), 71.3 (C^9), 64.9 (C^1), 55.4 (C^8), 22.3 (C^{10}, $C^{10'}$).
HRMS (ES^+) exact mass calculated for $[M+Na]^+$ ($C_{11}H_{16}O_2Na$) requires **m/z** 203.1043, found **m/z** 203.1034.

1-(isopropoxymethyl)-2-methylbenzene (8t)

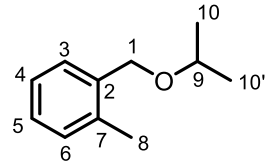

Prepared according to **General procedure E** from **7t**. Purification via FCC (8 : 2 pentane/CH_2Cl_2) gave **8t** as a colourless liquid (24.1 mg, 0.15 mmol, 73%).

^{1}H NMR (400 MHz, $CDCl_3$) δ 7.34 (dd, *J* = 6.9, 2.1 Hz, 1H, C^6H), 7.24 – 7.12 (m, 3H, C^3H, C^4H, C^5H), 4.50 (s, 2H, C^1H), 3.70 (hept, *J* = 6.1 Hz, 1H, C^9H), 2.35 (s, 3H, C^8H), 1.23 (d, *J* = 6.1 Hz, 6H, C^{10}H, $C^{10'}$H).

^{13}C NMR (101 MHz, $CDCl_3$) δ 137.0 (C^2), 136.8 (C^7), 130.3 (C^3), 128.6 (C^5), 127.7 (C^6), 125.9 (C^4), 71.3 (C^9), 68.7 (C^1), 22.3 (C^{10}, $C^{10'}$), 18.9 (C^8).

HRMS (ES^+) exact mass calculated for $[M+Na]^+$ ($C_{11}H_{16}ONa$) requires **m/z** 187.1093, found **m/z** 187.1087.

(3-isopropoxypropyl)benzene (8u)

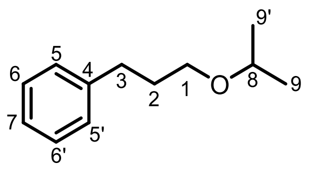

Prepared according to **General procedure E** from **7u**. Purification via FCC (8 : 2 pentane/CH_2Cl_2) gave **8u** as a colourless liquid (31.3 mg, 0.176 mmol, 88%). The NMR spectra and physical properties are consistent with the literature.[20]

^{1}H NMR ($CDCl_3$, 400 MHz) δ_H: 7.31 – 7.25 (m, 2H, C^6H, $C^{6'}$H), 7.23 – 7.16 (m, 3H, C^5H, $C^{5'}$H, C^7H), 3.55 (hept, *J* = 6.1 Hz, 1H, C^8H), 3.42 (t, *J* = 6.4 Hz, 2H, C^1H, $C^{1'}$H), 2.70 (t, *J* = 6.8 Hz, 2H, C^3H), 1.93 – 1.84 (m, 2H, C^2H), 1.16 (d, *J* = 6.1 Hz, 6H, C^9H, $C^{9'}$H).

^{13}C NMR ($CDCl_3$, 101 MHz) δ_C: 142.3 (C^4), 128.6 (C^5, $C^{5'}$), 128.4 (C^6, $C^{6'}$), 125.8 (C^7), 71.5 (C^8), 67.4 (C^1), 32.5 (C^3), 31.8 (C^2), 22.3 (C^9, $C^{9'}$).

1-(4-isopropoxybutyl)-4-methoxybenzene (8v)

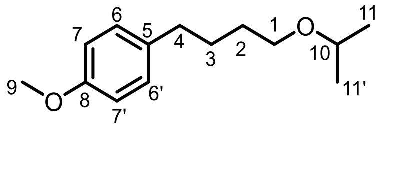

Prepared according to **General procedure E** from **7v**. Purification via FCC (8 : 2 pentane/CH_2Cl_2) gave **8v** as a colourless liquid (36.8 mg, 0.166 mmol, 83%).

IR 2952, 2910, 2862, 1510, 1240, 1113.

^{1}H NMR ($CDCl_3$, 400 MHz) δ_H: 7.13 – 7.07 (m, 2H, C^7H, $C^{7'}H$), 6.85 – 6.79 (m, 2H, C^6H, $C^{6'}H$), 3.79 (s, 3H, C^9H), 3.53 (hept, *J* = 6.1 Hz, 1H, $C^{10}H$), 3.41 (t, *J* = 6.4 Hz, 2H, C^1H, $C^{1'}H$), 2.57 (t, *J* = 7.4 Hz, 2H, C^4H), 1.70 – 1.54 (m, 4H, C^2H, C^3H), 1.14 (d, *J* = 6.1 Hz, 6H, $C^{11}H$, $C^{11'}H$).

^{13}C NMR ($CDCl_3$, 101 MHz) δ_C: 157.8 (C^8), 134.8 (C^5), 129.4 (C^6, $C^{6'}$), 113.8 (C^7, $C^{7'}$), 71.4 (C^{10}), 68.1 (C^1), 55.4 (C^9), 35.0 (C^4), 29.9 (C^3), 28.6 (C^2), 22.3 (C^{11}, $C^{11'}$).

HRMS (ES^+) exact mass calculated for $[M+H]^+$ ($C_{14}H_{23}O_2$) requires **m/z** 223.1693, found **m/z** 223.1696.

3-(2-isopropoxyethyl)-1H-indole (8w)

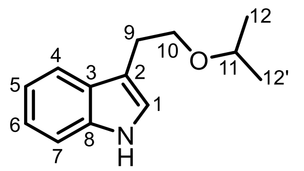

Prepared according to **General procedure E** from **7w** using 5.0 equivalent of TMDS. Purification via FCC (8 : 2 pentane/CH_2Cl_2) gave **8w** as a yellow liquid (29.2 mg, 0.144 mmol, 72%). The NMR spectra and physical properties are consistent with the literature.[21]

^{1}H NMR ($CDCl_3$, 400 MHz) δ_H: 7.99 (brs, 1H, NH), 7.66 (d, *J* = 7.7 Hz, 1H, C^4H), 7.38 (d, *J* = 8.1 Hz, 1H, C^7H), 7.21 (ddd, *J* = 8.2, 7.0, 1.2 Hz, 1H, C^5H), 7.14 (ddd, *J* = 8.0, 7.0, 1.0 Hz, 1H, C^6H), 7.11 – 7.06 (m, 1H, C^1H), 3.74 (t, *J* = 7.5 Hz, 2H, $C^{10}H$), 3.66 (hept, *J* = 6.1 Hz, 1H, $C^{11}H$), 3.06 (t, *J* = 7.5 Hz, 2H, C^9H), 1.21 (d, *J* = 6.1 Hz, 6H, $C^{12}H$, $C^{12'}H$).

^{13}C NMR ($CDCl_3$, 101 MHz) δ_C: 136.2 (C^8), 127.7 (C^3), 121.9 (C^1), 121.9 (C^5), 119.2 (C^6), 118.9 (C^4), 113.4 (C^2), 111.0 (C^7), 71.5 (C^{11}), 68.5 (C^{10}), 26.3 (C^9), 22.2 (C^{12}, $C^{12'}$).

(isopropoxymethyl)cyclohexane (8x)

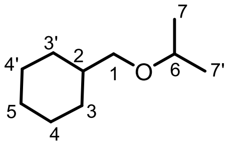

Prepared according to **General procedure E** from **7x**. Purification via FCC (8 : 2 pentane/CH_2Cl_2) gave **8x** as a colourless liquid (19.9 mg, 0.128 mmol, 64%). %). The NMR spectra and physical properties are consistent with the literature.[22]

^{1}H NMR ($CDCl_3$, 400 MHz) δ_H: 3.50 (hept, J = 6.1 Hz, 1H, C^6H), 3.19 (d, J = 6.6 Hz, 2H, C^1H), 1.82 – 1.60 (m, 3H, C^3H, C^5H), 1.60 – 1.44 (m, 1H, C^2H 2), 1.37 – 1.15 (m, 4H, C^4H, $C^{4'}H$), 1.13 (d, J = 6.1 Hz, 6H, C^7H, $C^{7'}H$), 0.97 – 0.81 (m, 3H, C^3H).

^{13}C NMR ($CDCl_3$, 101 MHz) δ_C: 74.4 (C^1), 71.6 (C^6), 38.5 (C^2), 30.4 (C^3, $C^{3'}$), 26.9 (C^5), 26.1 (C^4, $C^{4'}$).

1-(isopropoxymethyl)adamantane (8y)

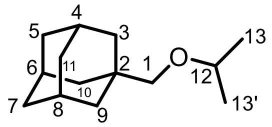

Prepared according to **General procedure F** from **7y**. Purification via FCC (8 : 2 pentane/CH_2Cl_2) gave **8y** as a colourless liquid (32.9 mg, 0.158 mmol, 79%).

IR 3005, 2950, 1731 (C=O), 1599, 1486, 1435.

^{1}H NMR ($CDCl_3$, 400 MHz) δ_H: 3.38 (hept, J = 6.1 Hz, 1H, $C^{12}H$), 2.88 (s, 2H, C^2H), 1.88 (p, J = 3.2 Hz, 3H, C^4H, C^6H, C^8H), 1.68 – 1.52 (m, 6H, C^3H, C^9H, $C^{10}H$), 1.44 (m, 6H, C^5H, C^7H, $C^{11}H$), 1.05 (d, J = 6.1 Hz, 6H, $C^{13}H$, $C^{13'}H$).

^{13}C NMR ($CDCl_3$, 101 MHz) δ_C: 79.6 (C^1), 72.3 (C^{12}), 39.9 (C^3, C^9, C^{10}), 37.5 (C^5, C^7, C^{11}), 28.5 (C^3, C^9, C^{10}), 22.2 (C^{13}, $C^{13'}$).

HRMS (ES^+) exact mass calculated for $[M+H]^+$ ($C_{14}H_{25}O$) requires **m/z** 209.1903, found **m/z** 209.1905.

(1-(isopropoxymethyl)cyclopropyl)benzene (8z)

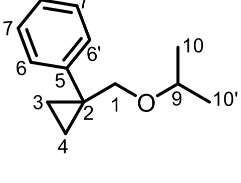

Prepared according to **General procedure F** from **7z**. Purification via FCC (8 : 2 pentane/CH_2Cl_2) gave **8z** as a colourless liquid (27.7 mg, 0.146 mmol, 73%).

IR 2972, 2872, 1446, 1260, 1110.

^{1}H NMR ($CDCl_3$, 400 MHz) δ_H: 7.33 (dd, J = 8.0, 1.5 Hz, 2H, C^7H, $C^{7'}H$), 7.28 (d, J = 7.6 Hz, 2H, C^6H, $C^{6'}H$), 7.17 (t, J = 7.4 Hz, 1H, C^8H), 3.63 – 3.35 (m, 3H, C^1H, C^9H), 1.09 (d, J = 6.1 Hz, 6H, $C^{10}H$, $C^{10'}H$), 0.87 (m, 4H, C^3H, C^4H).

^{13}C NMR ($CDCl_3$, 101 MHz) δ_C: 144.1 (C^5), 128.6 (C^7, $C^{7'}$), 128.1 (C^6, $C^{6'}$), 126.1 (C^8), 75.3 (C^1), 71.7 (C^9), 25.5 (C^2), 22.2 (C^3, C^4), 12.0, (C^{10}, $C^{10'}$).

HRMS (EI) exact mass calculated for $[M]^+$ ($C_{13}H_{18}O$) requires **m/z** 190.1352, found **m/z** 190.1357.

(1-(isopropoxymethyl)cyclobutyl)benzene (8aa)

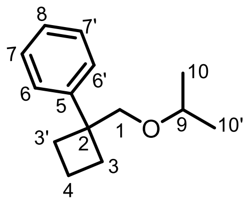

Prepared according to **General procedure F** from **7aa**. Purification via FCC (8 : 2 pentane/CH_2Cl_2) gave **8aa** as a colourless liquid (27.7 mg, 0.136 mmol, 68%).

IR 2973, 2872, 1510, 1224, 1087.

^{1}H NMR ($CDCl_3$, 400 MHz) δ_H: 7.30 (td, J = 7.3, 1.5 Hz, 2H, C^7H, $C^{7'}H$), 7.23 – 7.13 (m, 3H, C^6H, $C^{6'}H$, C^8H), 3.50 (s, 2H, C^1H), 3.37 (hept, J = 6.1 Hz, 1H, C^9H), 2.38 – 2.25 (m, 4H, C^3H, $C^{3'}H$), 2.15 – 1.99 (m, 1H, C^4H), 1.90 – 1.76 (m, 1H, $C^{4'}H$), 1.07 (d, J = 6.1 Hz, 6H, $C^{10}H$, $C^{10'}H$).

^{13}C NMR ($CDCl_3$, 101 MHz) δ_C: 149.1 (C^5), 127.8 (C^7, $^{C7'}$), 126.3 (C^6, $C^{6'}$), 125.5 (C^8), 76.5 (C^1), 72.3 (C^9), 47.1 (C^2), 30.3 (C^3, $C^{3'}$), 22.1 (d, J = 0.9 Hz, C^{10}, $C^{10'}$).

HRMS (ES^+) exact mass calculated for $[M+H]^+$ ($C_{14}H_{21}O$) requires **m/z** 205.1587, found **m/z** 205.1589.

(1-(isopropoxymethyl)cyclopentyl)benzene (8ab)

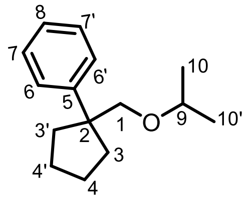

Prepared according to **General procedure F** from **7ab**. Purification via FCC (8 : 2 pentane/CH_2Cl_2) gave **8ab** as a colourless liquid (36.2mg, 0.166 mmol, 83%).

IR 2968, 2873, 1497, 1260, 1127.

^{1}H NMR ($CDCl_3$, 400 MHz) δ_H: 7.40 – 7.33 (m, 2H, C^7H, $C^{7'}H$), 7.29 (m, 2H, C^6H, $C^{6'}H$), 7.22 – 7.15 (m, 1H, C^8H), 3.39 (s, 2H, C^1H), 3.31 (hept, *J* = 6.1 Hz, 1H. C^9H), 2.11 – 1.98 (m, 2H, C^3H, $C^{3'}H$), 1.97 – 1.82 (m, 2H, C^3H, $C^{3'}H$), 1.82 – 1.63 (m, 4H, C^4H, $C^{4'}H$), 1.04 (d, *J* = 6.1 Hz, 6H, $C^{10}H$, $C^{10'}H$).

^{13}C NMR ($CDCl_3$, 101 MHz) δ_C: 148.6 (C^5), 127.8 (C^7, $C^{7'}$), 127.3 (C^6, $C^{6'}$), 125.6 (C^8), 75.8 (C^1), 72.3 (C^9), 52.1 (C^2), 35.3 (C^3, $C^{3'}$), 24.4 (C^4, $C^{4'}$), 22.1 (C^{10}, $C^{10'}$).
HRMS (ES^+) exact mass calculated for $[M+H]^+$ ($C_{15}H_{23}O$) requires **m/z** 219.1743, found **m/z** 219.1744.

(1-(isopropoxymethyl)cyclohexyl)benzene (8ac)

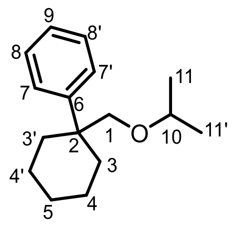

Prepared according to **General procedure F** from **7ac**. Purification via FCC (8 : 2 pentane/CH_2Cl_2) gave **8ac** as a colourless liquid (37.1 mg, 0.16 mmol, 80%).

IR 2971, 2931, 2860, 1455, 1260, 1128.

^{1}H NMR ($CDCl_3$, 400 MHz) δ_H: 7.36 – 7.29 (m, 2H, C^7H, $C^{7'}H$), 7.28 – 7.21 (m, 2H, C^8H, $C^{8'}H$), 7.16 – 7.06 (m, 1H, C^9H), 3.26 – 3.13 (m, 3H, C^1H, $C^{10}H$), 2.08 – 1.99 (m, 2H, C^3H, $C^{3'}H$), 1.71 – 1.59 (m, 2H, C^3H, $C^{3'}H$), 1.51 – 1.40 (m, 3H, C^4H, $C^{4'}H$, C^5H), 1.33 – 1.17 (m, 3H, C^4H, $C^{4'}H$, C^5H), 0.94 (d, *J* = 6.1 Hz, 6H, $C^{11}H$, $C^{11'}H$).

^{13}C NMR ($CDCl_3$, 101 MHz) δ_C: 144.1 (C^6), 126.9 (C^8, $C^{8'}$), 126.4 (C, $C^{7'}$), 124.4 (C^9), 77.5 (C^1), 71.2 (C^{10}), 42.2 (C^2), 31.4 (C^3, $C^{3'}$), 25.5 (C^5), 21.1 (C^4, $C^{4'}$), 20.9 (C^{11}, $C^{11'}$).
HRMS (ES^+) exact mass calculated for $[M+H]^+$ ($C_{16}H_{24}ONa$) requires **m/z** 255.1719, found **m/z** 255.1715.

(1-isopropoxy-2-methylpropan-2-yl)benzene (8ad)

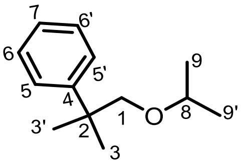

Prepared according to **General procedure F** from **7ad**. Purification via FCC (8 : 2 pentane/CH_2Cl_2) gave **8ad** as a colourless liquid (32.6 mg, 0.17 mmol, 85%).

IR 2969, 2873, 1497, 1260, 1127.

^{1}H NMR ($CDCl_3$, 400 MHz) δ_H: 7.43 – 7.38 (m, 2H, C^6H, $C^{6'}$H), 7.34 – 7.28 (m, 2H, C^5H, $C^{5'}$H), 7.22 – 7.16 (m, 1H, C^7H), 3.45 (hept, *J* = 6.1 Hz, 1H, C^8H), 3.40 (s, 2H, C^1H), 1.32 (s, 6H, C^3H, $C^{3'}$H), 1.10 (d, *J* = 6.1 Hz, 6H, C^9H, $C^{9'}$H).

^{13}C NMR ($CDCl_3$, 101 MHz) δ_C: 148.2 (C^4), 128.1 (C^5, $C^{5'}$), 126.3 (C^6, $C^{6'}$), 125.9 (C^7), 78.3 (C^1), 72.4 (C^8), 39.3 (C^2), 26.1 (C^3, $C^{3'}$), 22.2 (C^9, $C^{9'}$).

HRMS (ES^+) exact mass calculated for $[M+Na]^+$ ($C_{13}H_{20}ONa$) requires **m/z** 215.1406, found **m/z** 215.1402.

(2-(tert-butoxy)ethyl)benzene (8ae)

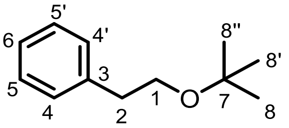

Prepared according to **General procedure E** from **7ae**. Purification via FCC (8 : 2 pentane/CH_2Cl_2) gave **8ae** as a colourless liquid (27.4 mg, 0.154 mmol, 77%).

IR 3023, 2977, 1417, 1143.

^{1}H NMR ($CDCl_3$, 500 MHz) δ_H: 7.35 – 7.19 (m, 5H, C^4H, $C^{4'}$H, C^5H, $C^{5'}$H, C^6H), 3.57 (t, *J* = 7.6 Hz, 2H, C^1H), 2.86 (t, *J* = 7.6 Hz, 2H, C^2H), 1.20 (s, 9H, C^8H, $C^{8'}$H, $C^{8''}$H).

^{13}C NMR ($CDCl_3$, 126 MHz) δ_C: 139.5 (C^3), 129.2 (C^5, $C^{5'}$), 128.4 (C^4, $C^{4'}$), 126.2 (C^6), 73.0 (C^7), 63.2 (C^1), 37.6 (C^2), 27.7 (C^8, C^8, $C^{8''}$).

HRMS (ES^+) exact mass calculated for $[M+Na]^+$ ($C_{12}H_{18}ONa$) requires **m/z** 201.1250, found **m/z** 201.1244.

1-((adamantan-1-yl)methoxy)adamantane (8af)

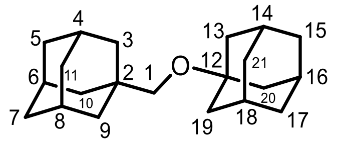

Prepared according to **General procedure F** from **7af**. Purification via FCC (8 : 2 pentane/CH_2Cl_2) gave **8af** as a colourless liquid (40.2 mg, 0.134 mmol, 67%).

IR 2906, 2850, 1451, 1354, 1154.

^{1}H NMR ($CDCl_3$, 400 MHz) δ_H: 2.94 (s, 2H), 2.14 – 2.09 (m, 3H), 1.97 – 1.91 (m, 3H), 1.74 – 1.67 (m, 9H), 1.67 – 1.55 (m, 9H), 1.52 – 1.48 (m, 6H).

^{13}C NMR ($CDCl_3$, 101 MHz) δ_C: 71.1, 70.5, 41.7, 40.0, 37.5, 36.8, 33.6, 30.7, 28.6.

HRMS (ES^+) exact mass calculated for $[M+Na]^+$ ($C_{21}H_{32}ONa$) requires **m/z** 323.2345, found **m/z** 323.2342.

Melting Point 198-196 °C.

2-ethoxyethyl)benzene (8ag)

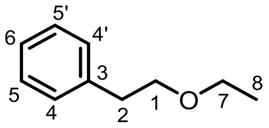

Prepared according to **General procedure G** from **7ag**. Purification via FCC (8 : 2 pentane/CH_2Cl_2) gave **8ag** as a colourless liquid (21.0 mg, 0.14 mmol, 70%). The NMR spectra and physical properties are consistent with the literature.[23]

^{1}H NMR ($CDCl_3$, 500 MHz) δ_H: 7.34 – 7.20 (m, 5H, C^4H, $C^{4'}$H, C^5H, $C^{5'}$H, C^6H), 3.67 (t, J = 7.5 Hz, 2H, C^1H), 3.54 (q, J = 7.0 Hz, 2H, C^7H), 2.95 (t, J = 7.4 Hz, 2H, C^2H), 1.25 (t, J = 7.0 Hz, 3H, C^8H).

^{13}C NMR ($CDCl_3$, 126 MHz) δ_C: 139.3, 129.0, 128.7, 126.4, 71.8, 66.3, 36.5, 15.6

(2-(benzyloxy)ethyl)benzene (8ah)

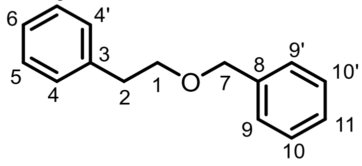

Prepared according to **General procedure G** from **7ah**. Purification via FCC (8 : 2 pentane/CH_2Cl_2) gave **8ah** as a colourless liquid (26.3 mg, 0.124 mmol, 62%). The NMR spectra and physical properties are consistent with the literature.[24]

^{1}H NMR ($CDCl_3$, 500 MHz) δ_H: 7.39 – 7.22 (m, 10H, C^4H, $C^{4'}H$ C^5H, $C^{5'}H$, C^6H, C^9H, $C^{9'}H$, $C^{10}H$, $C^{10'}H$, $C^{11}H$), 4.56 (s, 2H, C^7H), 3.74 (t, *J* = 7.2 Hz, 2H, C^1H), 2.98 (t, *J* = 7.2 Hz, 2H, C^2H)

^{13}C NMR ($CDCl_3$, 126 MHz) δ_C: 138.8, 138.3, 128.8, 128.2, 128.1, 127.4, 127.3, 126.1, 72.7, 71.0, 36.2.

2-methyltetrahydro-2H-pyran (11a)

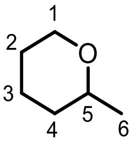

Prepared according to **General procedure G** from **10a**. Purification via FCC (8 : 2 pentane/CH_2Cl_2) gave **11a** as a colourless liquid (16.0 mg, 0.166 mmol, 83%). The NMR spectra and physical properties are consistent with the literature.[25]

^{1}H NMR ($CDCl_3$, 500 MHz) δ_H: 3.87 – 3.80 (m, 1 H), 3.74 – 3.66 (m, 2 H), 1.87 – 1.83 (m, 3 H), 1.62 – 1.53 (m, 1 H), 1.47 – 1.38 (m, 2 H), 0.90 (d, *J* = 7.5 Hz, 3 H).

^{13}C NMR ($CDCl_3$, 126 MHz) δ_C: 80.7, 67.6, 30.8, 28.5, 25.7, 10.4.

2-hexyltetrahydro-2H-pyran (11b)

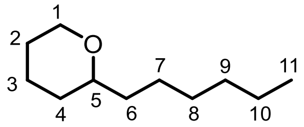

Prepared according to **General procedure G** from **10b**. Purification via FCC (8 : 2 pentane/CH_2Cl_2) gave **11b** as a colourless liquid (29.2 mg, 0.172 mmol, 86%). The NMR spectra and physical properties are consistent with the literature.[26]

^{1}H NMR ($CDCl_3$, 400 MHz) δ_H: 4.01 – 3.92 (m, 1H), 3.41 (td, *J* = 11.5, 2.6 Hz, 1H), 3.28 – 3.16 (m, 1H), 1.86 – 1.75 (m, 1H), 1.63 – 1.18 (m, 15H), 0.91 – 0.83 (m, 3H).

^{13}C NMR ($CDCl_3$, 126 MHz) δ_C: 78.1, 68.7, 36.8, 32.1, 32.0, 29.6, 26.4, 25.7, 23.8, 22.8, 14.2.

(2S,3R,4S)-3,4-bis(benzyloxy)-2-((benzyloxy)methyl)tetrahydro-2H-pyran (11c)

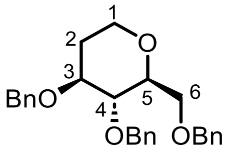

Prepared according to **General procedure G** from **10c**. Purification via FCC (8 : 2 pentane/CH_2Cl_2) gave **11c** as a colourless liquid (76.0 mg, 0.182 mmol, 91%). The NMR spectra and physical properties are consistent with the literature.[27]

IR 3030, 2920, 2859, 1453, 1361, 1093.

^{1}H NMR ($CDCl_3$, 500 MHz) δ_H: 7.40 – 7.22 (m, 13H), 7.23 – 7.14 (m, 2H), 4.90 (d, *J* = 10.8 Hz, 1H), 4.71 (d, *J* = 11.7 Hz, 1H), 4.67 – 4.57 (m, 2H), 4.57 – 4.49 (m, 2H), 4.01 (ddd, *J* = 11.7, 4.8, 1.7 Hz, 1H), 3.79 – 3.56 (m, 3H), 3.50 (dd, *J* = 9.6, 8.6 Hz, 1H), 3.44 – 3.31 (m, 2H), 2.13 – 1.99 (m, 1H), 1.79 – 1.64 (m, 1H).

^{13}C NMR ($CDCl_3$, 126 MHz) δ_C: 138.7, 138.6, 138.2, 128.6 – 128.4 (m), 128.2 – 128.1 (m), 127.8 – 127.7 (m), 81.4, 79.5, 78.6, 75.2, 73.7, 71.5, 69.6, 65.9, 31.6.

HRMS (ES^+) exact mass calculated for $[M+Na]^+$ ($C_{27}H_{30}O_4Na$) requires **m/z** 441.2036, found **m/z** 441.2046.

Ambrox (4)

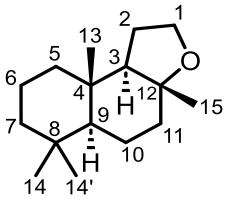

Prepared according to **General procedure G** from **10d**. Purification via FCC (8 : 2 pentane/CH_2Cl_2) gave **11d** as a colourless liquid (25.9 mg, 0.11 mmol, 55%). The NMR spectra and physical properties are consistent with the literature.[28]

^{1}H NMR ($CDCl_3$, 500 MHz) δ_H: 3.94 – 3.87 (m, 1H), 3.81 (q, *J* = 8.1 Hz, 1H), 1.93 (dt, *J* = 11.2, 3.1 Hz, 1H), 1.79 – 1.58 (m, 4H), 1.53 – 1.35 (m, 5H), 1.35 – 1.12 (m, 2H), 1.10 – 1.01 (m, 4H), 0.97 (td, *J* = 11.2, 2.4 Hz, 1H), 0.87 (s, 3H), 0.85 – 0.79 (m, 6H).

^{13}C NMR ($CDCl_3$, 126 MHz) δ_C: 80.1, 65.1, 60.3, 57.4, 42.6, 40.1, 39.9, 36.3, 33.7, 33.2, 22.8, 21.3, 20.8, 18.6, 15.2.

Eudesmin (11e) and Epieudesmin (11e')

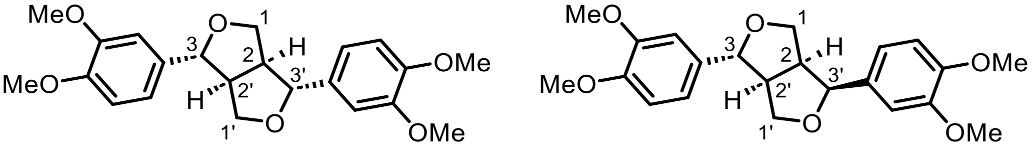

Prepared according to **General procedure G** from **10e** using 2.0 mol% of $IrCl(CO)[P(HFIP)_3]_2$ complex and 8.0 equivalent of TMDS. Purification via FCC (8 : 2 pentane/CH_2Cl_2) gave **11e** as a colourless liquid (38.6 mg, 0.10 mmol, 50%). The NMR spectra and physical properties are consistent with the literature.[29-30]

Eudesmin:

^{1}H NMR ($CDCl_3$, 500 MHz) δ_H: 6.94 – 6.81 (m, 6H), 4.76 (d, *J* = 4.2 Hz, 2H), 4.32 – 4.20 (m, 2H), 3.90 (s, 6H), 3.92 – 3.88 (m, 2H), 3.88 (s, 6H), 3.16 – 3.08 (m, 2H).

^{13}C NMR ($CDCl_3$, 126 MHz) δ_C: 149.4, 148.8, 133.7, 118.4, 111.2, 109.4, 85.9, 71.9, 56.1 (d, *J* = 2.9 Hz), 54.3.

Epieudesmin:

^{1}H NMR ($CDCl_3$, 500 MHz) δ_H: 6.98 – 6.80 (m, 6H), 4.88 (d, *J* = 5.6 Hz, 1H), 4.45 (d, *J* = 7.2 Hz, 1H), 4.14 (d, *J* = 9.1 Hz, 1H), 3.93 – 3.83 (m, 14H), 3.59 – 3.50 (m, 1H), 3.34 – 3.29 (m, 1H), 2.96 – 2.88 (m, 1H).

^{13}C NMR ($CDCl_3$, 126 MHz) δ_C 149.4, 148.9 (2 x C), 148.2, 133.8, 131.1, 118.6, 117.9, 111.1 (d, *J* = 4.0 Hz, 2 x C), 109.3, 109.1, 87.8, 82.2, 71.2, 69.9, 56.2 – 55.9 (3 x C), 54.7, 50.3.

7. Mechanistic experiments

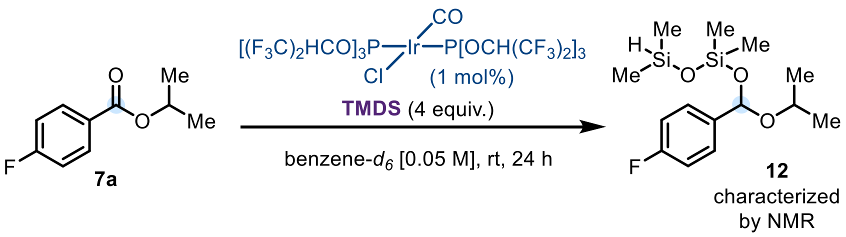

To a solution of ester **7a** (4.6 mg, 0.025 mmol) and IrCl(CO)(P[OCH(CF_3)$_2$]$_3$)$_2$ **Ir-3** (0.33 mg, 0.25 μmol) in 0.5 mL of benzene-d_6 was added TMDS (22.0 μL, 0.125 mmol, 5.0 equiv.) in an NMR tube at room temperature. After 24 h, the formation of mixed acetal **12** was confirmed by ^{1}H NMR spectroscopic analysis. **^{1}H NMR** ($CDCl_3$, 500 MHz) δ_H: 7.45 – 7.29 (m, 2H), 6.86 – 6.78 (m, 2H), 5.87 (s, 1H), 5.06 – 5.00 (m, 1H), 3.96 (p, J = 6.1 Hz, 1H), 1.19 (d, J = 6.2 Hz, 3H), 1.08 (d, J = 6.1 Hz, 3H).

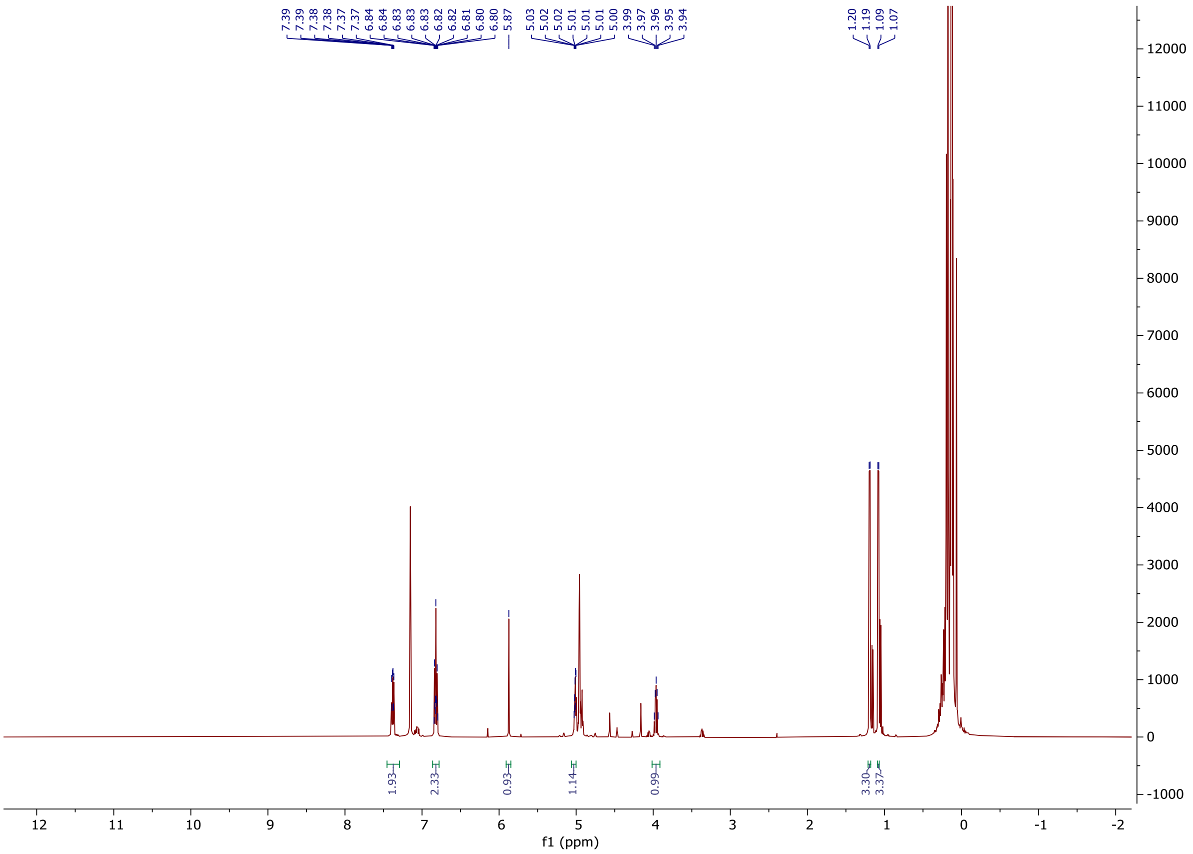

8. Robustness Screen

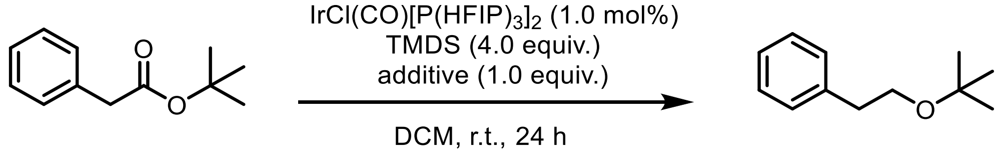

additive	*additive conversion/ %*	*product yield/ %*
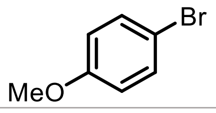	2	86
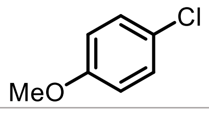	0	85
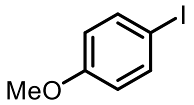	17	92
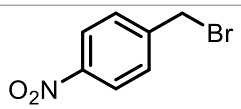	25	26
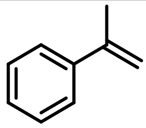	0	98
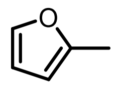	46	82
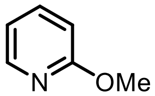	11	5
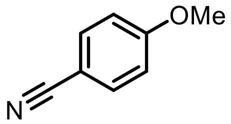	55	8
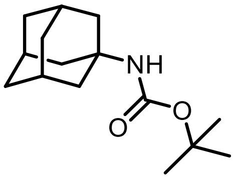	0	24

Table S2: Robustness screen of additives; 100% starting material conversion in all cases. Yields and conversions were determined via ^{1}H NMR using 1,2,4,5-tetrachloro-3-nitrobenzene as an internal standard. To a stirred solution of **8ae** (0.20 mmol), additive (0.20 mmol, 1.0 equiv.), and IrCl(CO)[P(HFIP)$_3$]$_2$ complex (2.60 mg, 1 mol%) in dry CH_2Cl_2 (1.0 M, 0.20 mL) in a 1.75 mL vial was added TMDS (0.80 mmol, 4 equiv.) at room temperature, which resulted in a gentle effervescence. The vial was purged with hydrogen gas before being capped and left to stir at rt for 24 h. Internal standard (0.10 mmol, 0.5 equiv.) was added and ^{1}H NMR was run to determine conversion and yield.

9. Scale Up

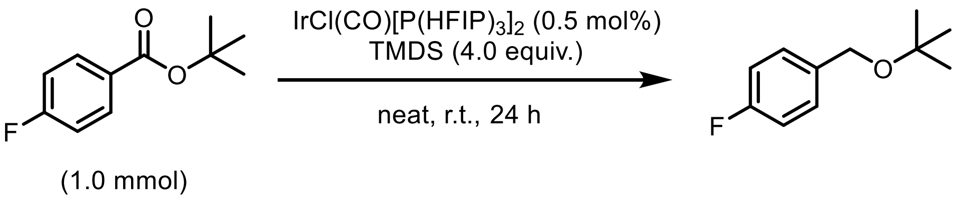

To a stirred solution of **7b** (1.0 mmol, 196.2 mg) and IrCl(CO)[P(HFIP)$_3$]$_2$ complex (6.92 mg, 0.5 mol%) in a 3.5 mL vial was added TMDS (4.0 mmol, 4.0 equiv.) at room temperature, which resulted in a gentle effervescence. The vial was purged with hydrogen gas before being capped and left to stir at rt for 24 h. The crude reaction mixture was purified by chromatography on silica gel (8:2 pentane/CH_2Cl_2) to yield product **8b** as a colourless liquid (167 mg, 0.92 mmol, 92%).

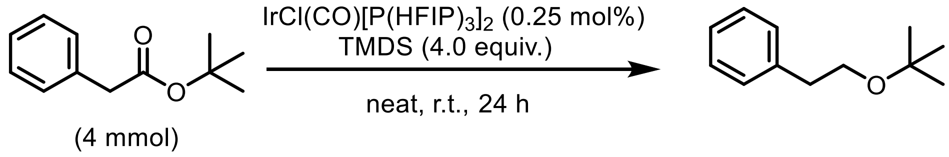

To a stirred solution of **7ae** (4.0 mmol, 769.0 mg) and IrCl(CO)[P(HFIP)$_3$]$_2$ complex (13.85 mg, 0.25 mol%) in a 10 mL vial was slowly added TMDS (16 mmol, 4.0 equiv.) at room temperature, which resulted in an effervescence and exotherm. The vial was purged with hydrogen gas before being capped and left to stir at rt for 24 h. The crude reaction mixture was purified by chromatography on silica gel (8:2 pentane/CH_2Cl_2) to yield product **8ae** as a colourless liquid (524 mg, 2.94 mmol, 74%).

10. References

[1] D. Roberto, E. Cariati, R. Psaro, R. Ugo, *Organometallics* **1994**, *13*, 4227-4231.

[2] A. Tahara, Y. Miyamoto, R. Aoto, K. Shigeta, Y. Une, Y. Sunada, Y. Motoyama, H. Nagashima, *Organometallics* **2015**, *34*, 4895-4907.

[3] B. A. Tschaen, J. R. Schmink, G. A. Molander, *Org. Lett.* **2013**, *15*, 500-503.

[4] L. Huck, A. De La Hoz, A. Díaz-Ortiz, J. Alcázar, *Org. Lett.* **2017**, *19*, 3747-3750.

[5] N. A. LaBerge, J. A. Love, *Eur. J. Org. Chem.* **2015**, *2015*, 5546-5553.

[6] W. Su, P. Xu, T. Ritter, *Angew. Chem.* **2021**, *133*, 24214-24219.

[7] B. Xing, C. Ni, J. Hu, *Angew. Chem. Int. Ed.* **2018**, *57*, 9896-9900.

[8] Z. Xin, T. M. Gøgsig, A. T. Lindhardt, T. Skrydstrup, *Org. Lett.* **2012**, *14*, 284-287.

[9] S. Cheng, J. Chen, W. Gao, H. Jin, J. Ding, H. Wu, *Journal of Chemical Research* **2010**, *34*, 130-132.

[10] M. Blumel, J.-M. Noy, D. Enders, M. H. Stenzel, T. V. Nguyen, *Org. Lett.* **2016**, *18*, 2208-2211.

[11] F. Hou, X.-C. Wang, Z.-J. Quan, *Organic & Biomolecular Chemistry* **2018**, *16*, 9472-9476.

[12] M. Majek, A. Jacobi von Wangelin, *Angew. Chem. Int. Ed.* **2015**, *54*, 2270-2274.

[13] M. Szostak, M. Spain, D. J. Procter, *J. Am. Chem. Soc.* **2014**, *136*, 8459-8466.

[14] P. Saxena, M. Kapur, *Chemistry–An Asian Journal* **2018**, *13*, 861-870.

[15] L. Lu, R. Shi, L. Liu, J. Yan, F. Lu, A. Lei, *Chem. Eur. J.* **2016**, *22*, 14484-14488.

[16] T. Shono, O. Ishige, H. Uyama, S. Kashimura, *J. Org. Chem.* **1986**, *51*, 546-549.

[17] U. Funke, H. Jia, S. Fischer, M. Scheunemann, J. Steinbach, *Journal of Labelled Compounds and Radiopharmaceuticals: The Official Journal of the International Isotope Society* **2006**, *49*, 745-755.

[18] H.-A. Cho, Y.-K. Lee, S.-H. Kim, *Synlett* **2022**, *33*, 1295-1301.

[19] M. Bakos, Á. Gyömöre, A. Domján, T. Soós, *Angew. Chem. Int. Ed.* **2017**, *56*, 5217-5221.

[20] S. Hosokawa, M. Toya, A. Noda, M. Morita, T. Ogawa, Y. Motoyama, *ChemistrySelect* **2018**, *3*, 2958-2961.

[21] L. Serusi, M. Bonnans, A. Luridiana, F. Secci, P. Caboni, T. Boddaert, D. J. Aitken, A. Frongia, *Adv. Synth. Catal.* **2019**, *361*, 1908-1912.

[22] A. Pelosi, D. Lanari, A. Temperini, M. Curini, O. Rosati, *Adv. Synth. Catal.* **2019**, *361*, 4527-4539.

[23] N. Sakai, T. Moriya, T. Konakahara, *J. Org. Chem.* **2007**, *72*, 5920-5922.

[24] T. Yamada, S. Tsukagoshi, O. Kitagawa, *Tetrahedron Lett.* **2017**, *58*, 317-320.

[25] X. Zhu, G. Li, F. Xu, Y. Zhang, M. Xue, Q. Shen, *Tetrahedron* **2017**, *73*, 1451-1458.

[26] K. Matsubara, T. Iura, T. Maki, H. Nagashima, *J. Org. Chem.* **2002**, *67*, 4985-4988.

[27] H. Narama, M. Funabashi*, *J. Carbohydr. Chem.* **2001**, *20*, 257-262.

[28] S. Yang, H. Tian, B. Sun, Y. Liu, Y. Hao, Y. Lv, *Scientific reports* **2016**, *6*, 32650.

[29] A. Rahim, Y. Saito, S. Fukuyoshi, K. Miyake, M. Goto, C.-H. Chen, G. Alam, K.-H. Lee, K. Nakagawa-Goto, *J. Nat. Prod.* **2020**, *83*, 2931-2939.

[30] G. Bravo-Arrepol, J. Becerra, L. Ortiz, J. Cabrera-Pardo, B. Schmidt, M. Heydenreich, A. Kelling, E. Sperlich, T. M. Karpiński, C. Paz, *Natural Product Research* **2023**, *37*, 2466-2471.

11. NMR Spectra

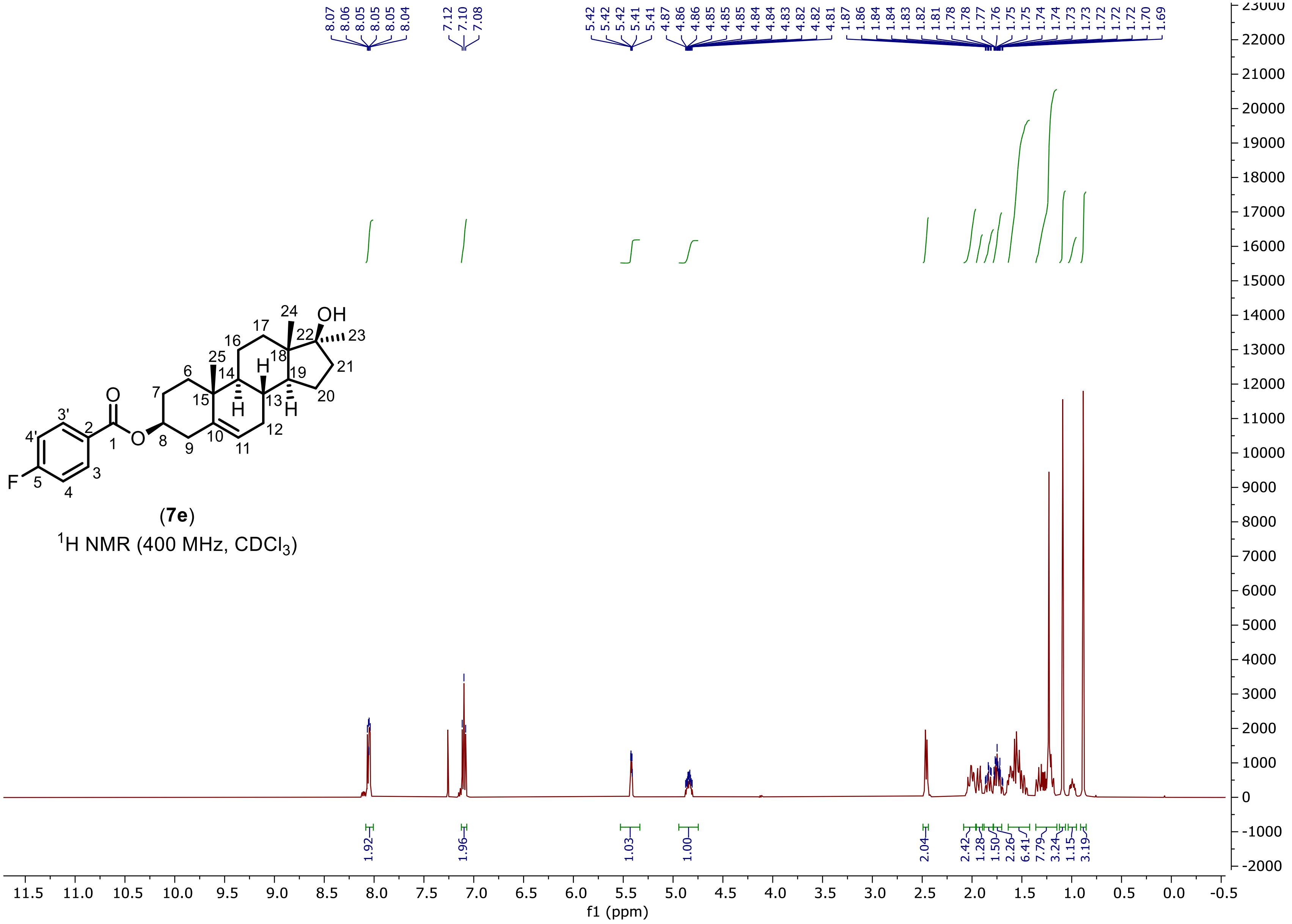

(**7e**)

^{1}H NMR (400 MHz, $CDCl_3$)

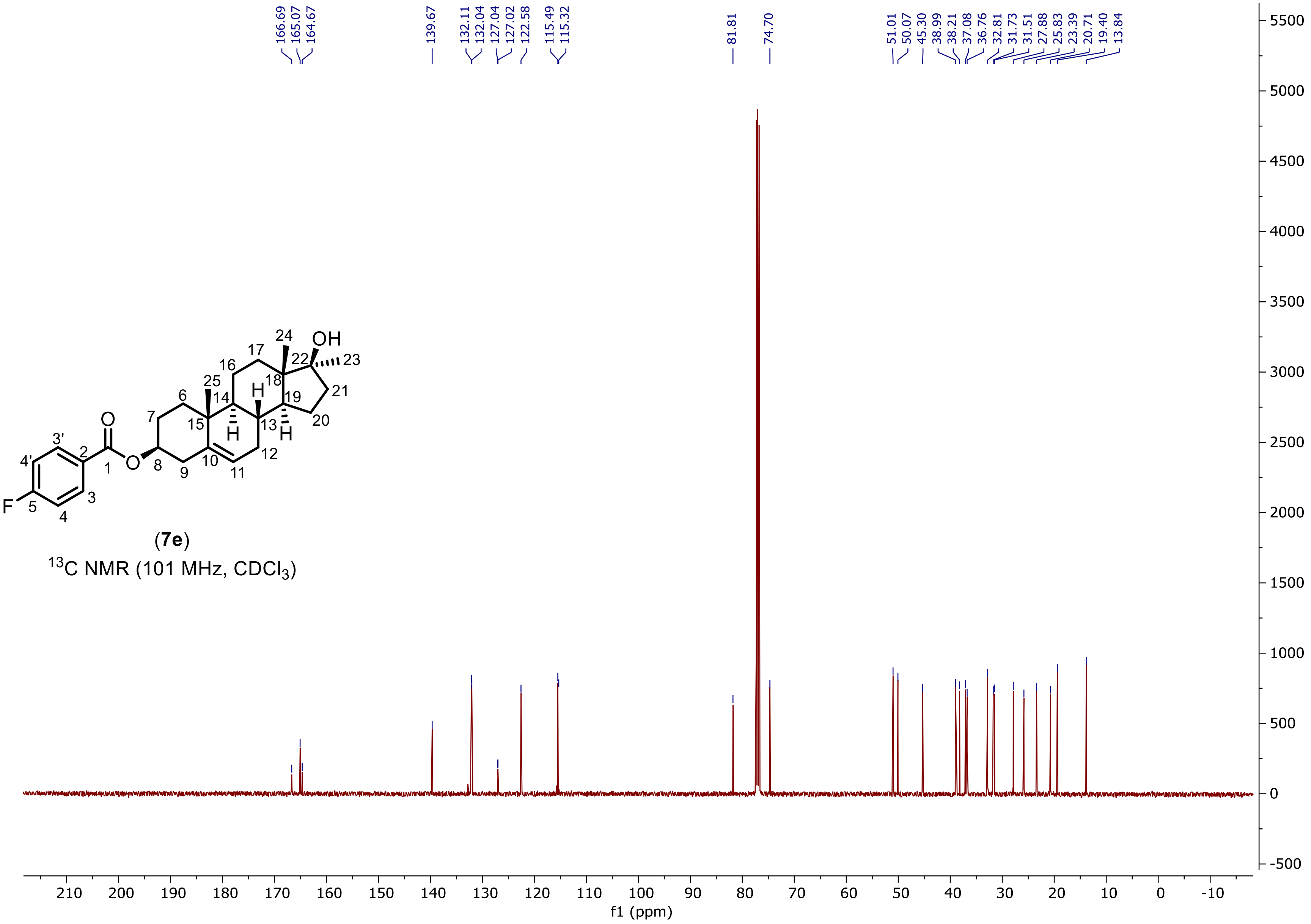

(**7e**)

^{13}C NMR (101 MHz, $CDCl_3$)

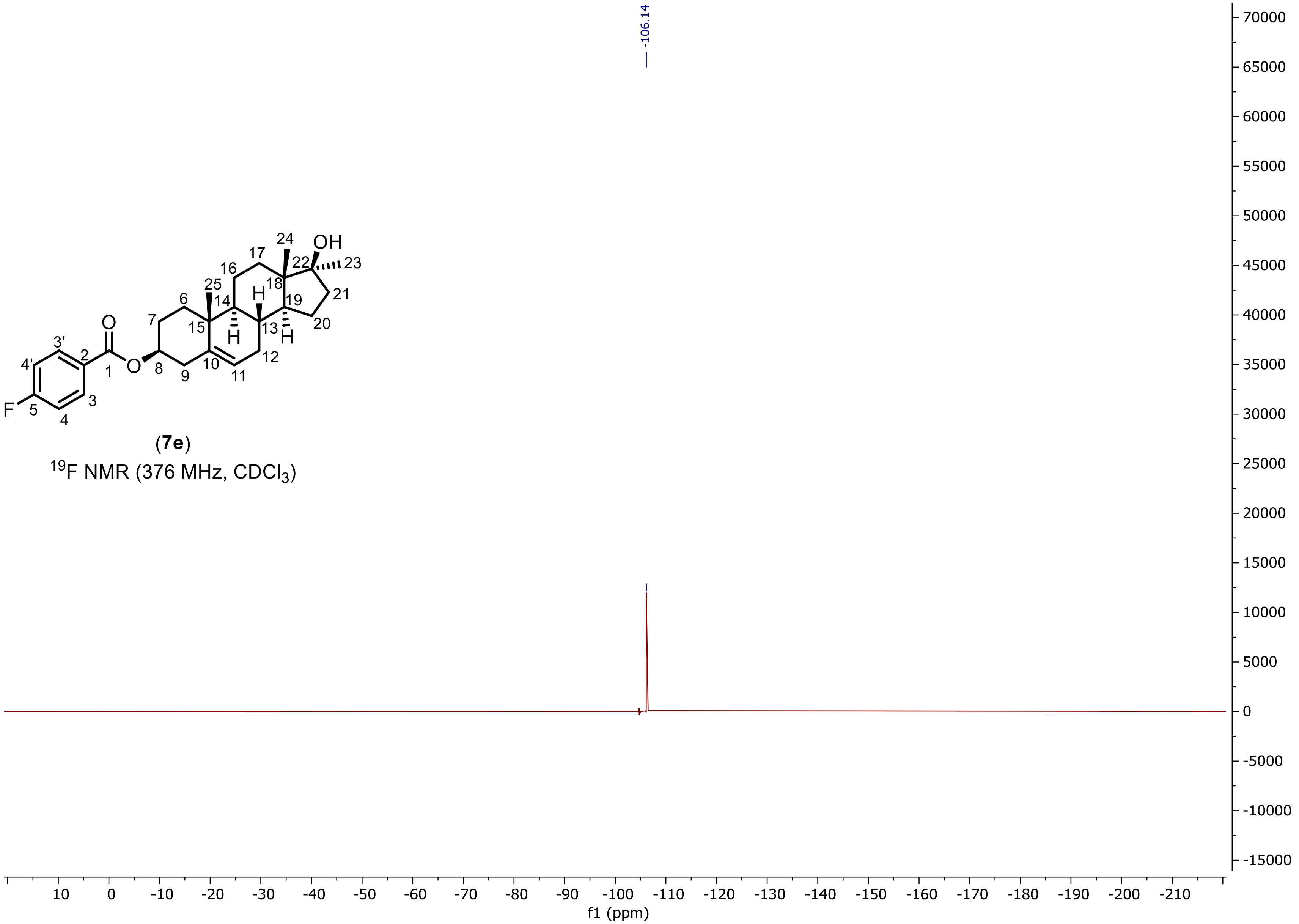

(**7e**)

^{19}F NMR (376 MHz, $CDCl_3$)

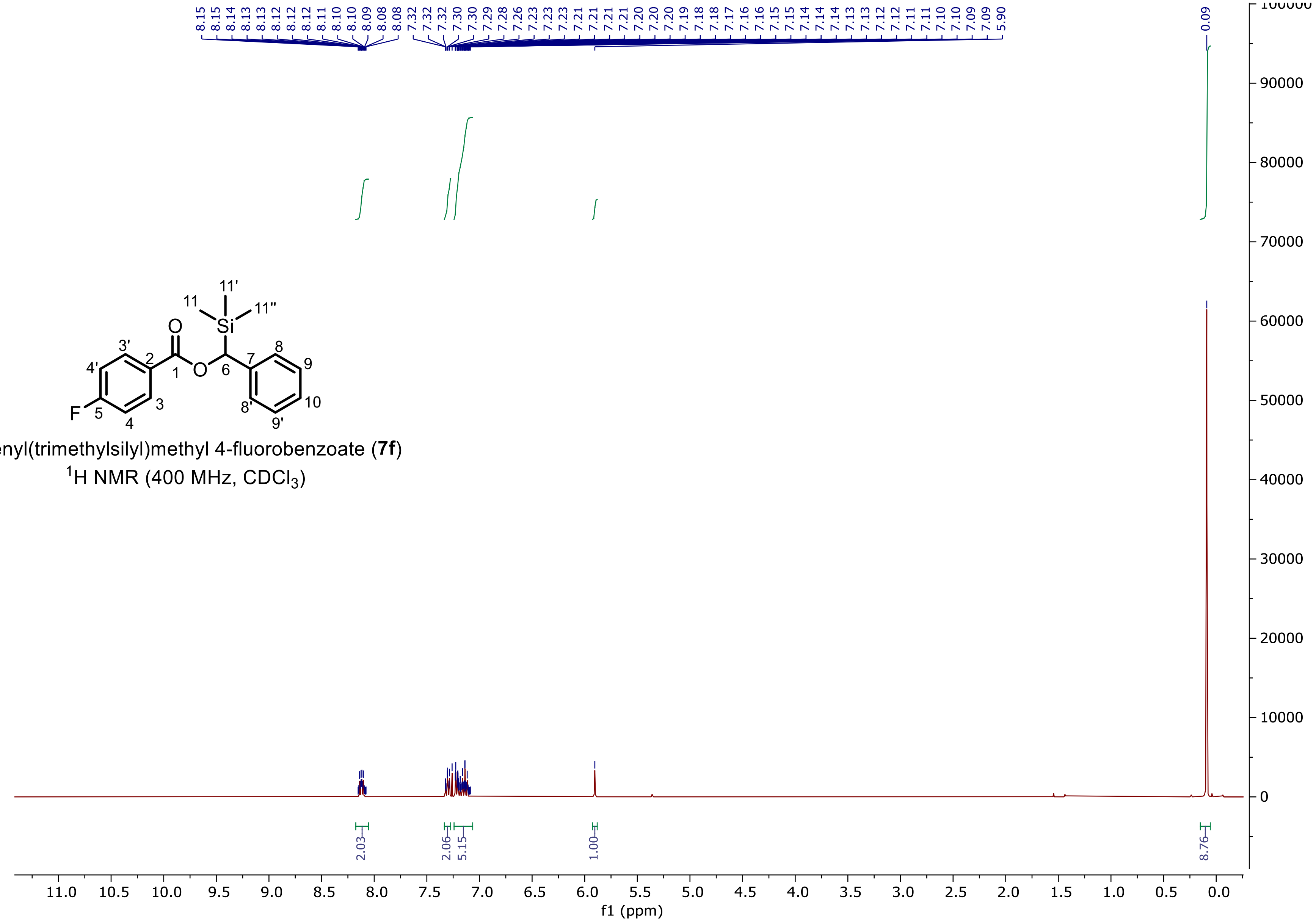

phenyl(trimethylsilyl)methyl 4-fluorobenzoate (**7f**)

^{1}H NMR (400 MHz, $CDCl_3$)

phenyl(trimethylsilyl)methyl 4-fluorobenzoate (**7f**)
^{13}C NMR (101 MHz, $CDCl_3$)

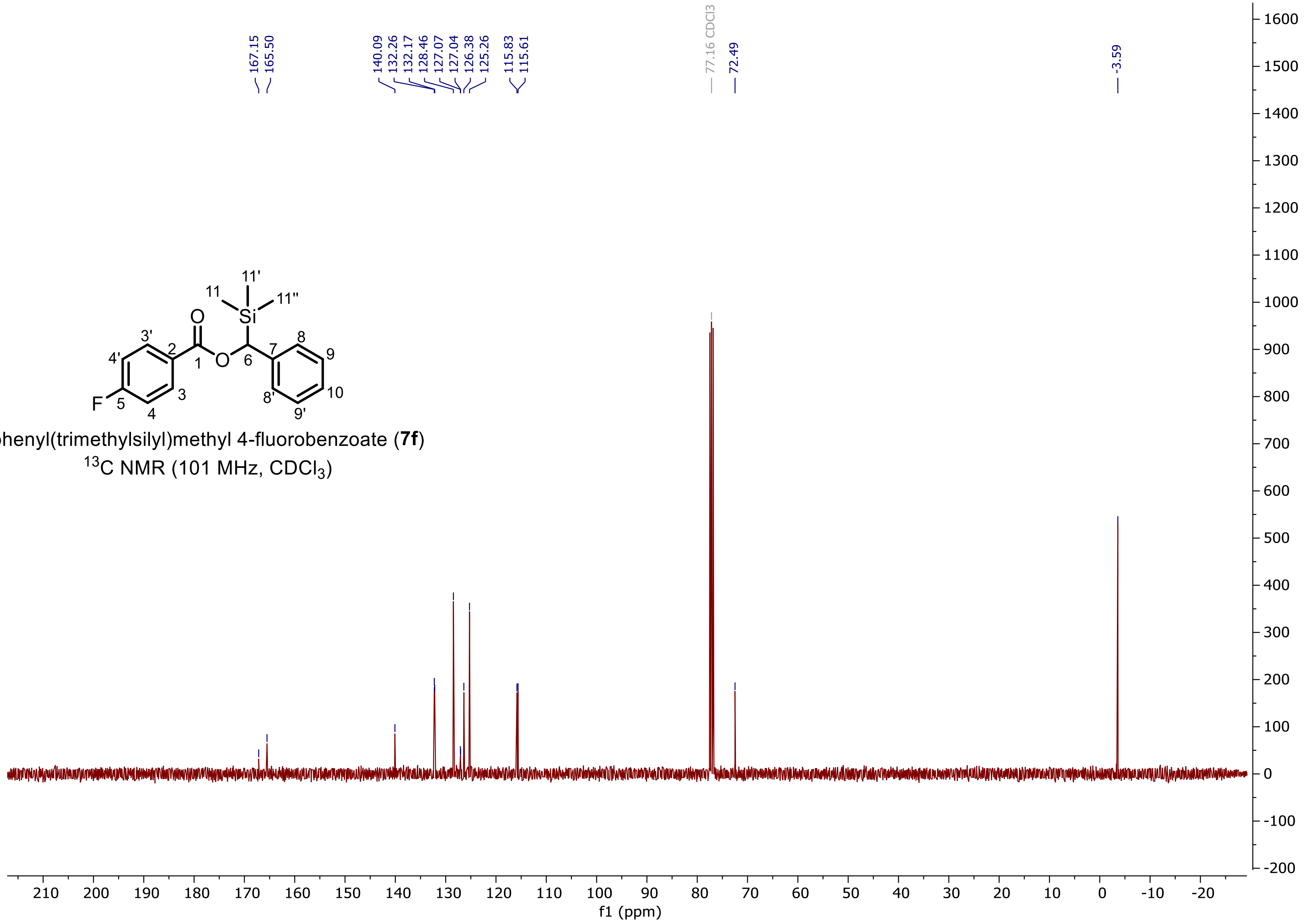

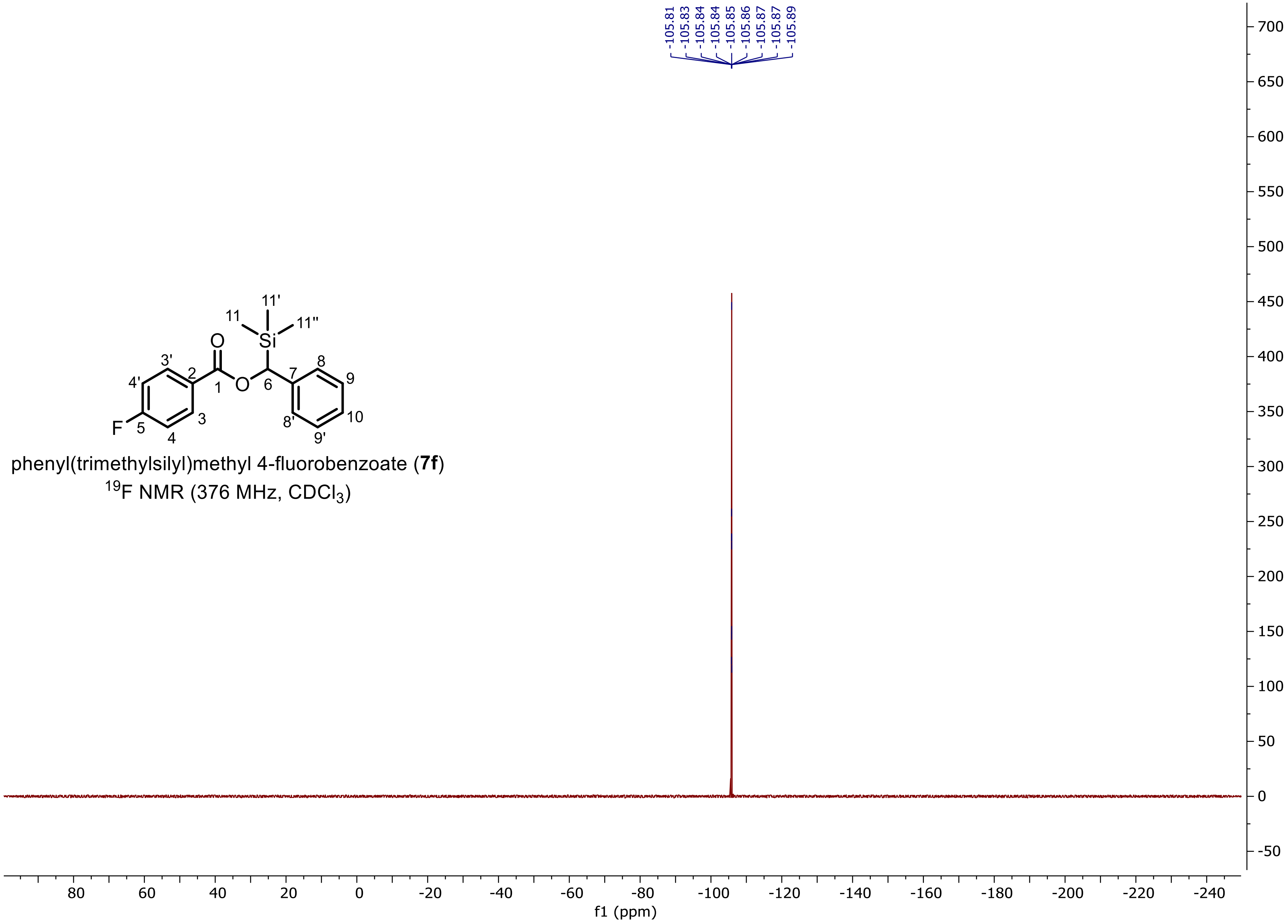

phenyl(trimethylsilyl)methyl 4-fluorobenzoate (**7f**)
^{19}F NMR (376 MHz, $CDCl_3$)

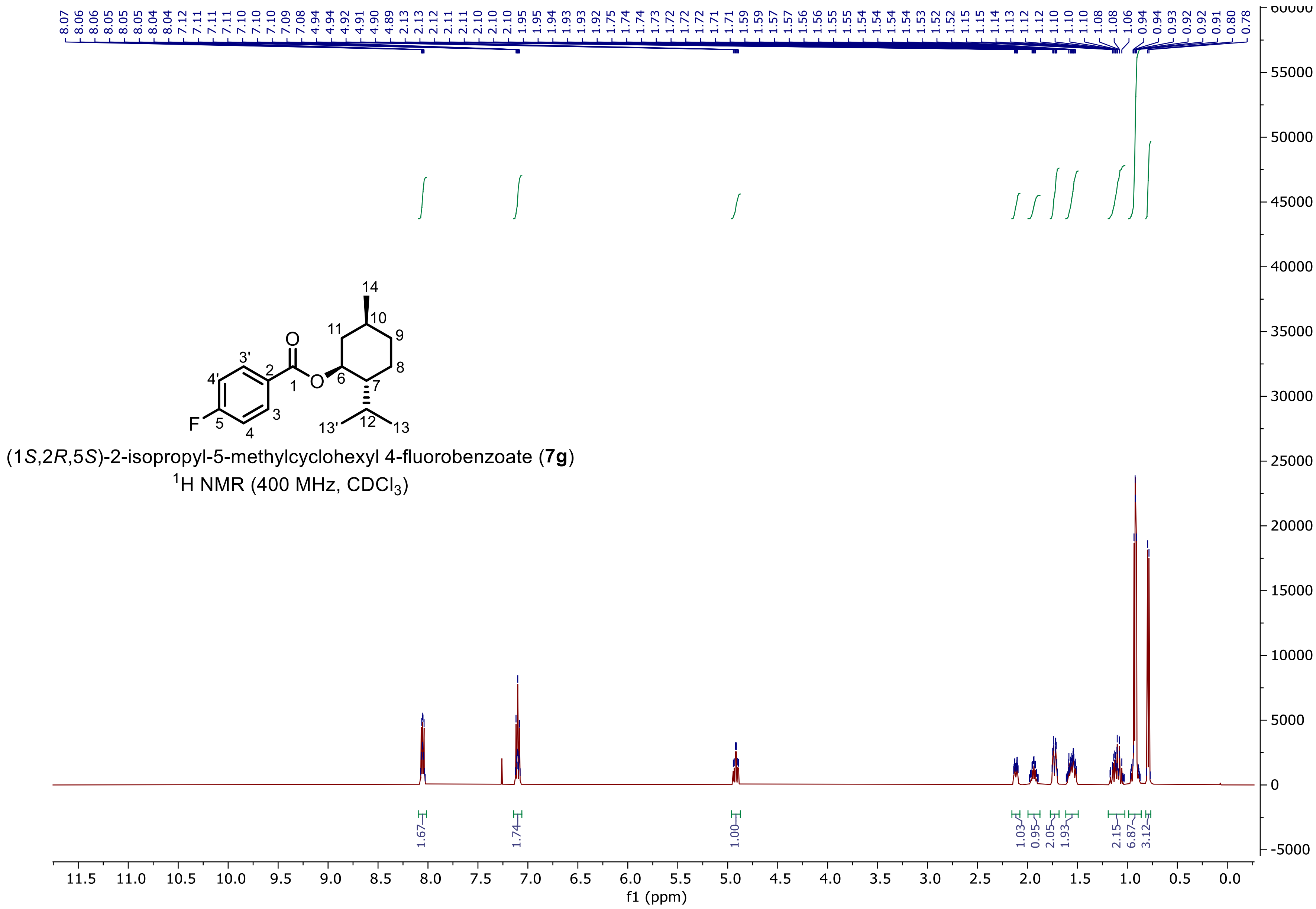

(1*S*,2*R*,5*S*)-2-isopropyl-5-methylcyclohexyl 4-fluorobenzoate (**7g**)

^{1}H NMR (400 MHz, $CDCl_3$)

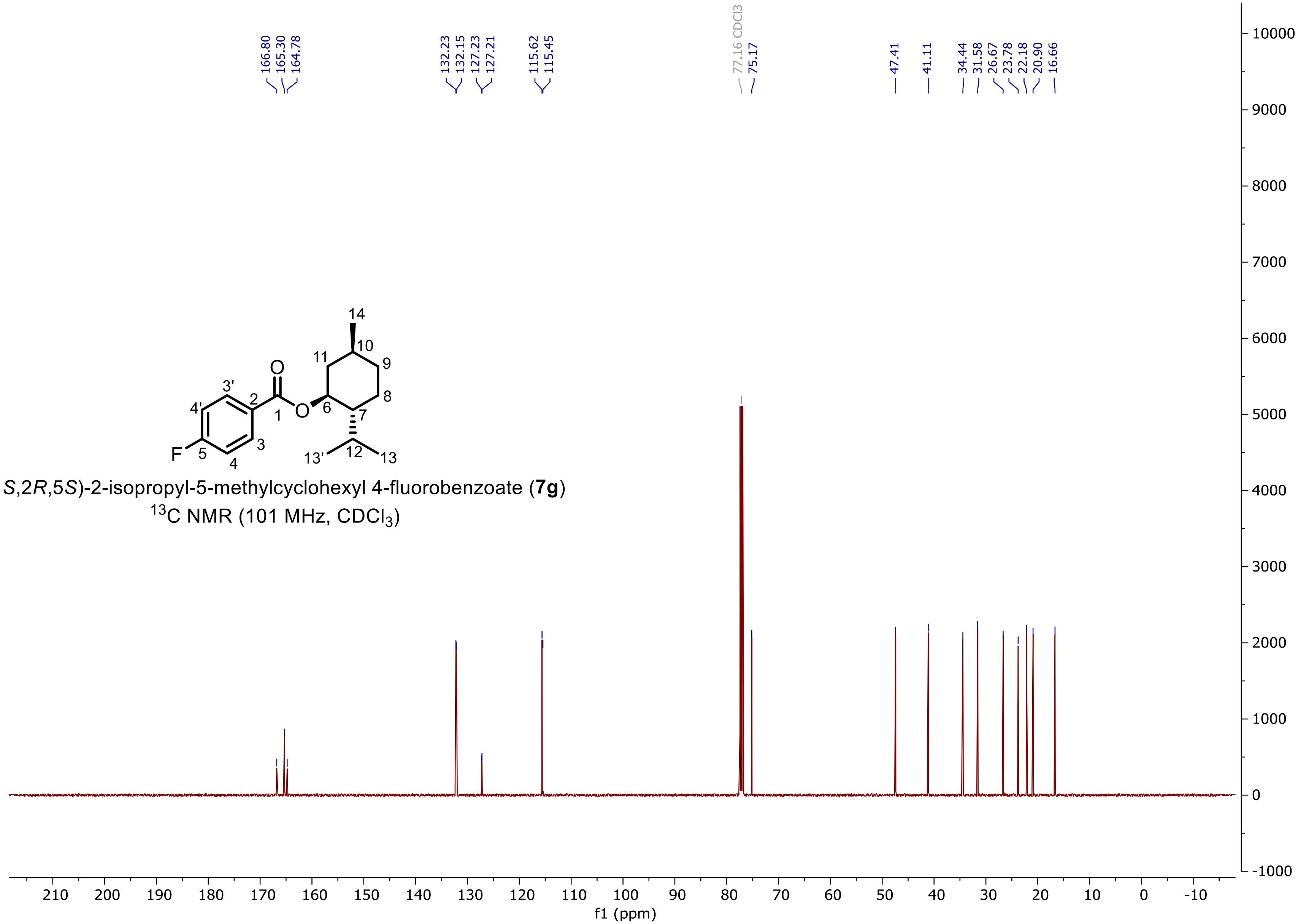

(1*S*,2*R*,5*S*)-2-isopropyl-5-methylcyclohexyl 4-fluorobenzoate (**7g**)
^{13}C NMR (101 MHz, $CDCl_3$)

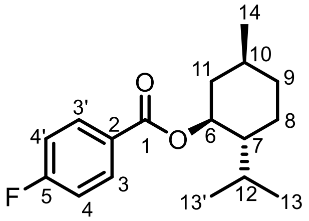

(1*S*,2*R*,5*S*)-2-isopropyl-5-methylcyclohexyl 4-fluorobenzoate (**7g**)
^{19}F NMR (376 MHz, $CDCl_3$)

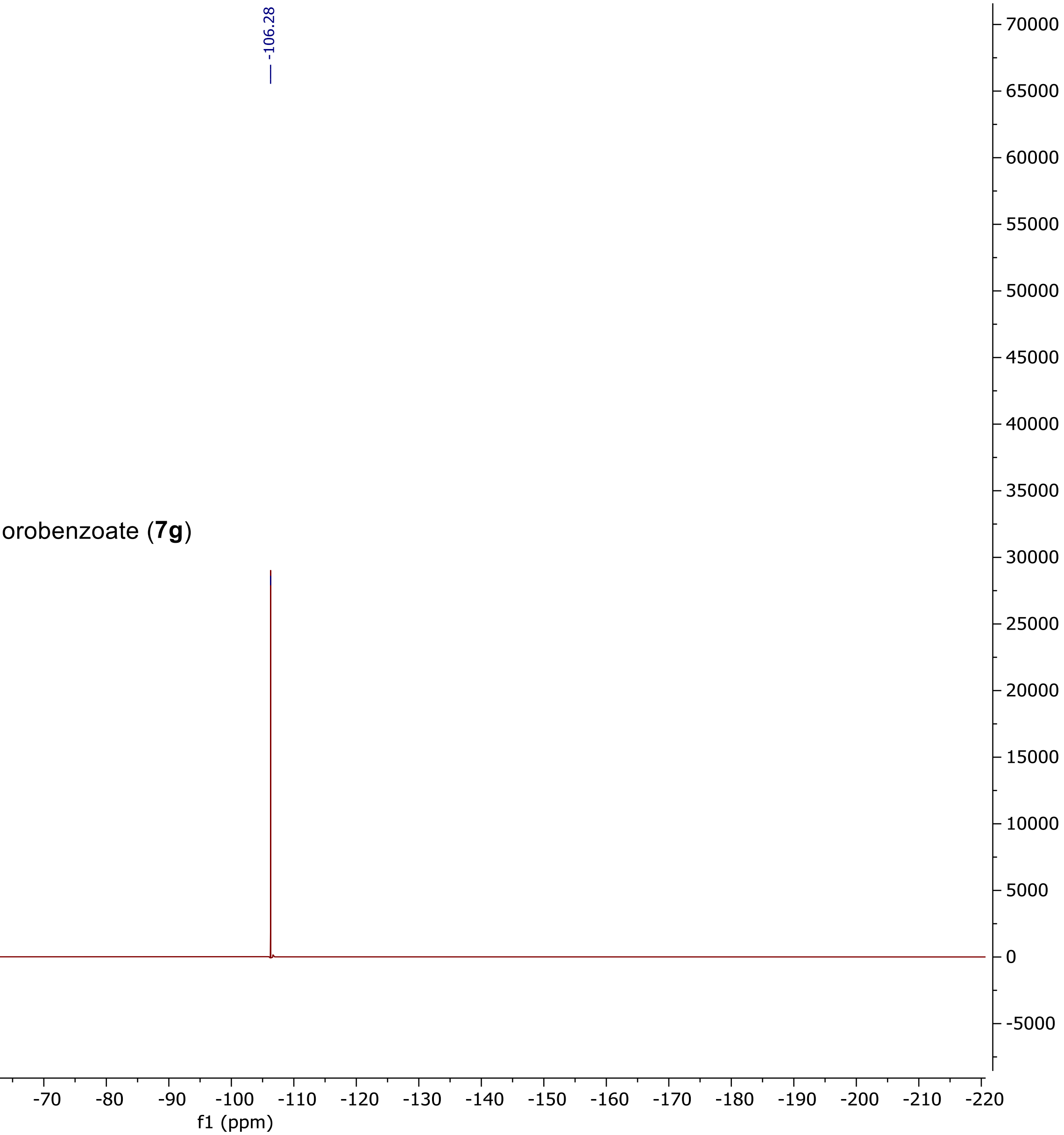

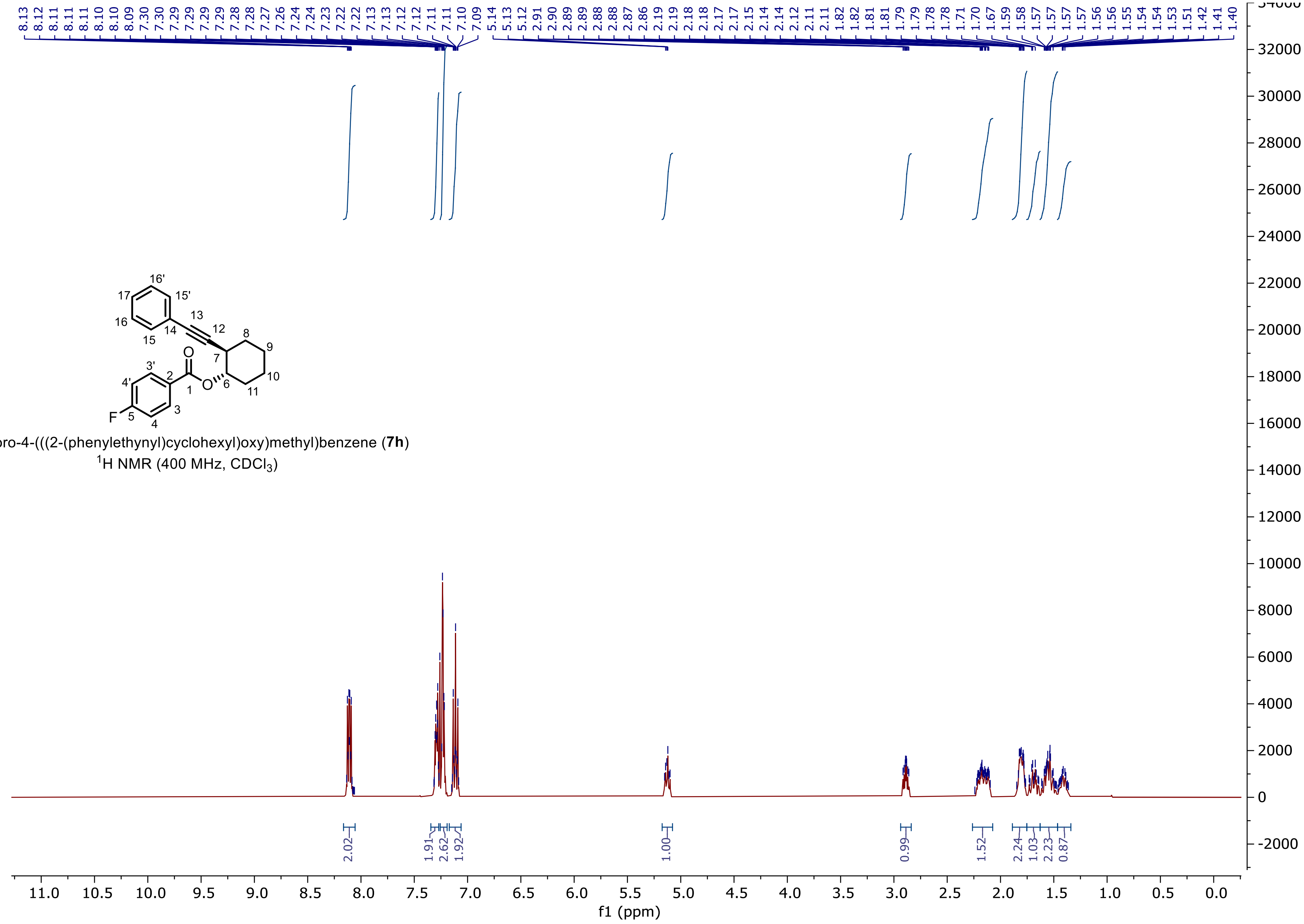

1-fluoro-4-(((2-(phenylethynyl)cyclohexyl)oxy)methyl)benzene (**7h**)
^{1}H NMR (400 MHz, $CDCl_3$)

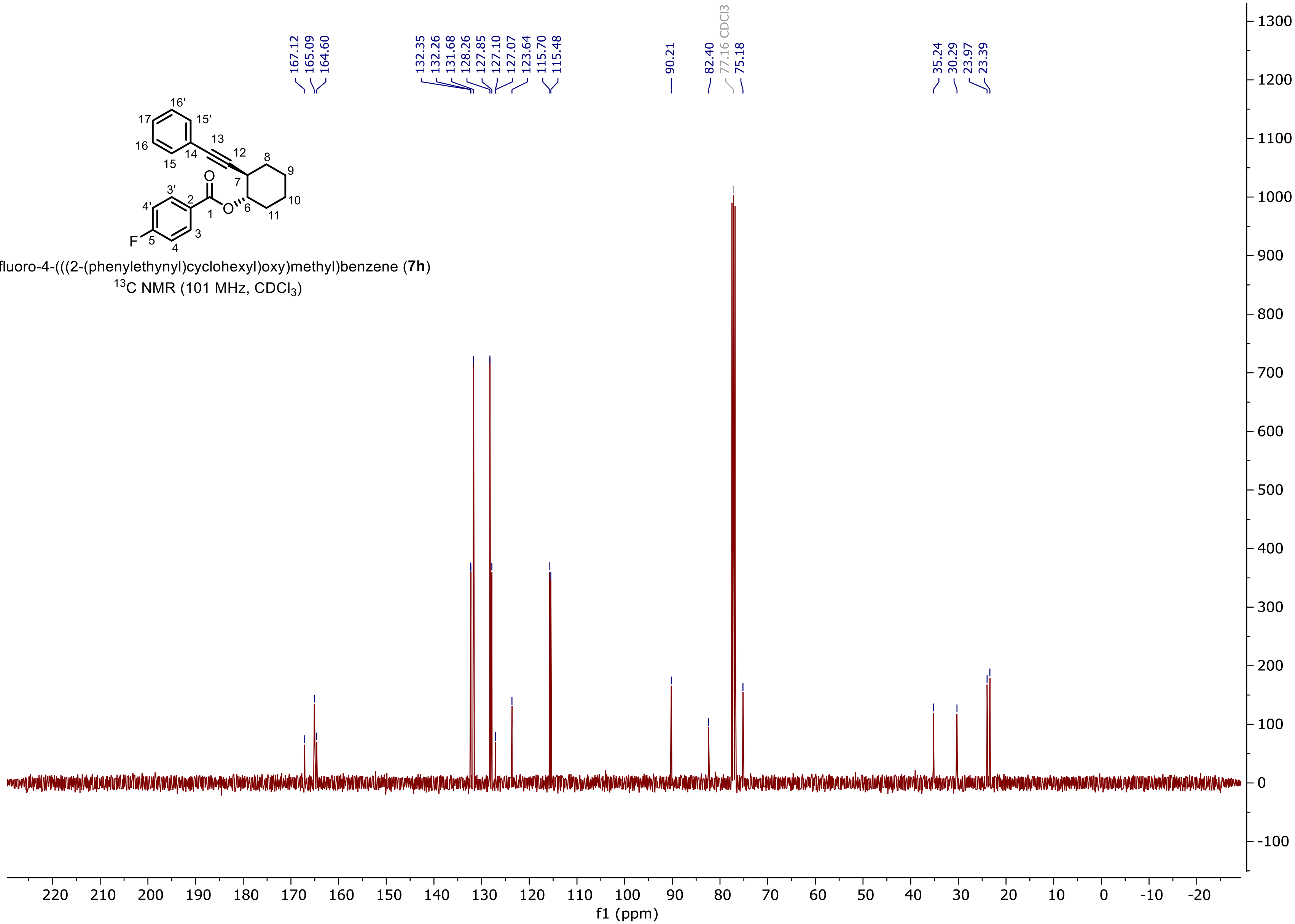

1-fluoro-4-(((2-(phenylethynyl)cyclohexyl)oxy)methyl)benzene (**7h**)

^{13}C NMR (101 MHz, CDCl$_3$)

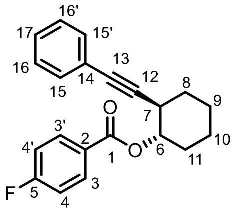

1-fluoro-4-(((2-(phenylethynyl)cyclohexyl)oxy)methyl)benzene (**7h**)

^{19}F NMR (376 MHz, $CDCl_3$)

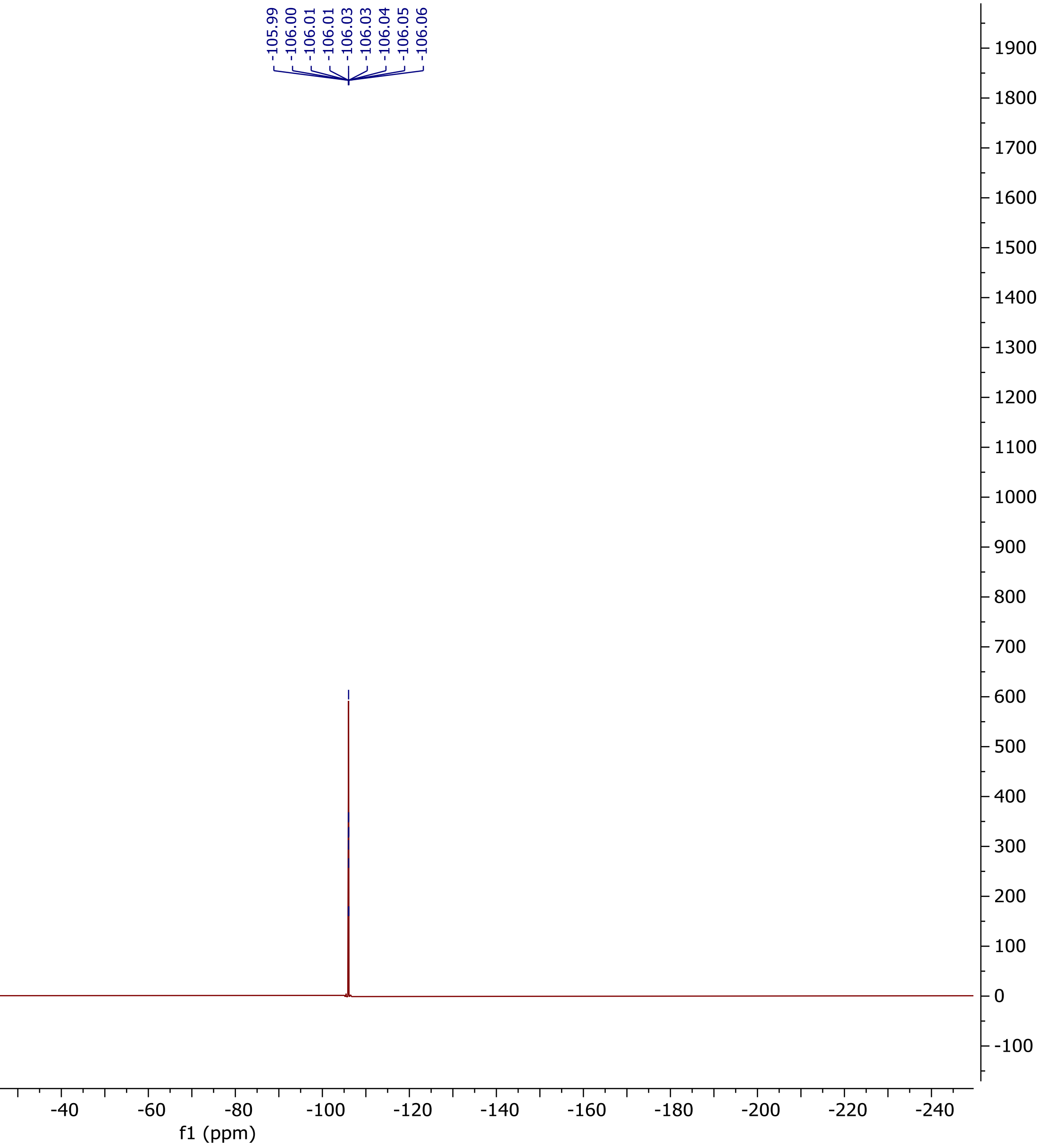

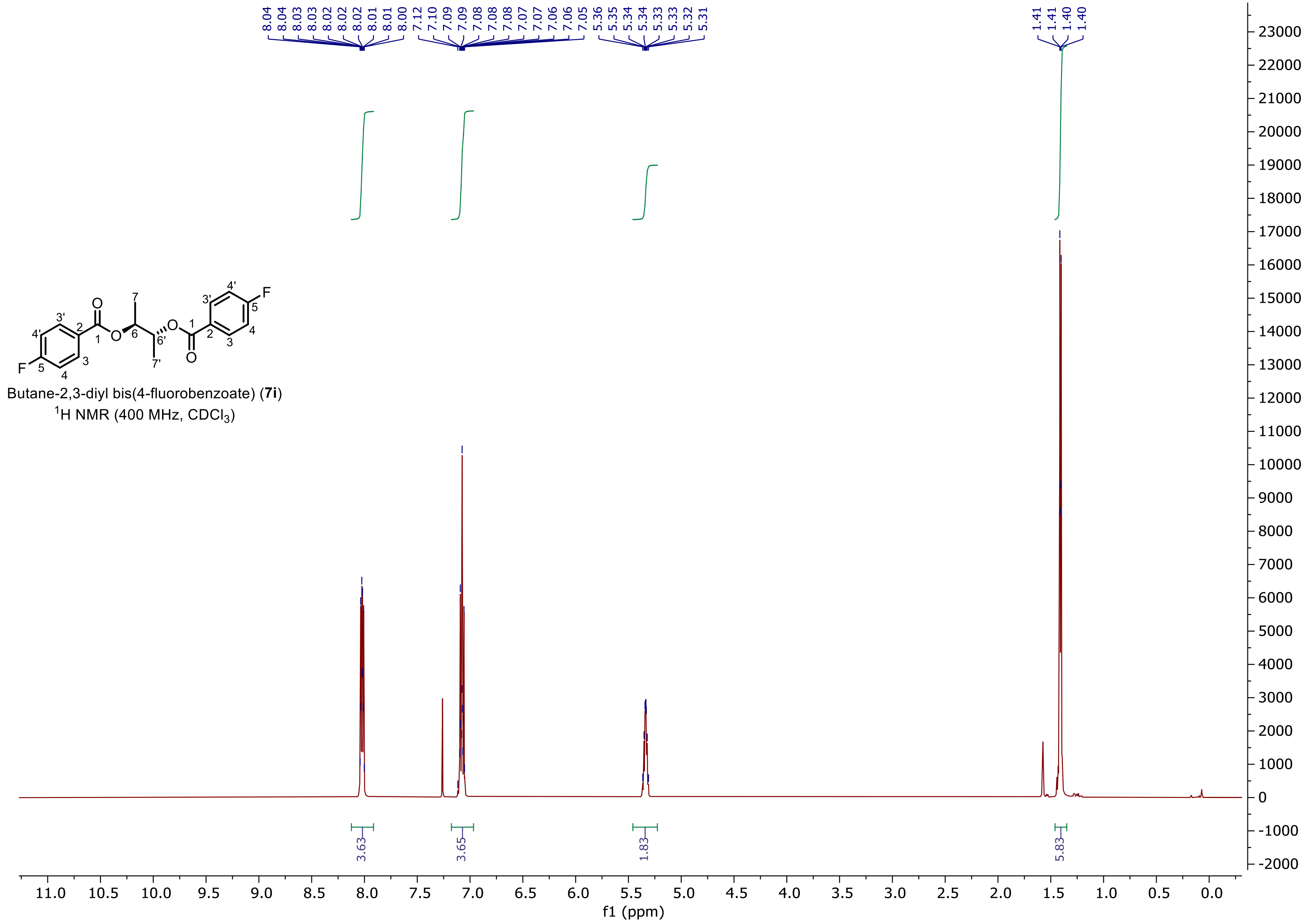
8.04 8.04 8.03 8.03 8.02 8.02 8.02 8.01 8.01 8.00 7.12 7.10 7.09 7.09 7.08 7.08 7.08 7.07 7.07 7.06 7.06 7.05 5.36 5.35 5.34 5.34 5.33 5.33 5.32 5.31
1.41 1.41 1.40 1.40
Butane-2,3-diyl bis(4-fluorobenzoate) (**7i**)
^{1}H NMR (400 MHz, $CDCl_3$)
3.63
3.65
1.83
5.83
f1 (ppm)

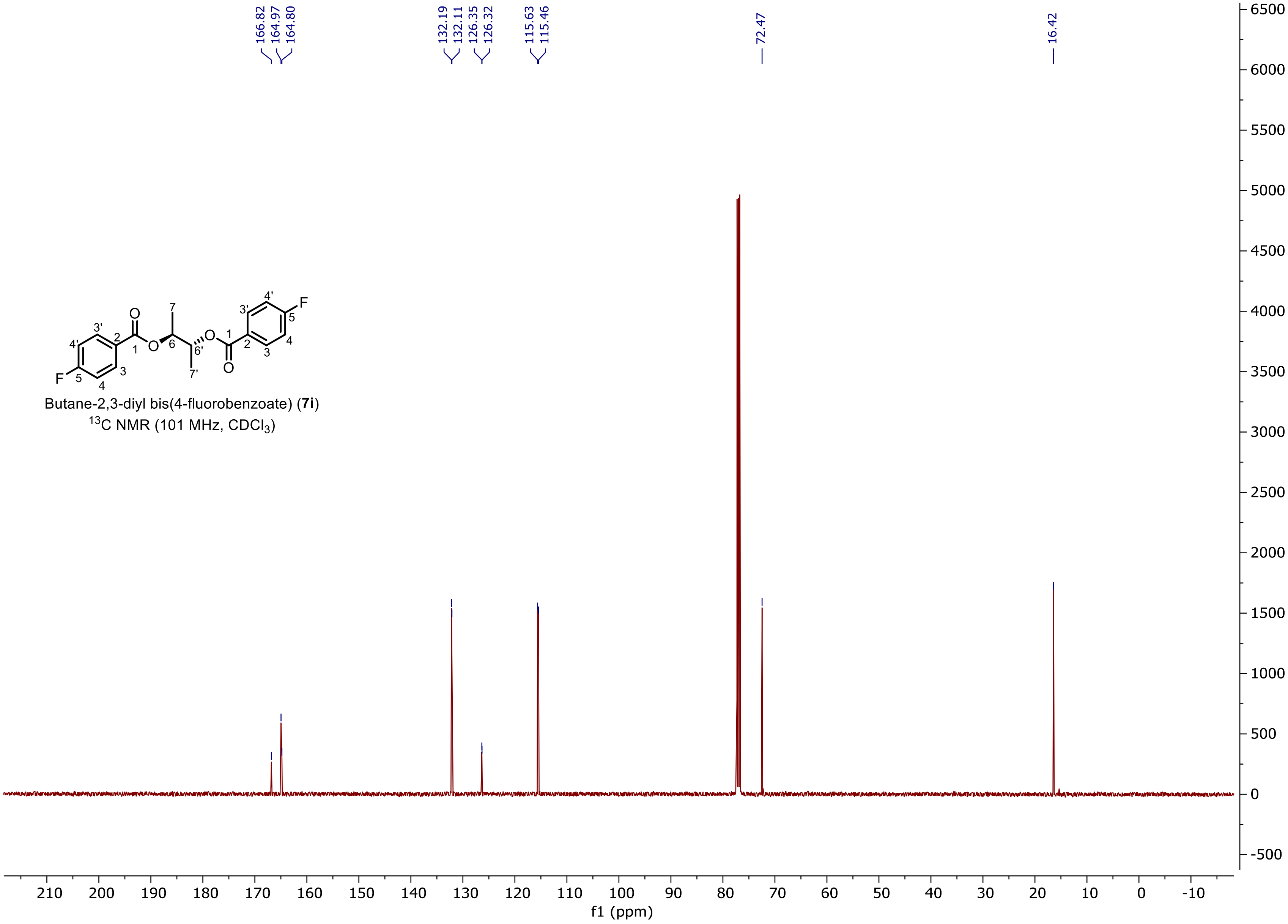

166.82
164.97
164.80
132.19
132.11
126.35
126.32
115.63
115.46
72.47
16.42
Butane-2,3-diyl bis(4-fluorobenzoate) (7i)
13C NMR (101 MHz, CDCl3)
f1 (ppm)

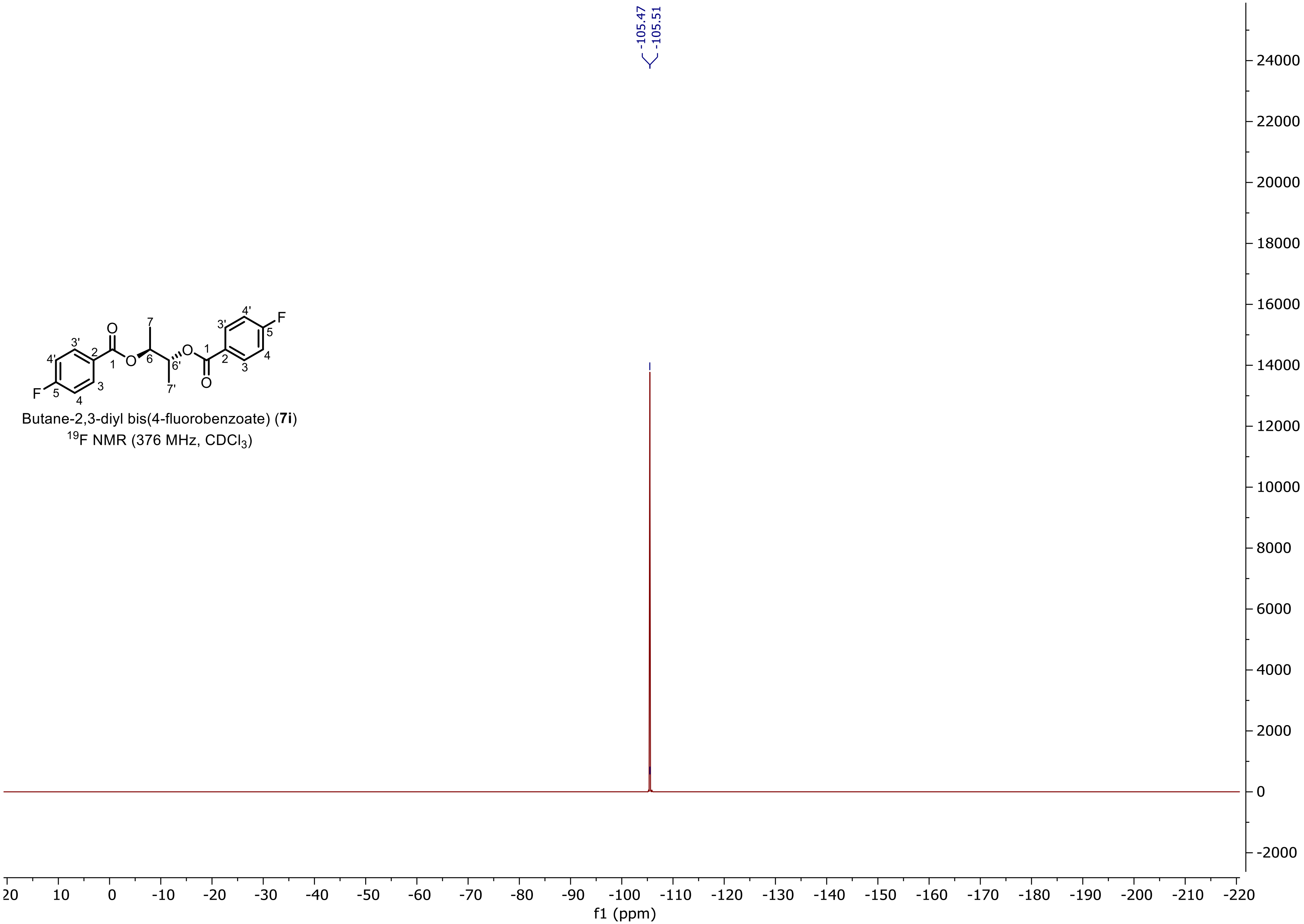

Butane-2,3-diyl bis(4-fluorobenzoate) (**7i**)
^{19}F NMR (376 MHz, $CDCl_3$)

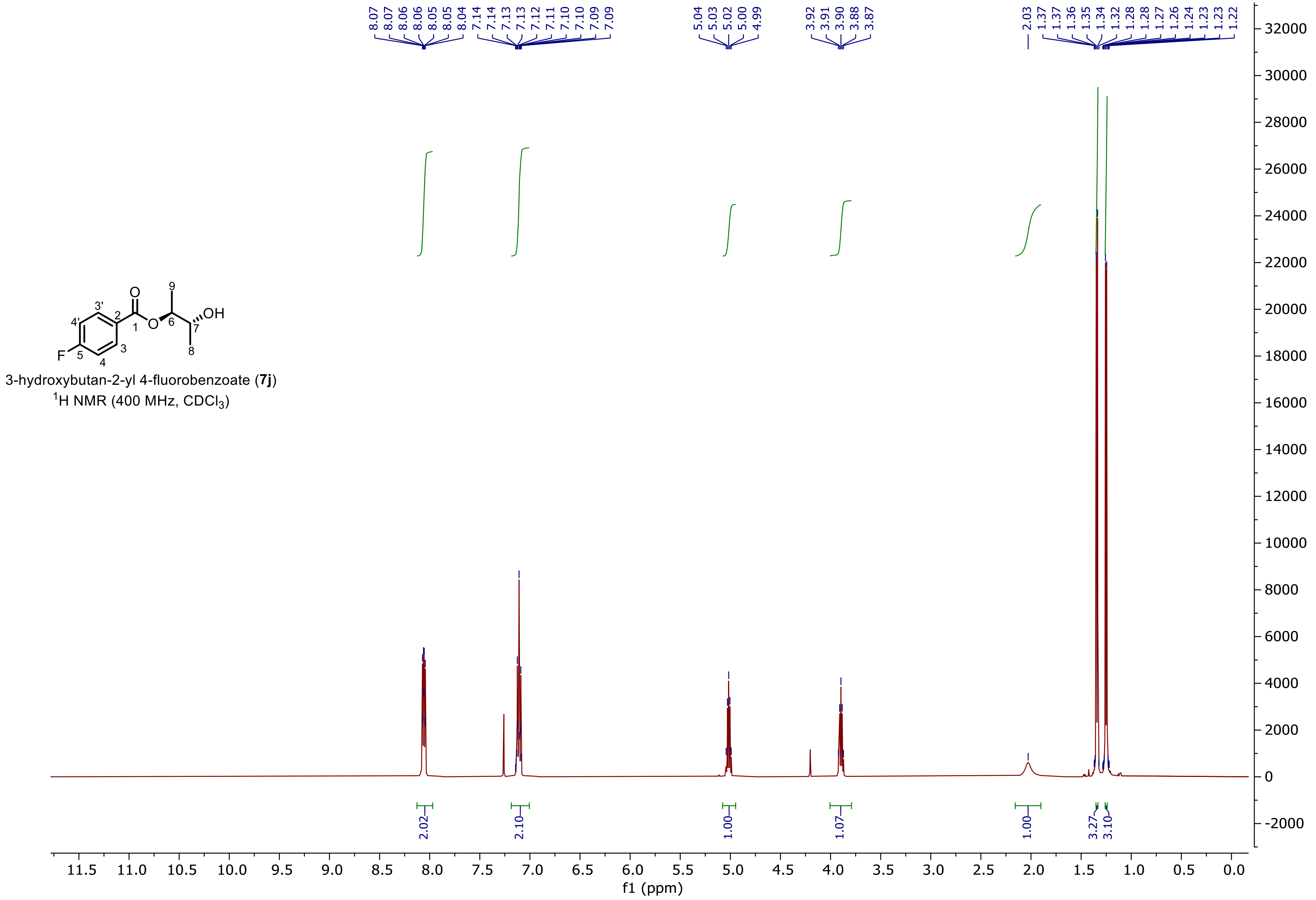

3-hydroxybutan-2-yl 4-fluorobenzoate (**7j**)

^{1}H NMR (400 MHz, $CDCl_3$)

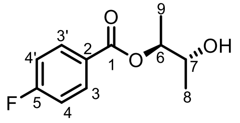

3-hydroxybutan-2-yl 4-fluorobenzoate (**7j**)
^{13}C NMR (101 MHz, $CDCl_3$)

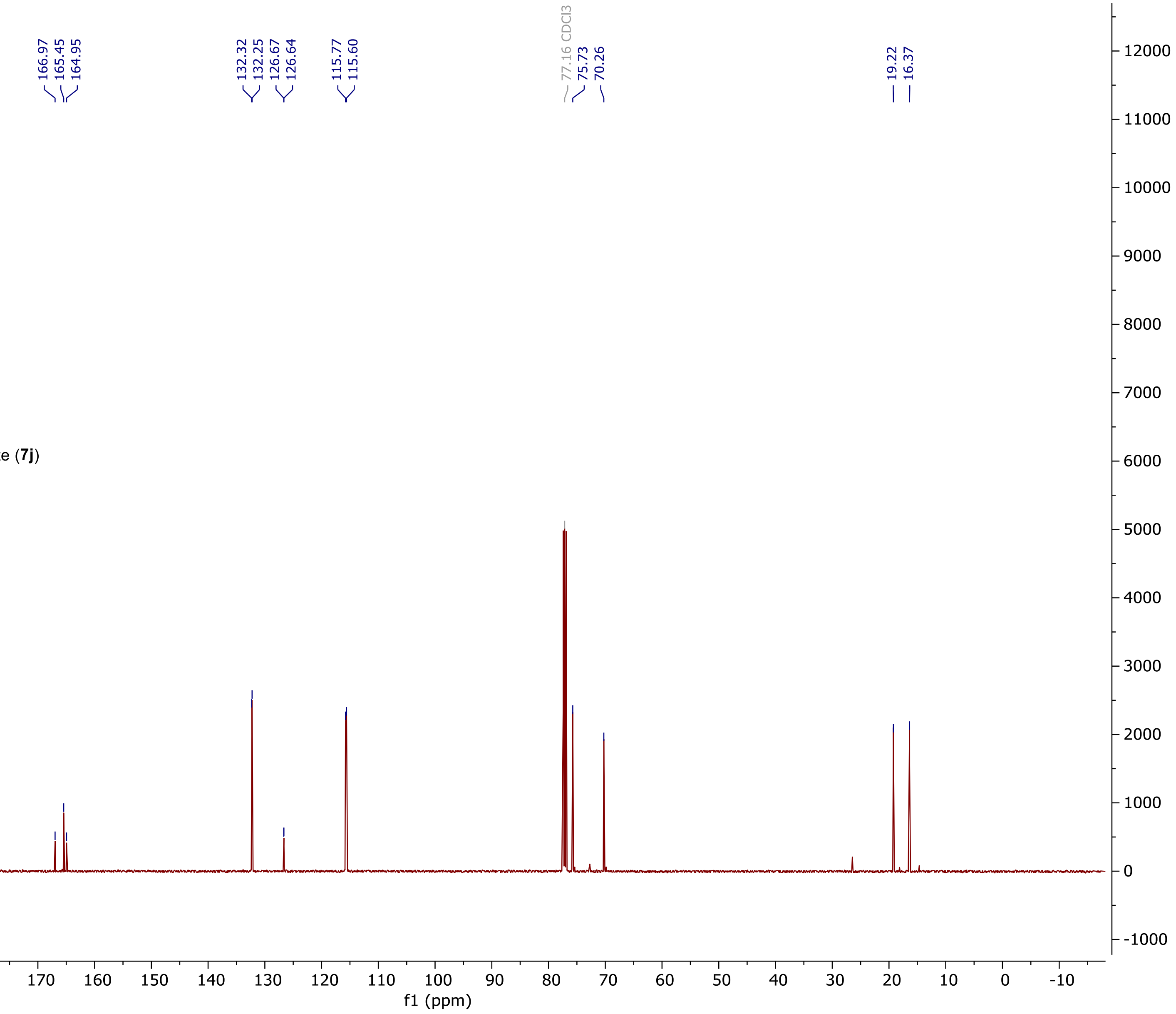

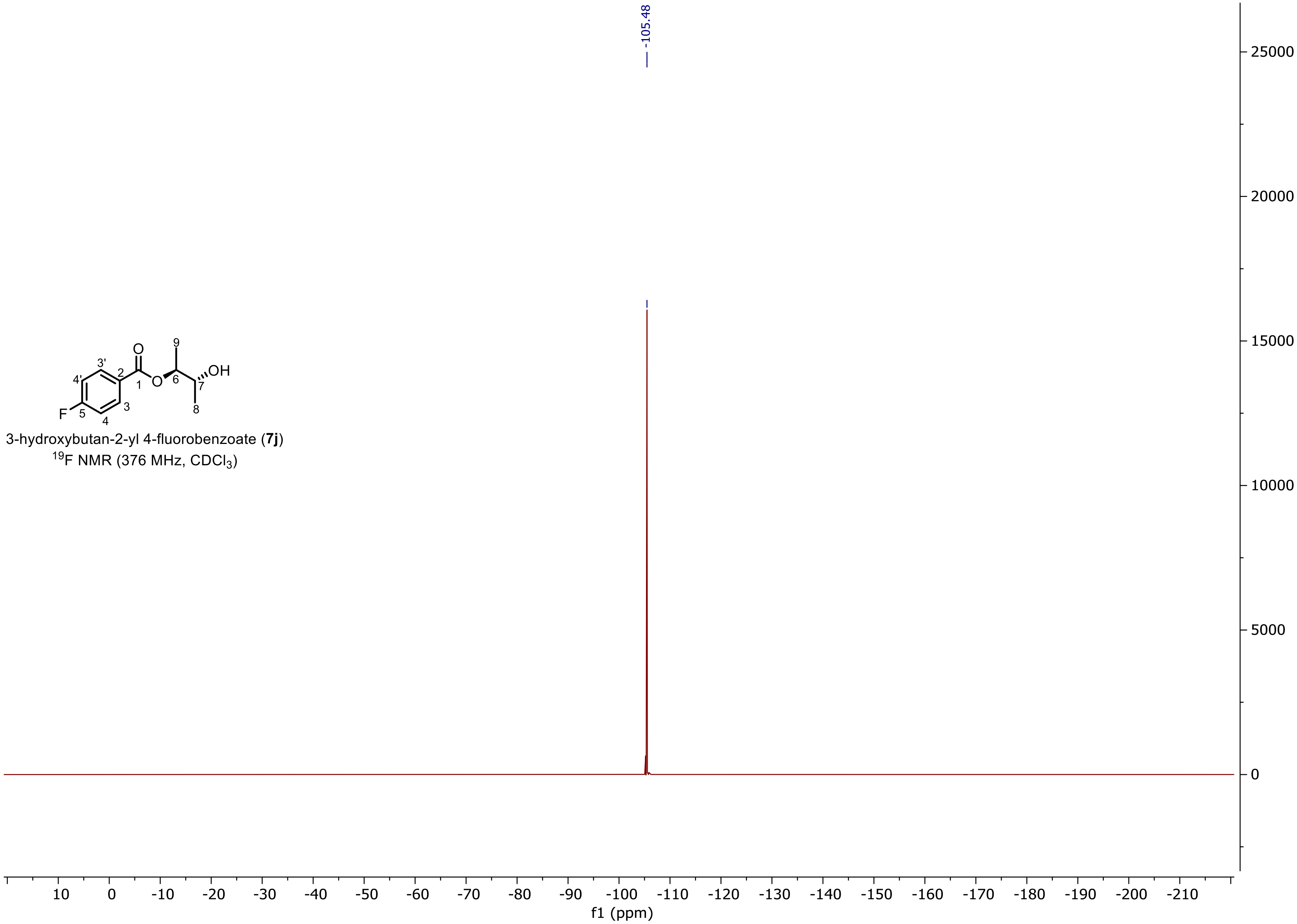

3-hydroxybutan-2-yl 4-fluorobenzoate (**7j**)

^{19}F NMR (376 MHz, $CDCl_3$)

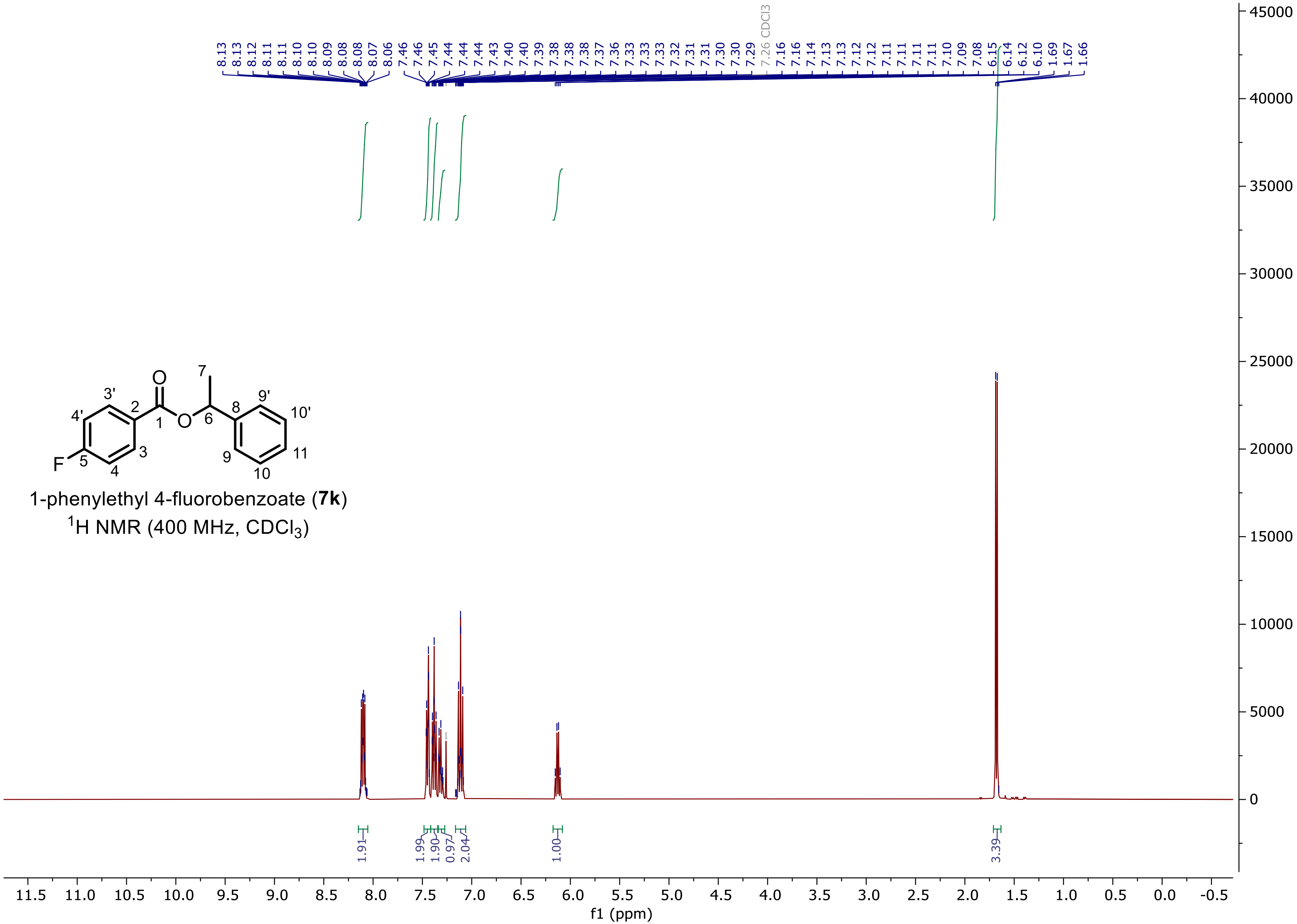

1-phenylethyl 4-fluorobenzoate (**7k**)

^{1}H NMR (400 MHz, $CDCl_3$)

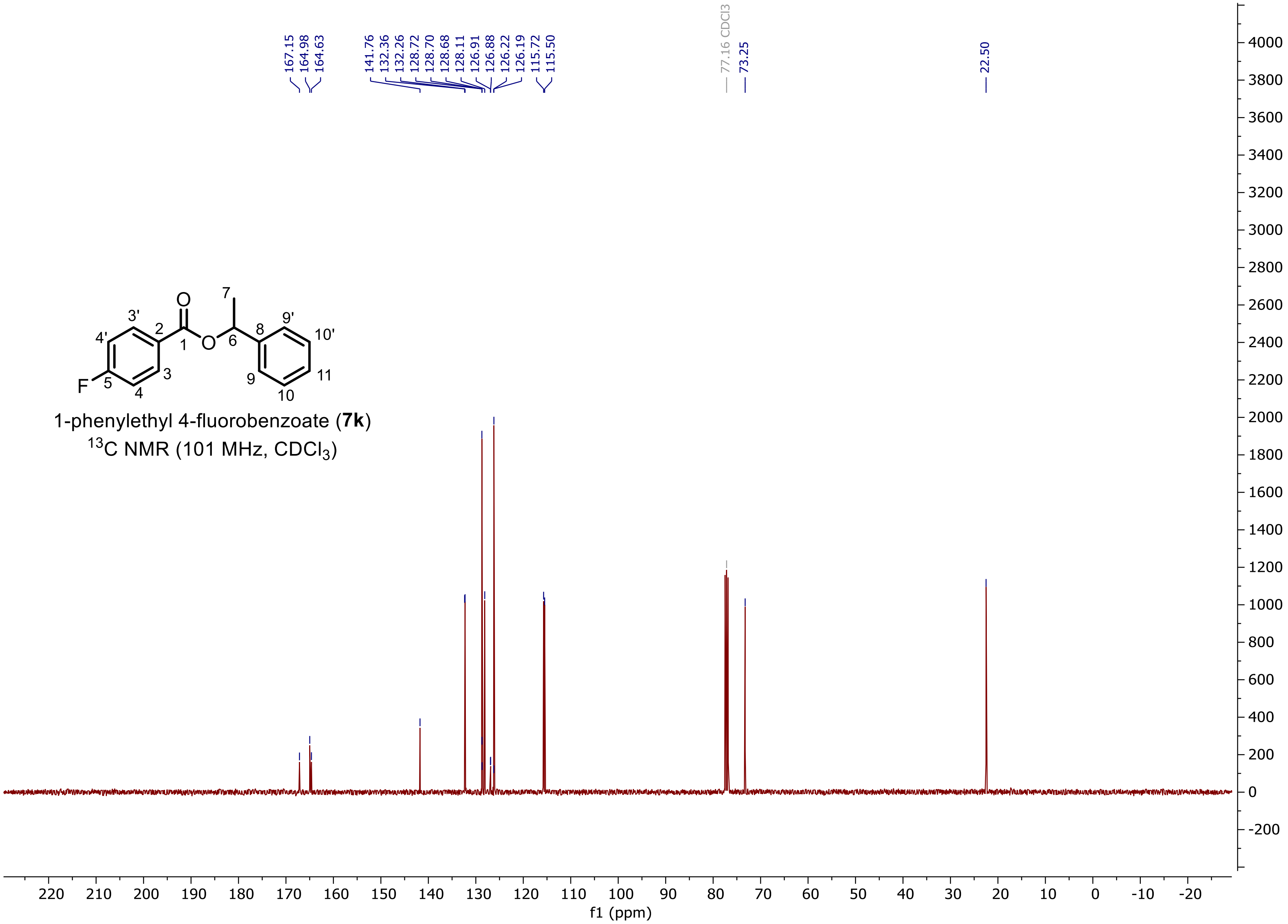

1-phenylethyl 4-fluorobenzoate (**7k**)

^{13}C NMR (101 MHz, $CDCl_3$)

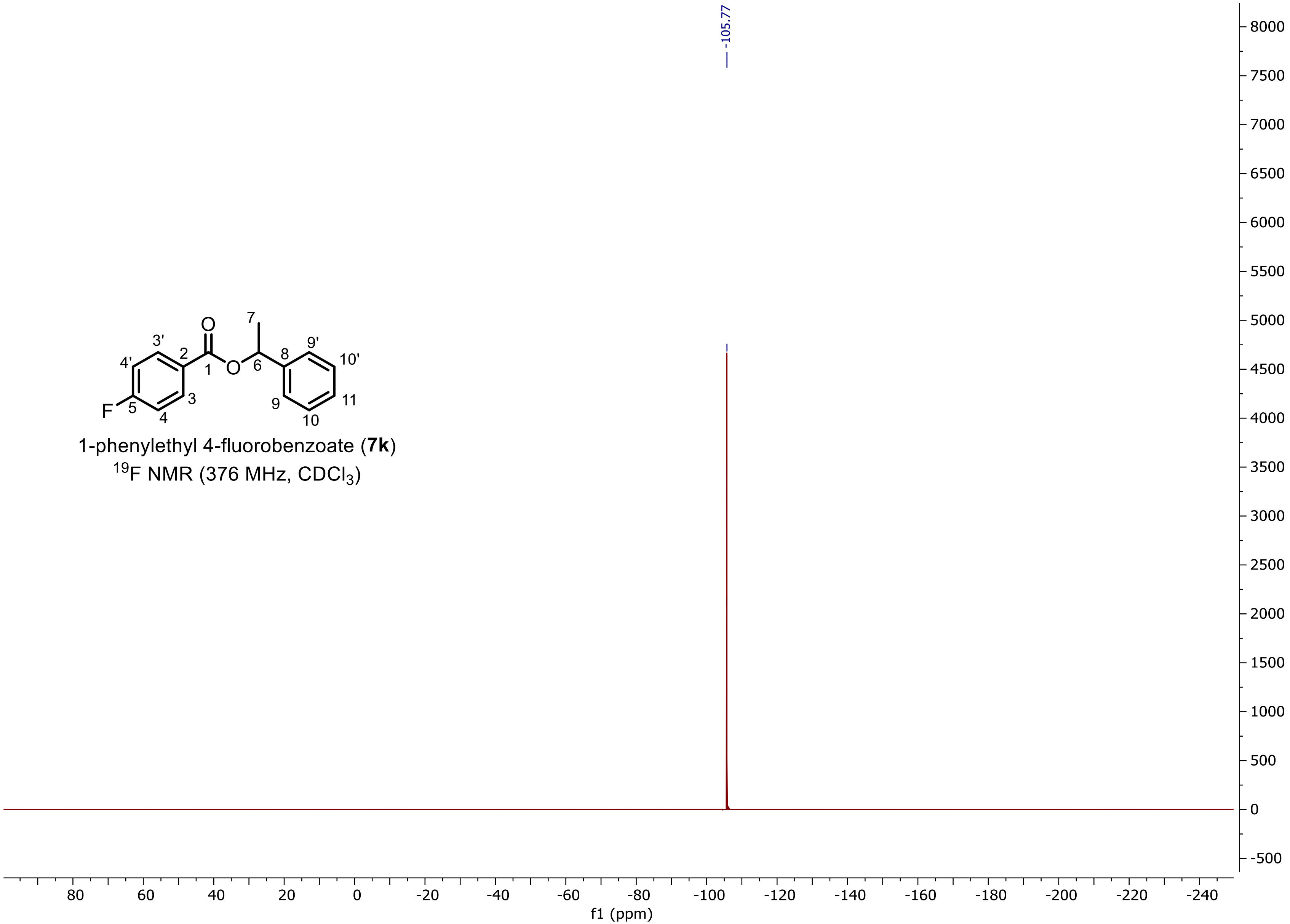

O
7
3'
2
9'
4'
1
O
6
8
10'
F
5
3
9
11
4
10
1-phenylethyl 4-fluorobenzoate (7k)
19F NMR (376 MHz, CDCl3)
-105.77
8000
7500
7000
6500
6000
5500
5000
4500
4000
3500
3000
2500
2000
1500
1000
500
0
-500
80
60
40
20
0
-20
-40
-60
-80
-100
-120
-140
-160
-180
-200
-220
-240
f1 (ppm)

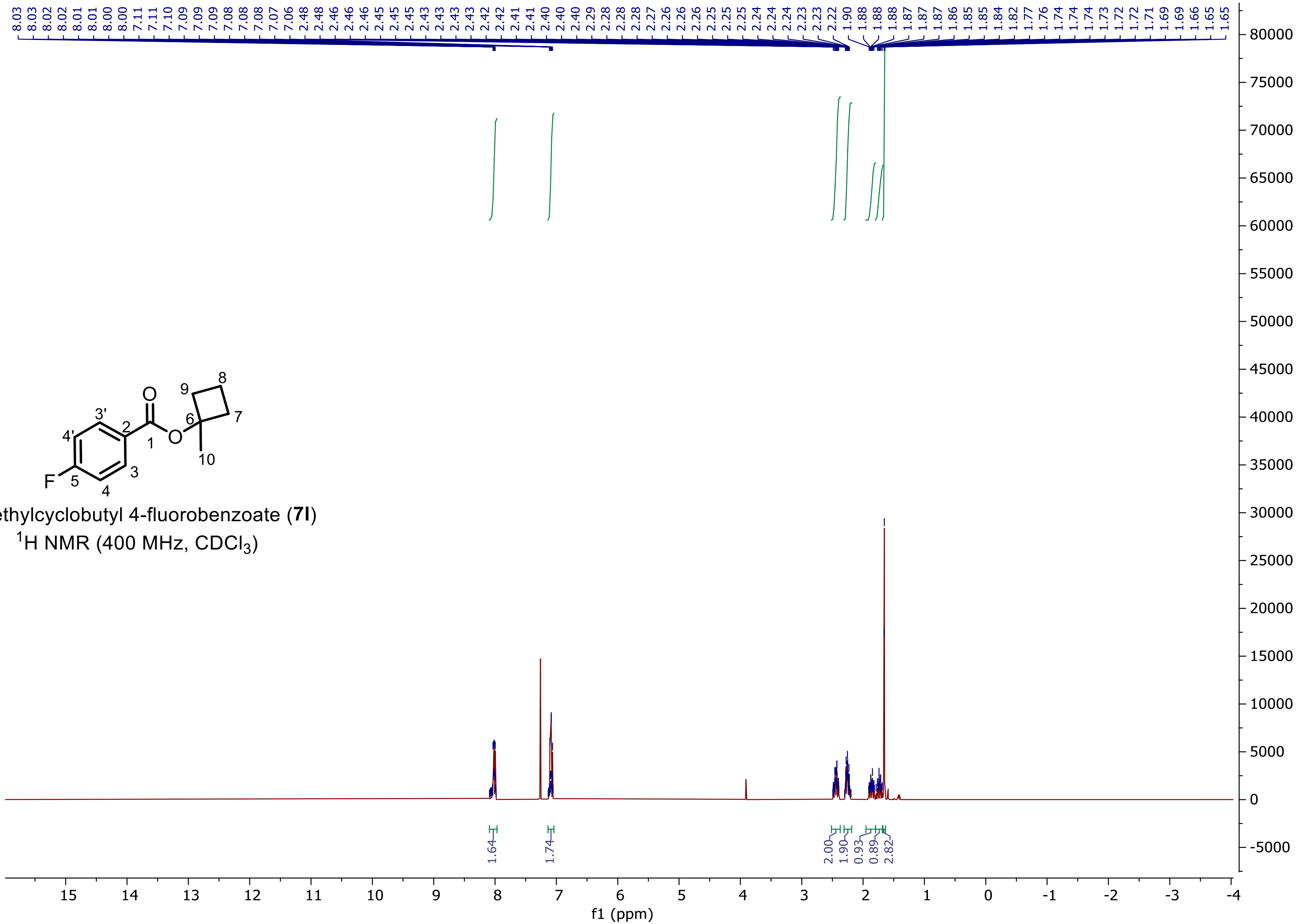

1-methylcyclobutyl 4-fluorobenzoate (**7l**)

^{1}H NMR (400 MHz, $CDCl_3$)

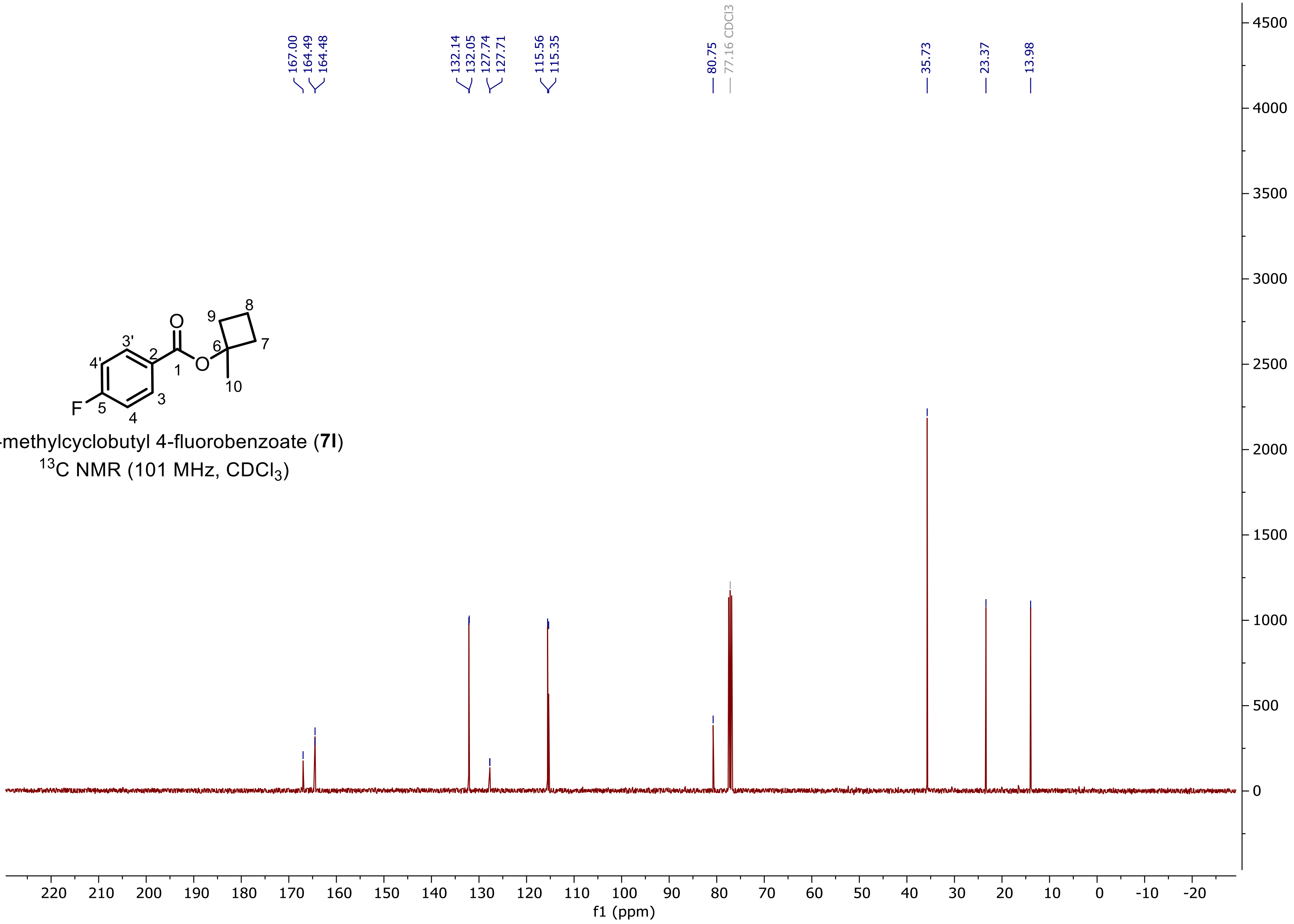

1-methylcyclobutyl 4-fluorobenzoate (**7l**)

^{13}C NMR (101 MHz, $CDCl_3$)

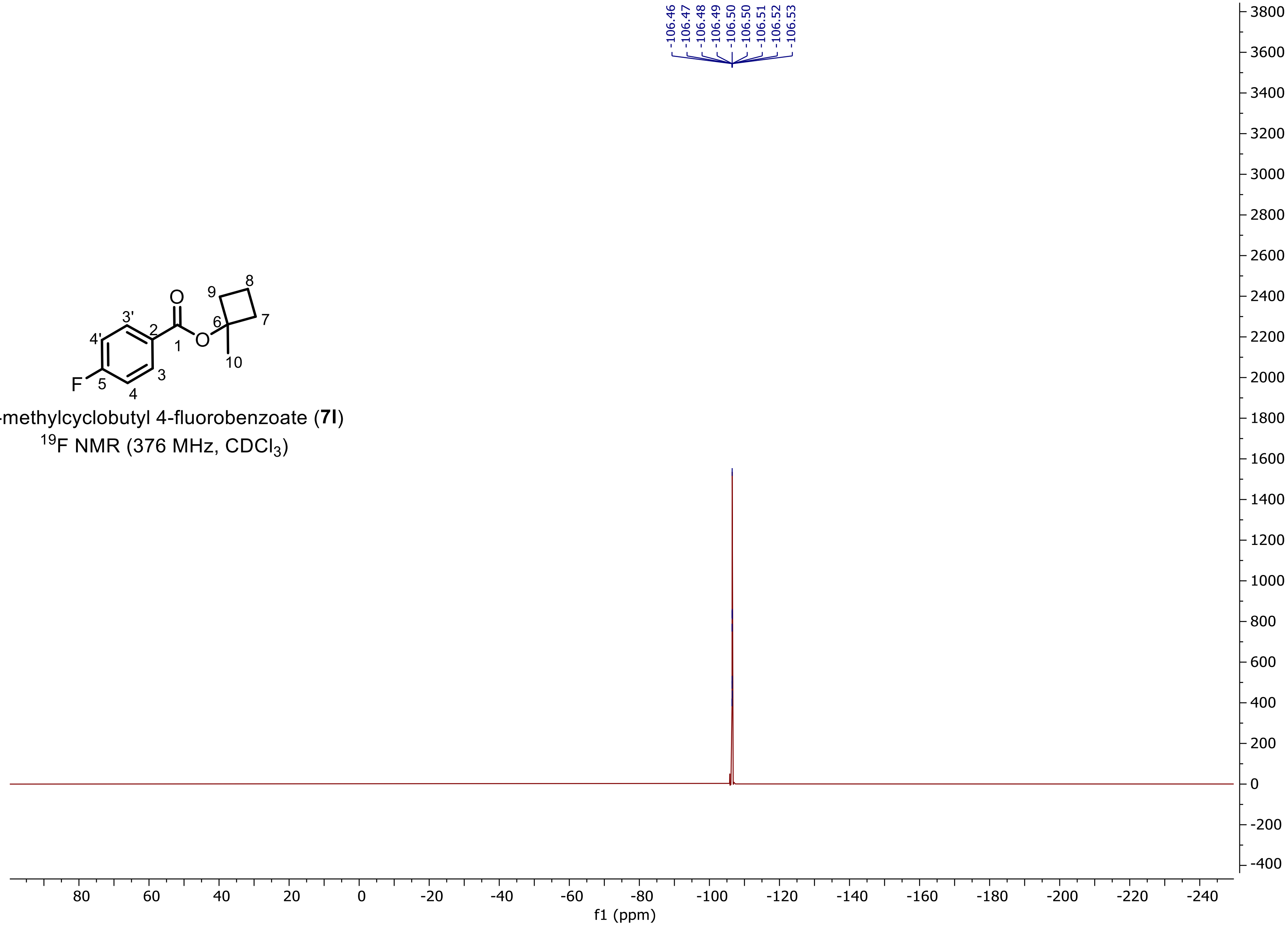

1-methylcyclobutyl 4-fluorobenzoate (**7l**)
^{19}F NMR (376 MHz, $CDCl_3$)

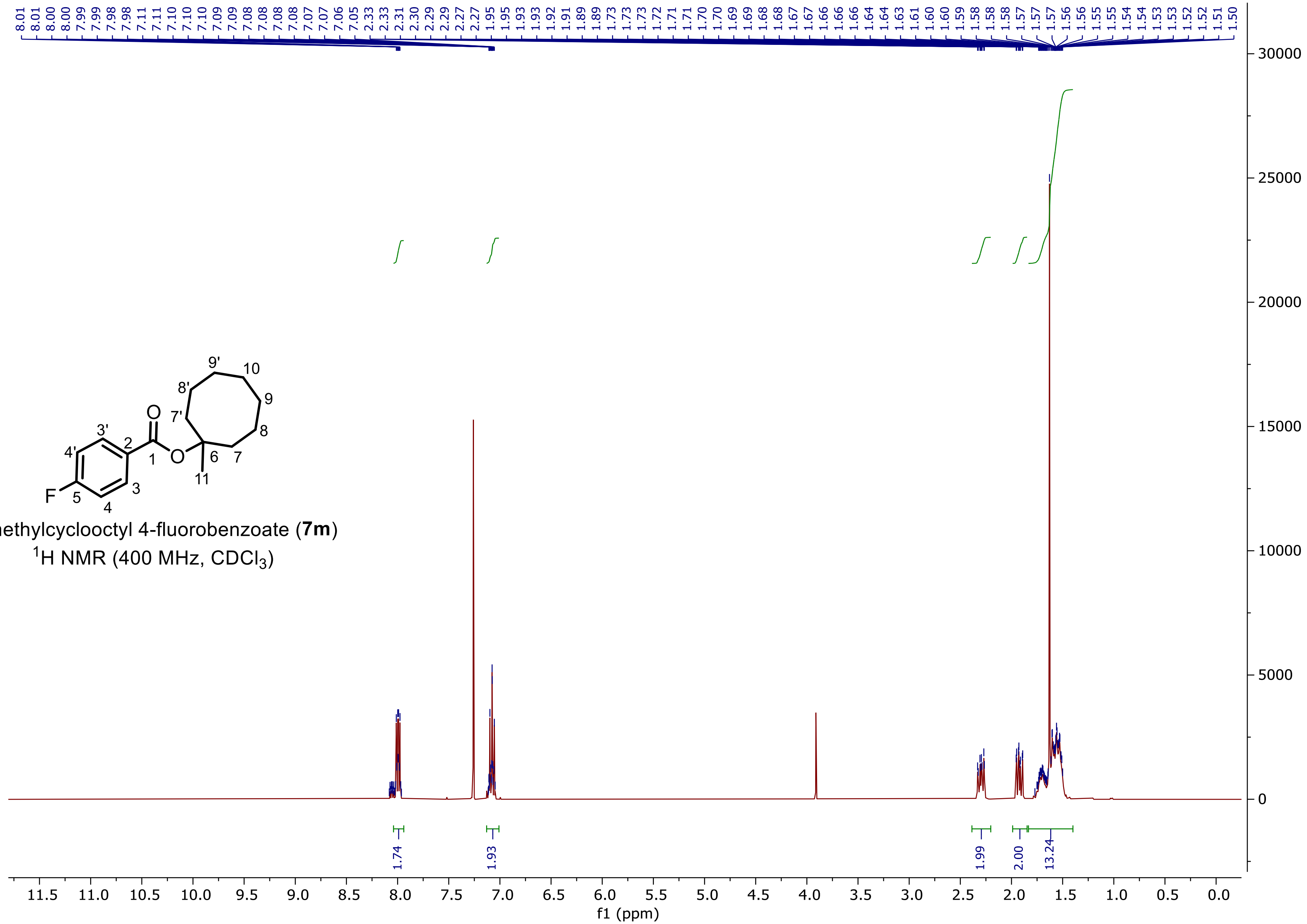

1-methylcyclooctyl 4-fluorobenzoate (**7m**)

^{1}H NMR (400 MHz, $CDCl_3$)

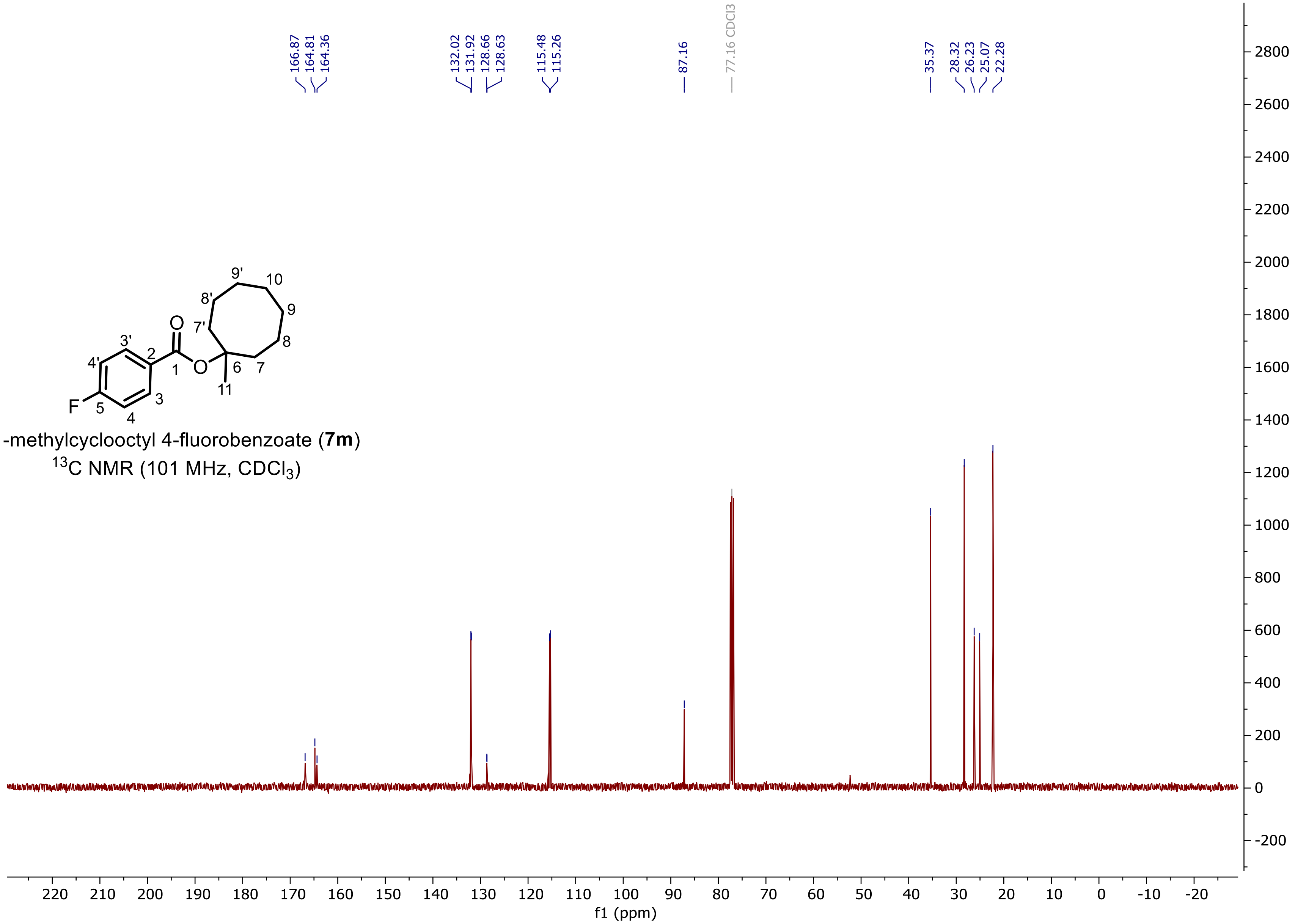

1-methylcyclooctyl 4-fluorobenzoate (**7m**)

^{13}C NMR (101 MHz, $CDCl_3$)

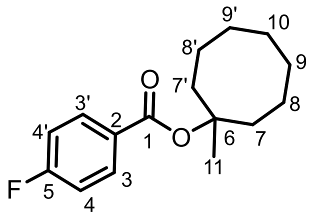

1-methylcyclooctyl 4-fluorobenzoate (**7m**)

^{19}F NMR (376 MHz, $CDCl_3$)

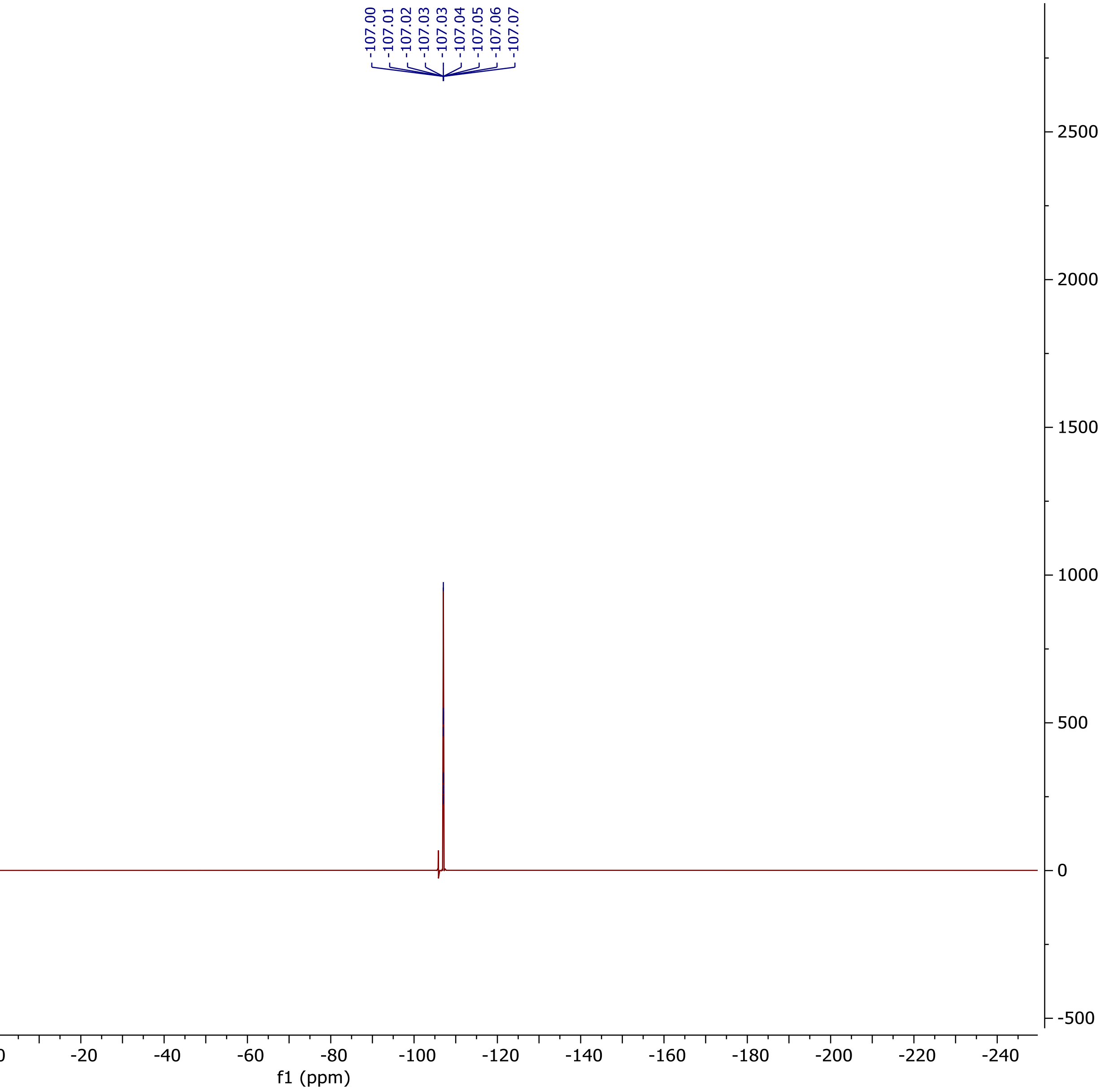

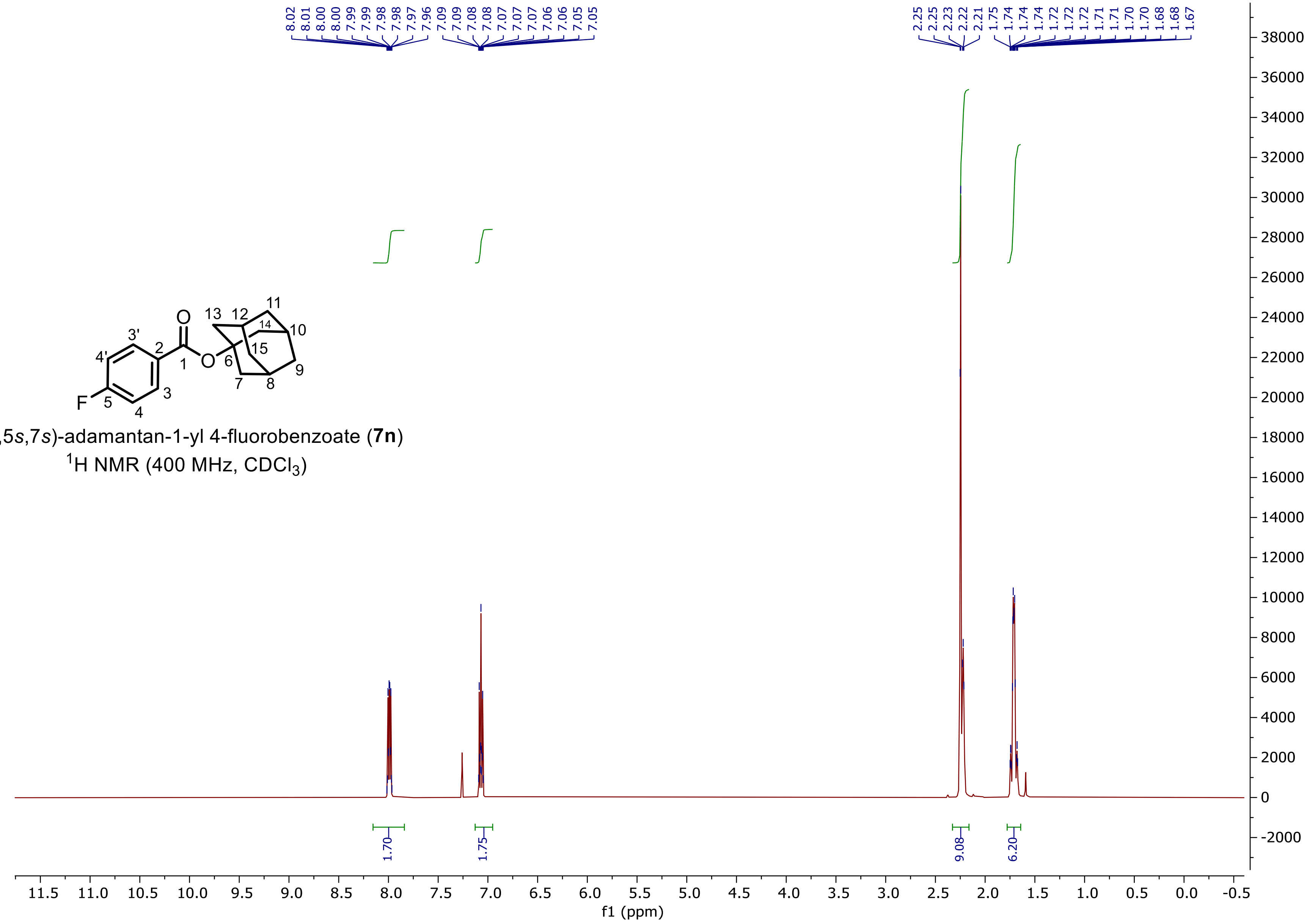

(3*s*,5*s*,7*s*)-adamantan-1-yl 4-fluorobenzoate (**7n**)

^{1}H NMR (400 MHz, $CDCl_3$)

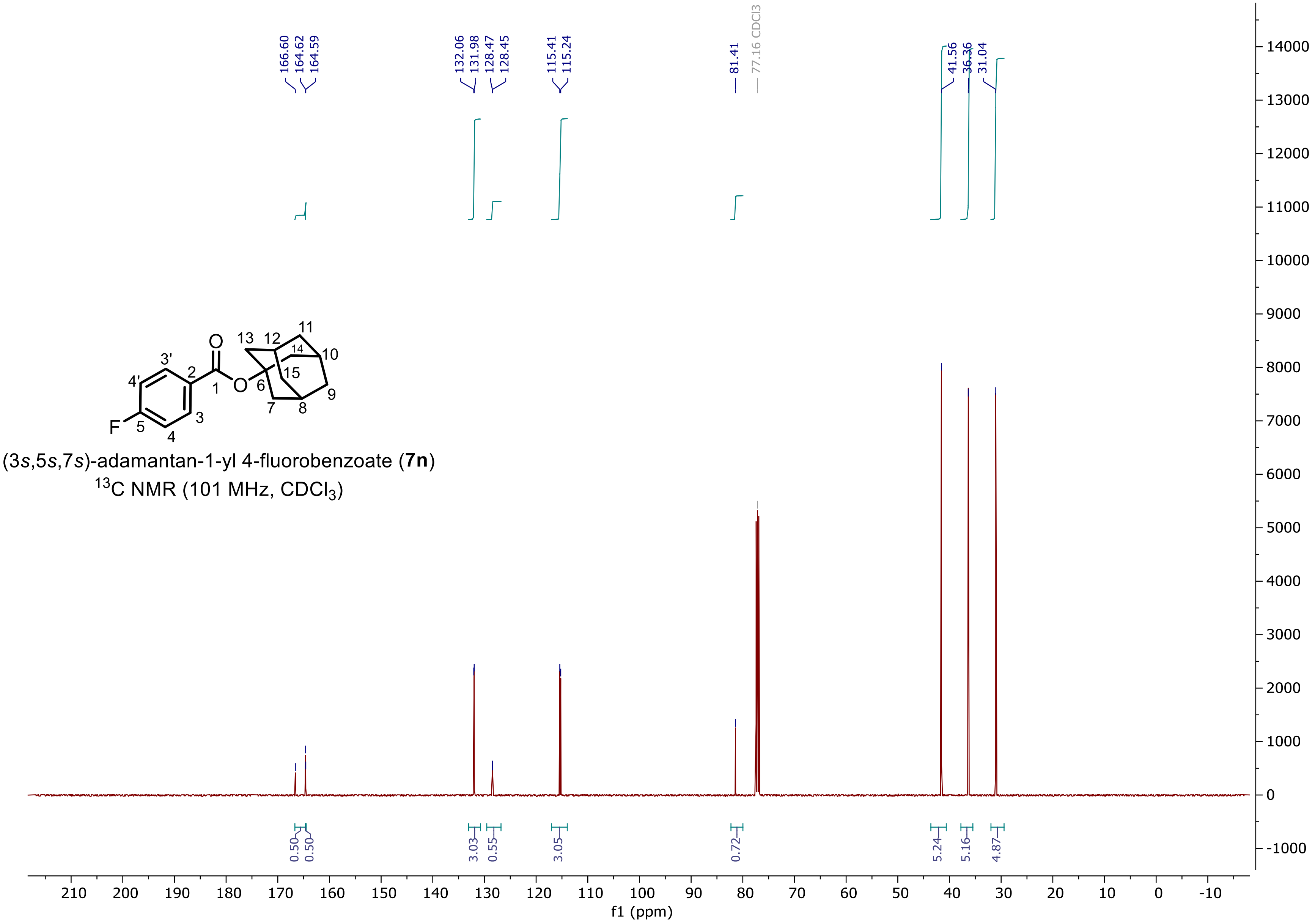

(3*s*,5*s*,7*s*)-adamantan-1-yl 4-fluorobenzoate (**7n**)
^{13}C NMR (101 MHz, $CDCl_3$)

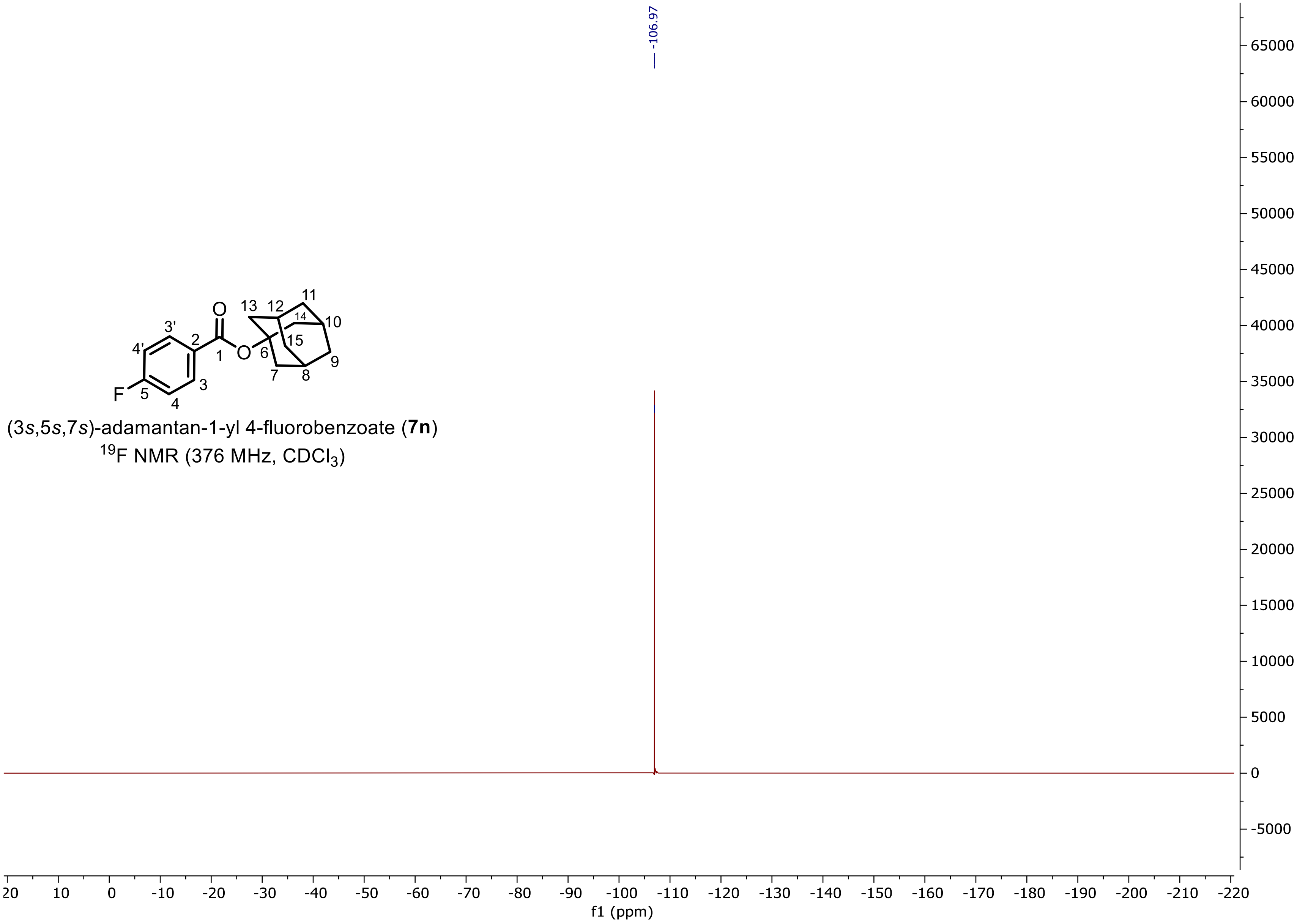

(3*s*,5*s*,7*s*)-adamantan-1-yl 4-fluorobenzoate (**7n**)
^{19}F NMR (376 MHz, $CDCl_3$)

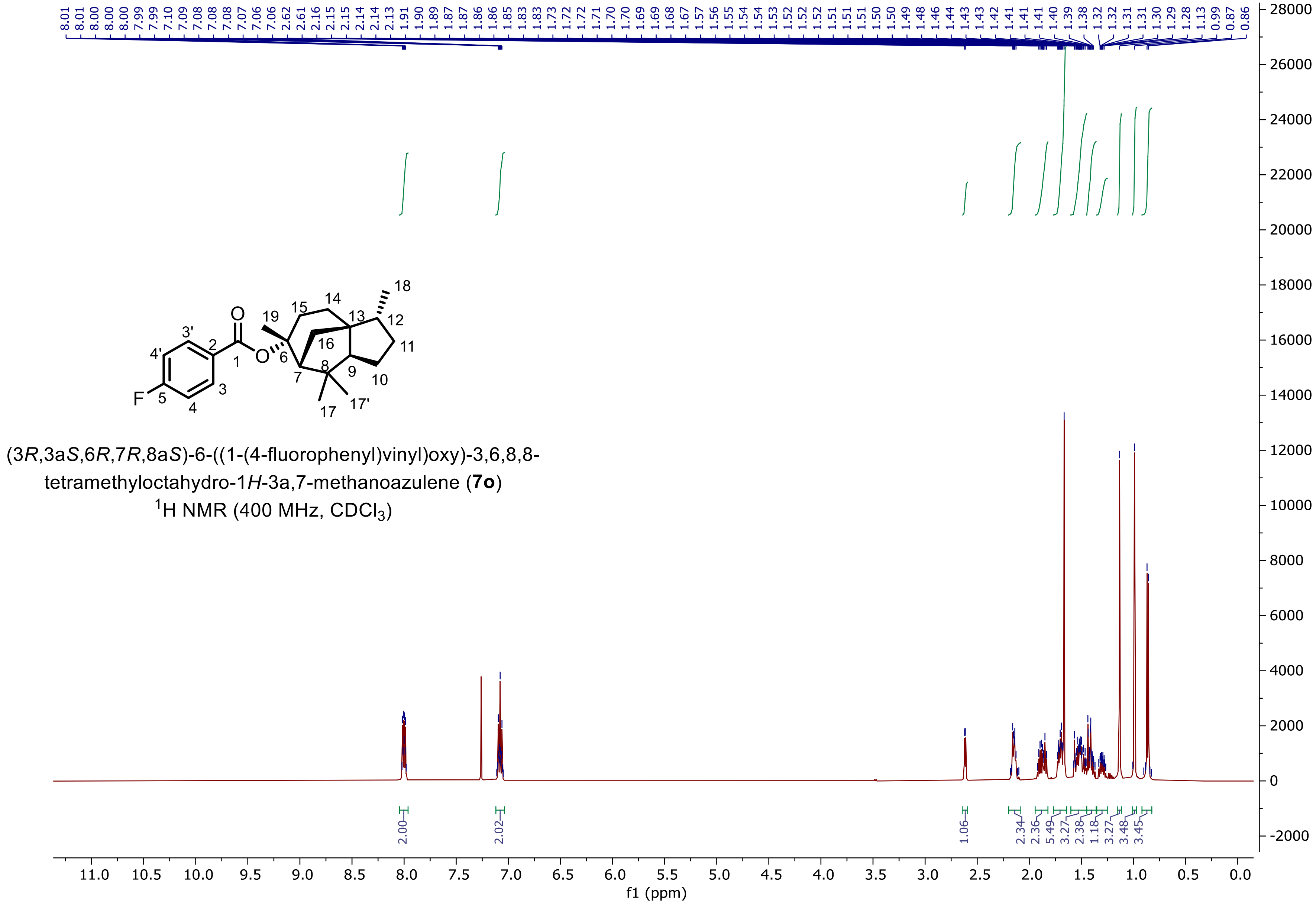

(3*R*,3a*S*,6*R*,7*R*,8a*S*)-6-((1-(4-fluorophenyl)vinyl)oxy)-3,6,8,8-tetramethyloctahydro-1*H*-3a,7-methanoazulene (**7o**)

^{1}H NMR (400 MHz, $CDCl_3$)

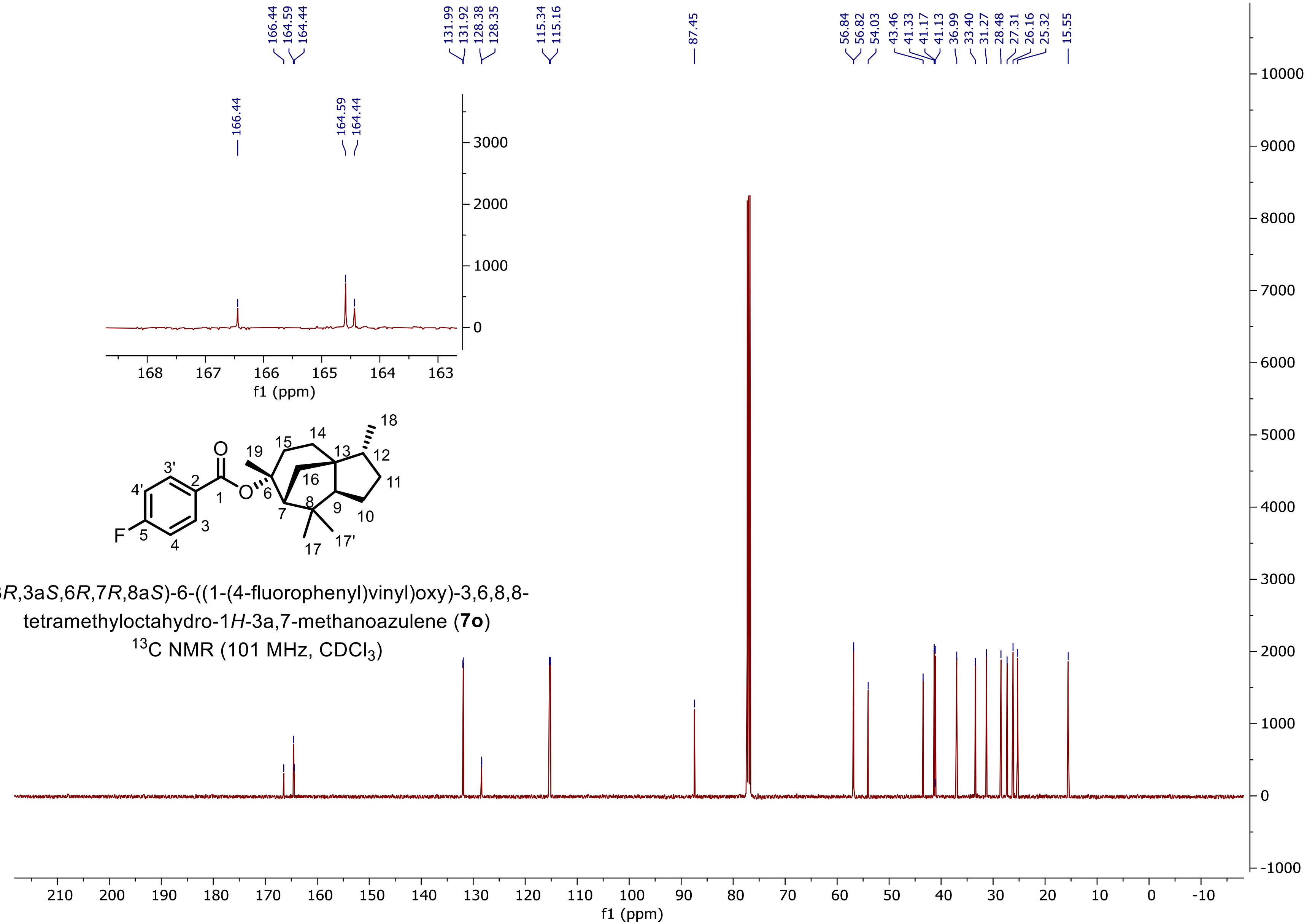

(3*R*,3a*S*,6*R*,7*R*,8a*S*)-6-((1-(4-fluorophenyl)vinyl)oxy)-3,6,8,8-tetramethyloctahydro-1*H*-3a,7-methanoazulene (**7o**)

^{13}C NMR (101 MHz, $CDCl_3$)

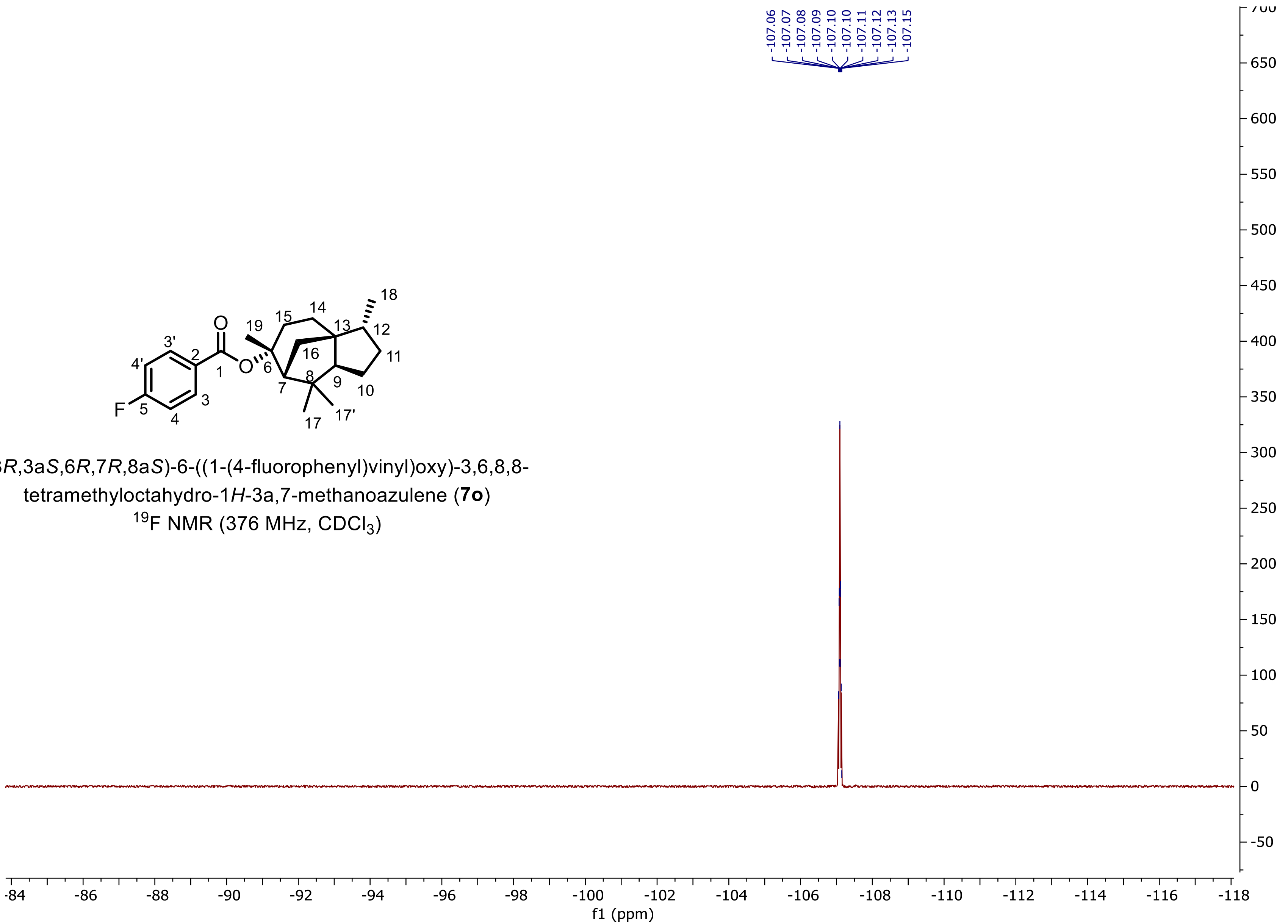

(3*R*,3a*S*,6*R*,7*R*,8a*S*)-6-((1-(4-fluorophenyl)vinyl)oxy)-3,6,8,8-tetramethyloctahydro-1*H*-3a,7-methanoazulene (**7o**)

^{19}F NMR (376 MHz, $CDCl_3$)

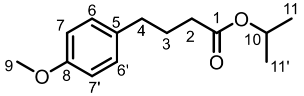

isopropyl 4-(4-methoxyphenyl)butanoate (**7v**)
^{1}H NMR (400 MHz, $CDCl_3$)

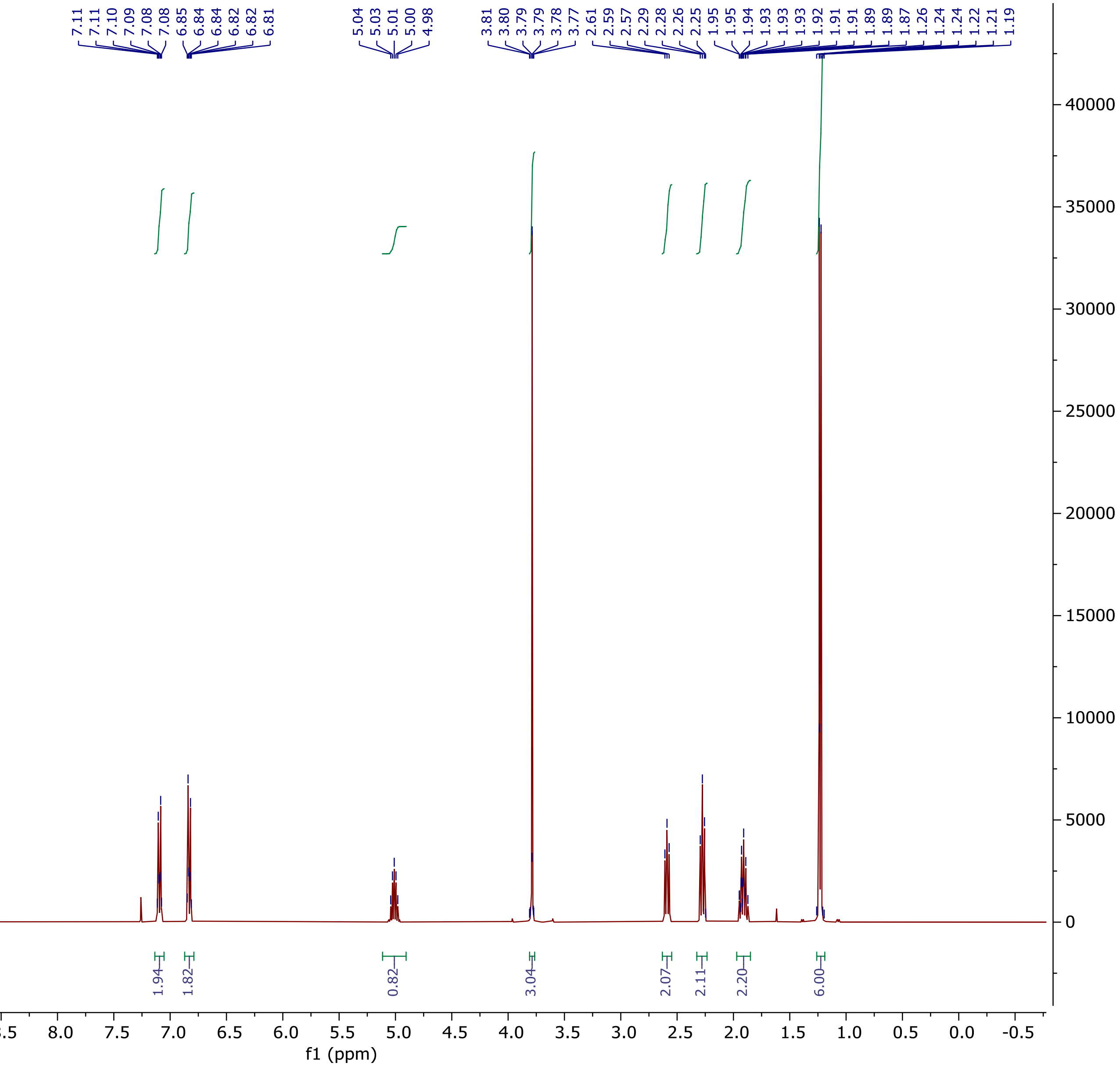

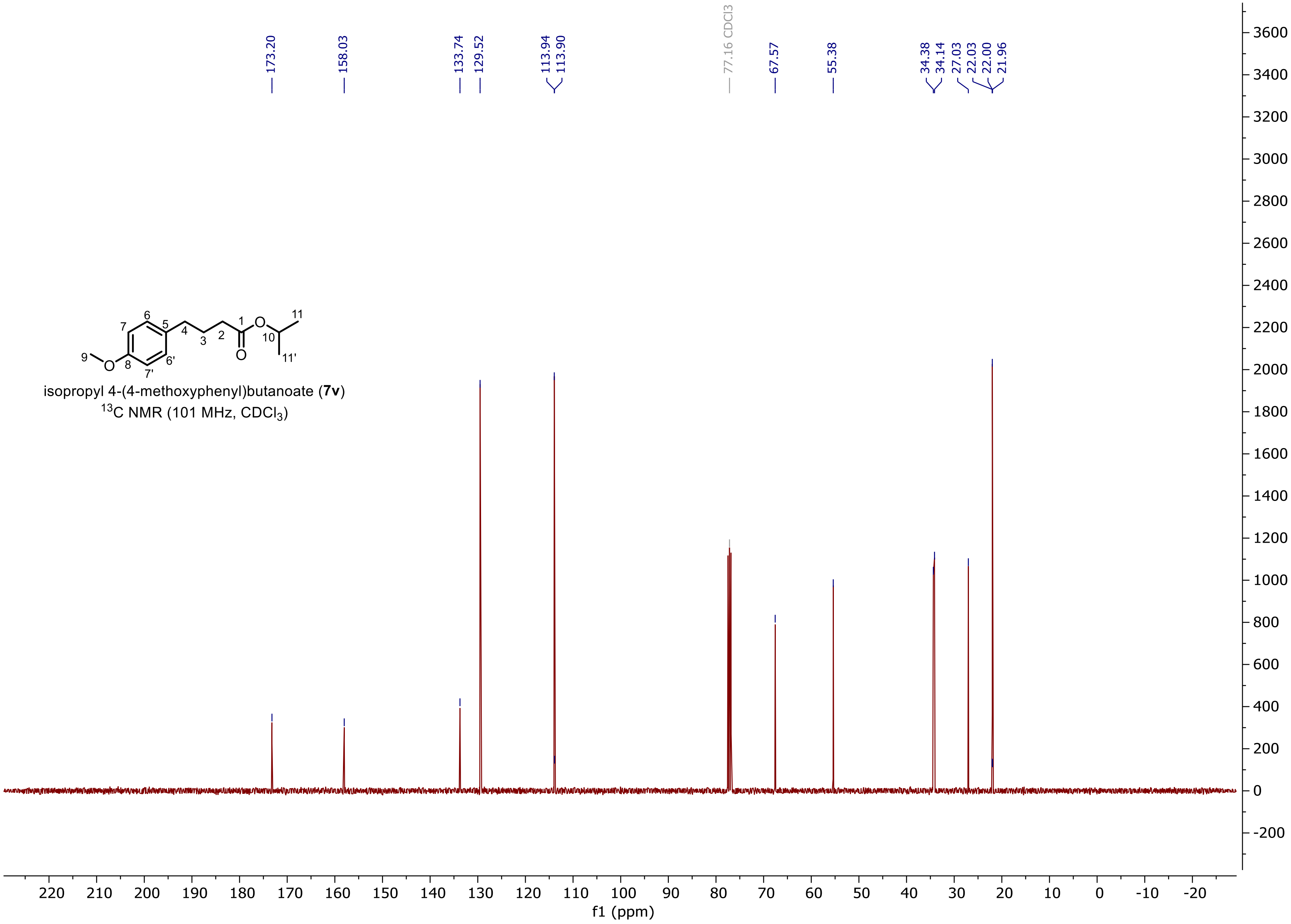
173.20
158.03
133.74
129.52
113.94
113.90
77.16 CDCl3
67.57
55.38
34.38
34.14
27.03
22.03
22.00
21.96
isopropyl 4-(4-methoxyphenyl)butanoate (7v)
13C NMR (101 MHz, CDCl3)
f1 (ppm)

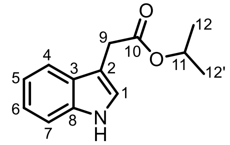

isopropyl 2-(1*H*-indol-3-yl)acetate (**7w**)
^{1}H NMR (400 MHz, $CDCl_3$)

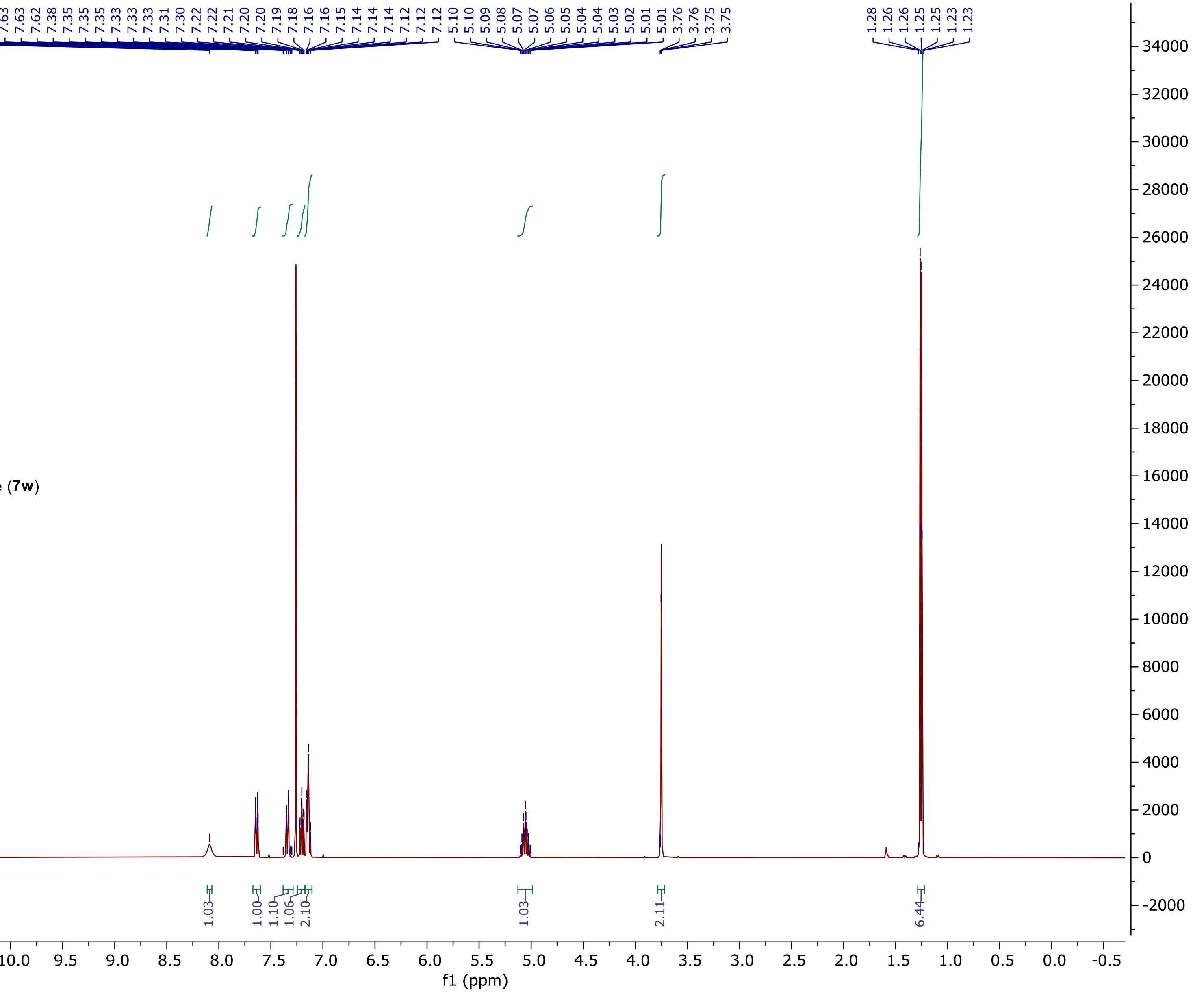

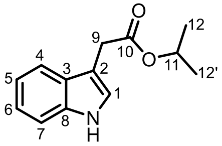

isopropyl 2-(1*H*-indol-3-yl)acetate (**7w**)
^{13}C NMR (101 MHz, $CDCl_3$)

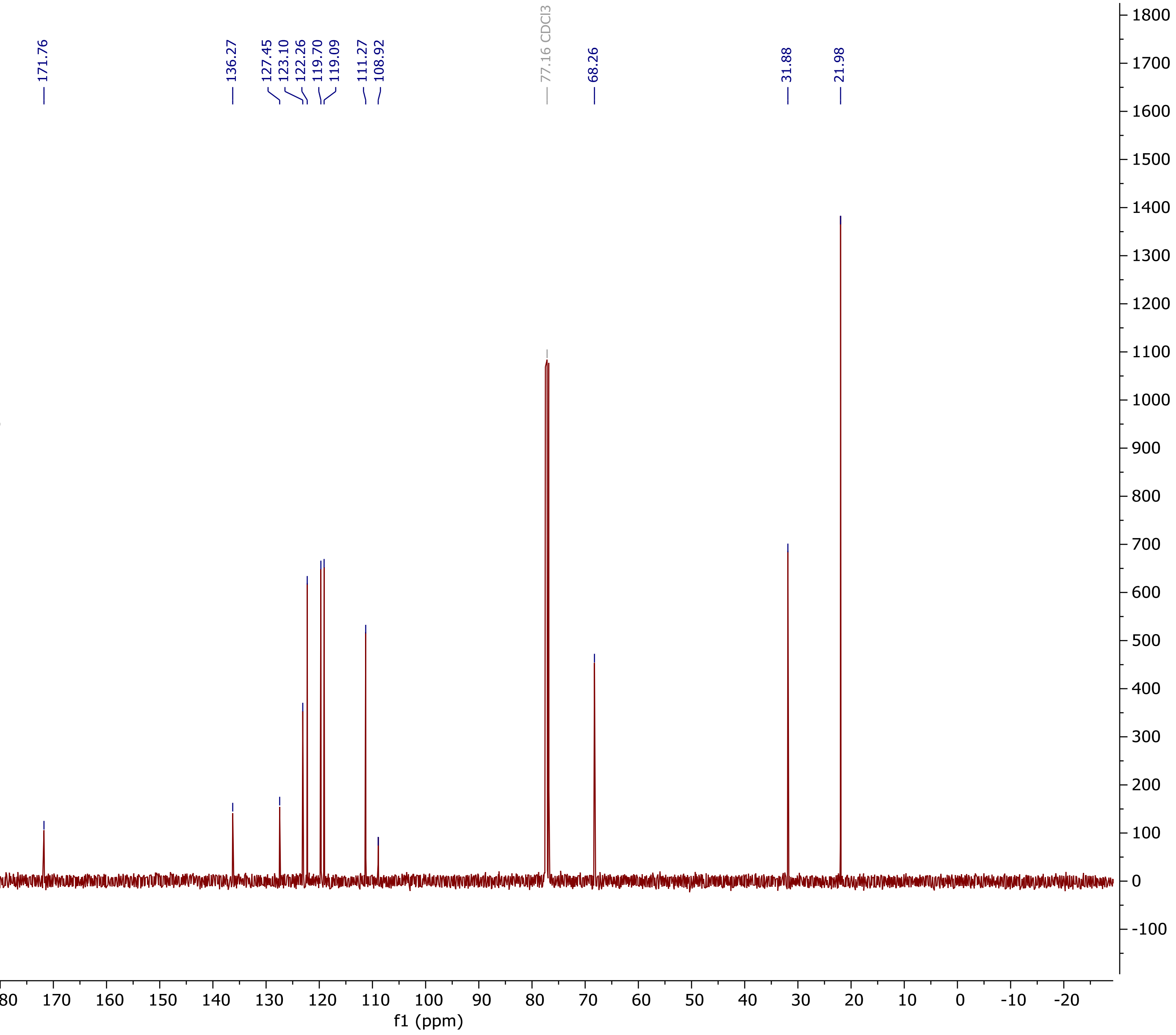

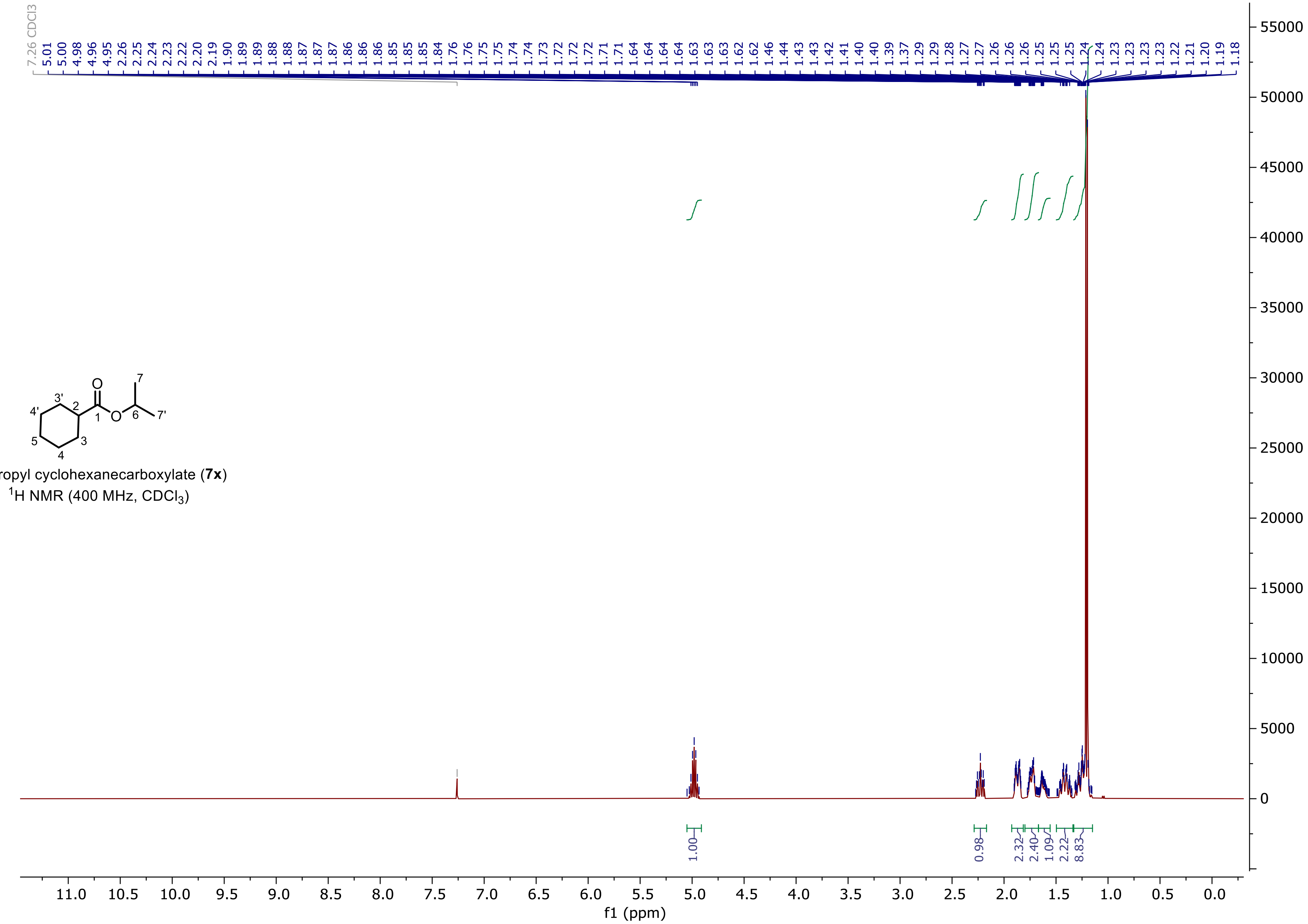

isopropyl cyclohexanecarboxylate (**7x**)

^{1}H NMR (400 MHz, $CDCl_3$)

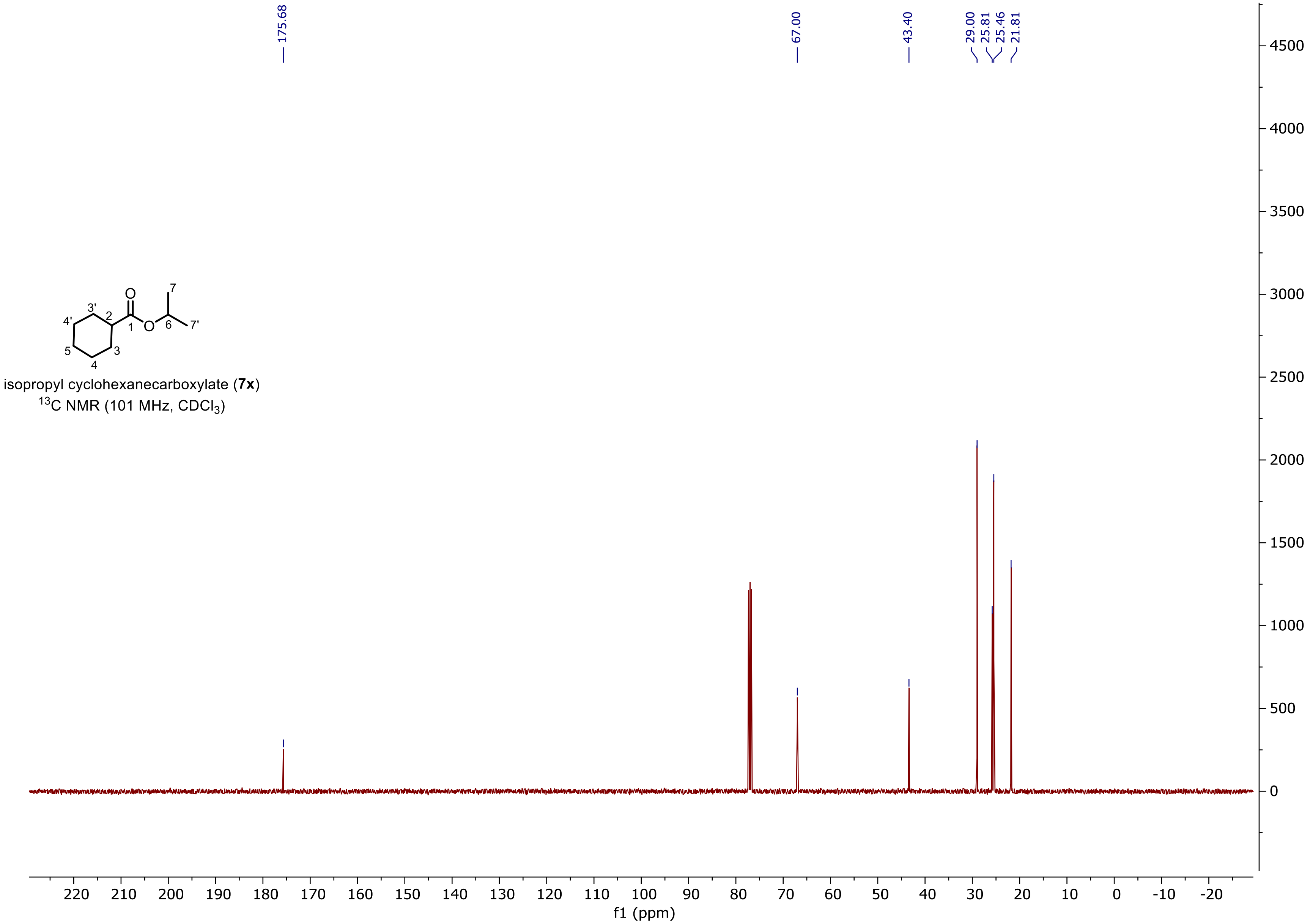

isopropyl cyclohexanecarboxylate (**7x**)
^{13}C NMR (101 MHz, $CDCl_3$)

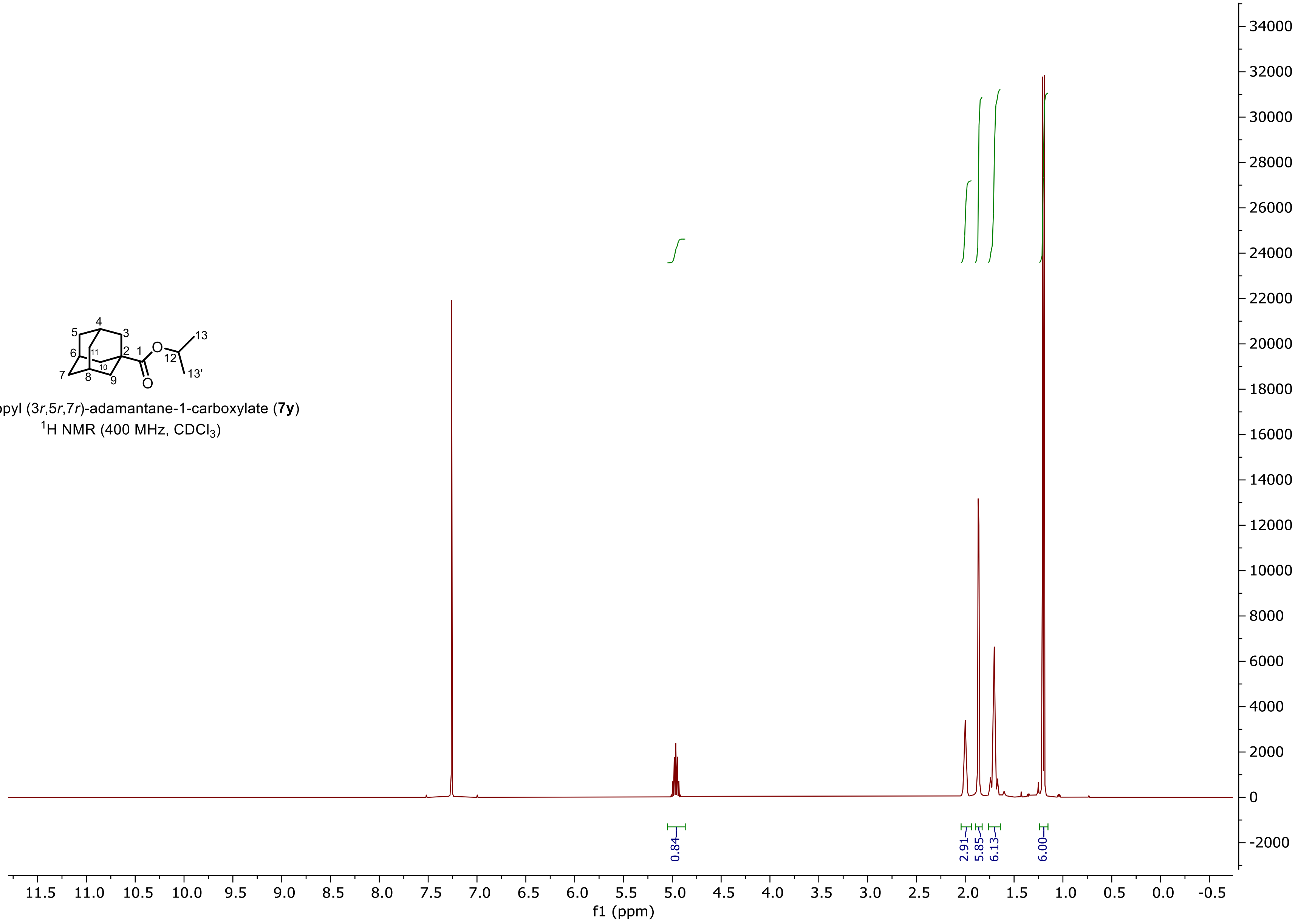

isopropyl (3*r*,5*r*,7*r*)-adamantane-1-carboxylate (**7y**)

^{1}H NMR (400 MHz, $CDCl_3$)

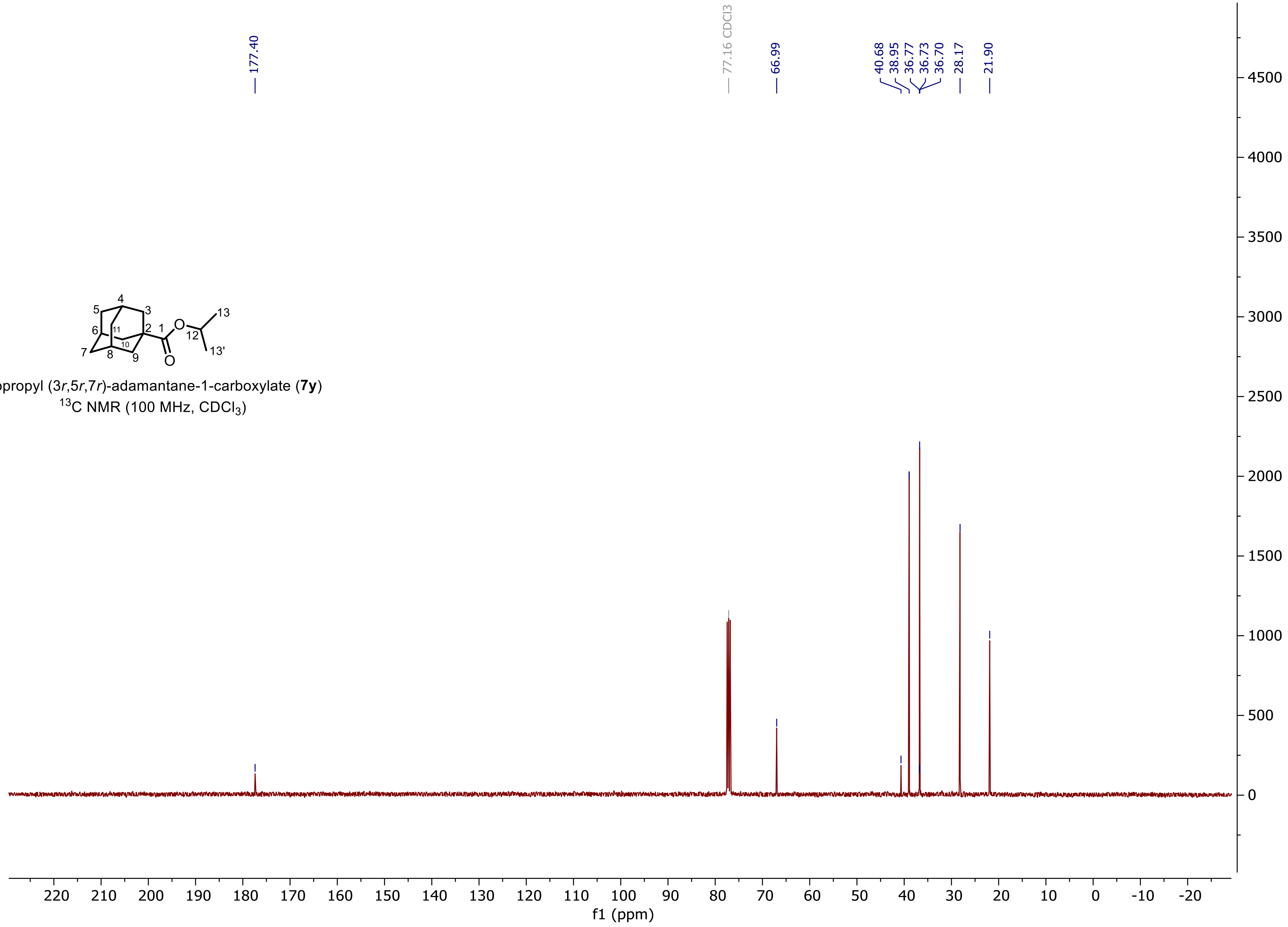

isopropyl (3*r*,5*r*,7*r*)-adamantane-1-carboxylate (**7y**)

^{13}C NMR (100 MHz, $CDCl_3$)

isopropyl 1-phenylcyclopropane-1-carboxylate (**7z**)
^{1}H NMR (400 MHz, $CDCl_3$)

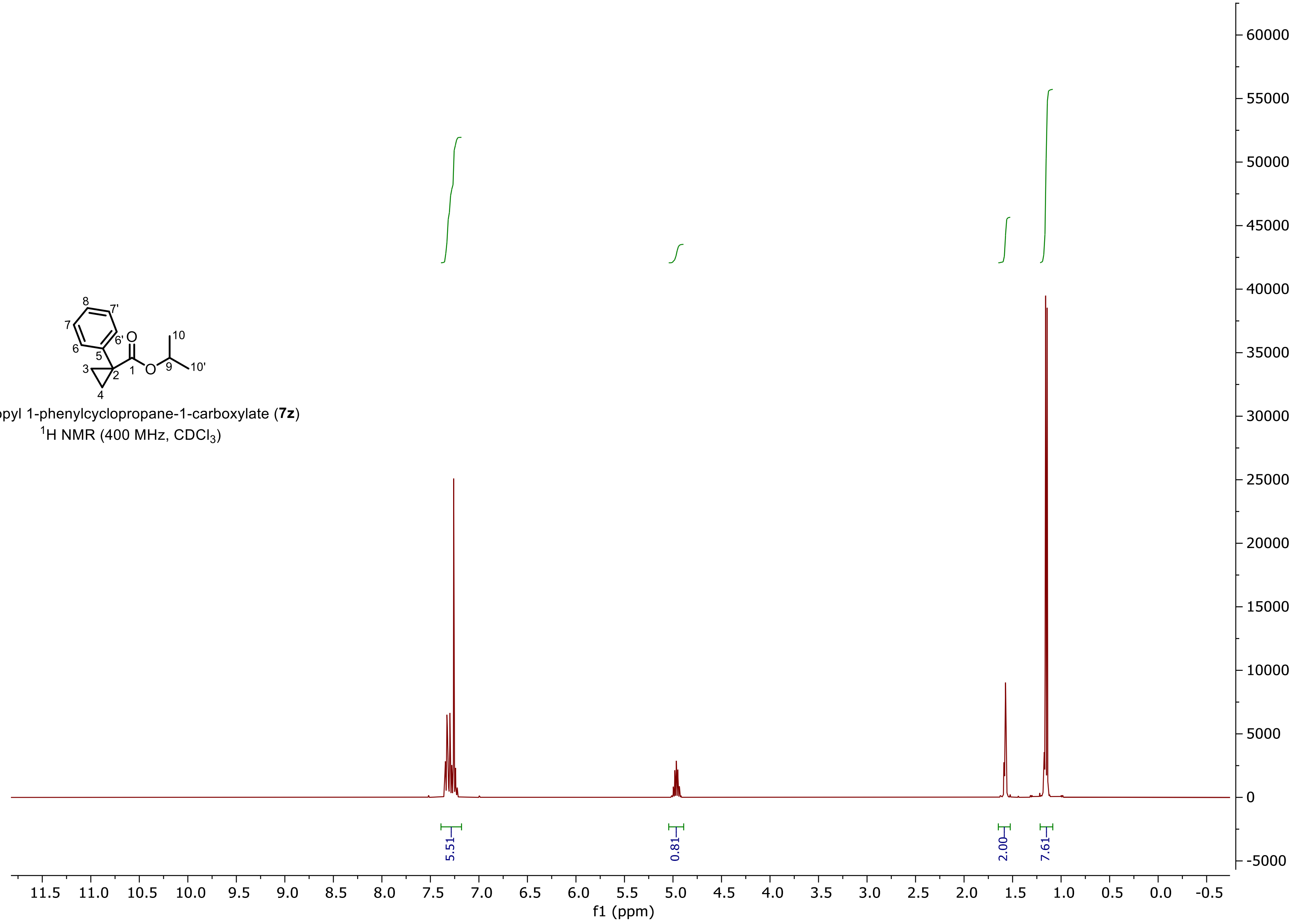

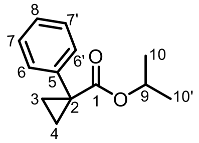

isopropyl 1-phenylcyclopropane-1-carboxylate (**7z**)
^{13}C NMR (101 MHz, $CDCl_3$)

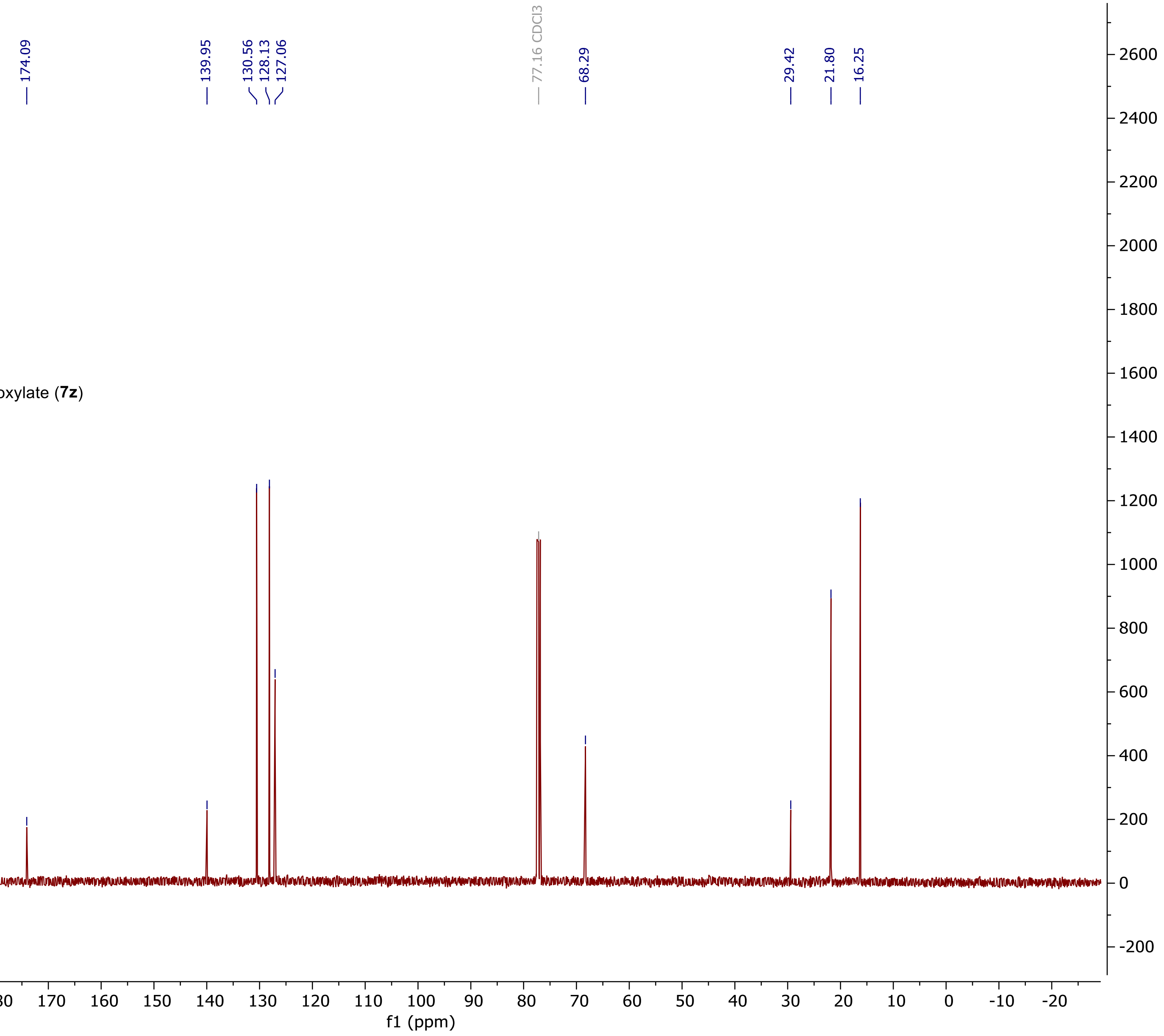

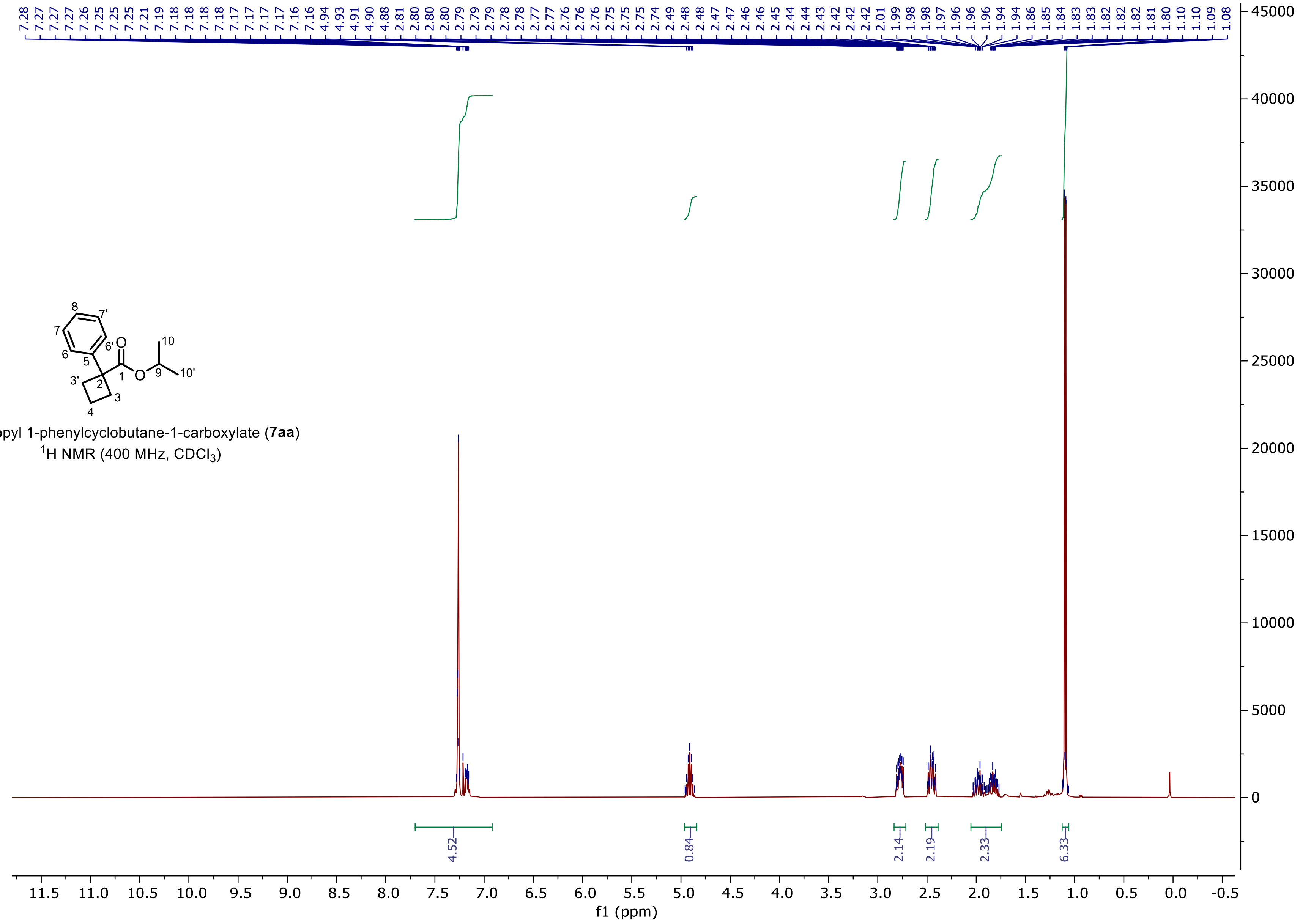

isopropyl 1-phenylcyclobutane-1-carboxylate (**7aa**)
^{1}H NMR (400 MHz, $CDCl_3$)

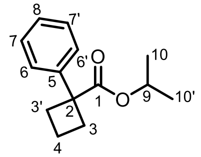

isopropyl 1-phenylcyclobutane-1-carboxylate (**7aa**)
^{13}C NMR (101 MHz, $CDCl_3$)

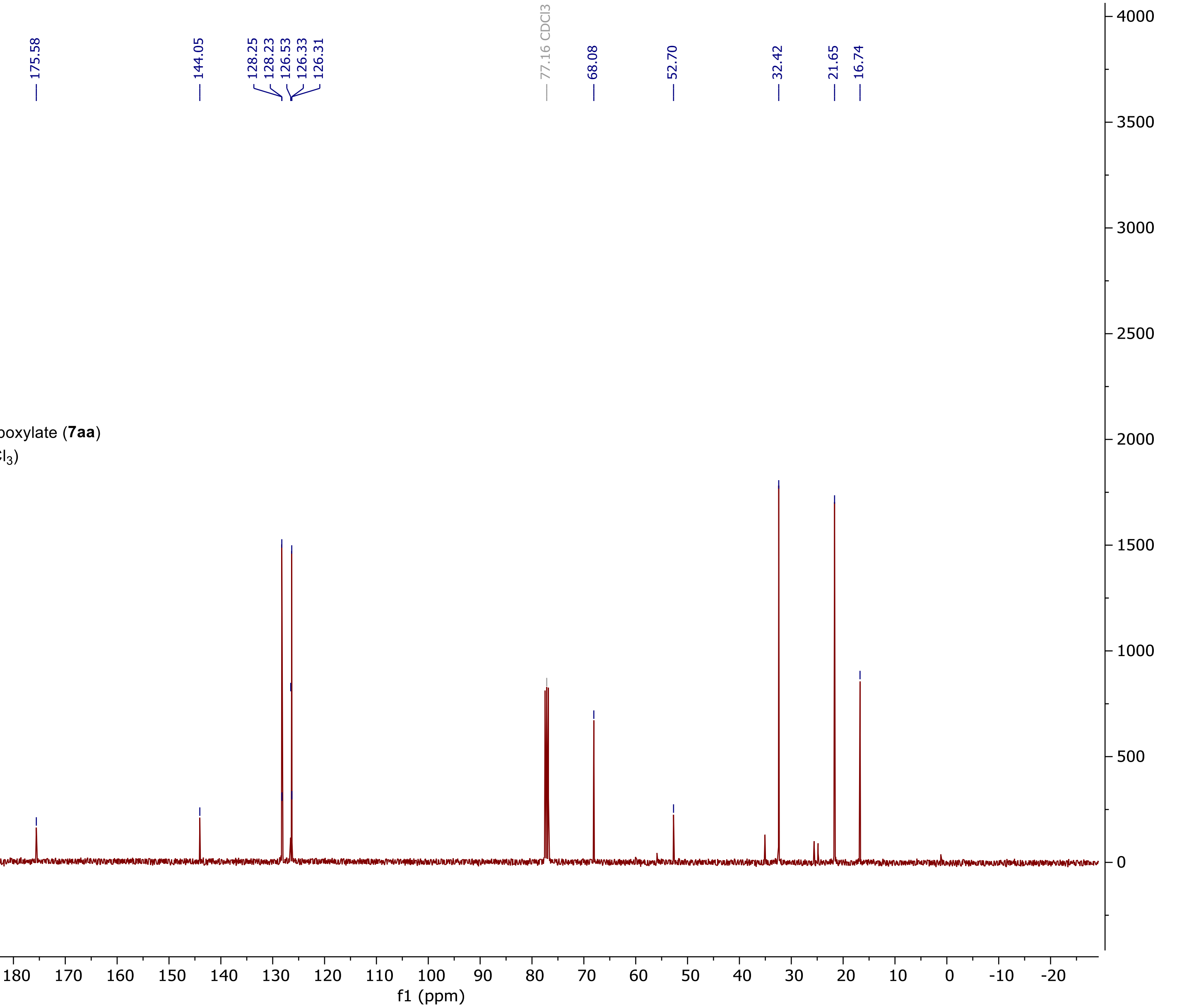

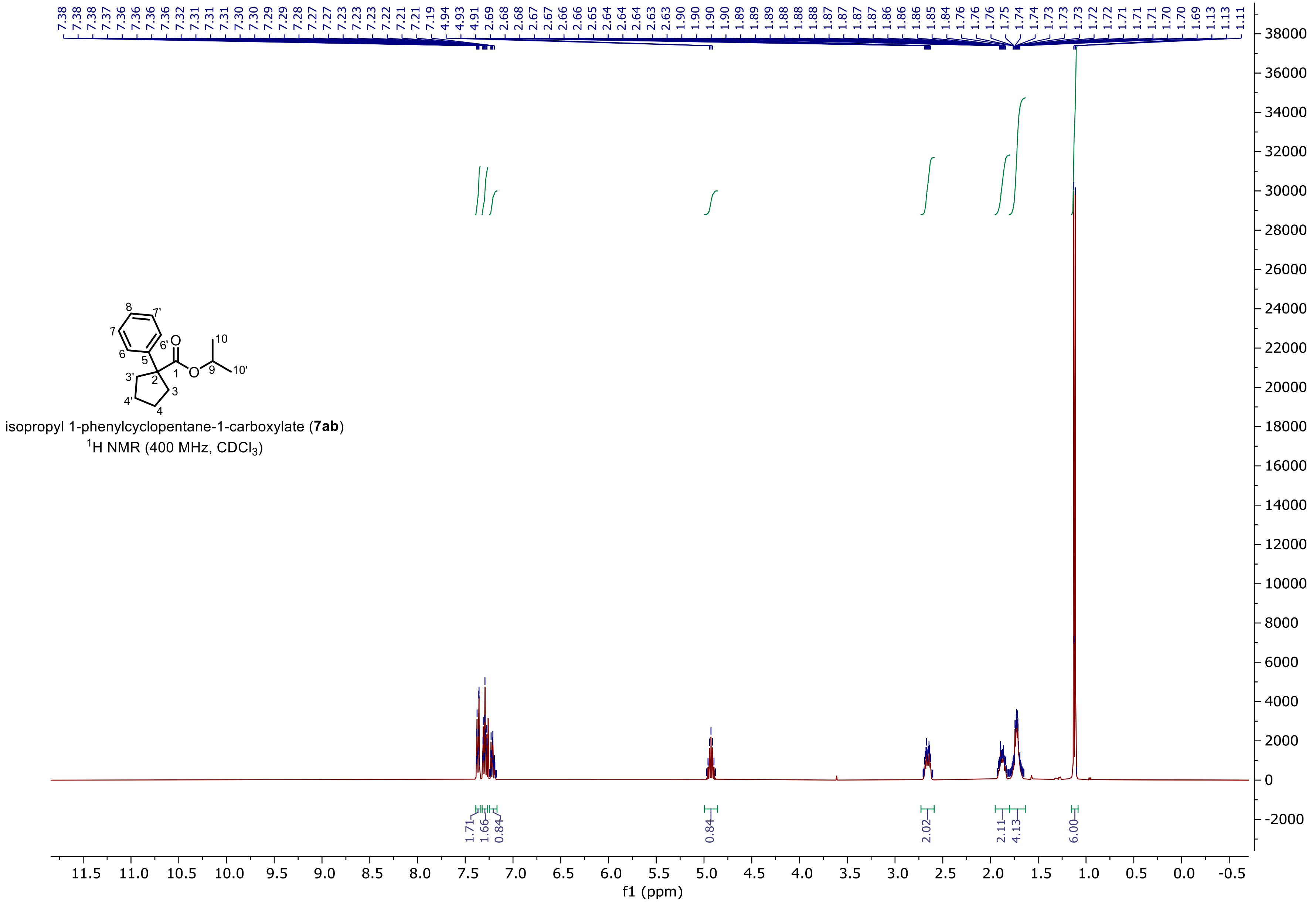

isopropyl 1-phenylcyclopentane-1-carboxylate (**7ab**)
^{1}H NMR (400 MHz, $CDCl_3$)
f1 (ppm)

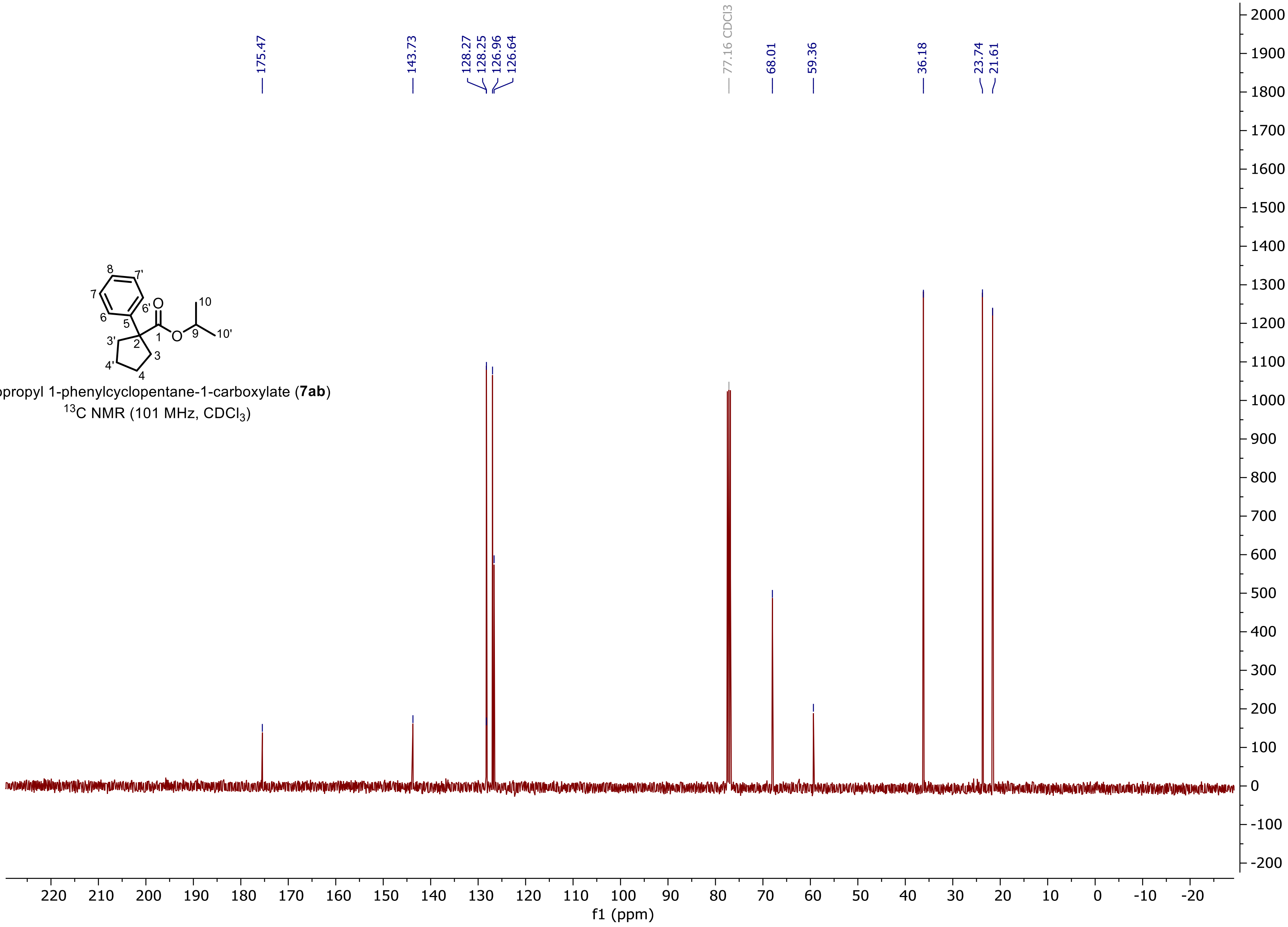

isopropyl 1-phenylcyclopentane-1-carboxylate (**7ab**)

^{13}C NMR (101 MHz, $CDCl_3$)

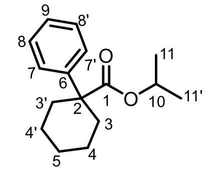

isopropyl 1-phenylcyclohexane-1-carboxylate (**7ac**)

^{1}H NMR (400 MHz, CDCl$_3$)

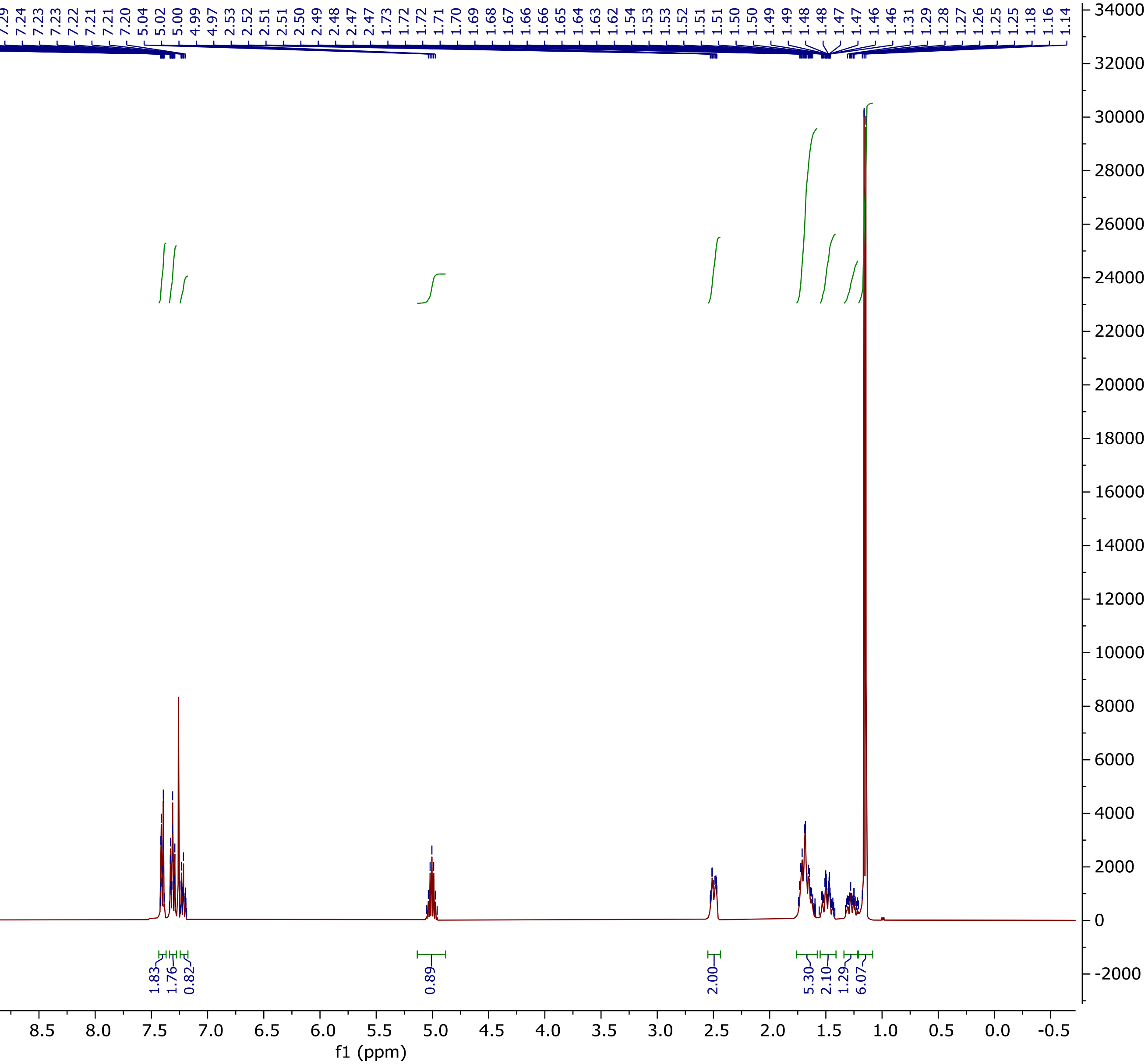

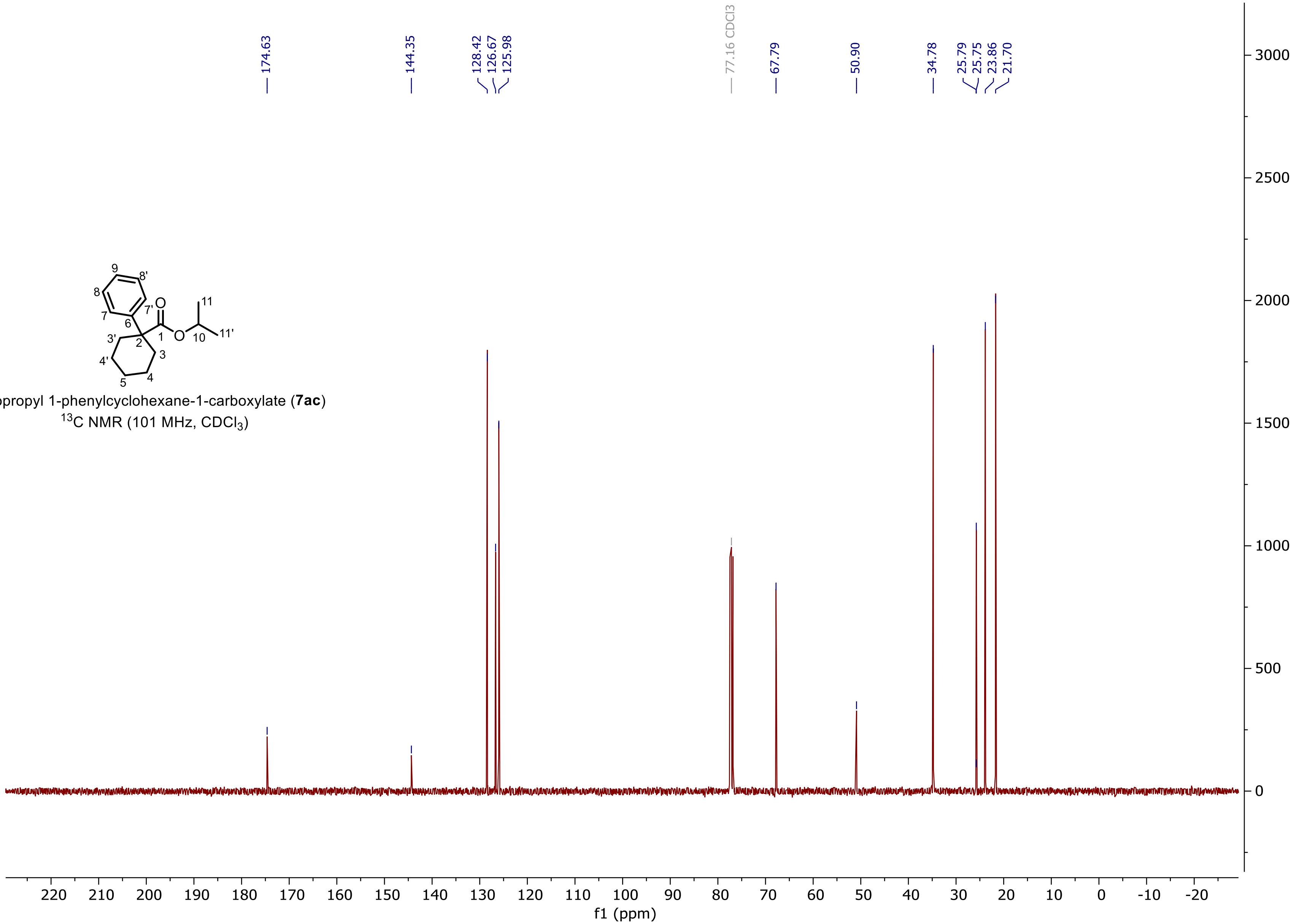

isopropyl 1-phenylcyclohexane-1-carboxylate (**7ac**)
^{13}C NMR (101 MHz, $CDCl_3$)

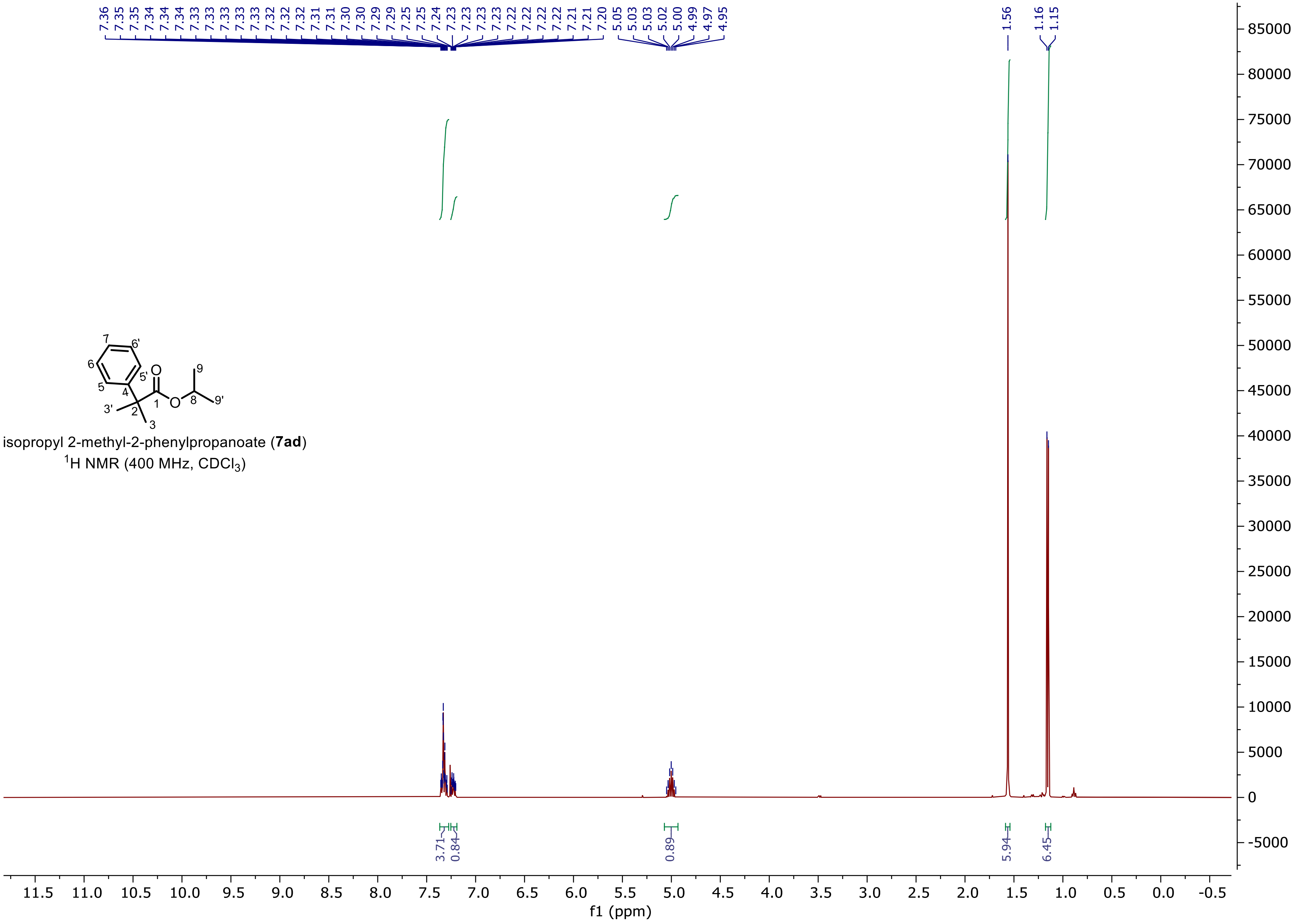

isopropyl 2-methyl-2-phenylpropanoate (**7ad**)

^{1}H NMR (400 MHz, $CDCl_3$)

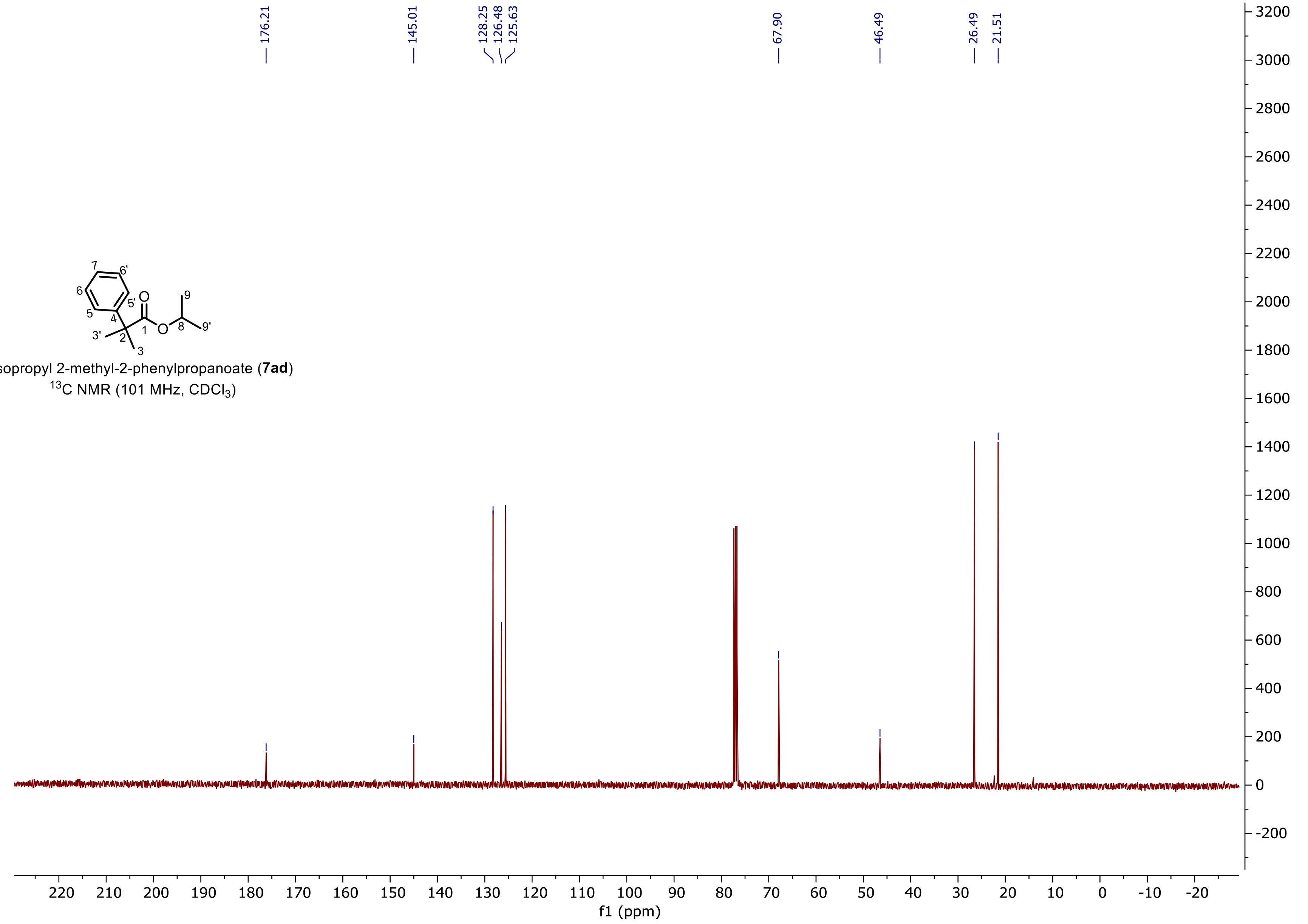

isopropyl 2-methyl-2-phenylpropanoate (**7ad**)

^{13}C NMR (101 MHz, $CDCl_3$)

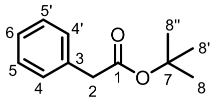

tert-butyl 2-phenylacetate (**7ae**)
^{1}H NMR (400 MHz, $CDCl_3$)

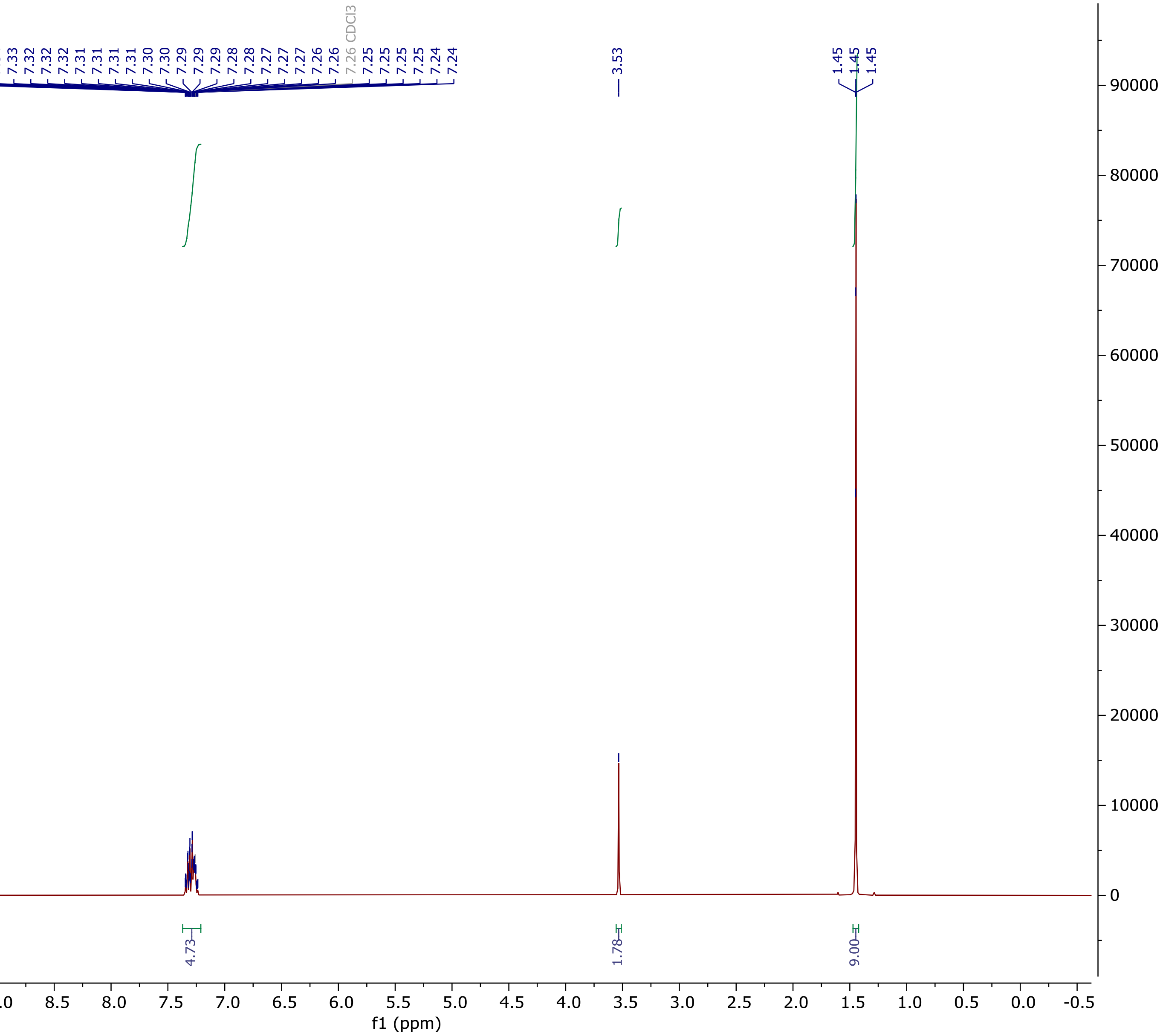

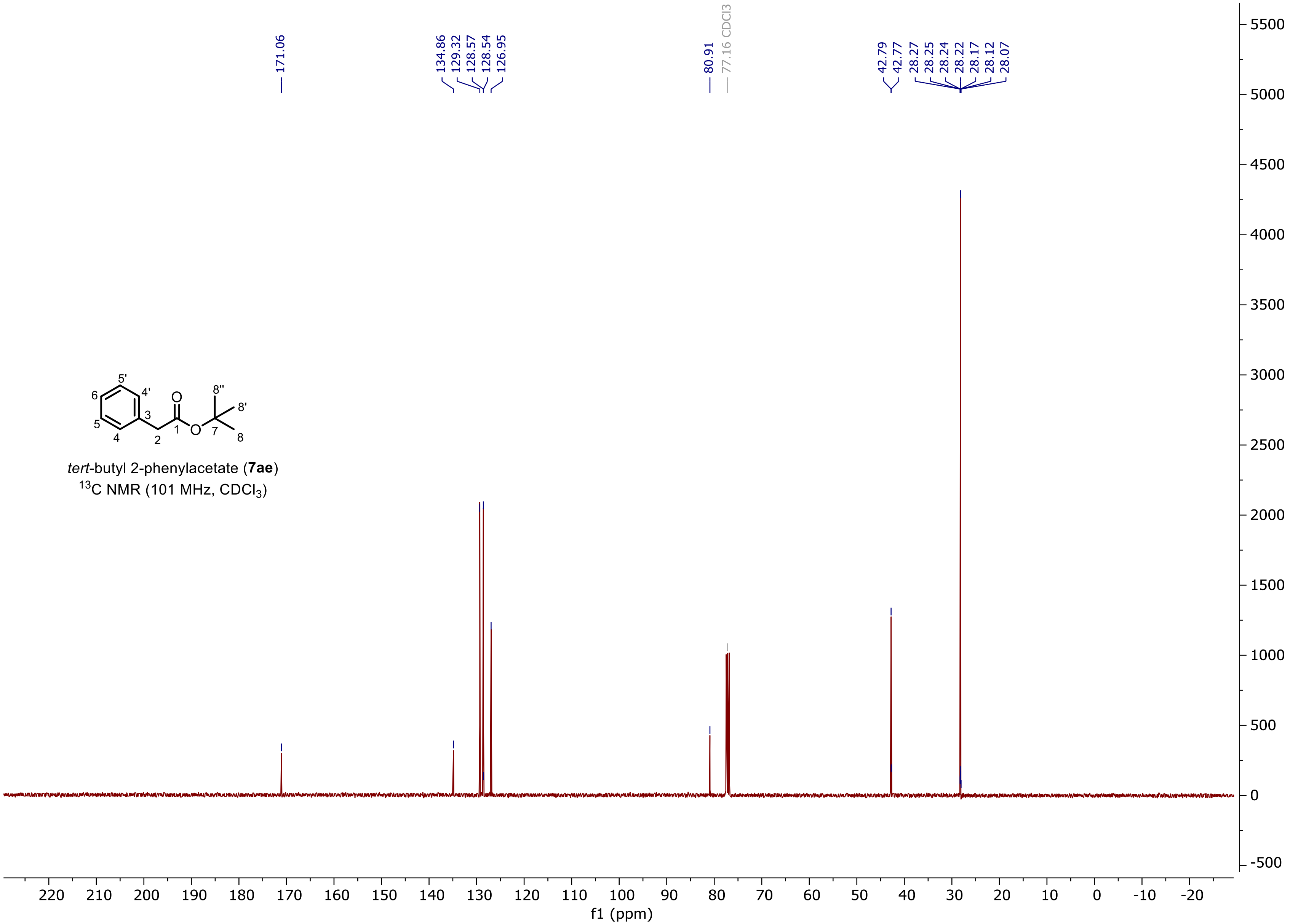
171.06
134.86
129.32
128.57
128.54
126.95
80.91
77.16 CDCl3
42.79
42.77
28.27
28.25
28.24
28.22
28.17
28.12
28.07
tert-butyl 2-phenylacetate (**7ae**)
^{13}C NMR (101 MHz, $CDCl_3$)
f1 (ppm)

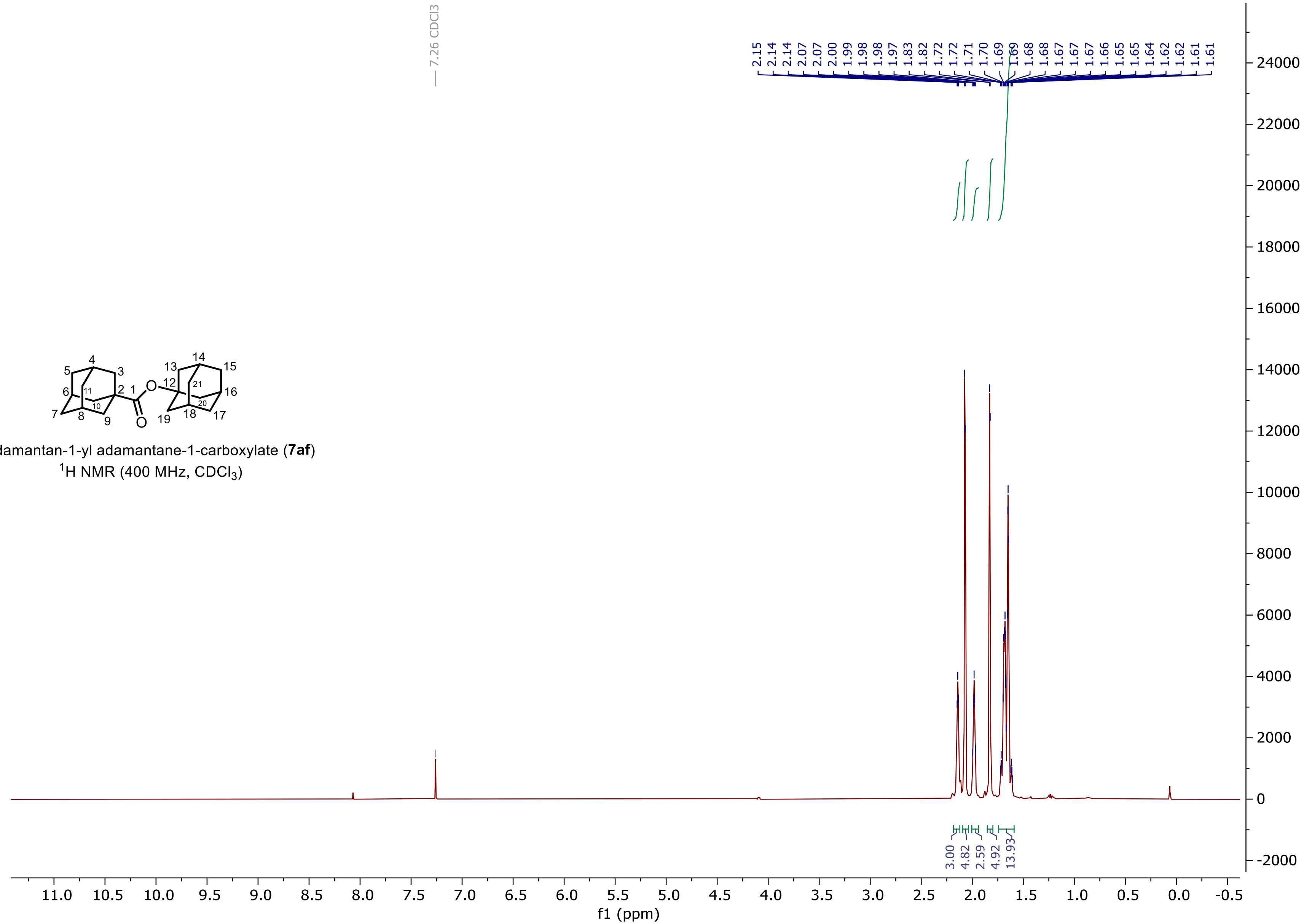

adamantan-1-yl adamantane-1-carboxylate (**7af**)
^{1}H NMR (400 MHz, $CDCl_3$)

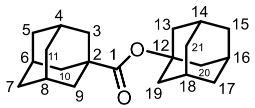

adamantan-1-yl adamantane-1-carboxylate (**7af**)

^{13}C NMR (101 MHz, $CDCl_3$)

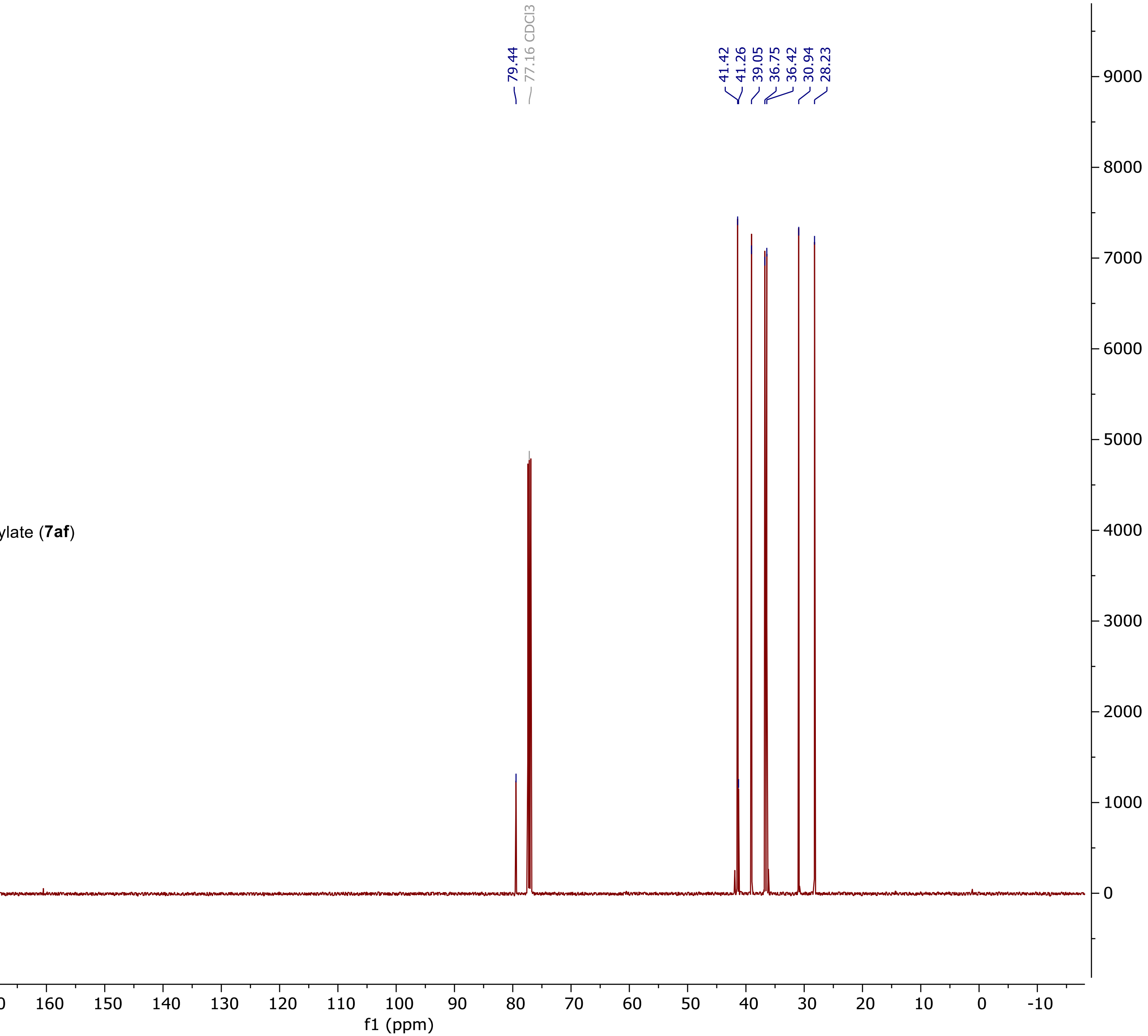

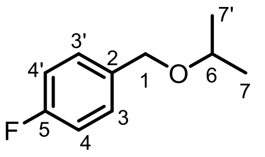

1-fluoro-4-(isopropoxymethyl)benzene (**8a**)
^{1}H NMR (400 MHz, $CDCl_3$)

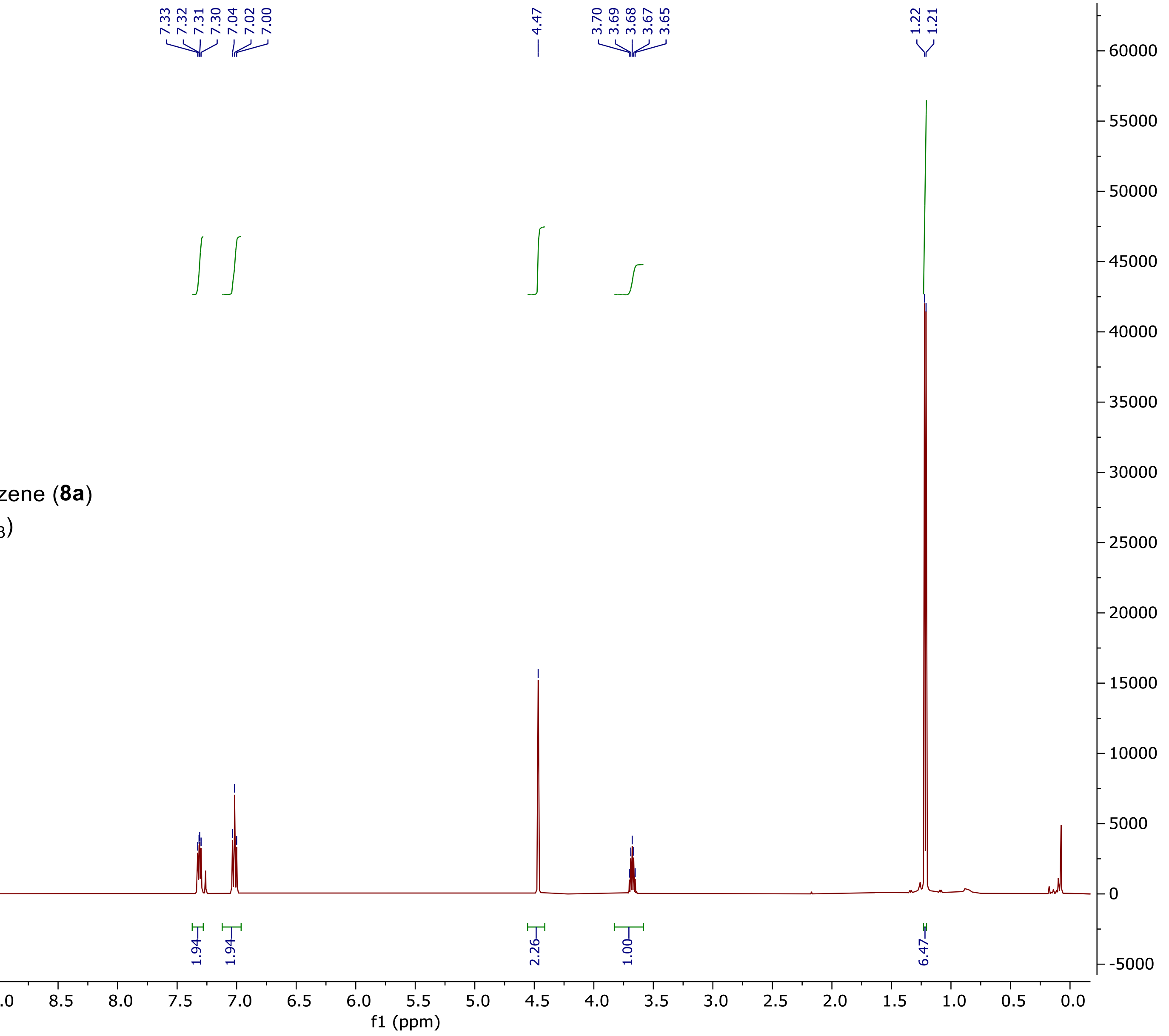

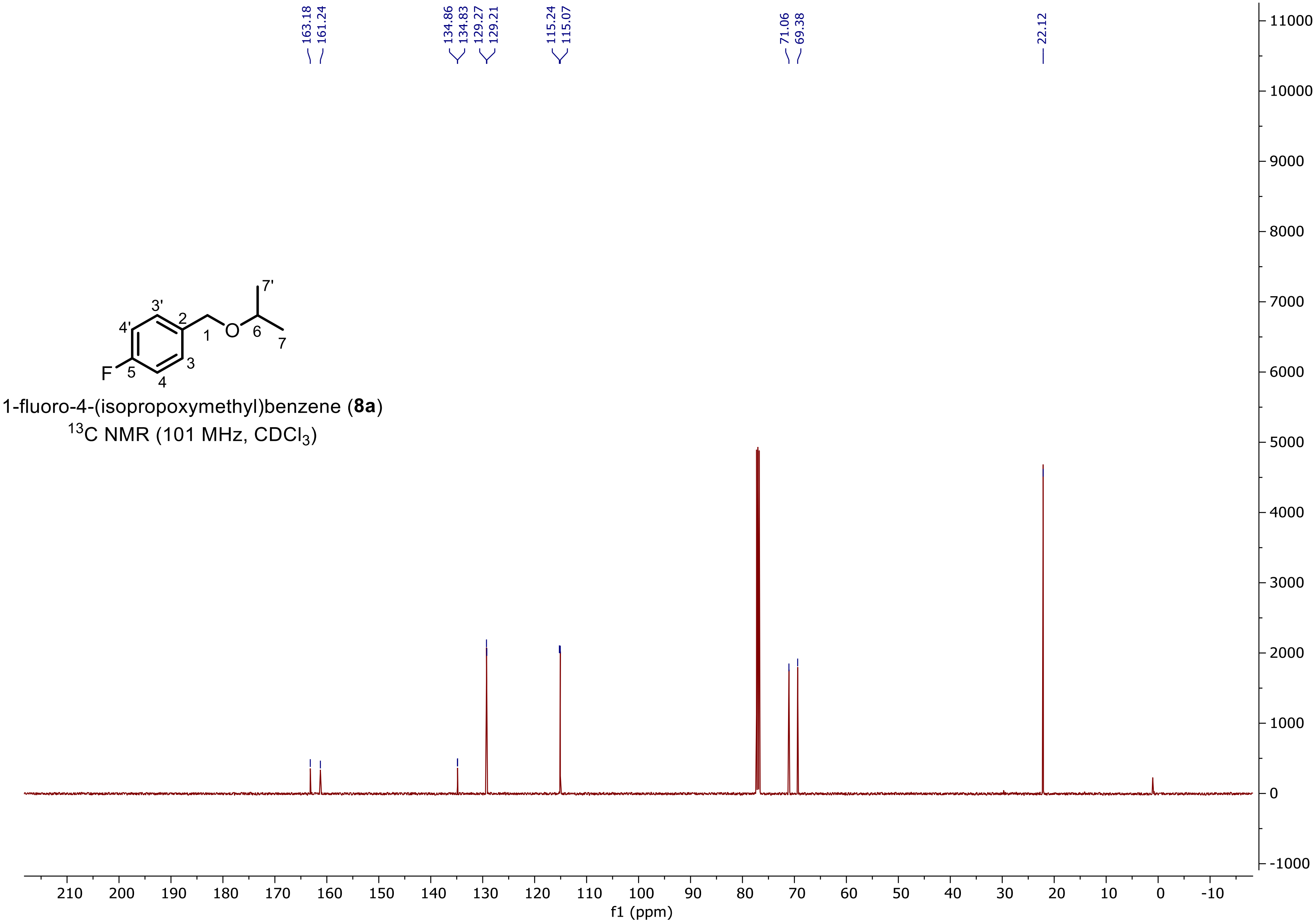
163.18
161.24
134.86
134.83
129.27
129.21
115.24
115.07
71.06
69.38
22.12
7'
3'
2
4'
1
O
6
7
F
5
3
4
1-fluoro-4-(isopropoxymethyl)benzene (**8a**)
^{13}C NMR (101 MHz, $CDCl_3$)
11000
10000
9000
8000
7000
6000
5000
4000
3000
2000
1000
0
-1000
210 200 190 180 170 160 150 140 130 120 110 100 90 80 70 60 50 40 30 20 10 0 -10
f1 (ppm)

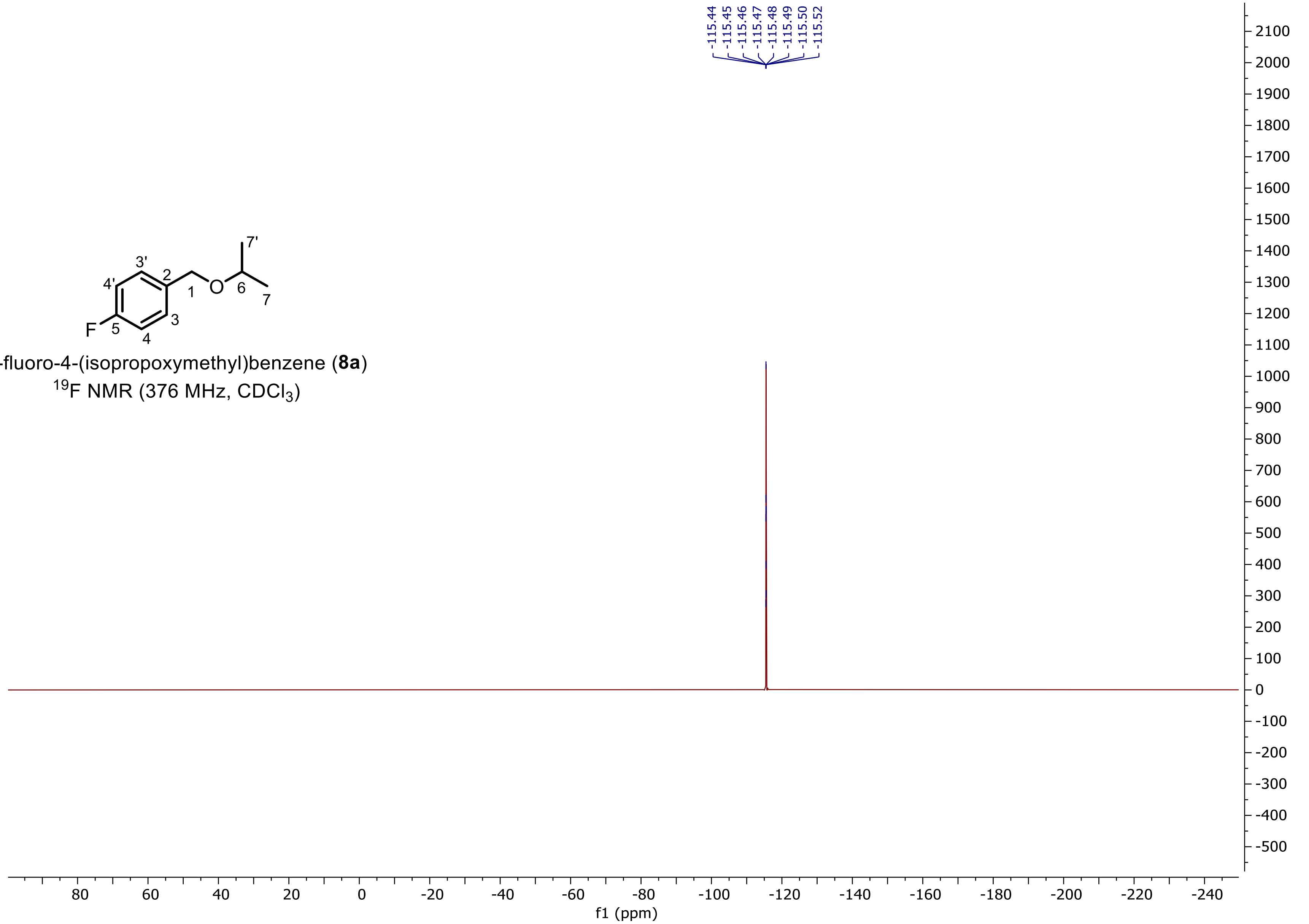

1-fluoro-4-(isopropoxymethyl)benzene (**8a**)

^{19}F NMR (376 MHz, $CDCl_3$)

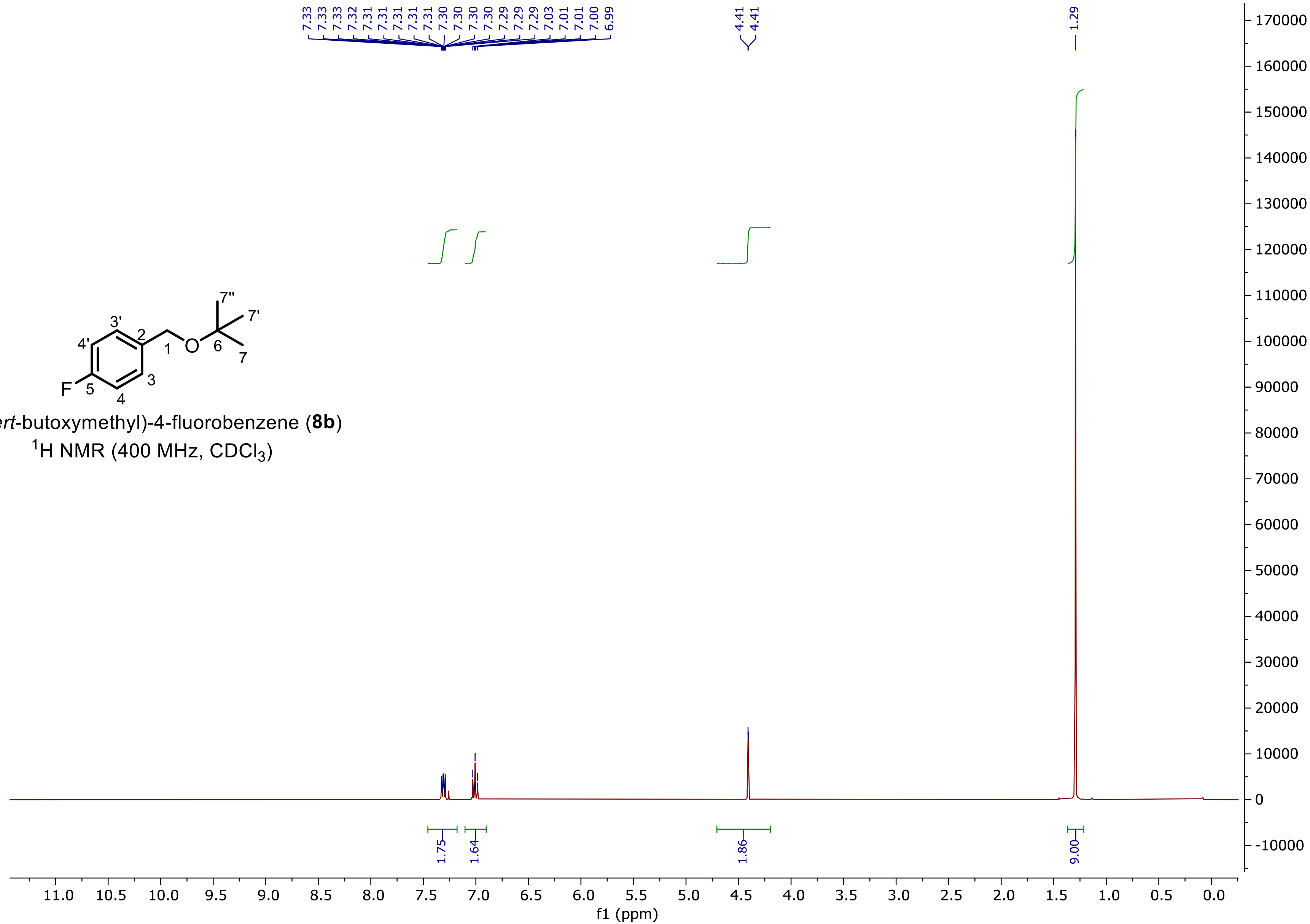

1-(*tert*-butoxymethyl)-4-fluorobenzene (**8b**)

^{1}H NMR (400 MHz, $CDCl_3$)

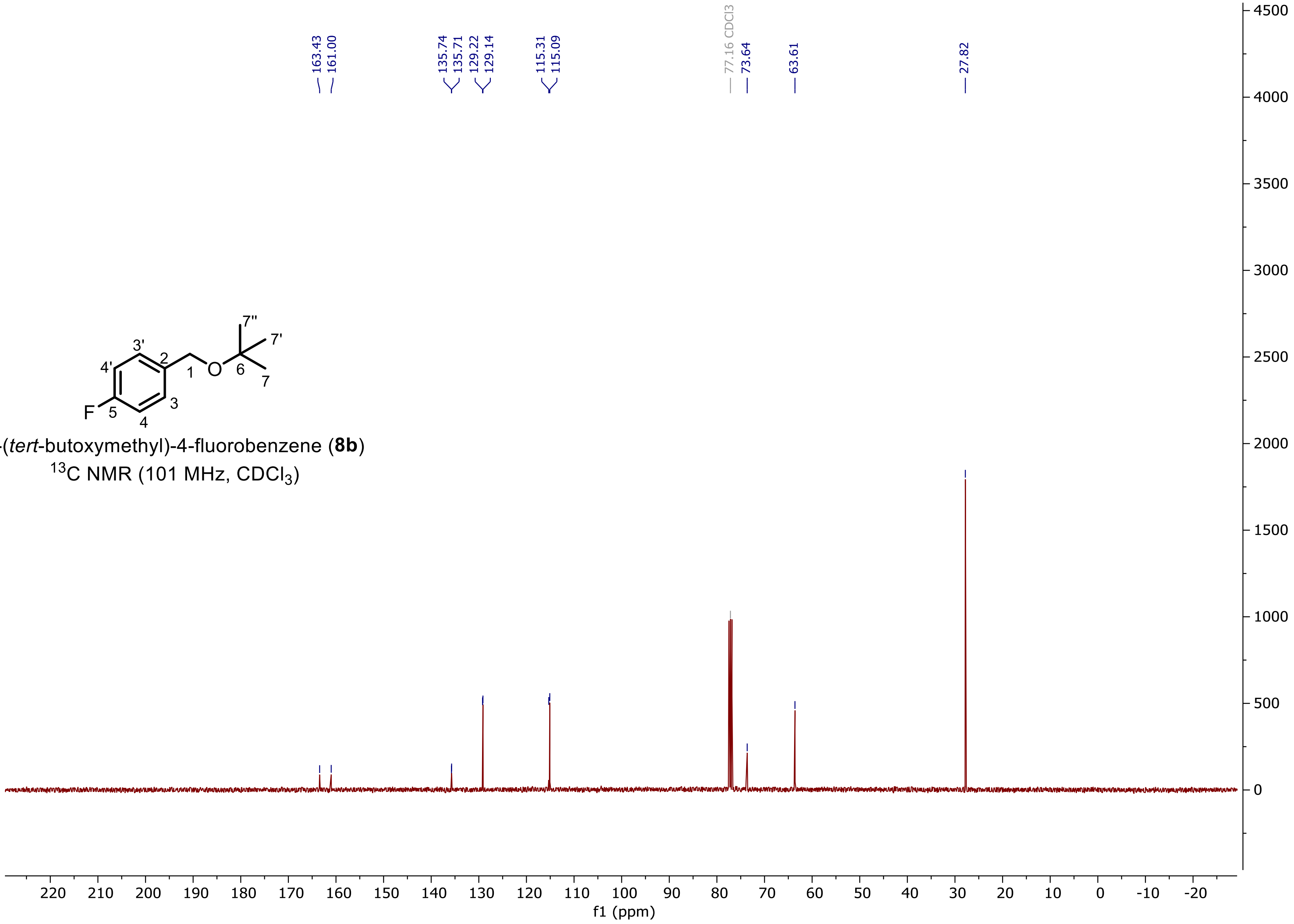

1-(*tert*-butoxymethyl)-4-fluorobenzene (**8b**)

^{13}C NMR (101 MHz, $CDCl_3$)

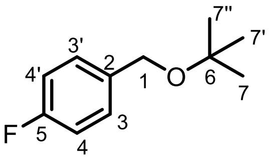

1-(*tert*-butoxymethyl)-4-fluorobenzene (**8b**)
^{19}F NMR (376 MHz, CDCl$_3$)

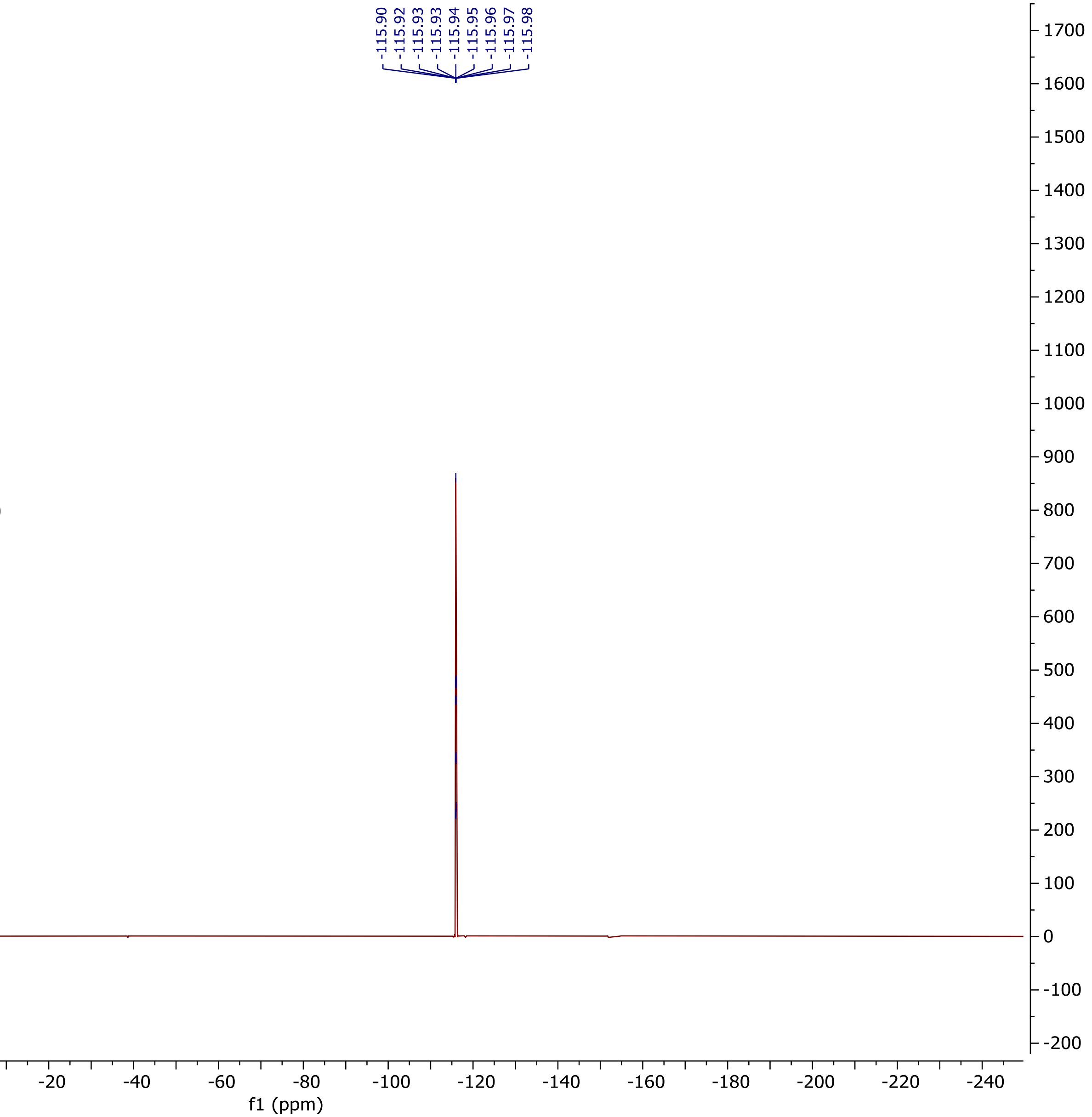

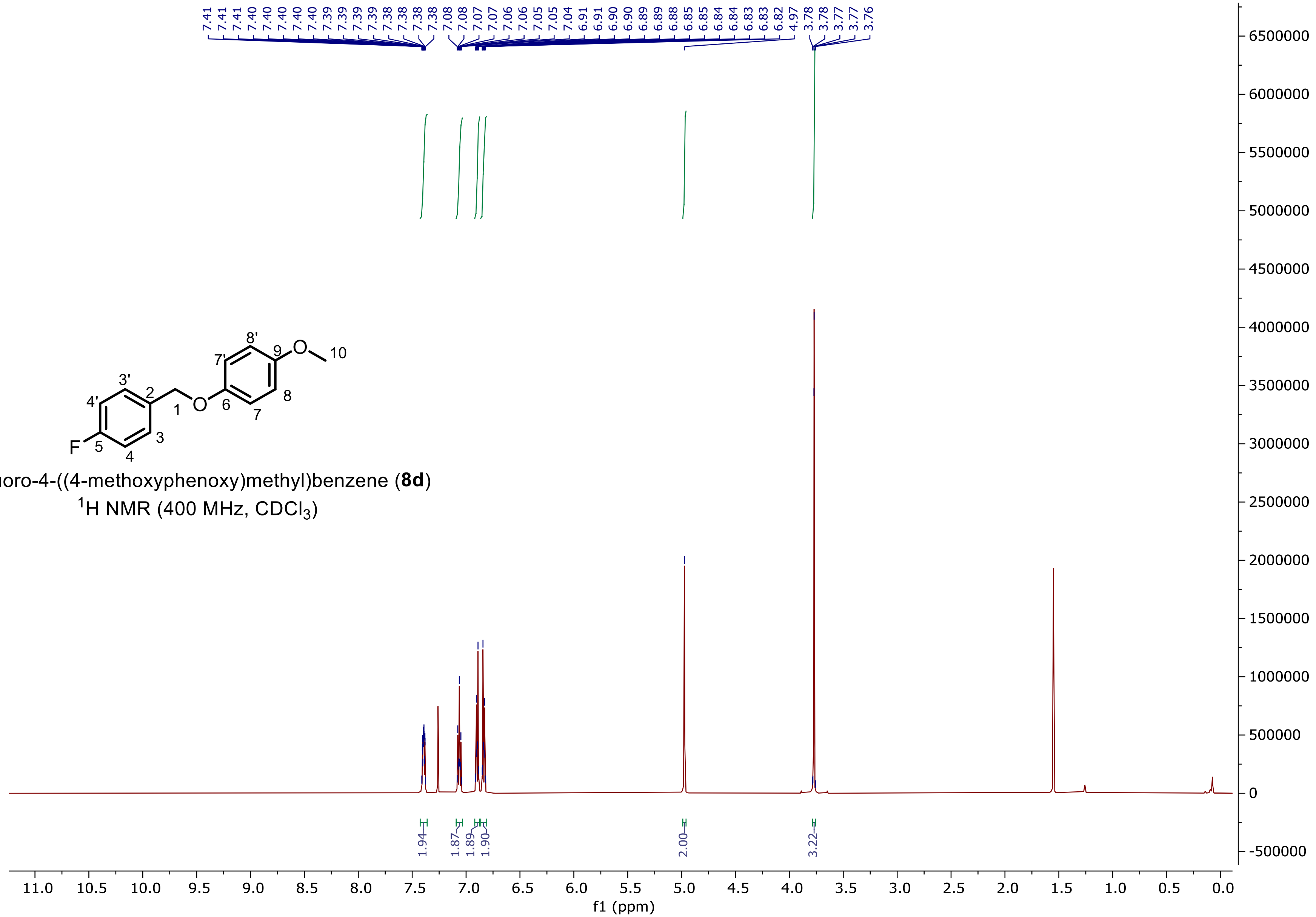

1-fluoro-4-((4-methoxyphenoxy)methyl)benzene (**8d**)
^{1}H NMR (400 MHz, $CDCl_3$)

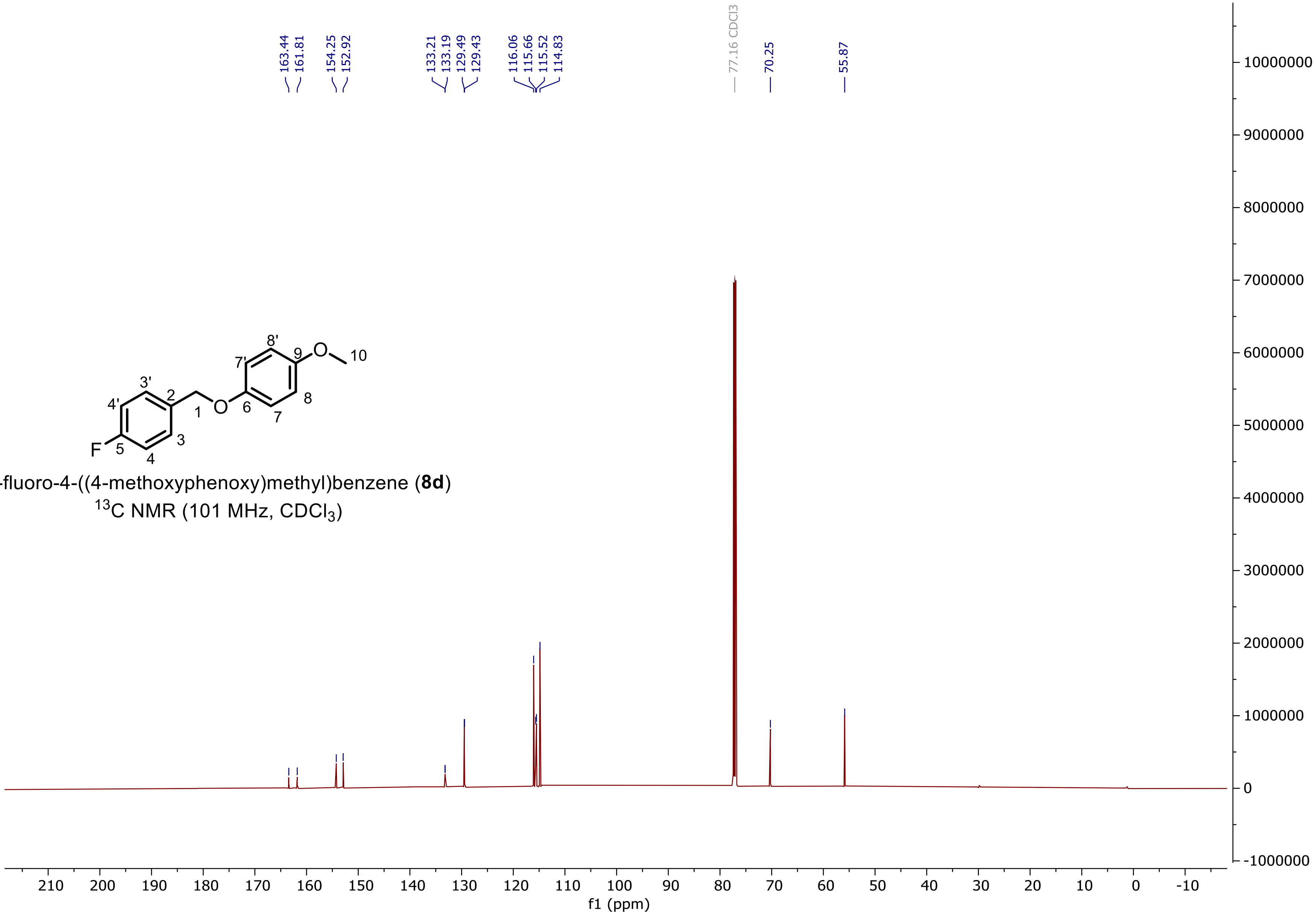

1-fluoro-4-((4-methoxyphenoxy)methyl)benzene (**8d**)

^{13}C NMR (101 MHz, $CDCl_3$)

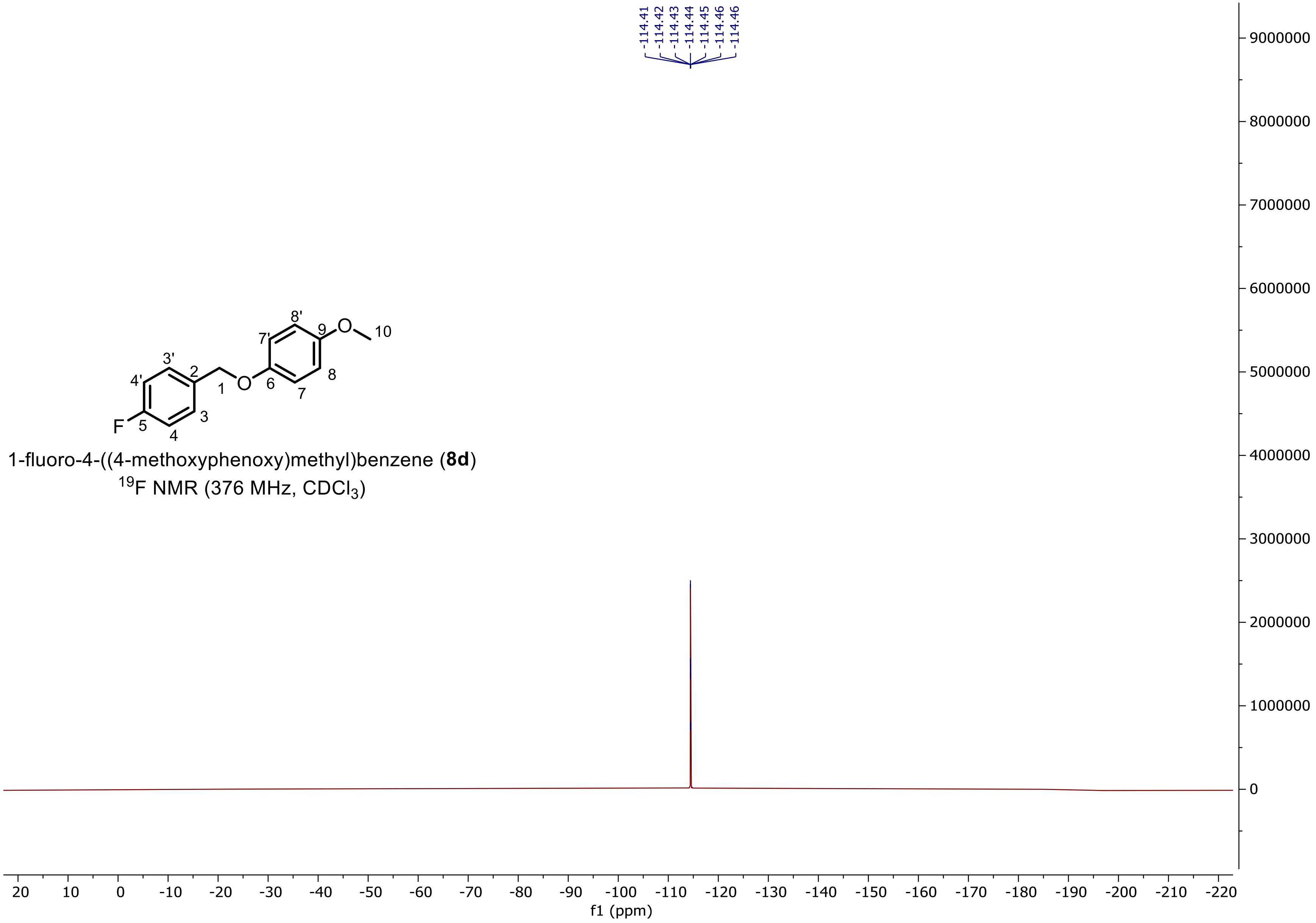

1-fluoro-4-((4-methoxyphenoxy)methyl)benzene (**8d**)

^{19}F NMR (376 MHz, $CDCl_3$)

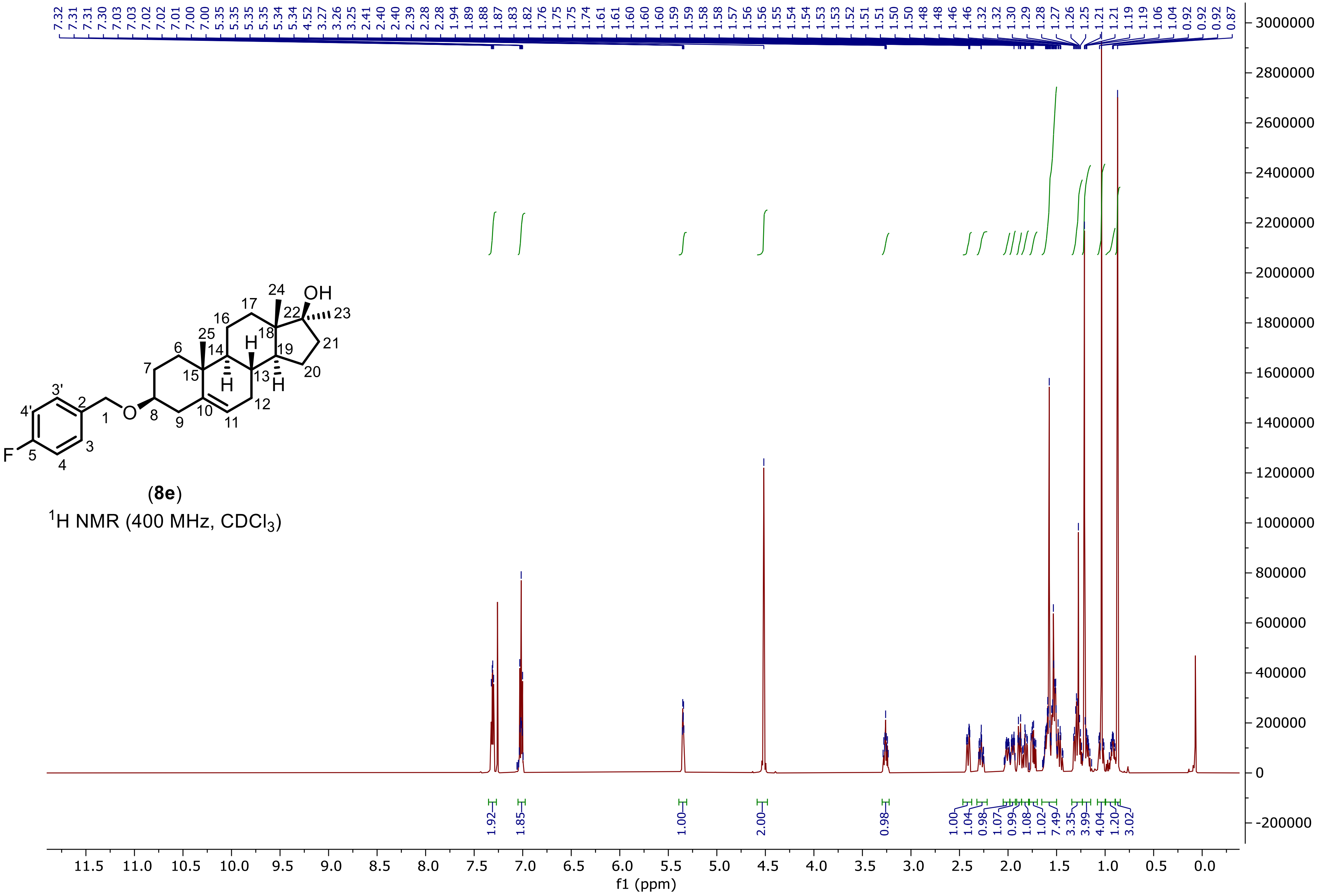

(8e)
^{1}H NMR (400 MHz, CDCl$_3$)
f1 (ppm)

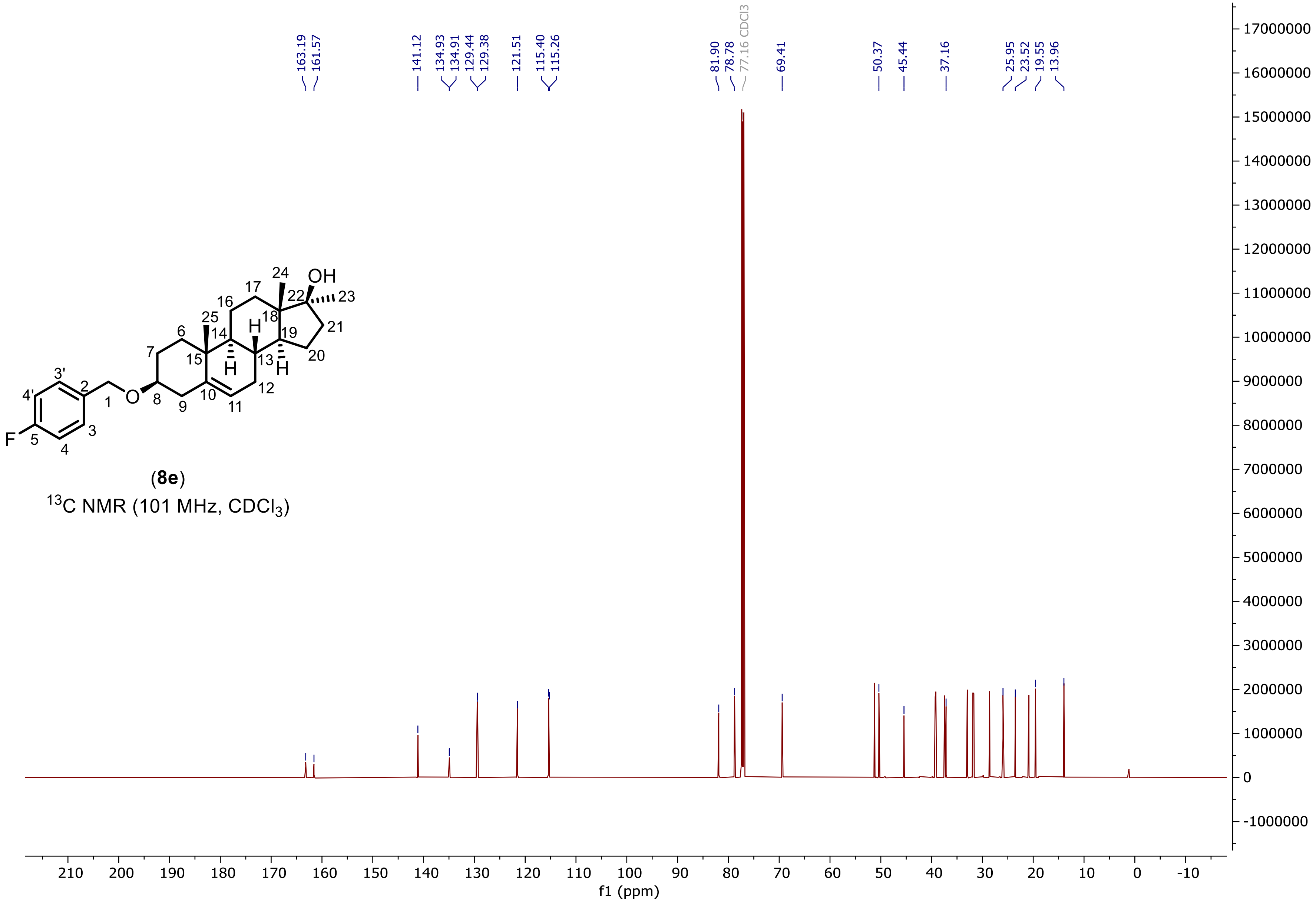
163.19
161.57
141.12
134.93
134.91
129.44
129.38
121.51
115.40
115.26
81.90
78.78
77.16 CDCl3
69.41
50.37
45.44
37.16
25.95
23.52
19.55
13.96
(8e)
^{13}C NMR (101 MHz, $CDCl_3$)
f1 (ppm)

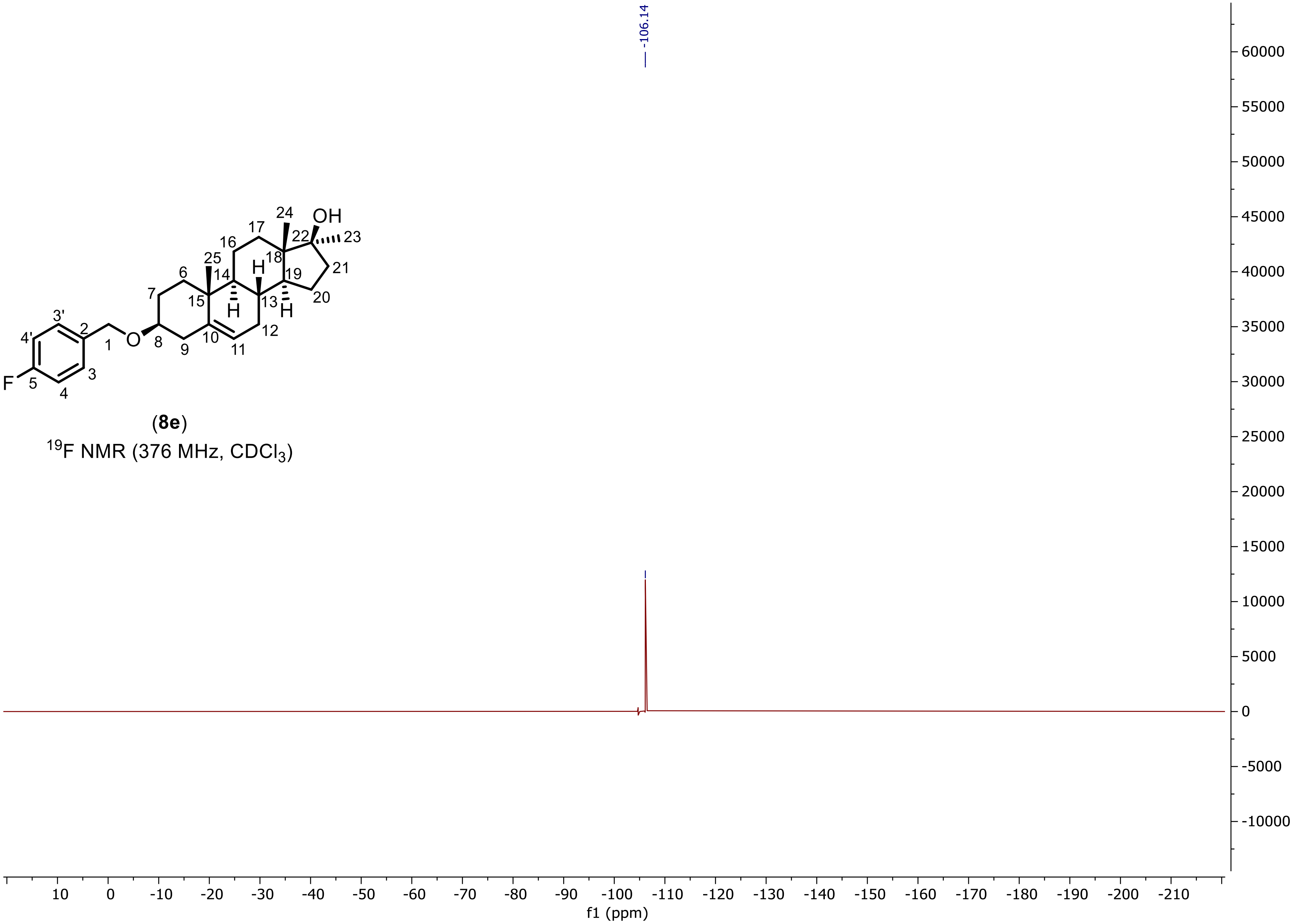

(**8e**)

^{19}F NMR (376 MHz, $CDCl_3$)

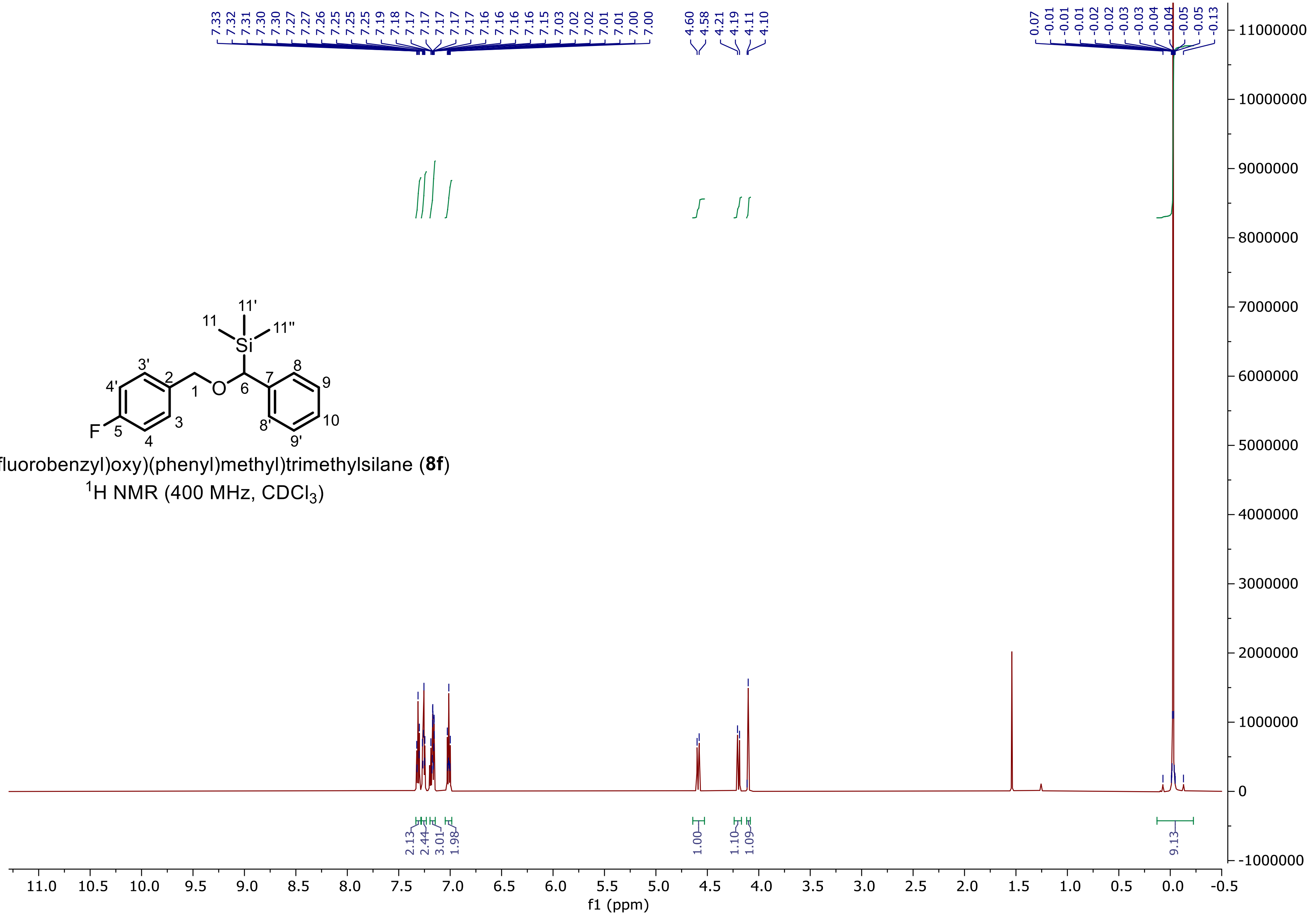

(((4-fluorobenzyl)oxy)(phenyl)methyl)trimethylsilane (**8f**)

^{1}H NMR (400 MHz, $CDCl_3$)

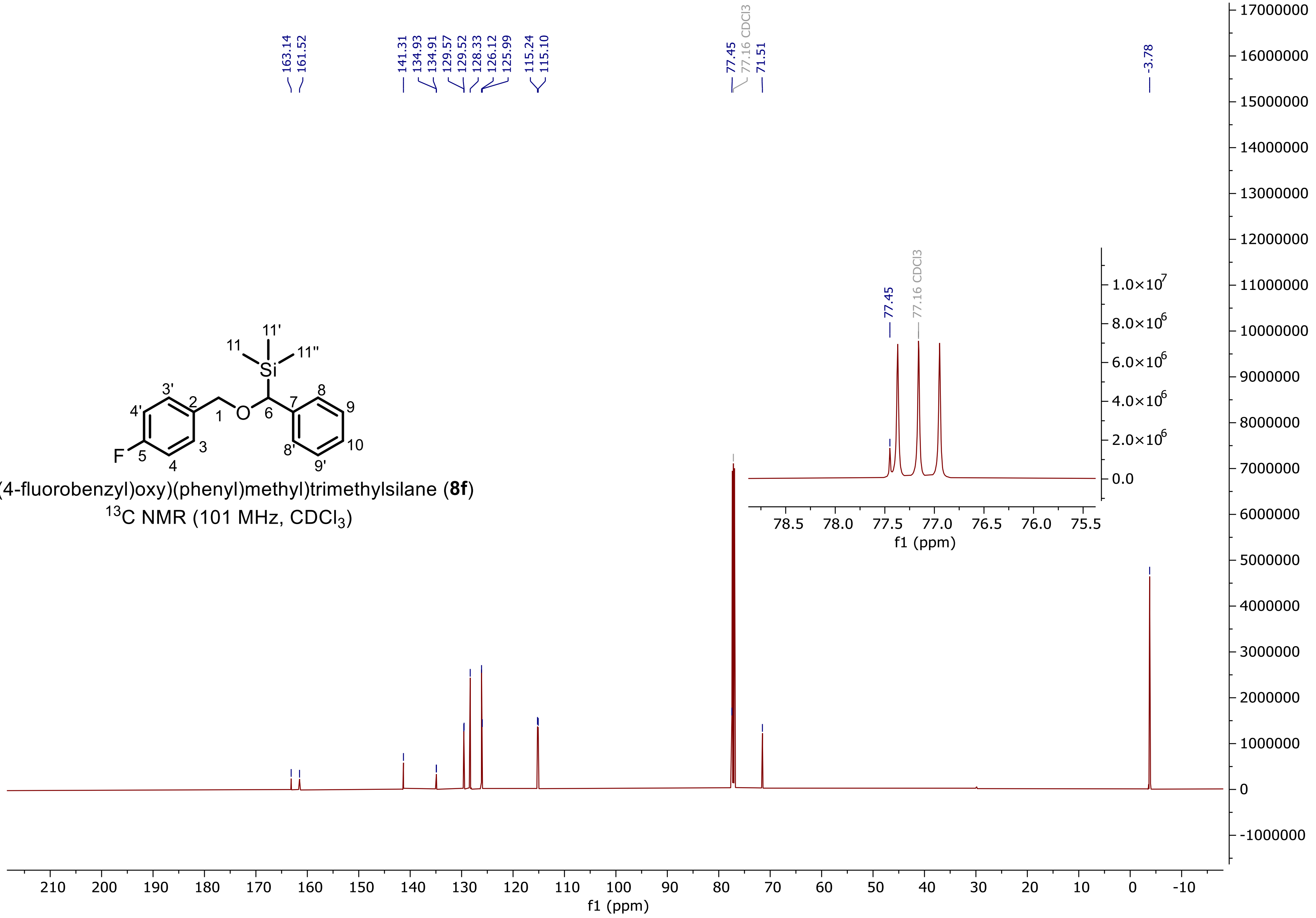

(((4-fluorobenzyl)oxy)(phenyl)methyl)trimethylsilane (**8f**)

^{13}C NMR (101 MHz, $CDCl_3$)

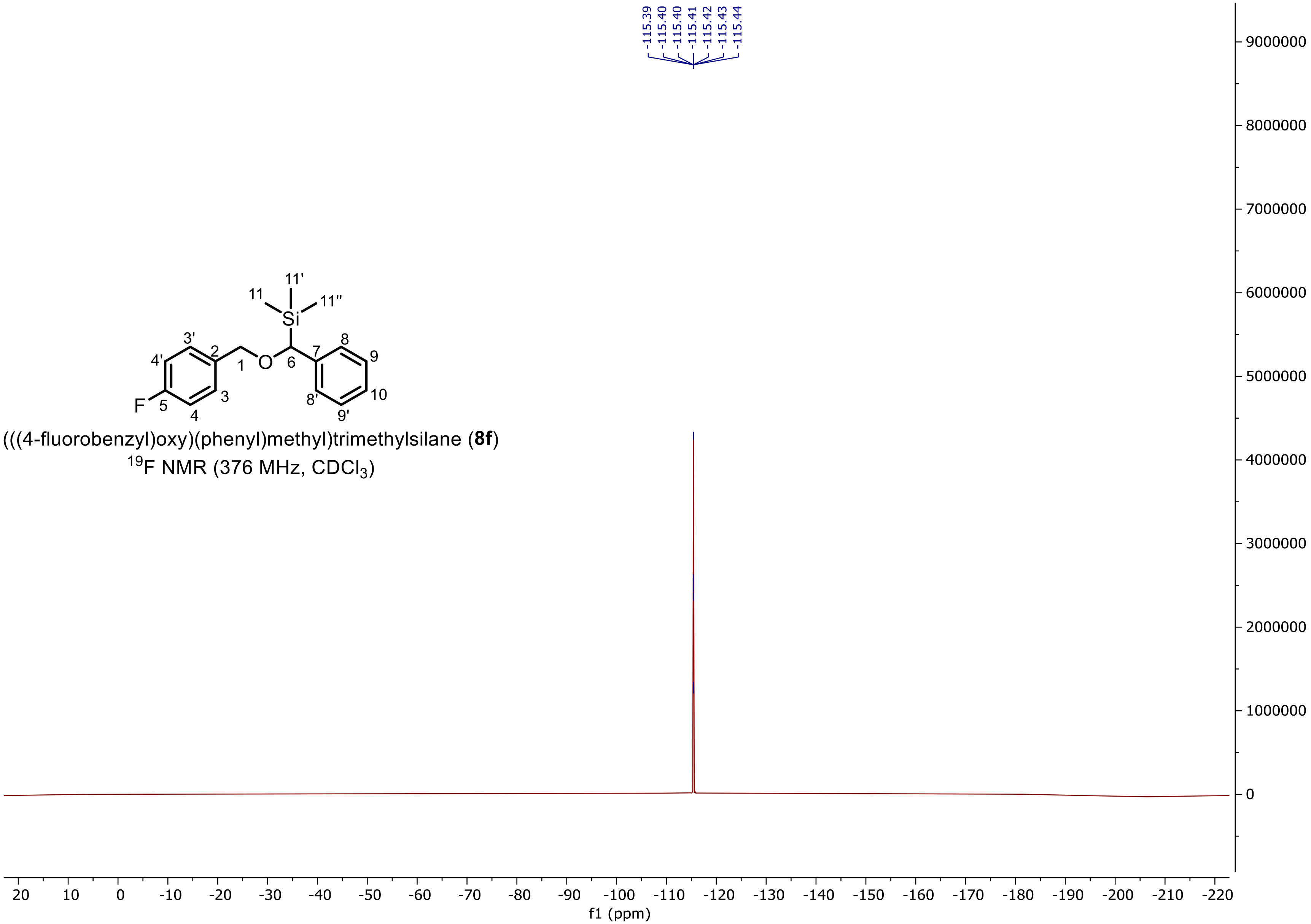

(((4-fluorobenzyl)oxy)(phenyl)methyl)trimethylsilane (**8f**)

^{19}F NMR (376 MHz, $CDCl_3$)

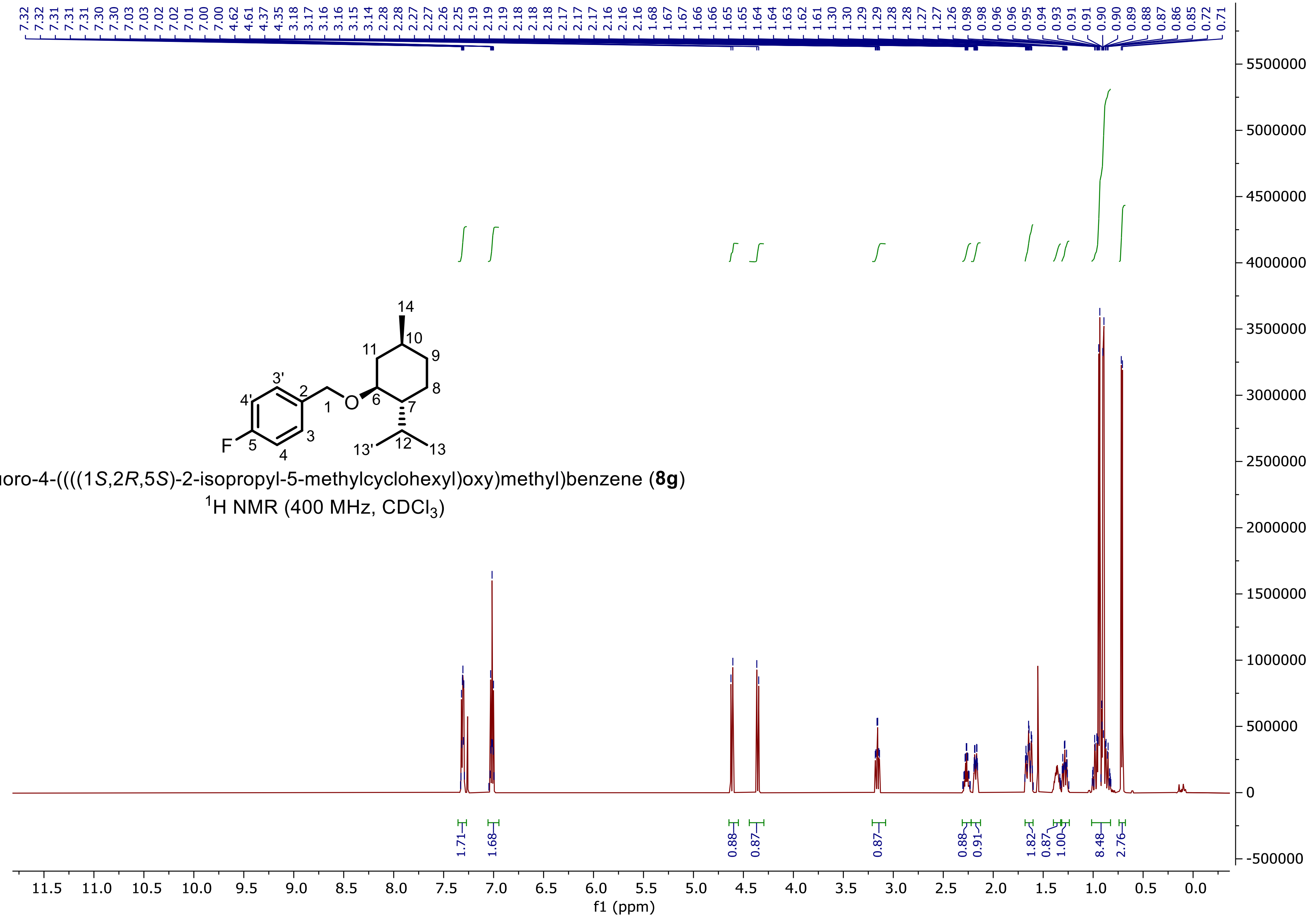

1-fluoro-4-((((1*S*,2*R*,5*S*)-2-isopropyl-5-methylcyclohexyl)oxy)methyl)benzene (**8g**)

^{1}H NMR (400 MHz, $CDCl_3$)

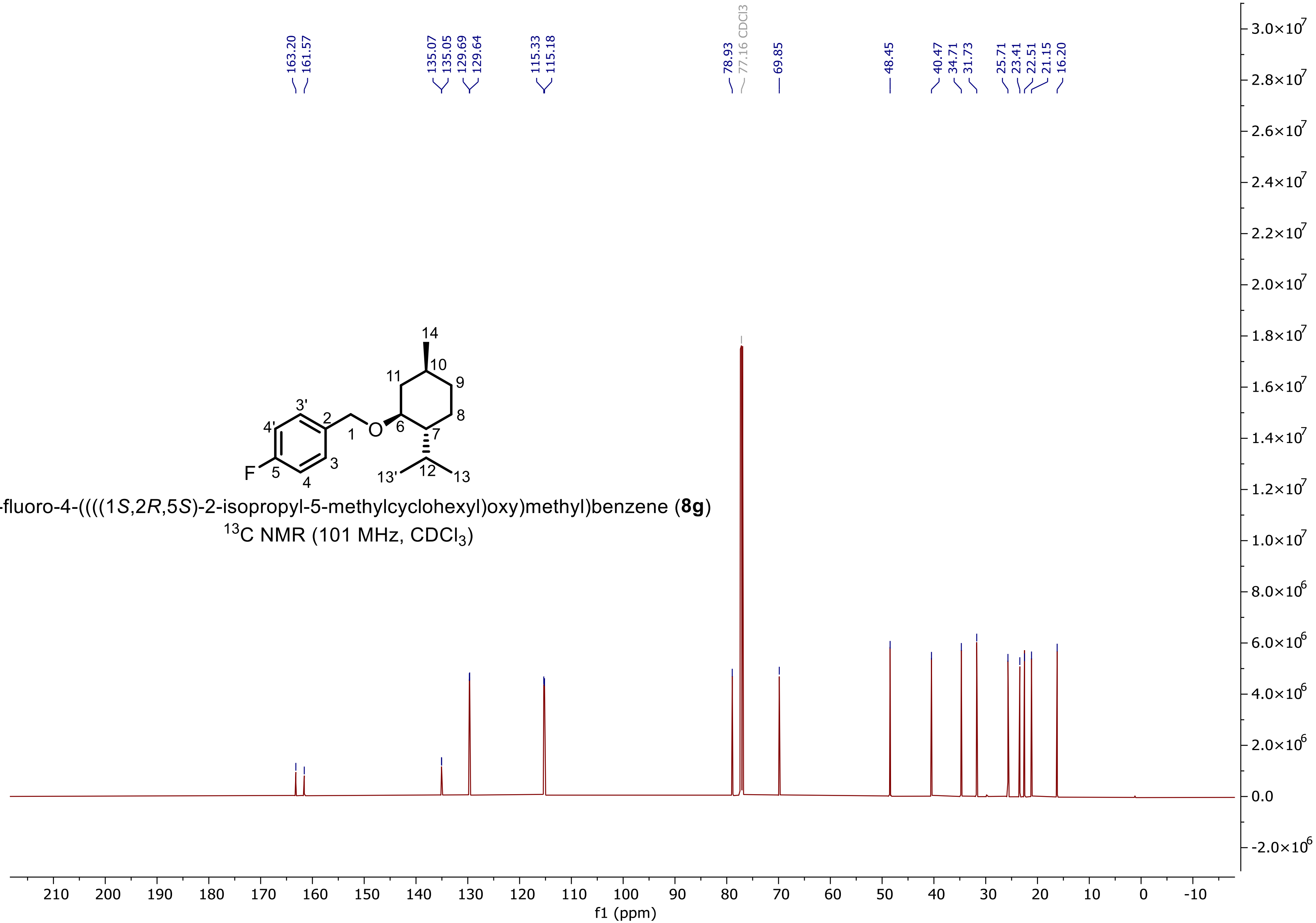

1-fluoro-4-((((1*S*,2*R*,5*S*)-2-isopropyl-5-methylcyclohexyl)oxy)methyl)benzene (**8g**)

^{13}C NMR (101 MHz, $CDCl_3$)

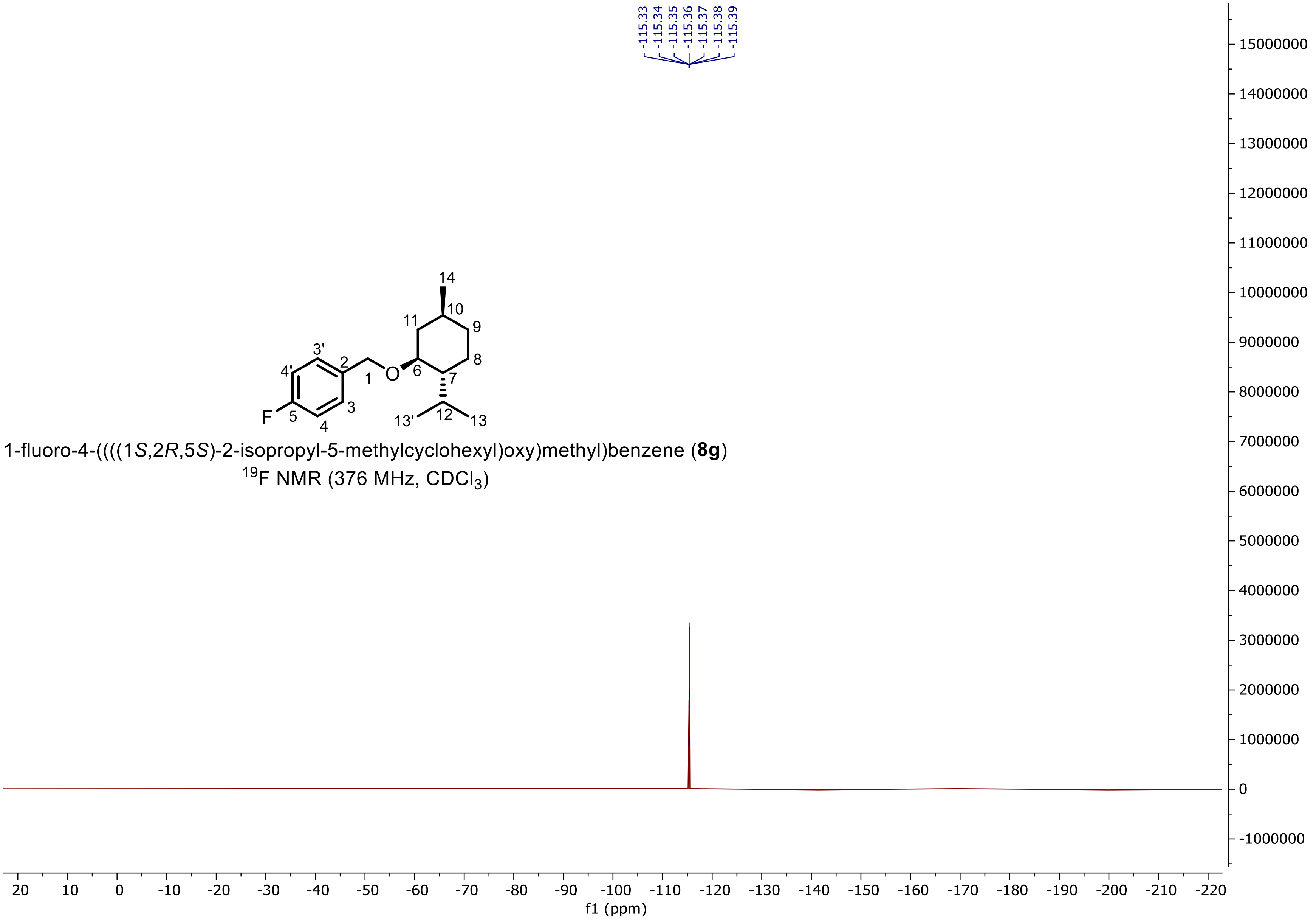

1-fluoro-4-((((1*S*,2*R*,5*S*)-2-isopropyl-5-methylcyclohexyl)oxy)methyl)benzene (**8g**)

^{19}F NMR (376 MHz, $CDCl_3$)

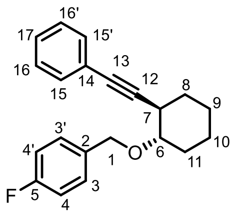

1-fluoro-4-(((2-(phenylethynyl)cyclohexyl)oxy)methyl)benzene (**8h**)

^{1}H NMR (400 MHz, $CDCl_3$)

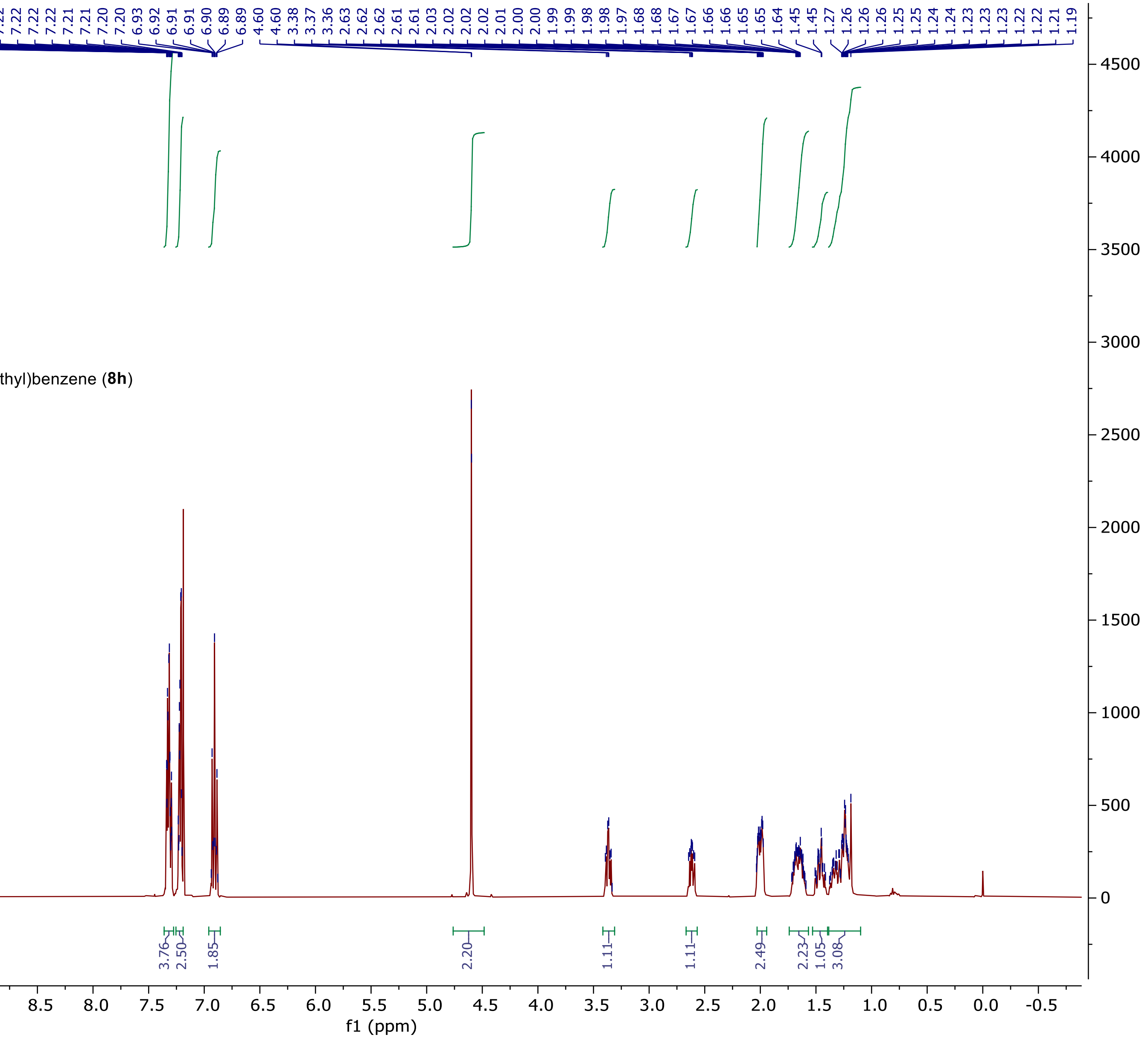

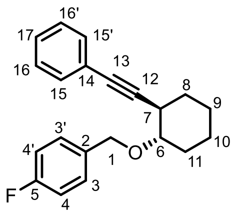

1-fluoro-4-(((2-(phenylethynyl)cyclohexyl)oxy)methyl)benzene (**8h**)

^{13}C NMR (101 MHz, $CDCl_3$)

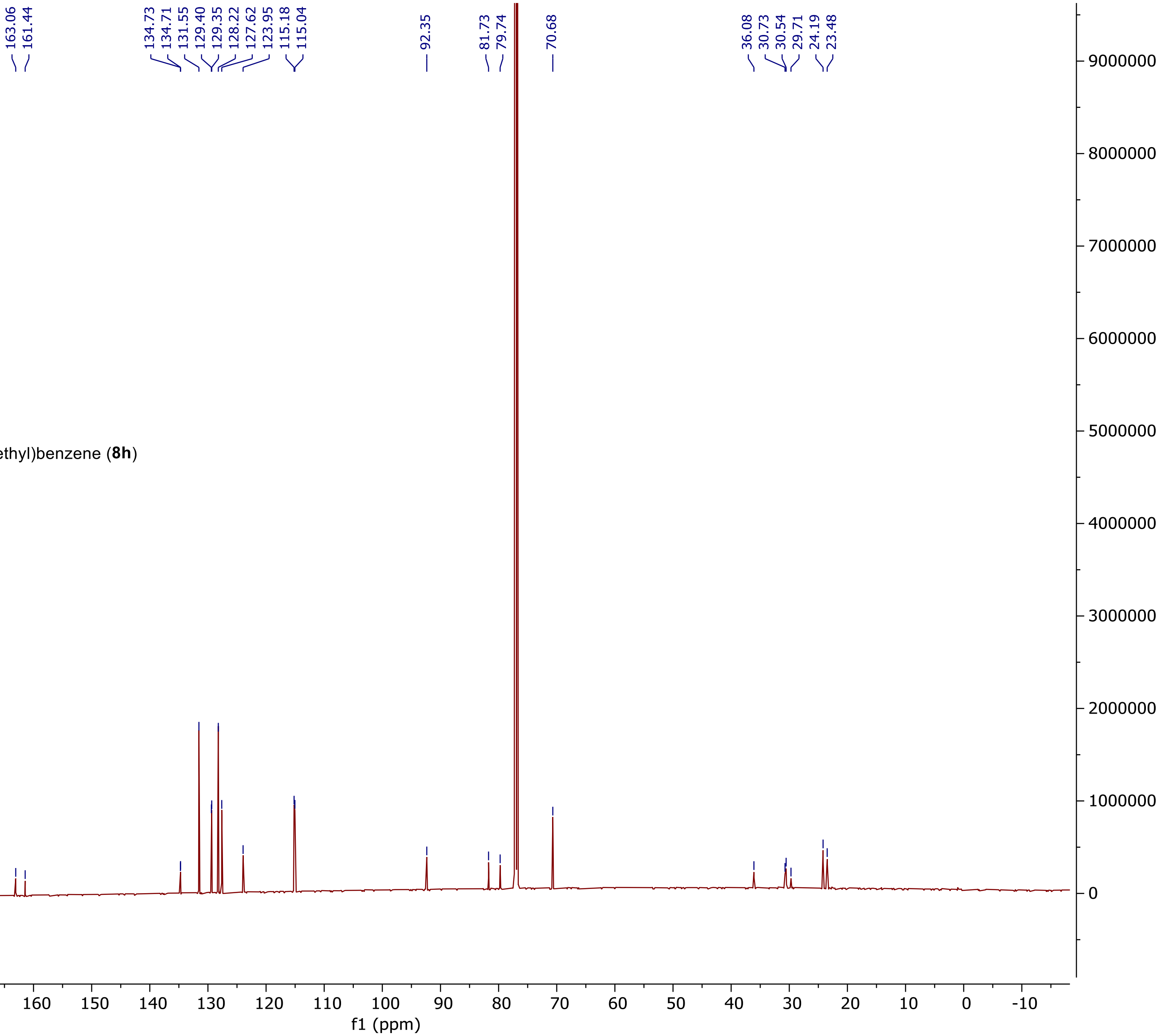

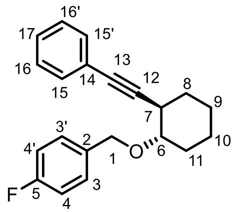

1-fluoro-4-(((2-(phenylethynyl)cyclohexyl)oxy)methyl)benzene (**8h**)

^{19}F NMR (376 MHz, CDCl$_3$)

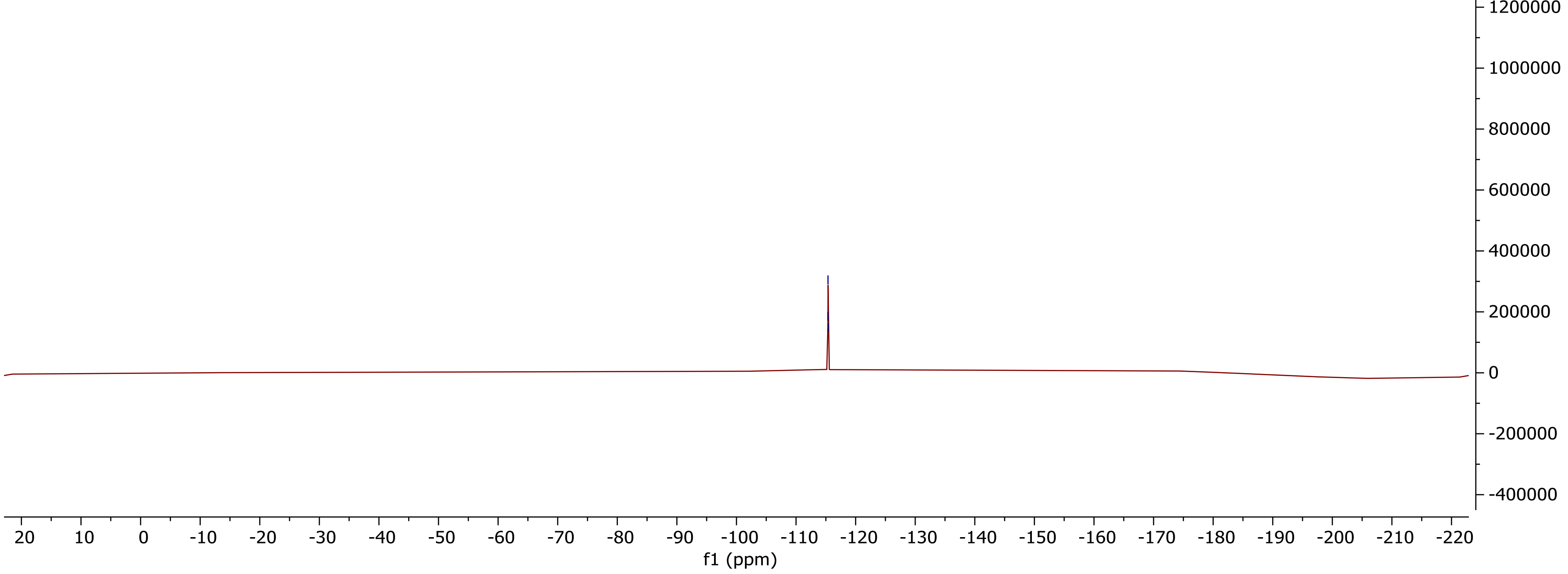

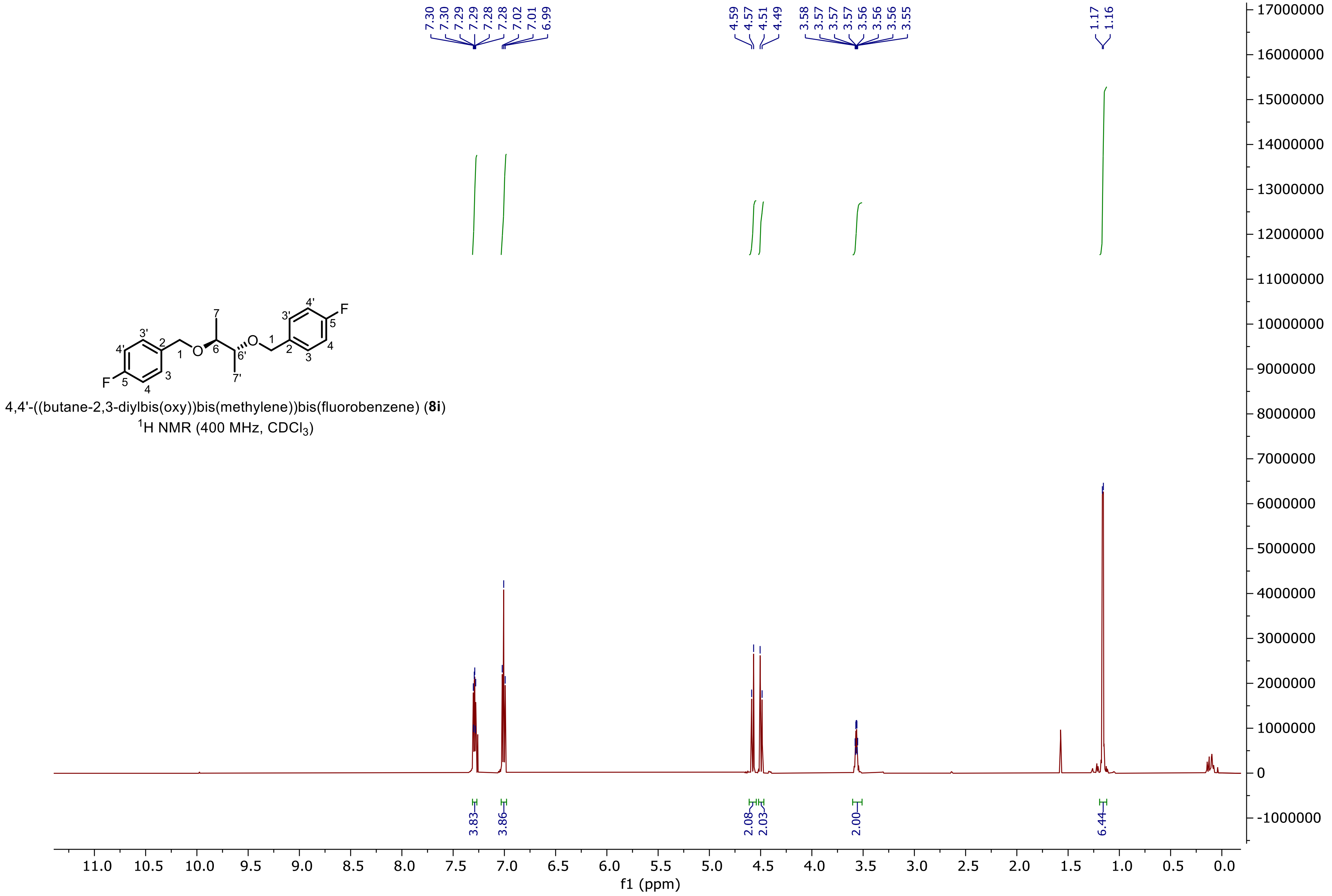

4,4'-((butane-2,3-diylbis(oxy))bis(methylene))bis(fluorobenzene) (**8i**)

^{1}H NMR (400 MHz, $CDCl_3$)

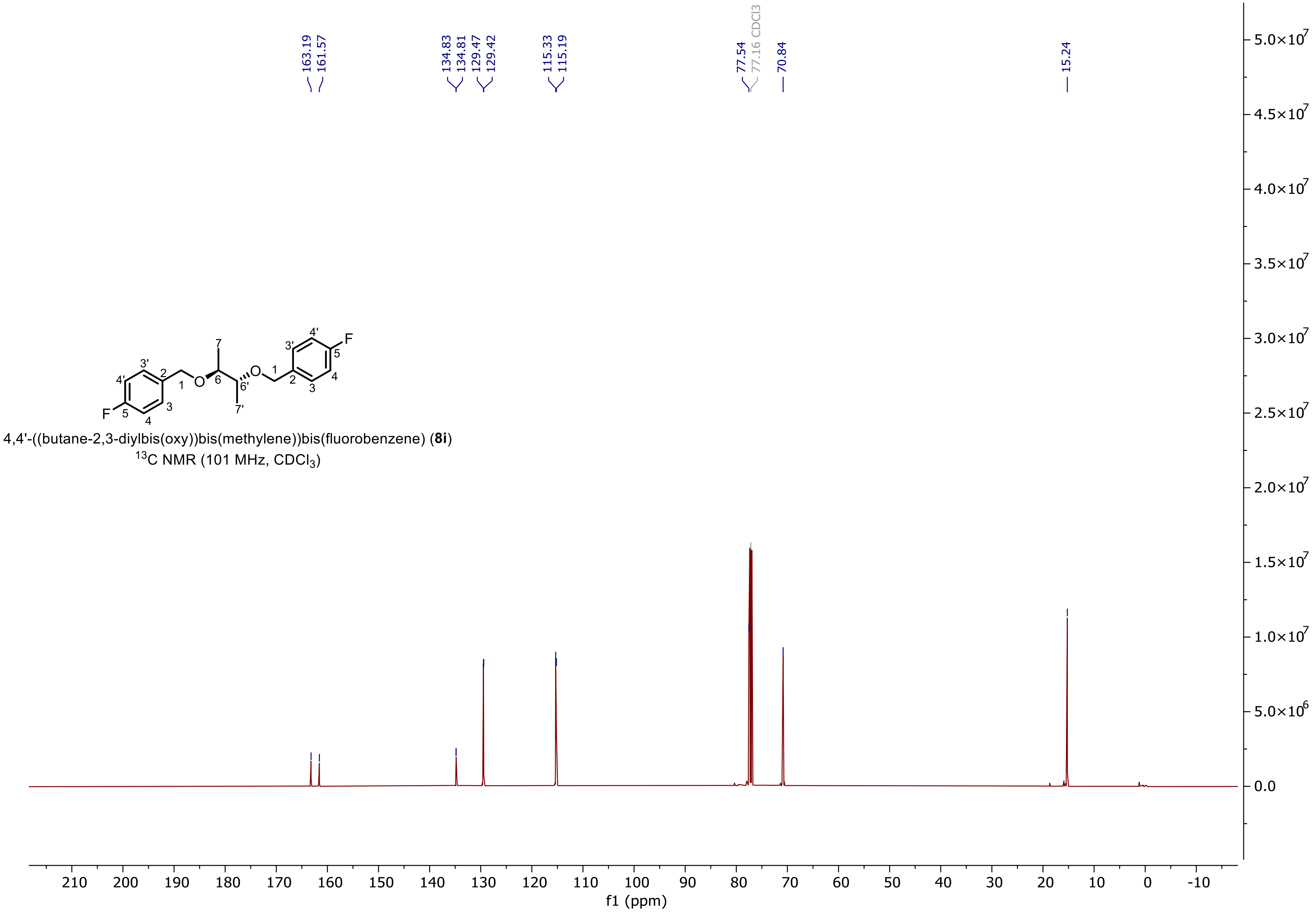

4,4'-((butane-2,3-diylbis(oxy))bis(methylene))bis(fluorobenzene) (**8i**)
^{13}C NMR (101 MHz, $CDCl_3$)

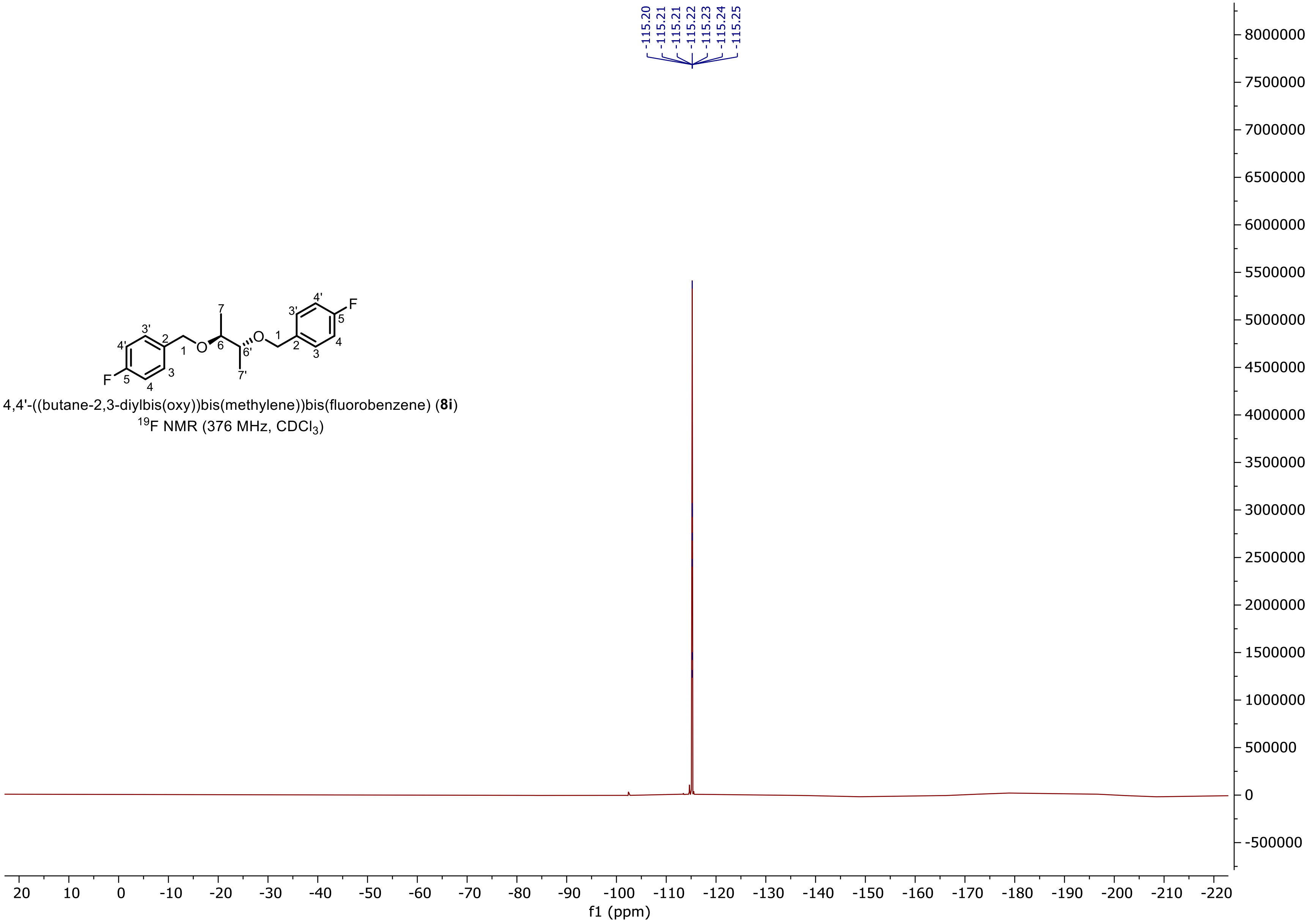

4,4'-((butane-2,3-diylbis(oxy))bis(methylene))bis(fluorobenzene) (**8i**)

^{19}F NMR (376 MHz, $CDCl_3$)

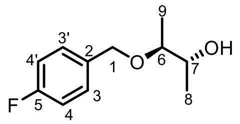

3-((4-fluorobenzyl)oxy)butan-2-ol (**8j**)
^{1}H NMR (400 MHz, $CDCl_3$)

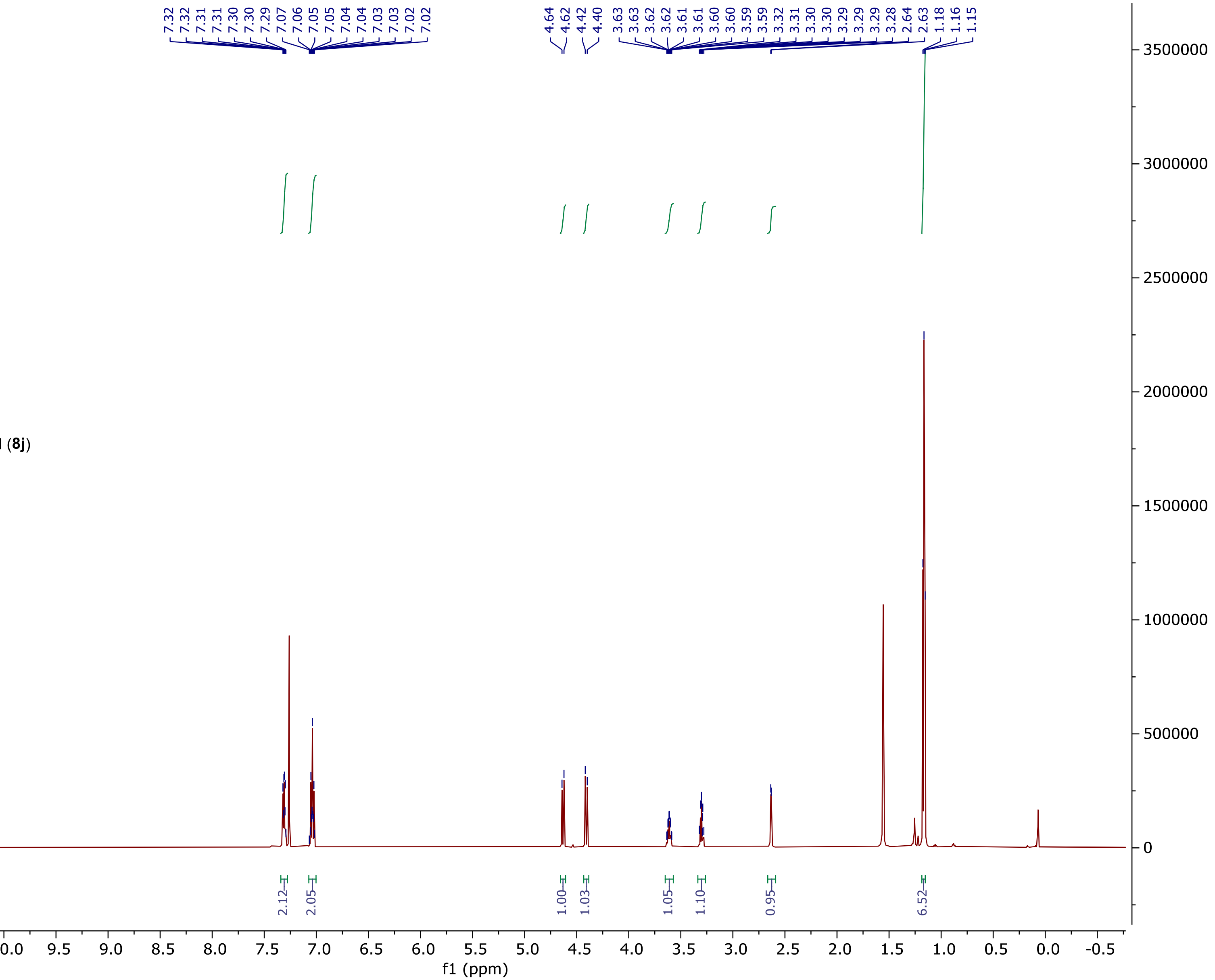

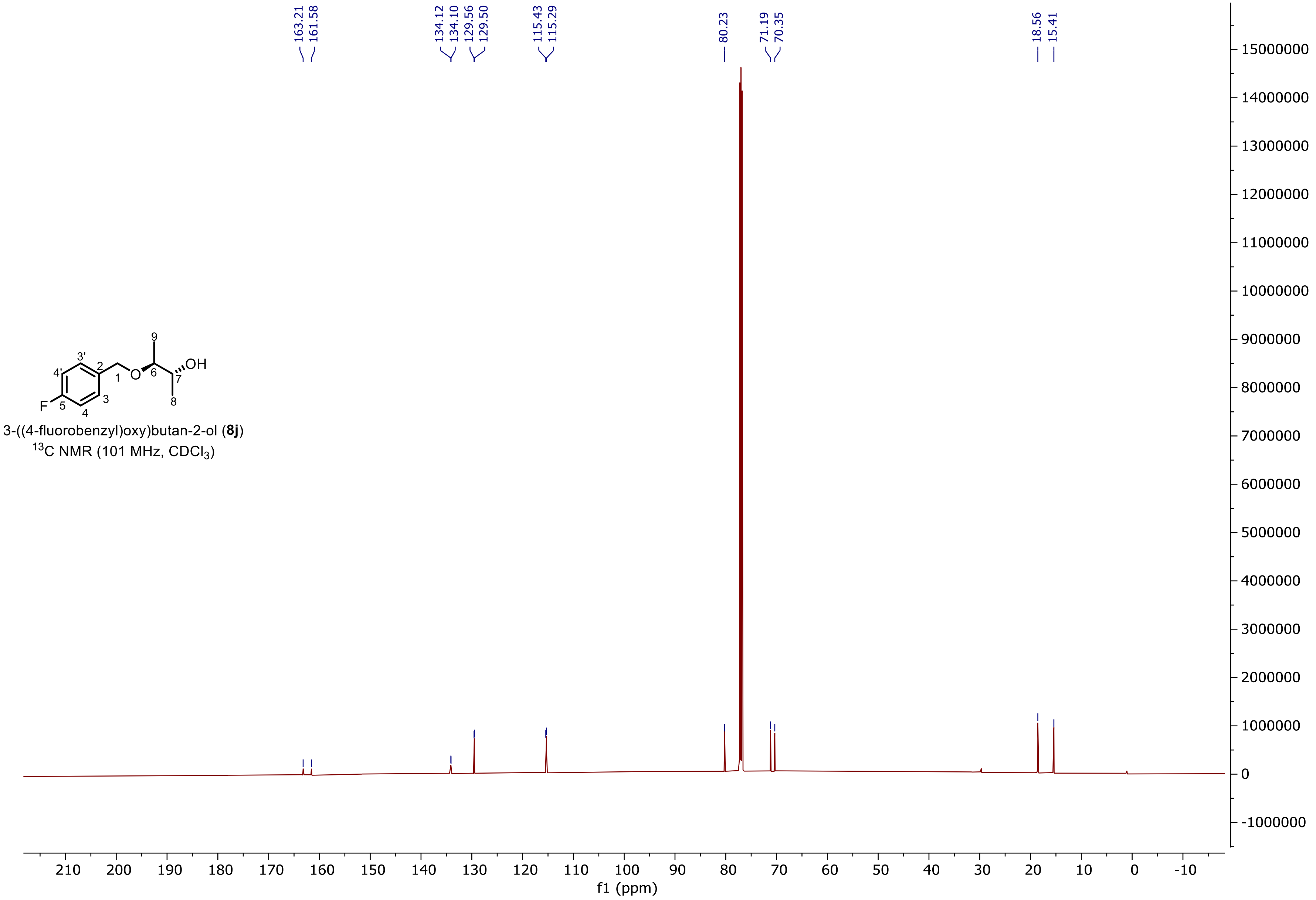

3-((4-fluorobenzyl)oxy)butan-2-ol (**8j**)

^{13}C NMR (101 MHz, $CDCl_3$)

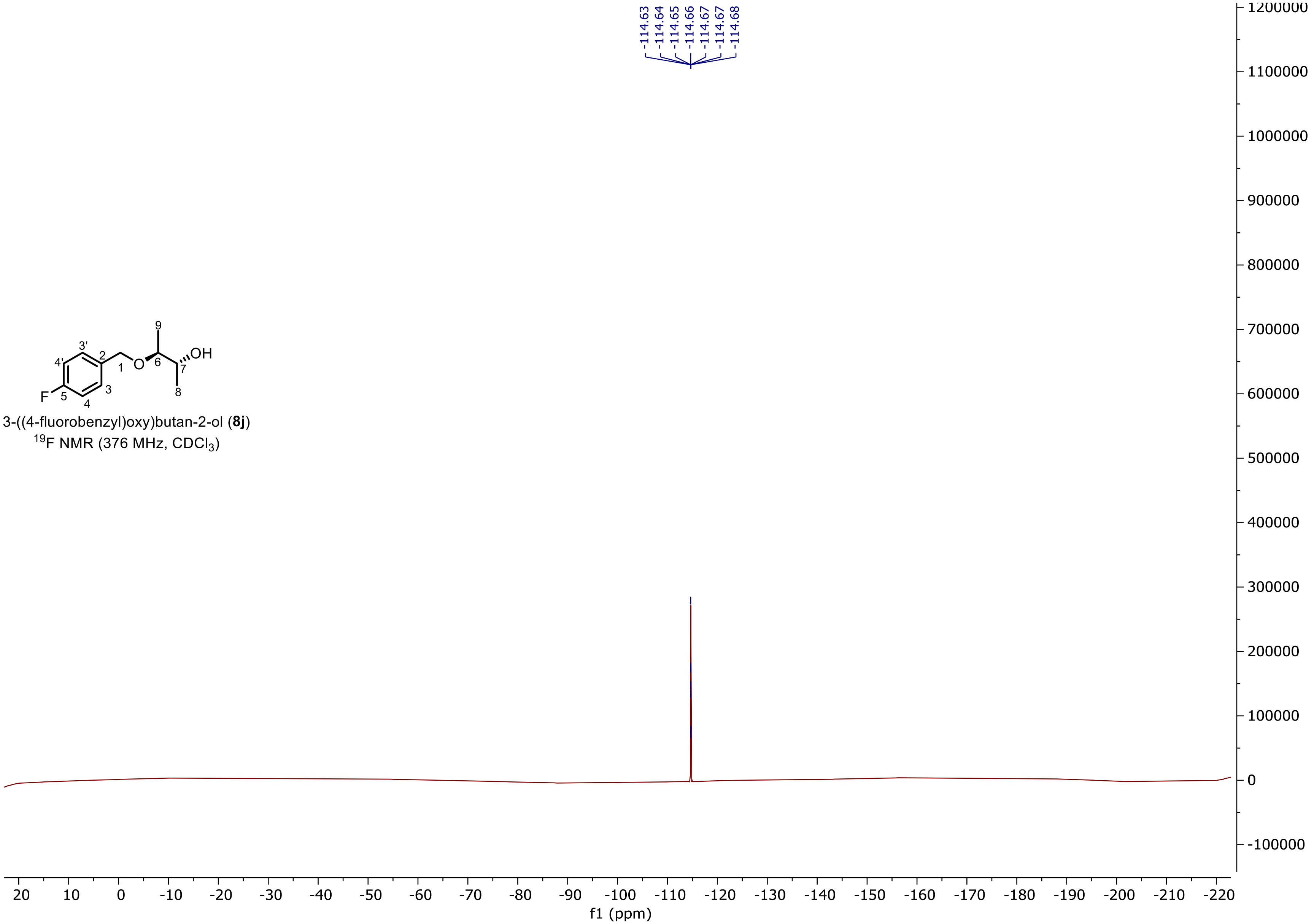

3-((4-fluorobenzyl)oxy)butan-2-ol (**8j**)

^{19}F NMR (376 MHz, $CDCl_3$)

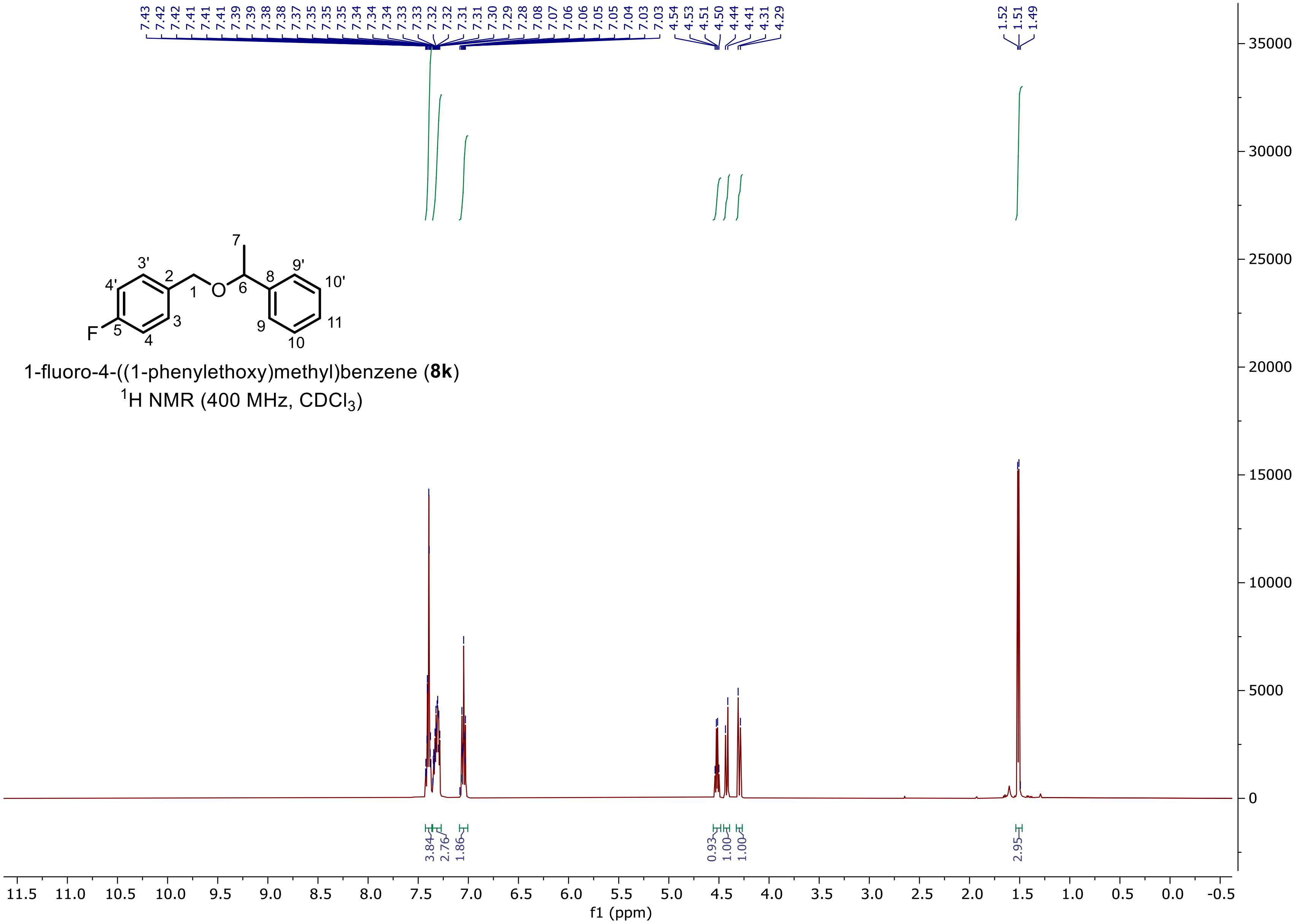
1-fluoro-4-((1-phenylethoxy)methyl)benzene (**8k**)
^{1}H NMR (400 MHz, $CDCl_3$)
7.43 7.42 7.42 7.41 7.41 7.39 7.39 7.38 7.38 7.37 7.35 7.35 7.35 7.34 7.34 7.34 7.33 7.33 7.32 7.32 7.31 7.31 7.30 7.29 7.28 7.08 7.07 7.06 7.06 7.05 7.05 7.04 7.03 4.54 4.53 4.51 4.50 4.44 4.41 4.31 4.29
1.52 1.51 1.49
3.84 2.76 1.86 0.93 1.00 1.00 2.95
f1 (ppm)

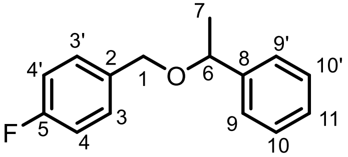

1-fluoro-4-((1-phenylethoxy)methyl)benzene (**8k**)
^{13}C NMR (101 MHz, $CDCl_3$)

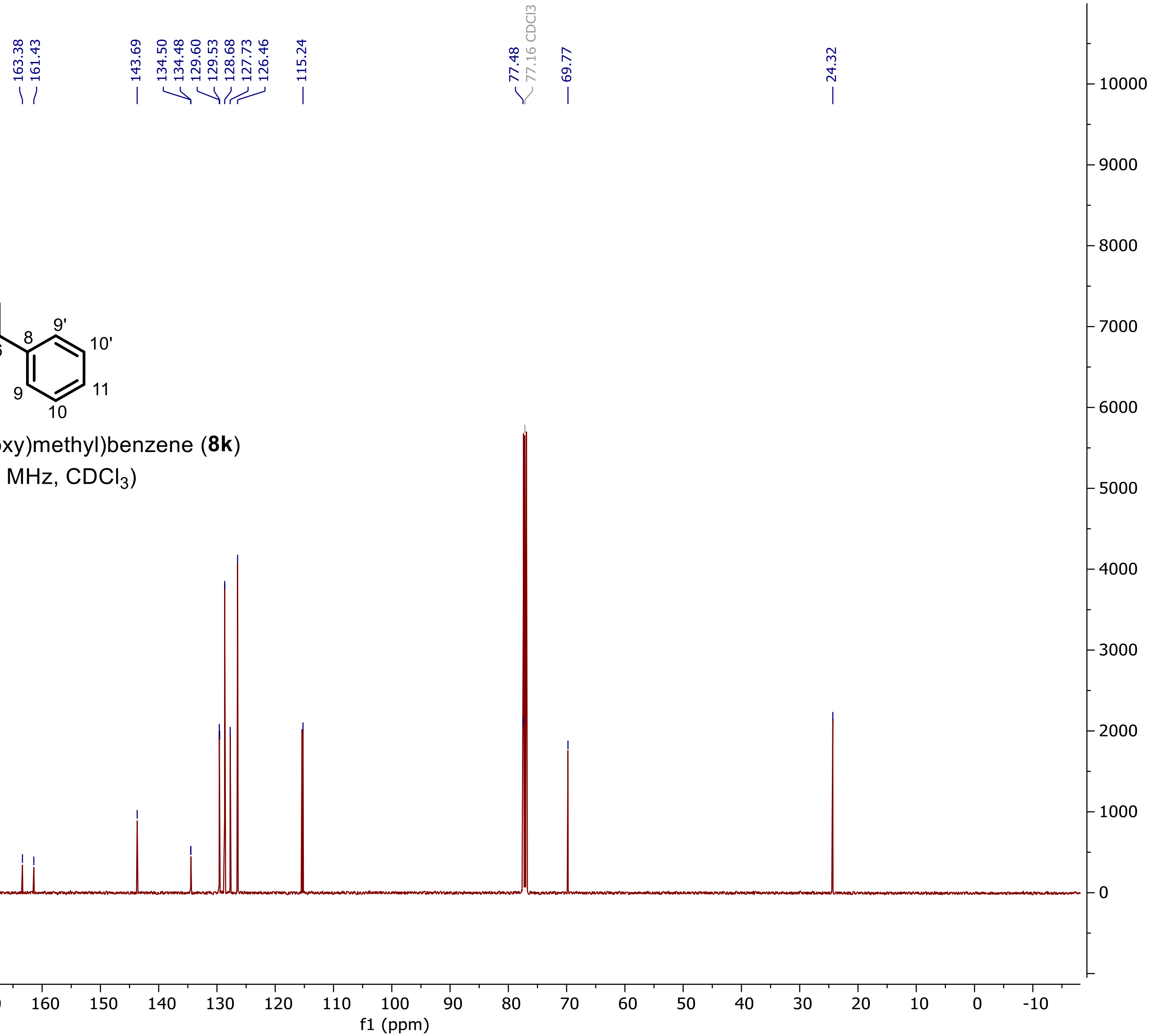

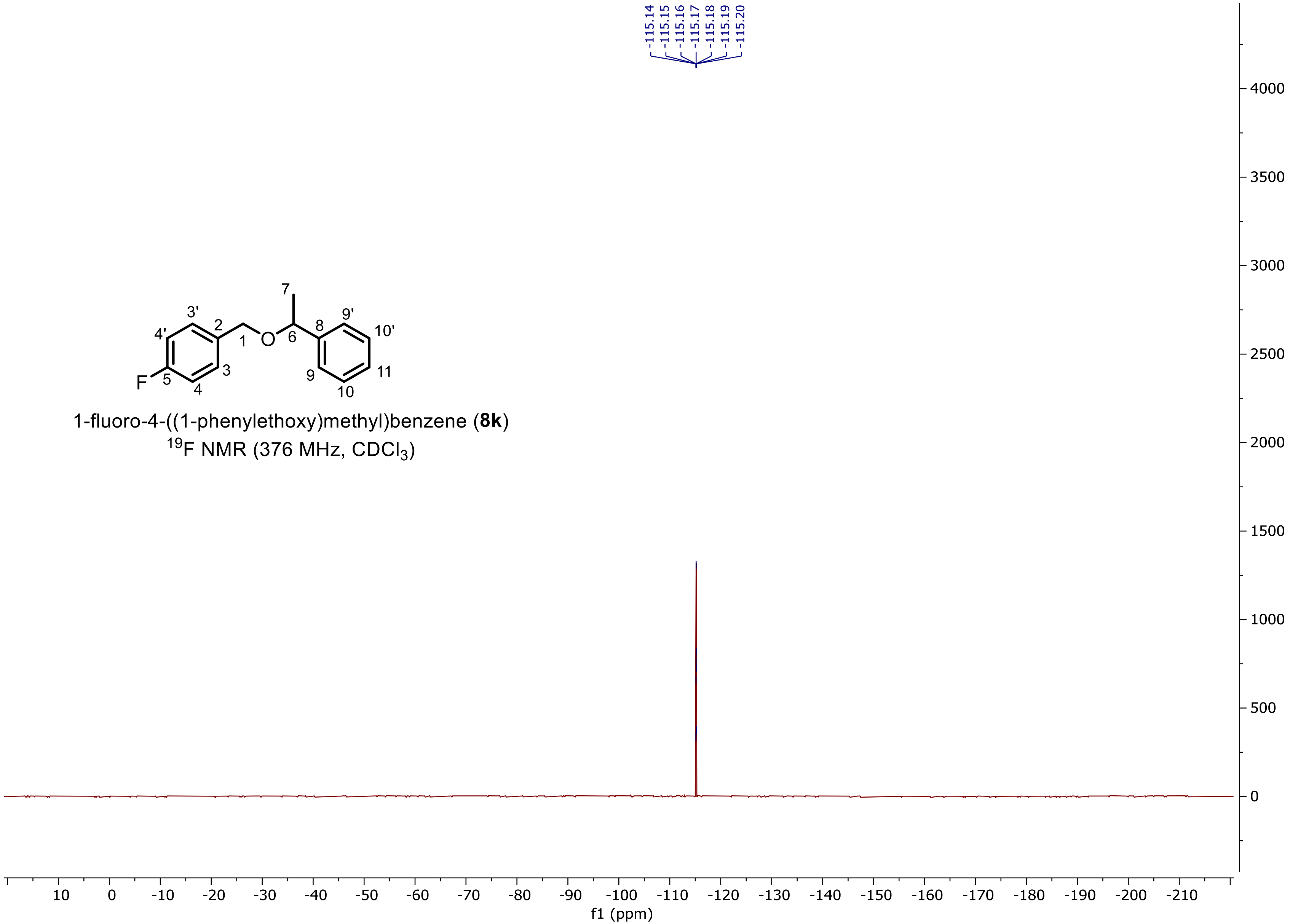
-115.14
-115.15
-115.16
-115.17
-115.18
-115.19
-115.20
1-fluoro-4-((1-phenylethoxy)methyl)benzene (**8k**)
^{19}F NMR (376 MHz, $CDCl_3$)
f1 (ppm)

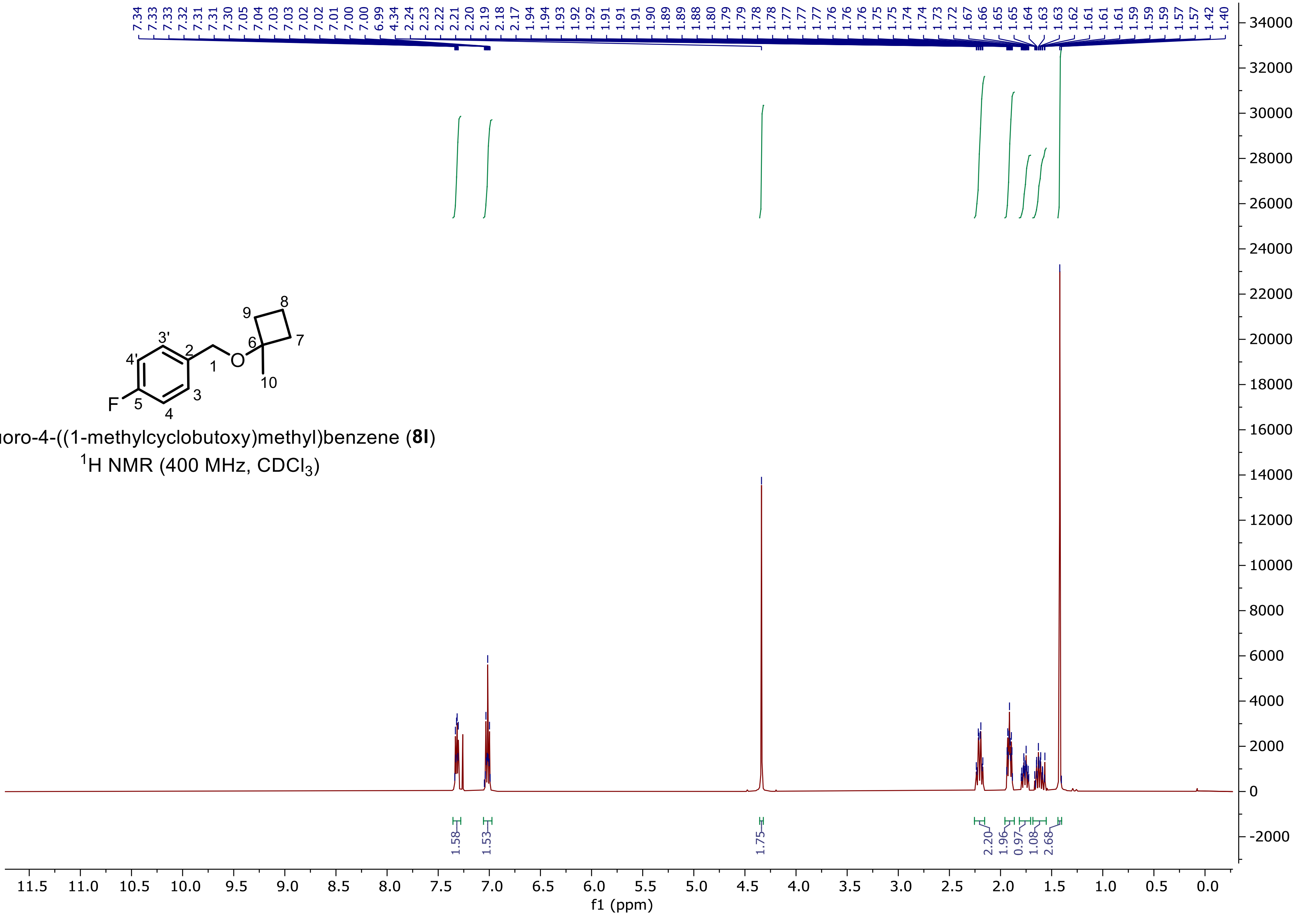

1-fluoro-4-((1-methylcyclobutoxy)methyl)benzene (**8l**)
^{1}H NMR (400 MHz, $CDCl_3$)

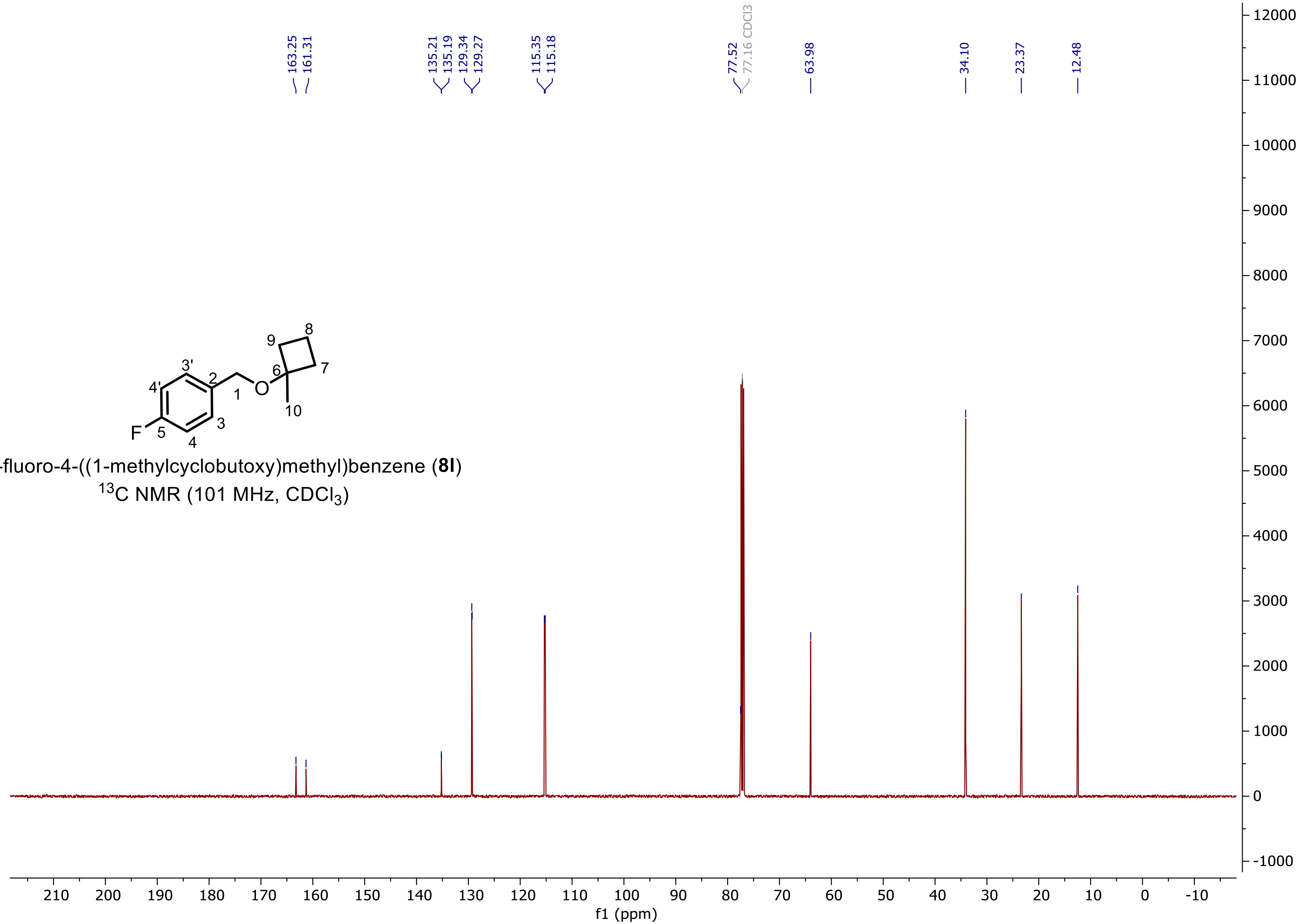

1-fluoro-4-((1-methylcyclobutoxy)methyl)benzene (**8l**)

^{13}C NMR (101 MHz, $CDCl_3$)

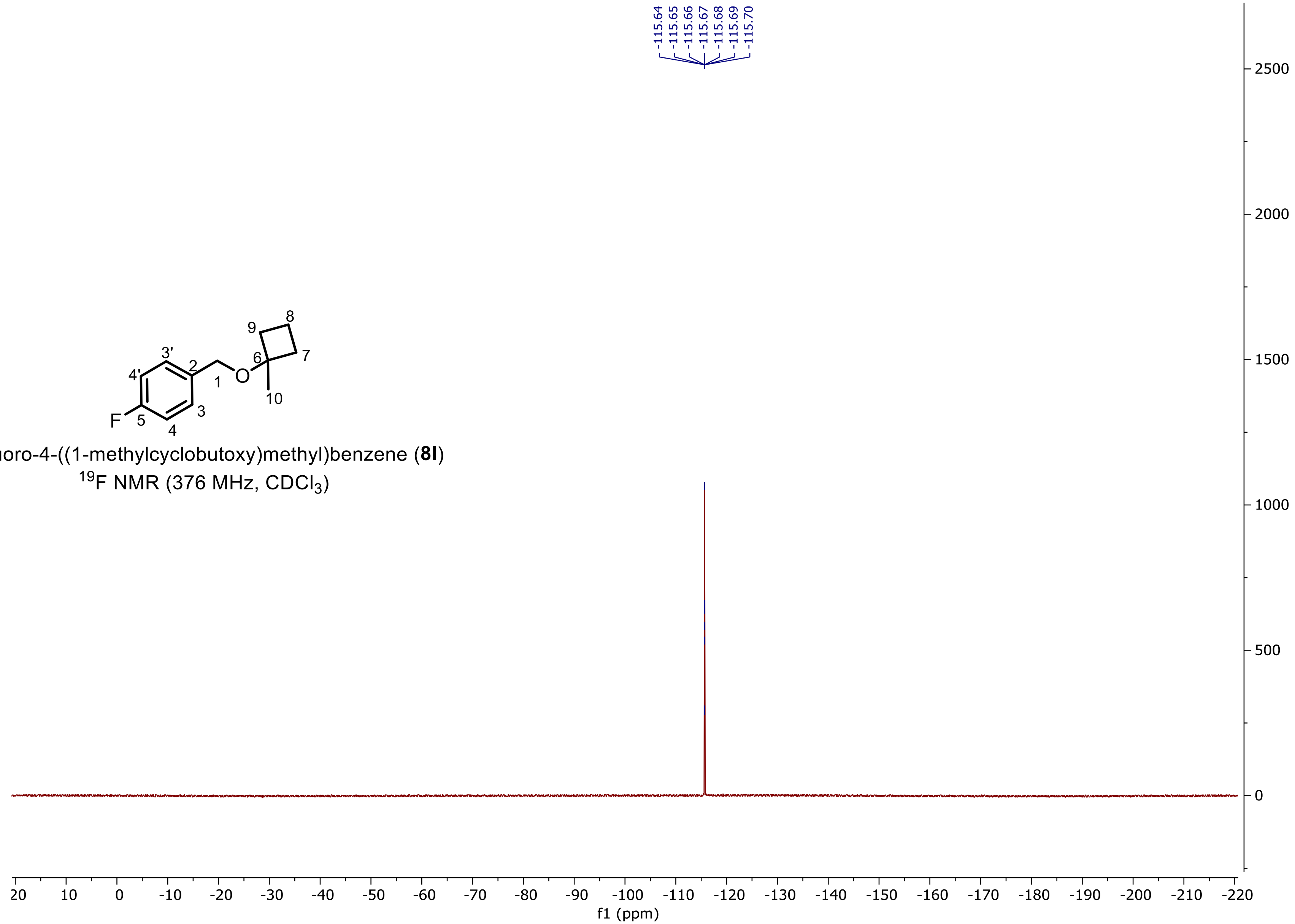

1-fluoro-4-((1-methylcyclobutoxy)methyl)benzene (**8l**)

^{19}F NMR (376 MHz, $CDCl_3$)

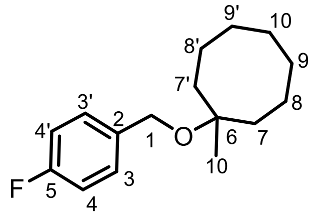

1-((4-fluorobenzyl)oxy)-1-methylcyclooctane (**8m**)

^{1}H NMR (400 MHz, $CDCl_3$)

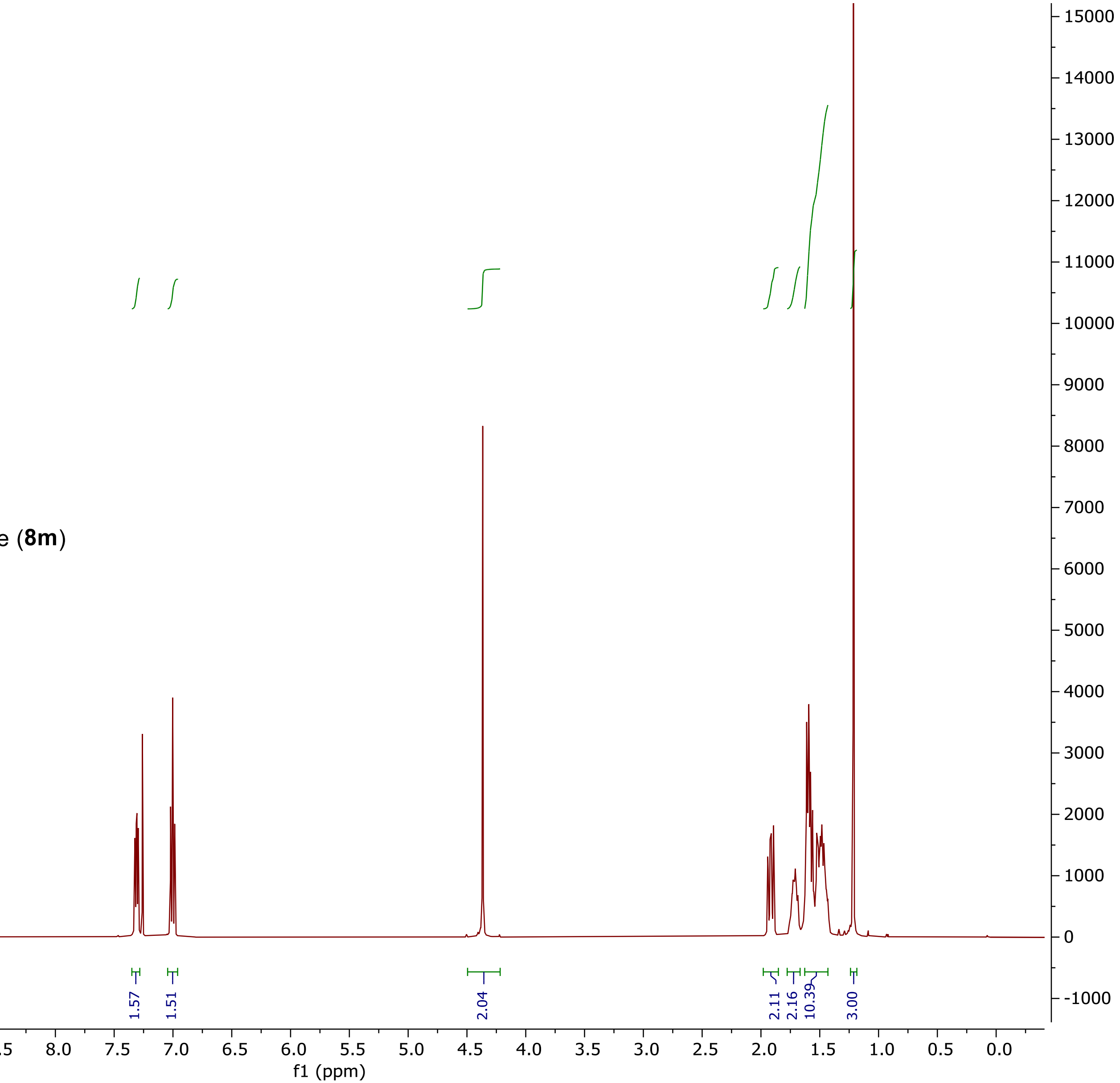

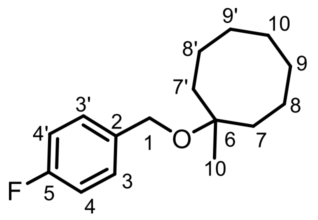

1-((4-fluorobenzyl)oxy)-1-methylcyclooctane (**8m**)
^{13}C NMR (101 MHz, $CDCl_3$)

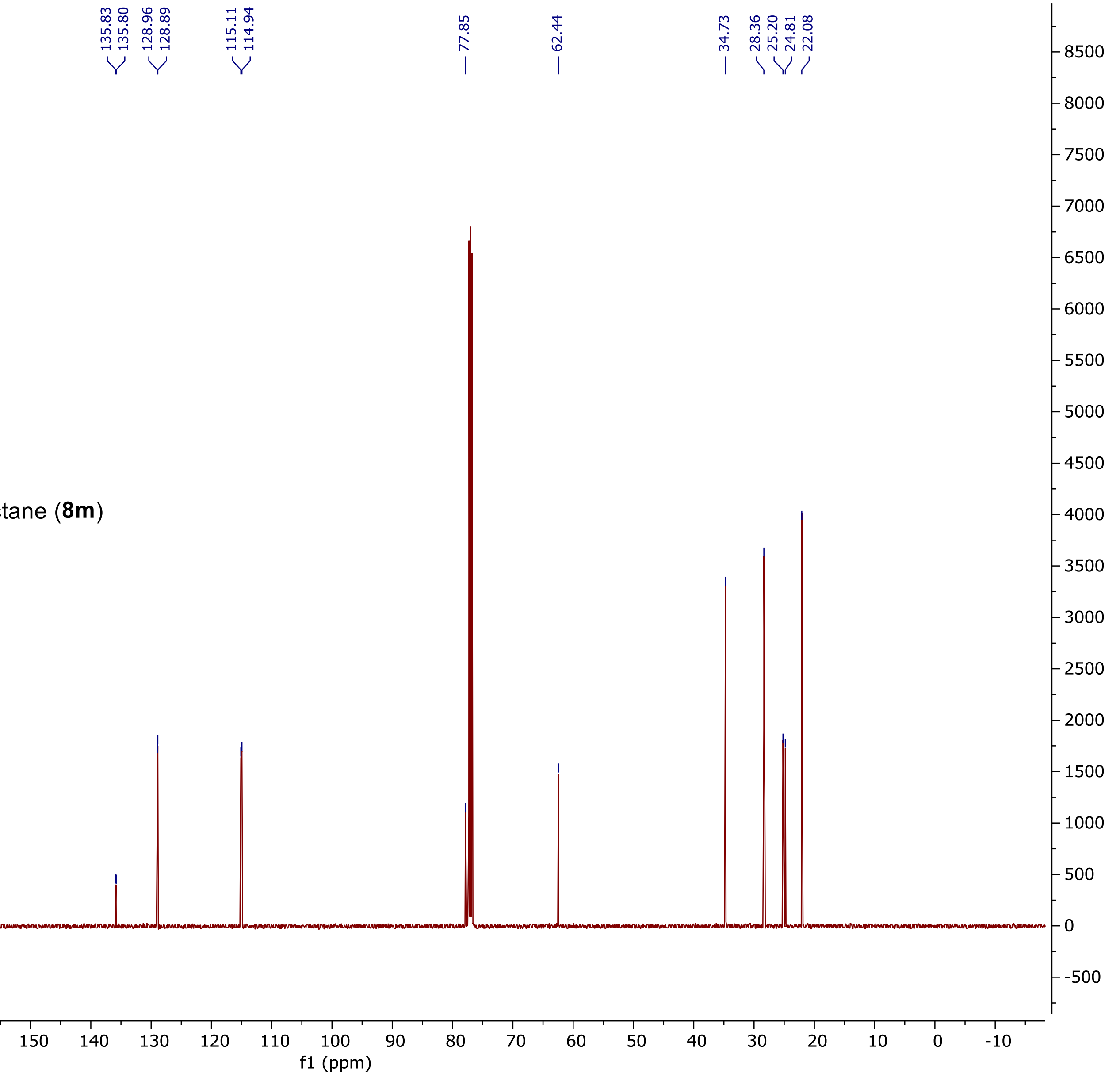

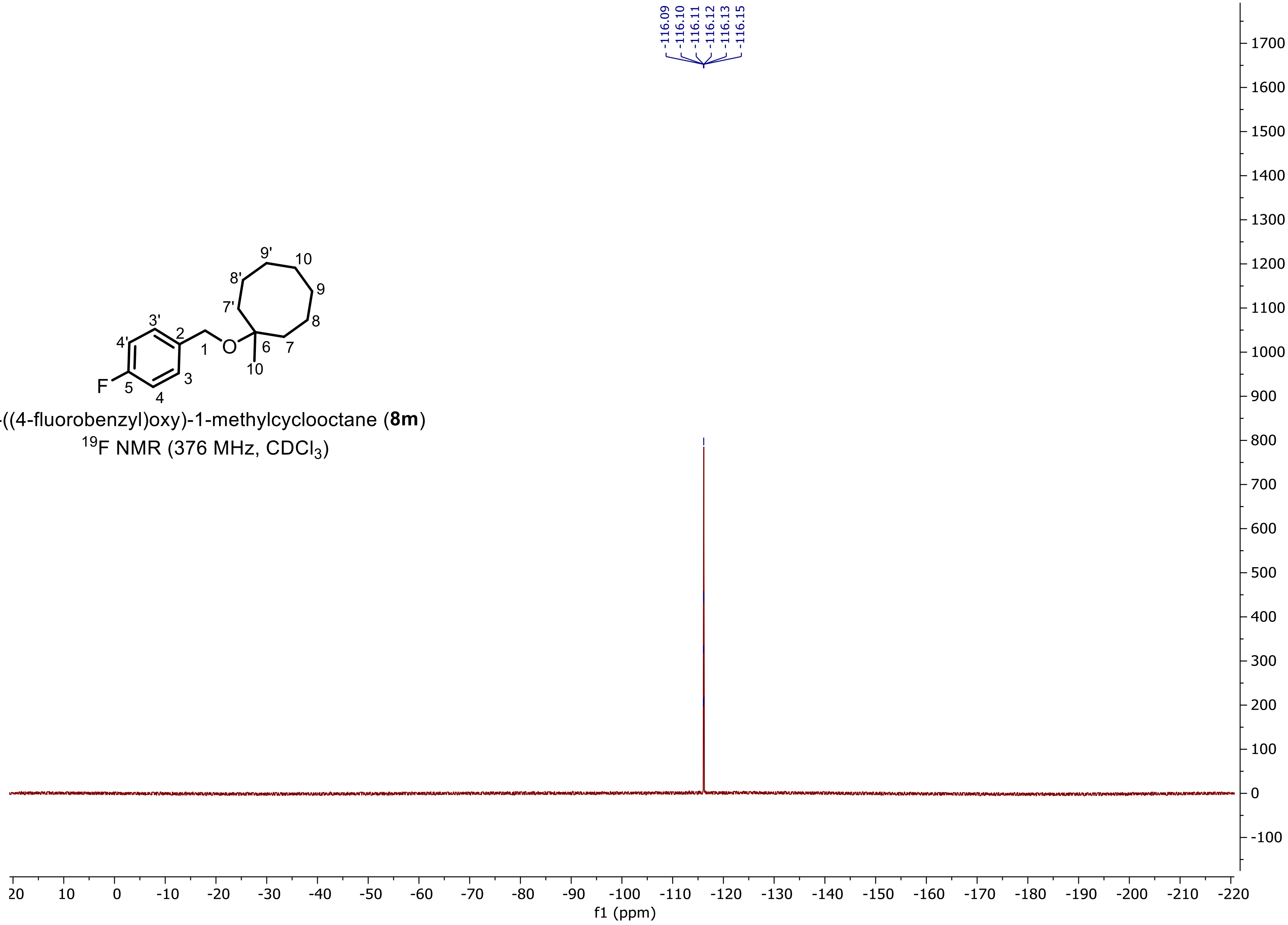

1-((4-fluorobenzyl)oxy)-1-methylcyclooctane (**8m**)

^{19}F NMR (376 MHz, $CDCl_3$)

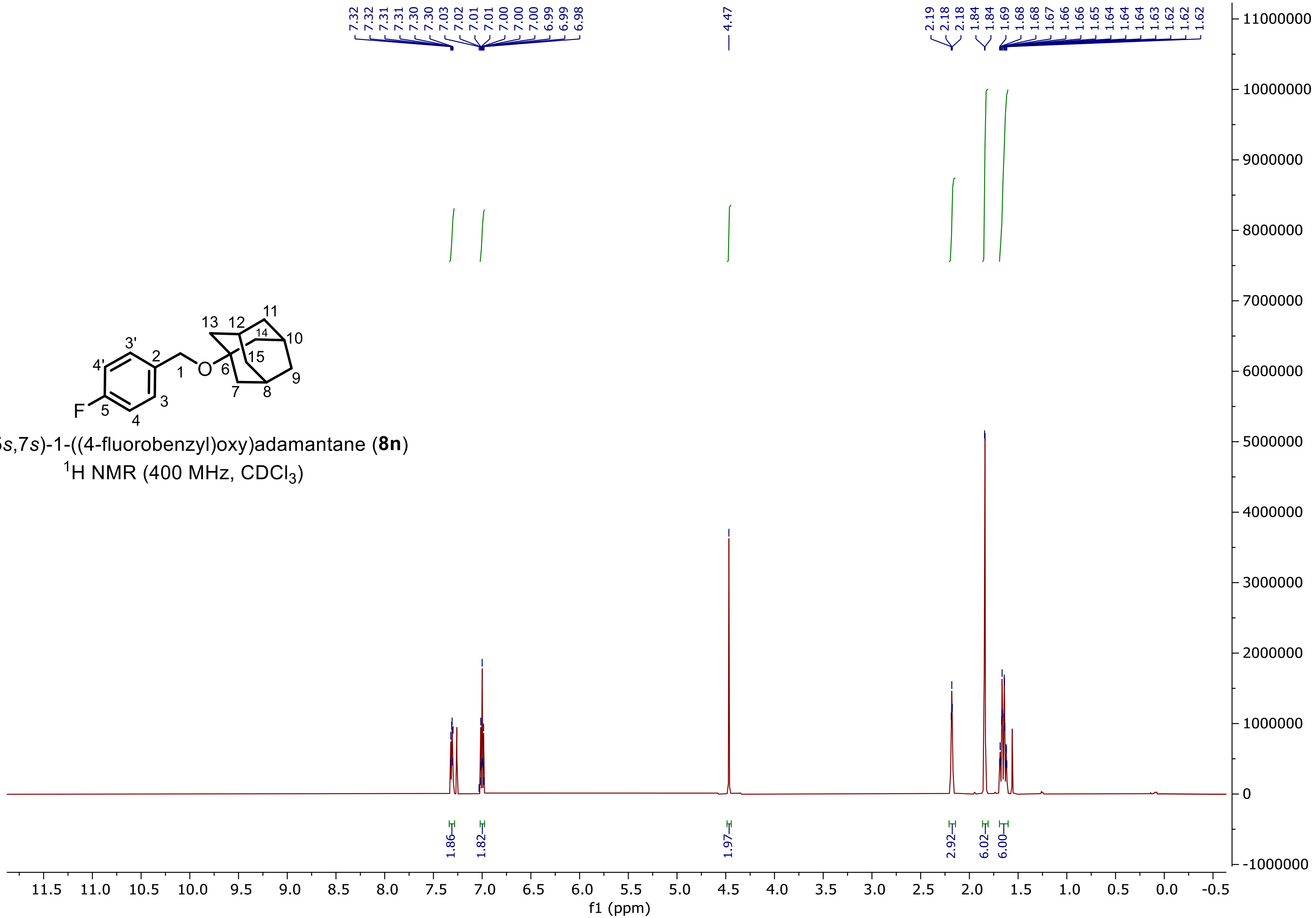

(3*s*,5*s*,7*s*)-1-((4-fluorobenzyl)oxy)adamantane (**8n**)
^{1}H NMR (400 MHz, $CDCl_3$)

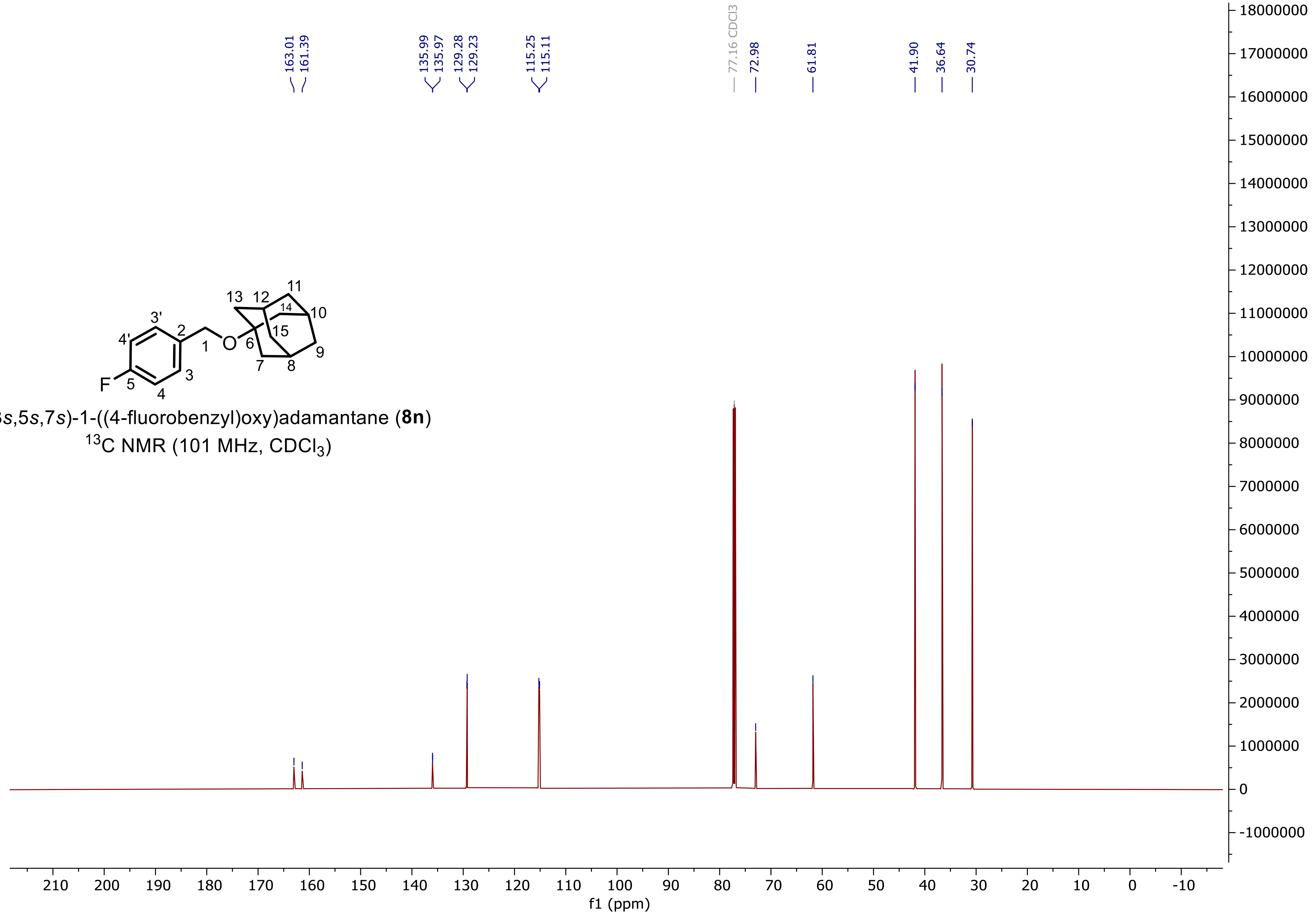

(3*s*,5*s*,7*s*)-1-((4-fluorobenzyl)oxy)adamantane (**8n**)

^{13}C NMR (101 MHz, $CDCl_3$)

(3*s*,5*s*,7*s*)-1-((4-fluorobenzyl)oxy)adamantane (**8n**)

^{19}F NMR (376 MHz, $CDCl_3$)

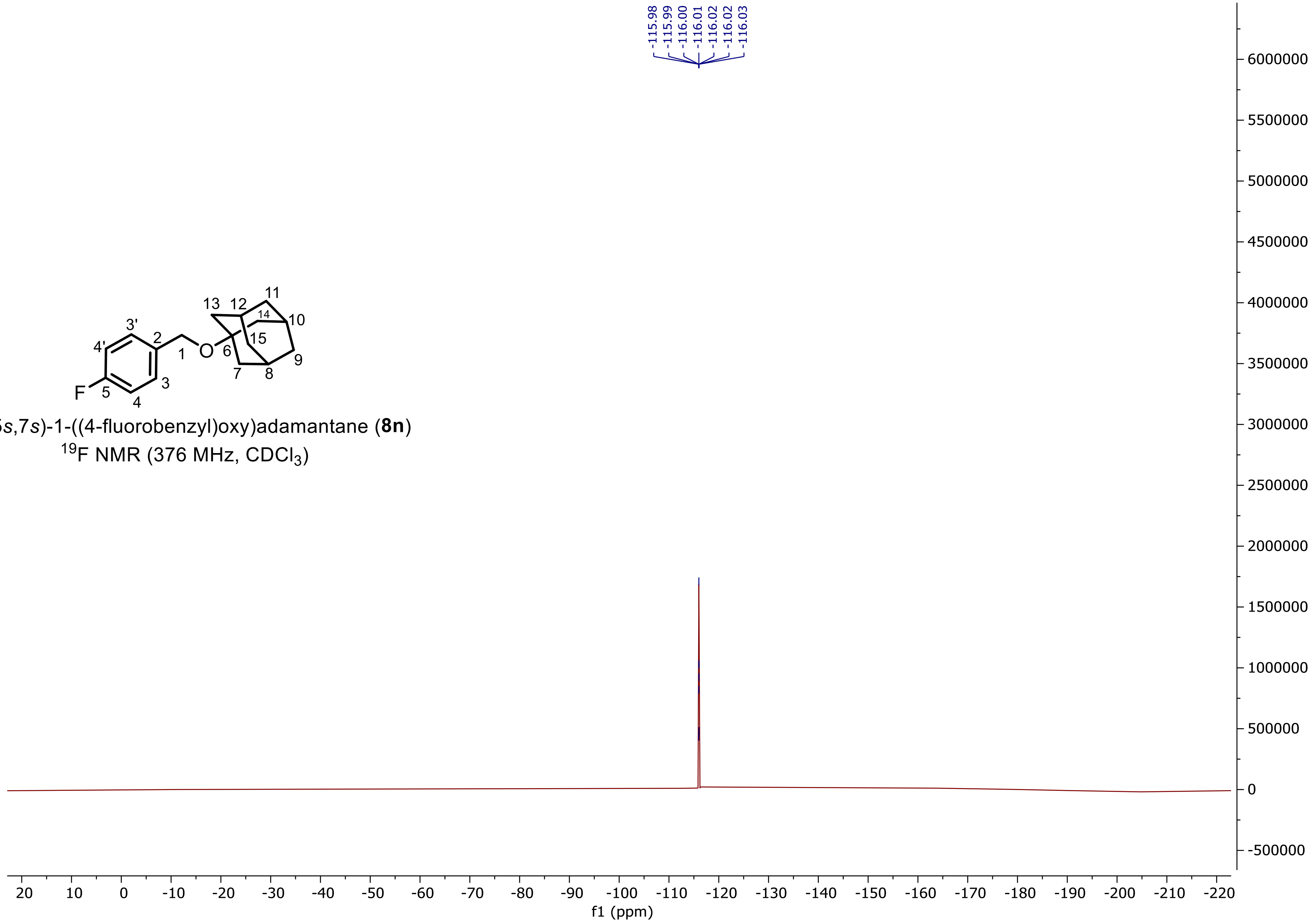

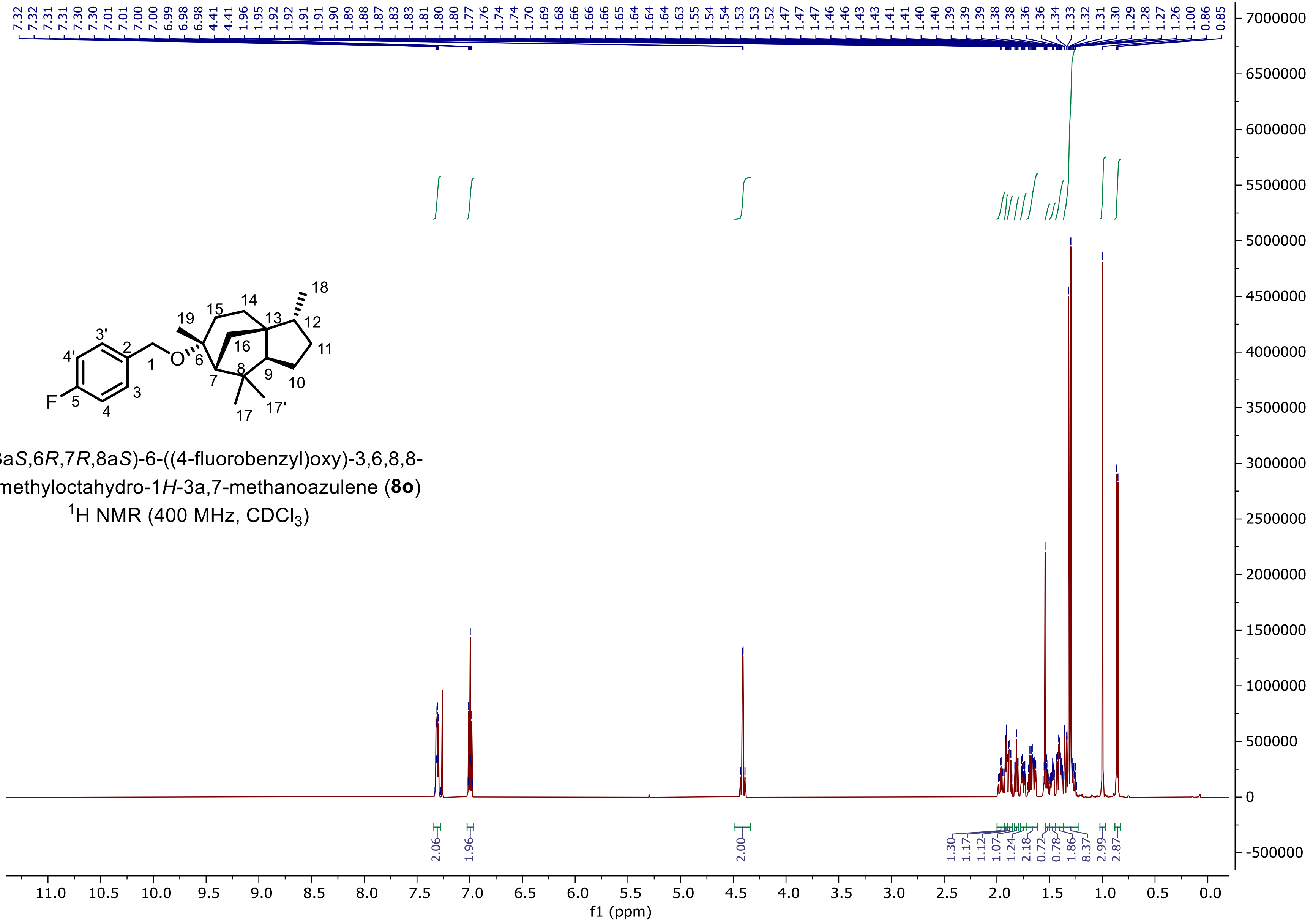

(3*R*,3a*S*,6*R*,7*R*,8a*S*)-6-((4-fluorobenzyl)oxy)-3,6,8,8-tetramethyloctahydro-1*H*-3a,7-methanoazulene (**8o**)
^{1}H NMR (400 MHz, $CDCl_3$)

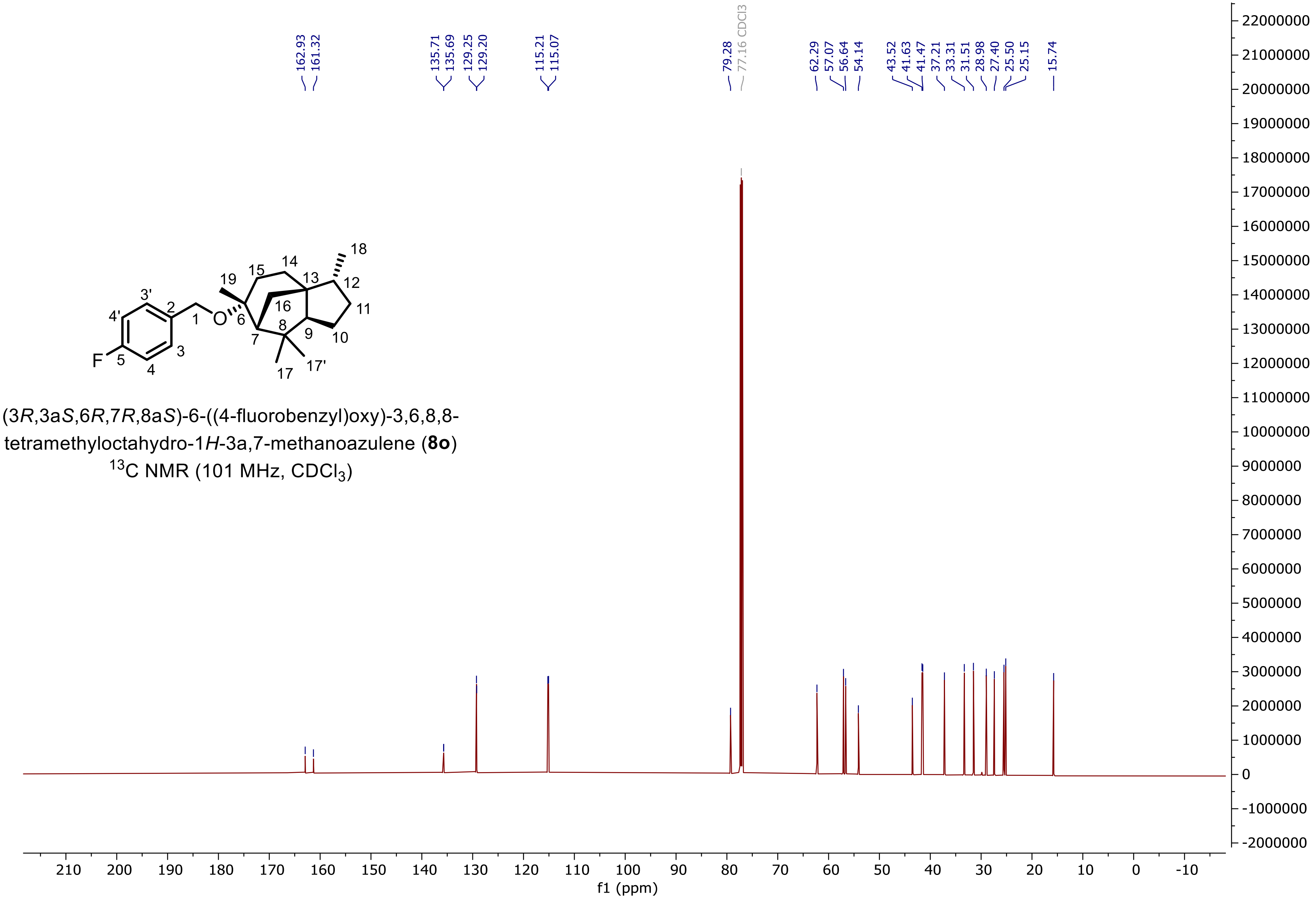

(3*R*,3a*S*,6*R*,7*R*,8a*S*)-6-((4-fluorobenzyl)oxy)-3,6,8,8-tetramethyloctahydro-1*H*-3a,7-methanoazulene (**8o**)

^{13}C NMR (101 MHz, $CDCl_3$)

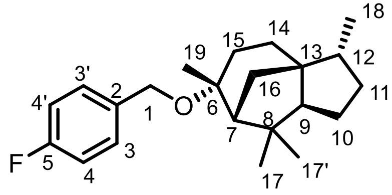

(3*R*,3a*S*,6*R*,7*R*,8a*S*)-6-((4-fluorobenzyl)oxy)-3,6,8,8-tetramethyloctahydro-1*H*-3a,7-methanoazulene (**8o**)
^{19}F NMR (376 MHz, $CDCl_3$)

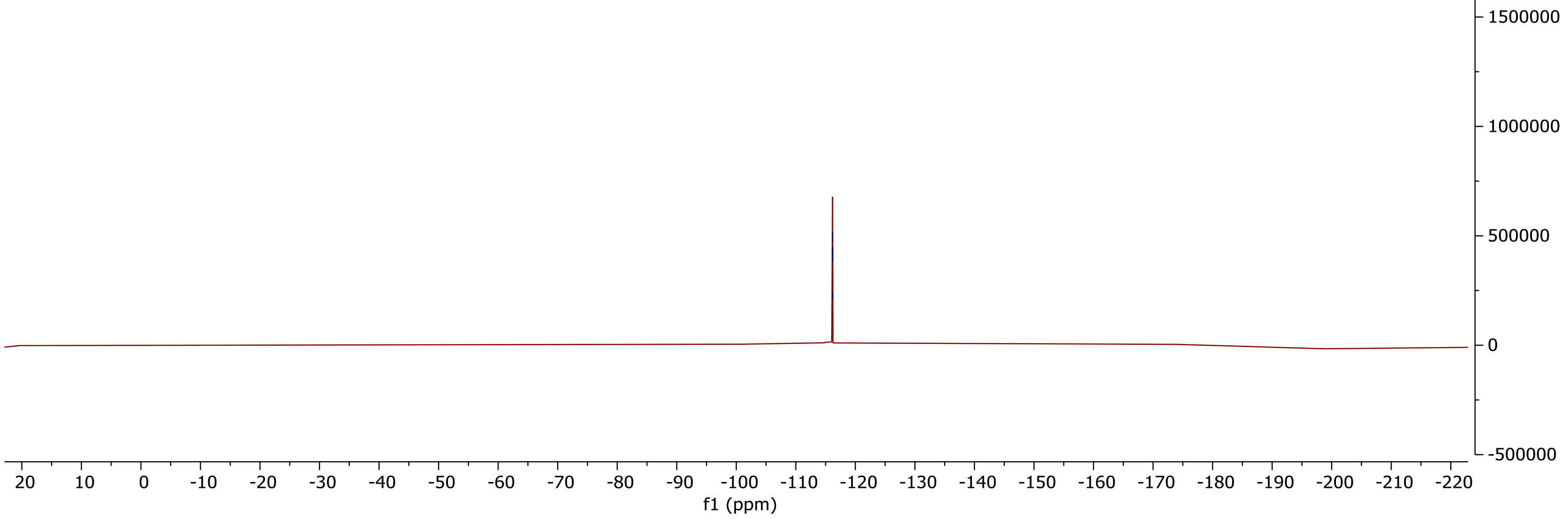

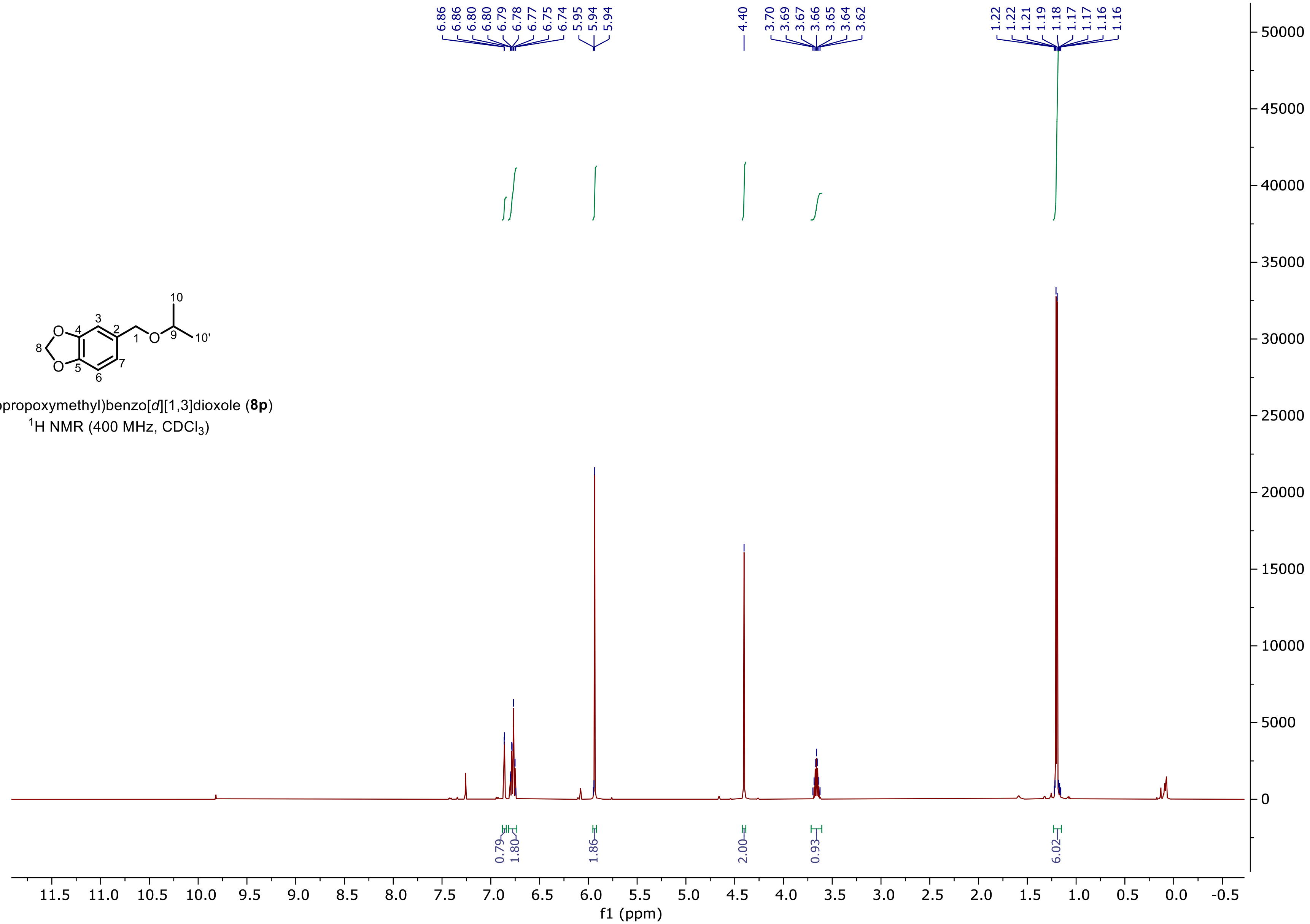

5-(isopropoxymethyl)benzo[*d*][1,3]dioxole (**8p**)
^{1}H NMR (400 MHz, $CDCl_3$)

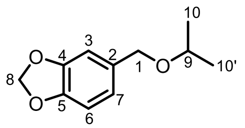

5-(isopropoxymethyl)benzo[*d*][1,3]dioxole (**8p**)
^{13}C NMR (101 MHz, CDCl$_3$)

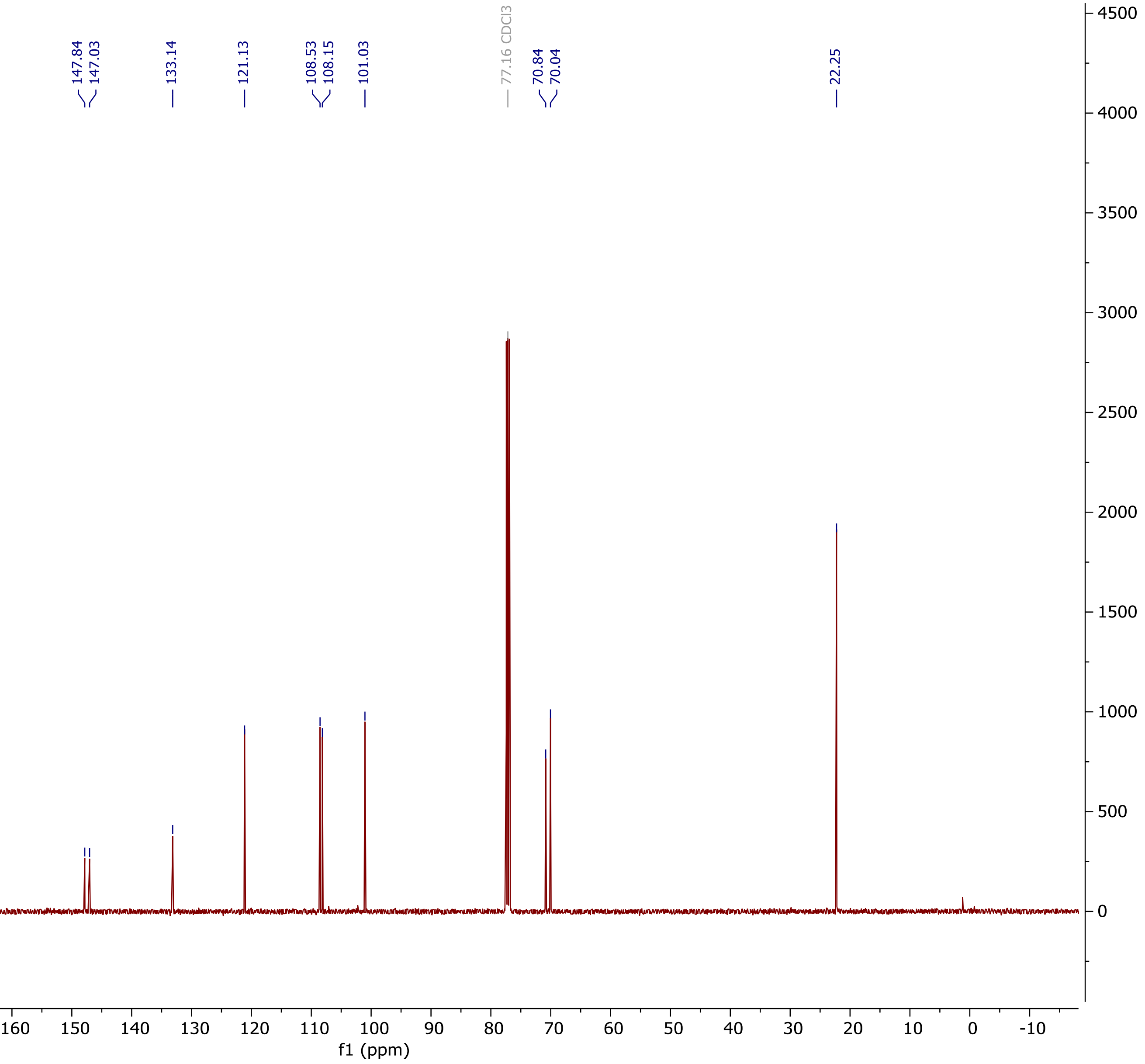

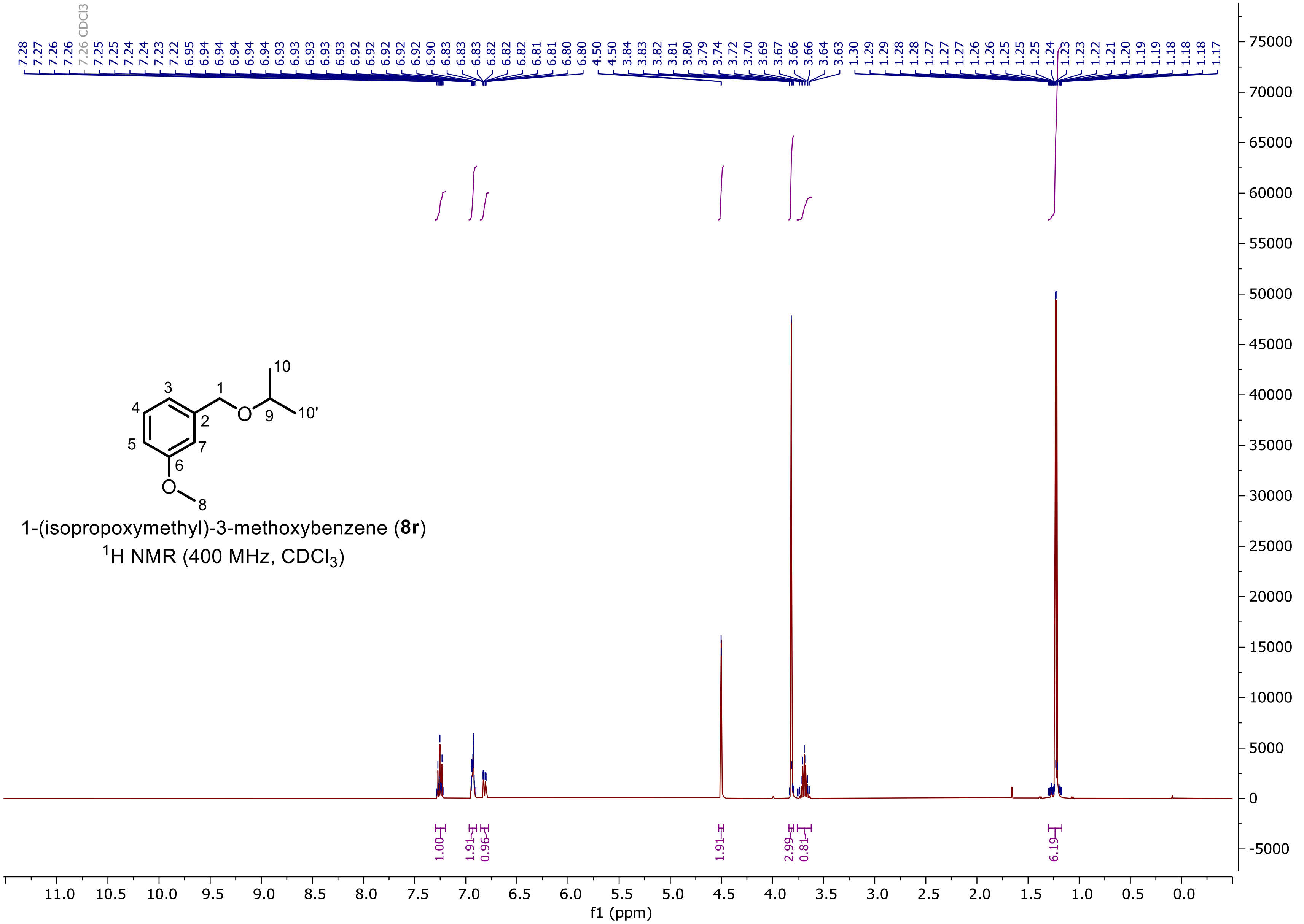

1-(isopropoxymethyl)-3-methoxybenzene (**8r**)

^{1}H NMR (400 MHz, $CDCl_3$)

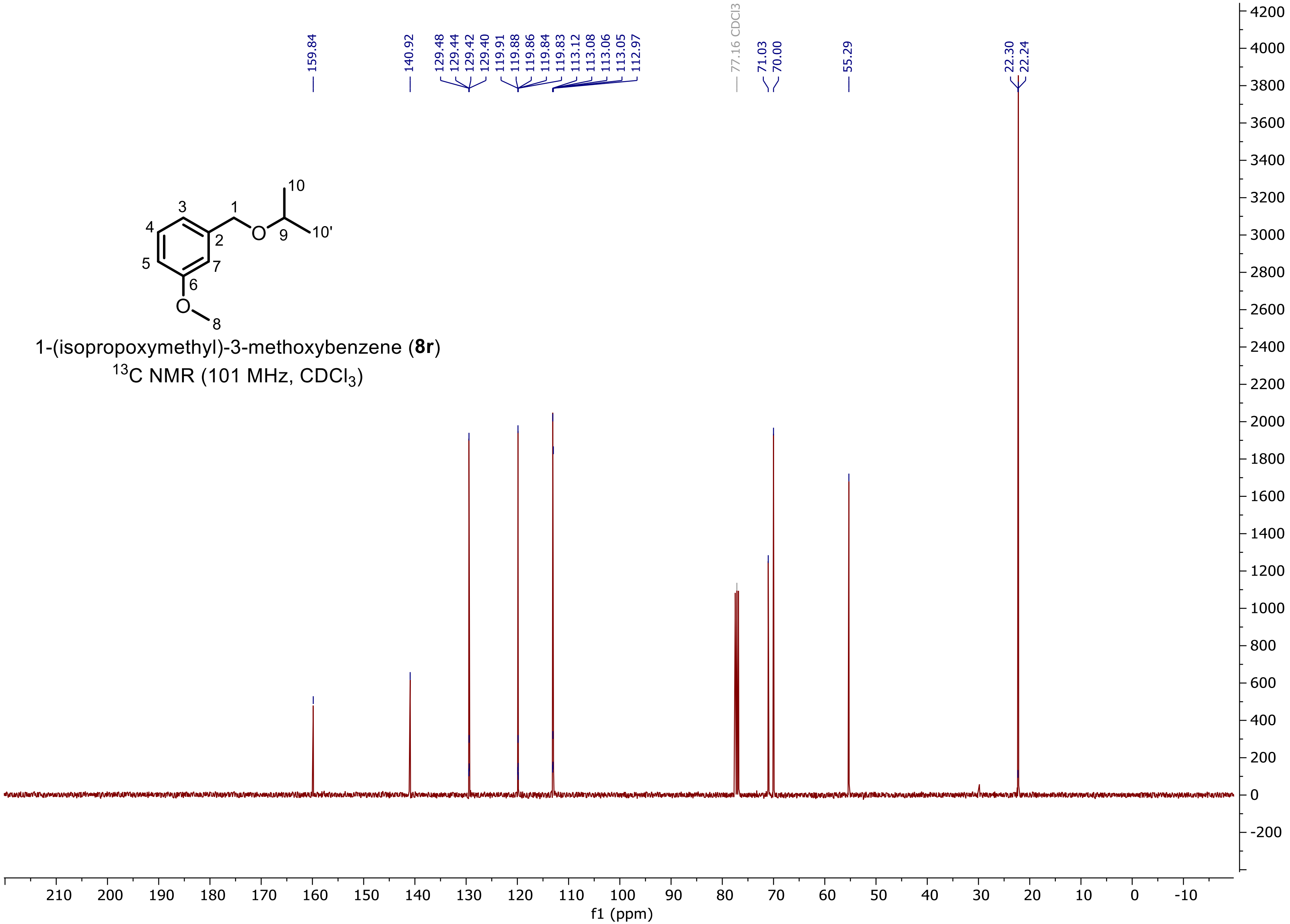
159.84
140.92
129.48
129.44
129.42
129.40
119.91
119.88
119.86
119.84
119.83
113.12
113.08
113.06
113.05
112.97
77.16 CDCl3
71.03
70.00
55.29
22.30
22.24
1-(isopropoxymethyl)-3-methoxybenzene (8r)
13C NMR (101 MHz, CDCl3)
f1 (ppm)

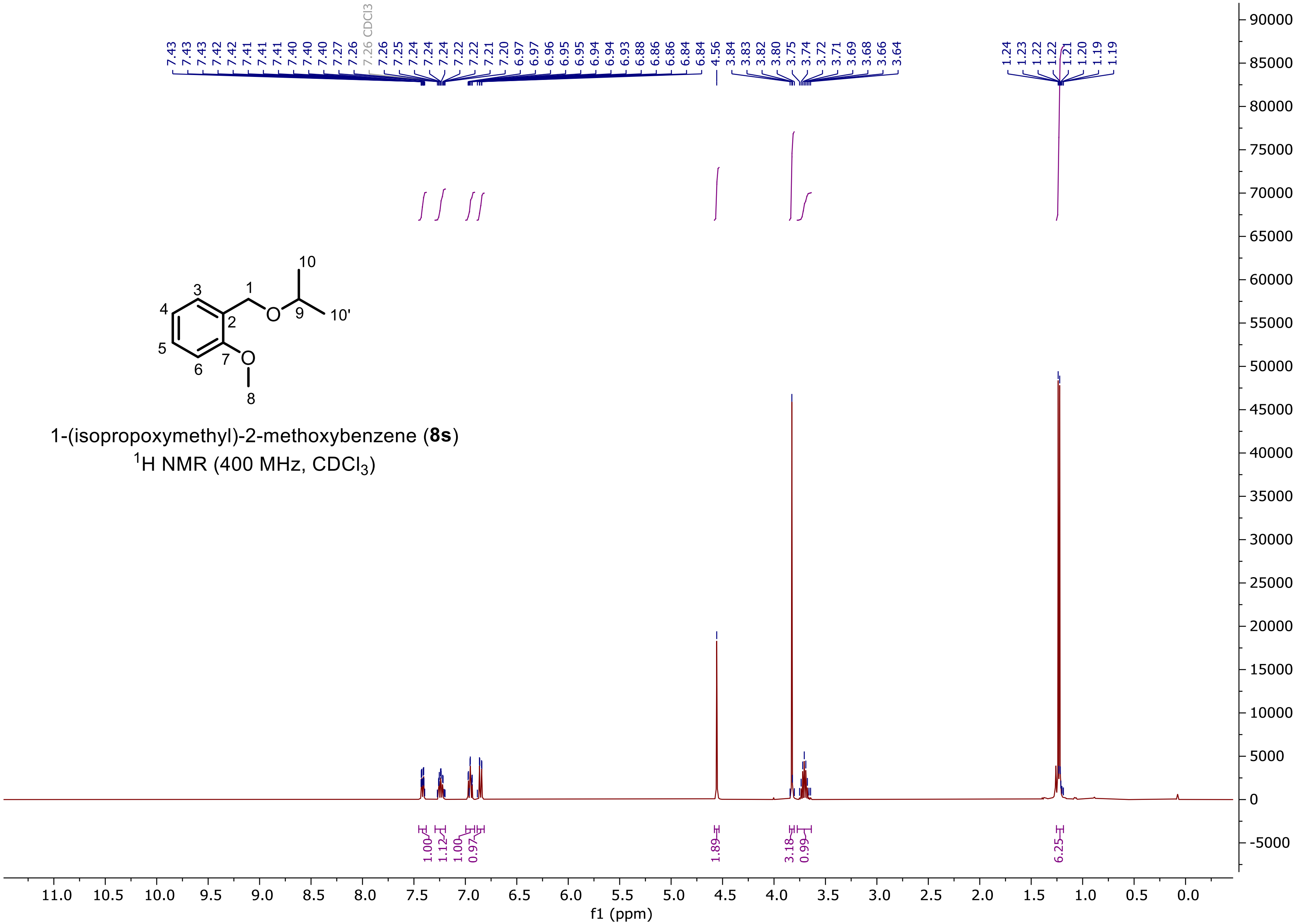

1-(isopropoxymethyl)-2-methoxybenzene (**8s**)

^{1}H NMR (400 MHz, $CDCl_3$)

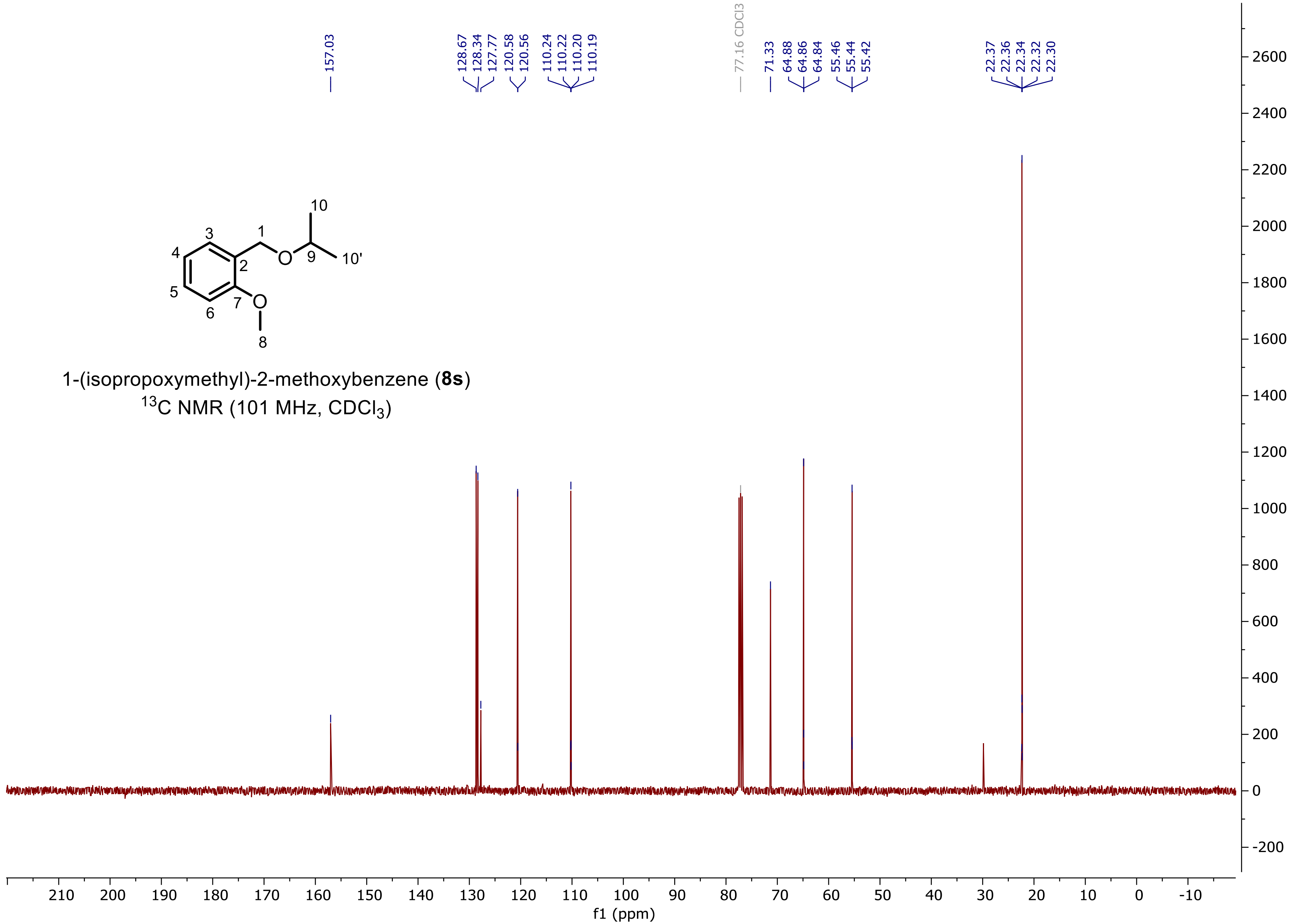

1-(isopropoxymethyl)-2-methoxybenzene (**8s**)

^{13}C NMR (101 MHz, $CDCl_3$)

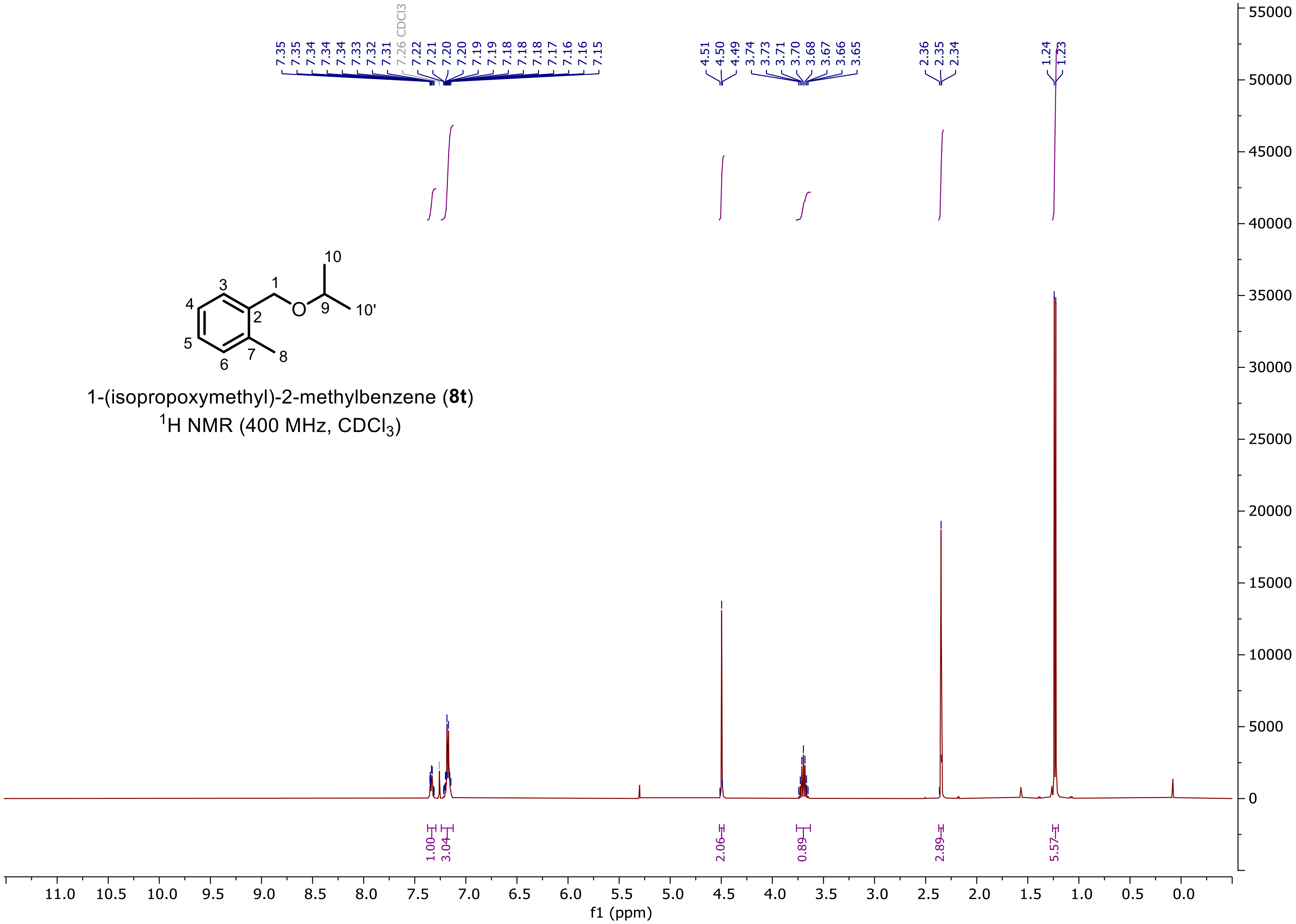

1-(isopropoxymethyl)-2-methylbenzene (**8t**)

^{1}H NMR (400 MHz, $CDCl_3$)

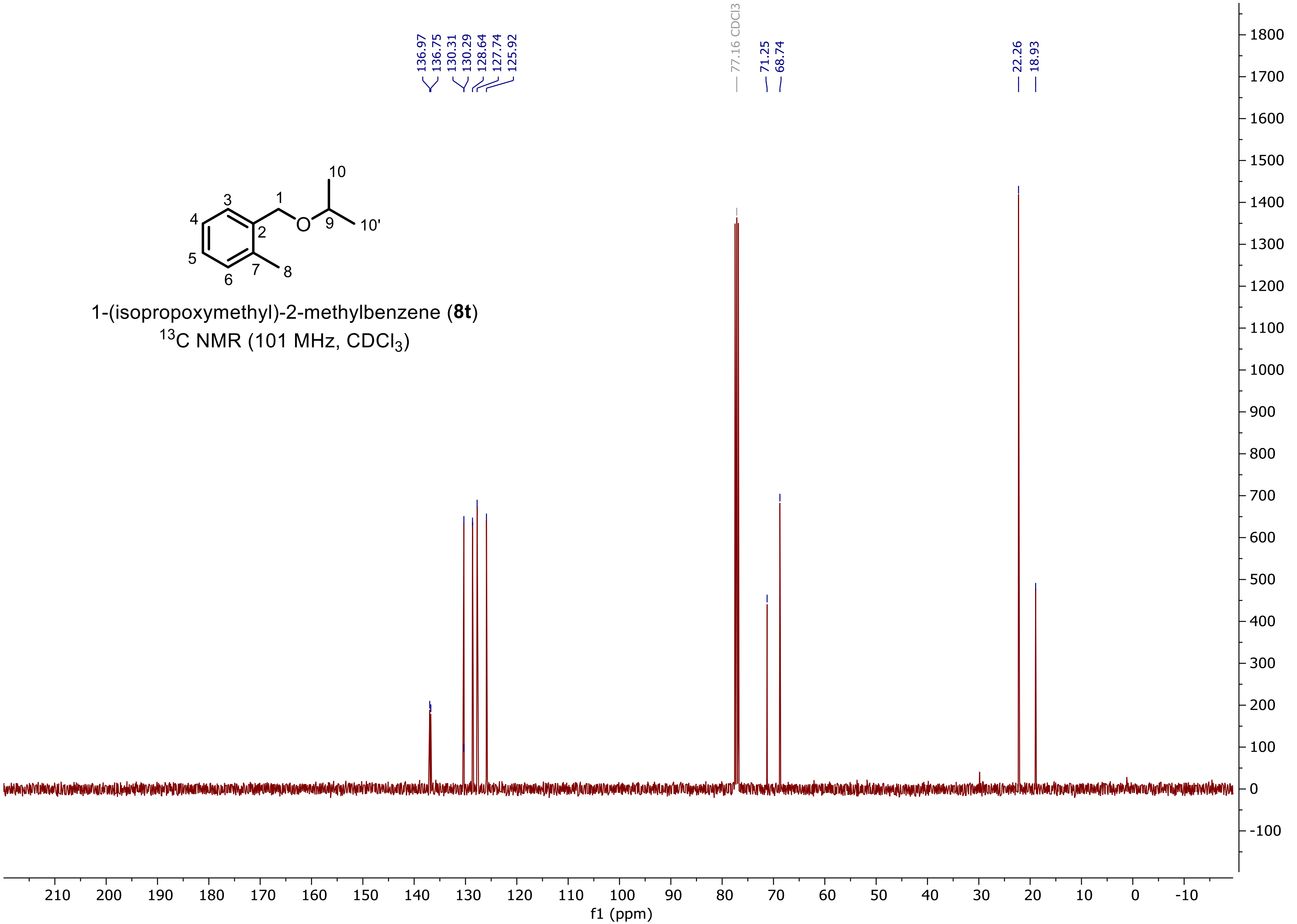
136.97
136.75
130.31
130.29
128.64
127.74
125.92
77.16 CDCl3
71.25
68.74
22.26
18.93
10
1
3
4
2
O
9
10'
5
7
8
6
1-(isopropoxymethyl)-2-methylbenzene (8t)
13C NMR (101 MHz, CDCl3)
f1 (ppm)

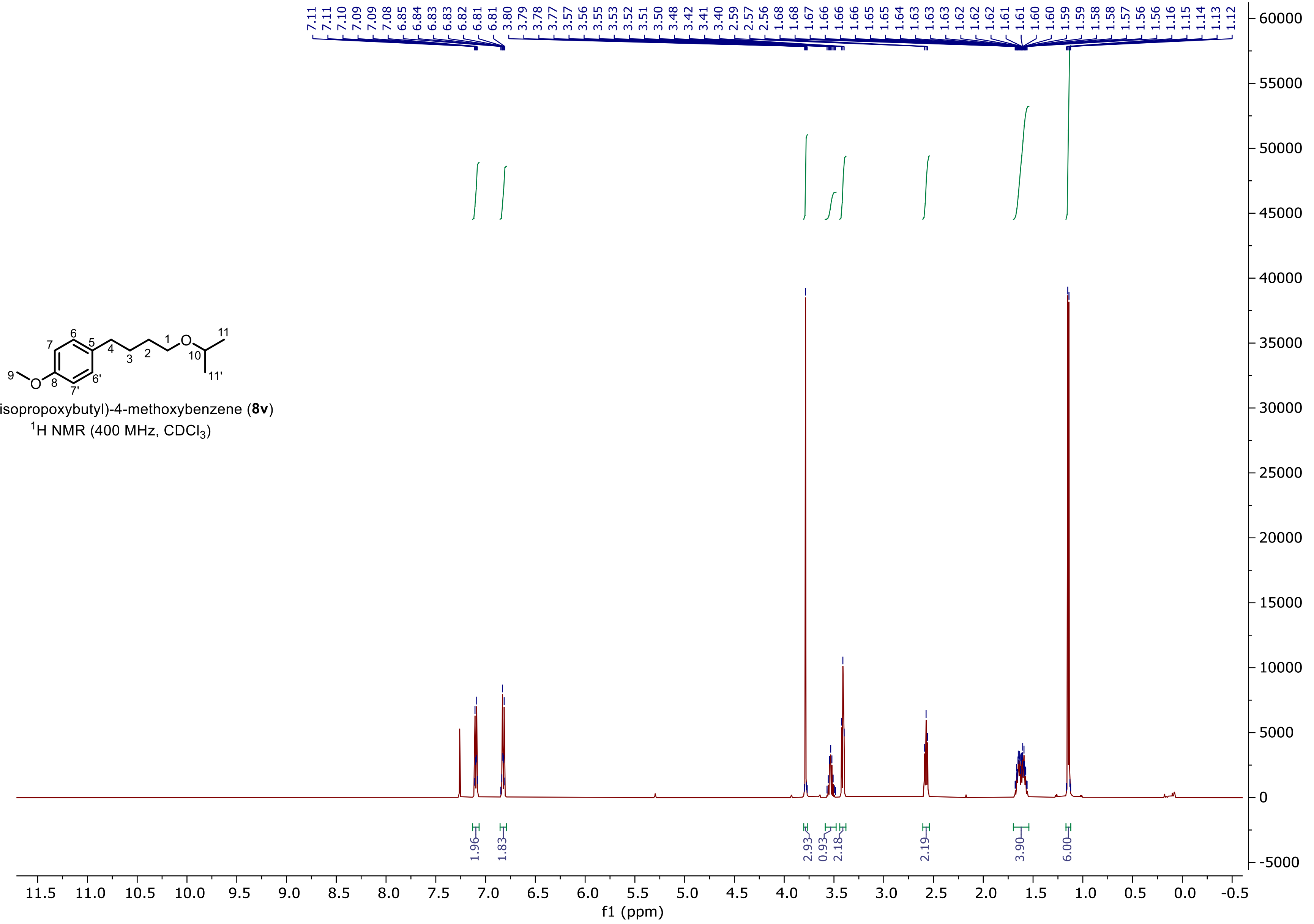

1-(4-isopropoxybutyl)-4-methoxybenzene (**8v**)
^{1}H NMR (400 MHz, $CDCl_3$)

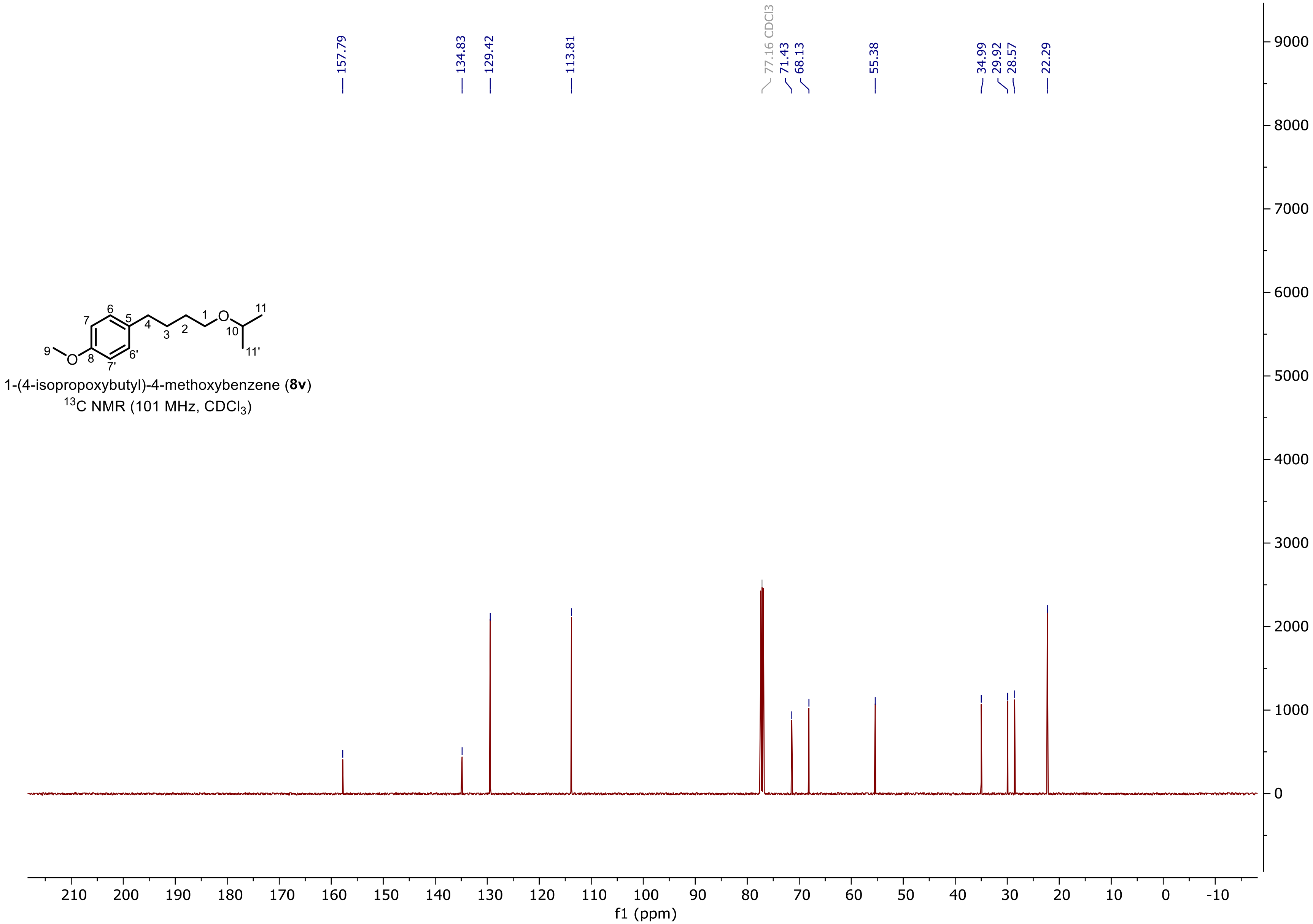

1-(4-isopropoxybutyl)-4-methoxybenzene (**8v**)

^{13}C NMR (101 MHz, $CDCl_3$)

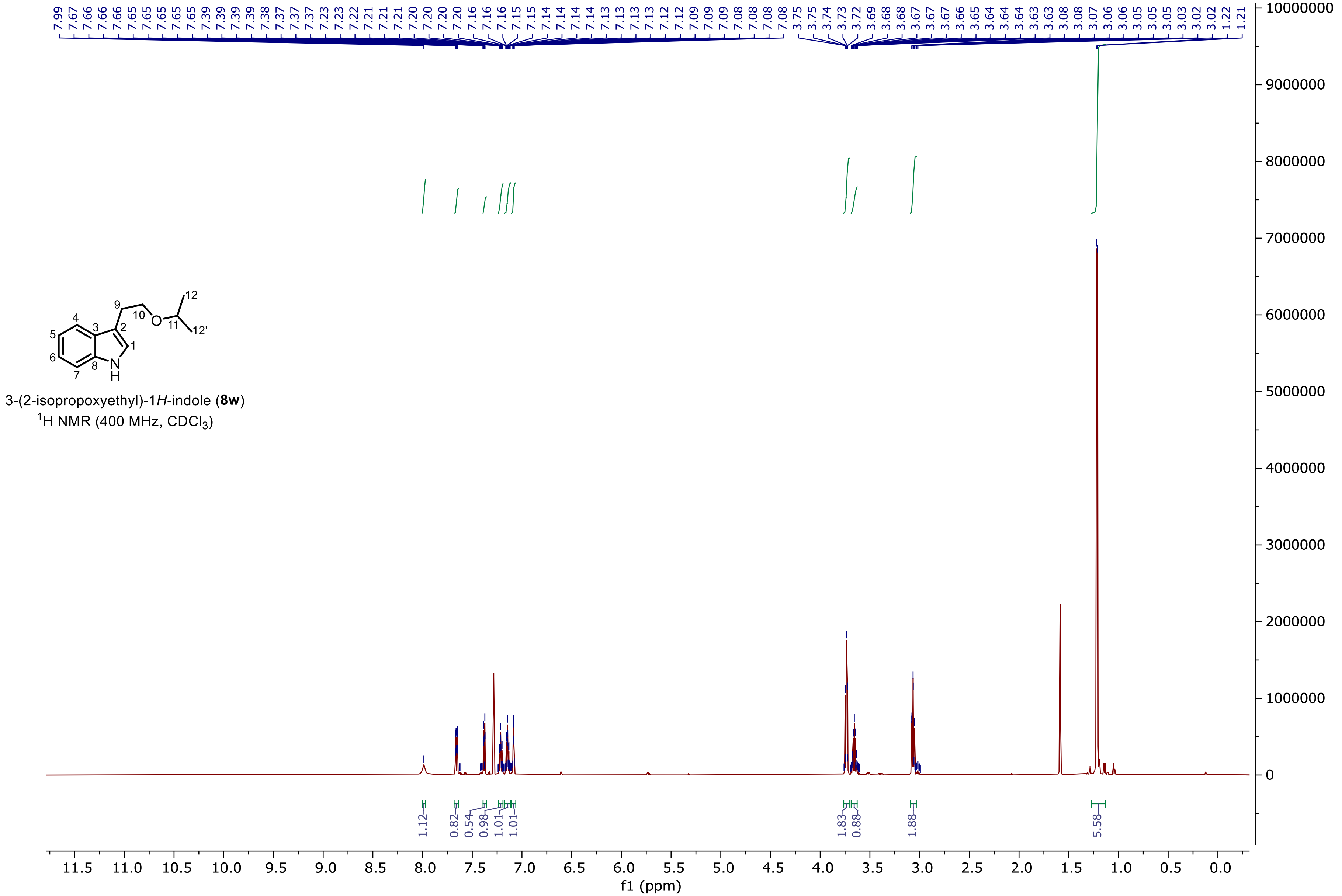

3-(2-isopropoxyethyl)-1*H*-indole (**8w**)
^{1}H NMR (400 MHz, $CDCl_3$)

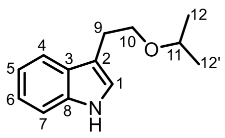

3-(2-isopropoxyethyl)-1*H*-indole (**8w**)
^{13}C NMR (101 MHz, $CDCl_3$)

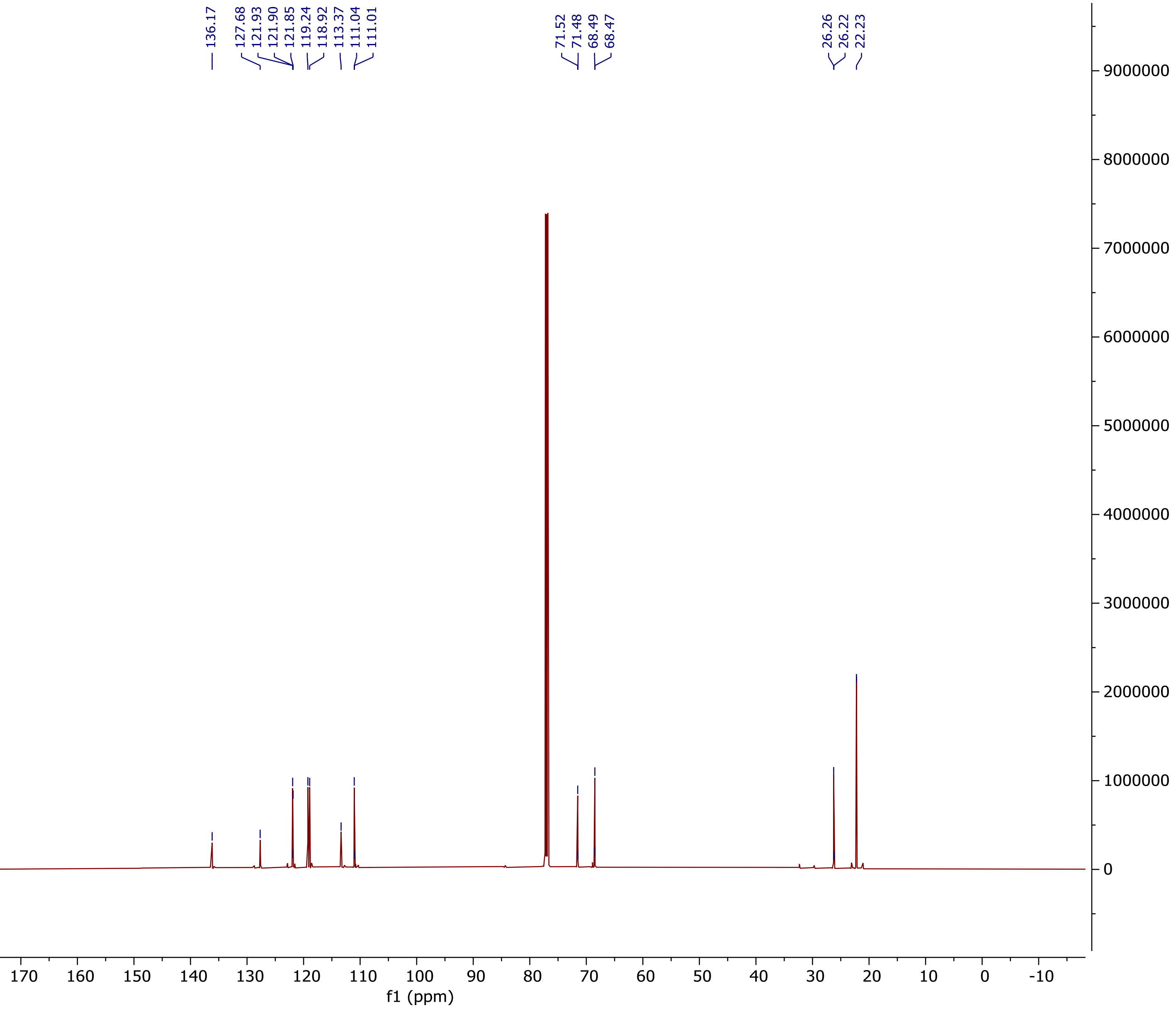

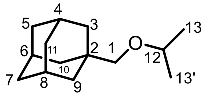

(3*r*,5*r*,7*r*)-1-(isopropoxymethyl)adamantane (**8y**)
^{1}H NMR (400 MHz, $CDCl_3$)

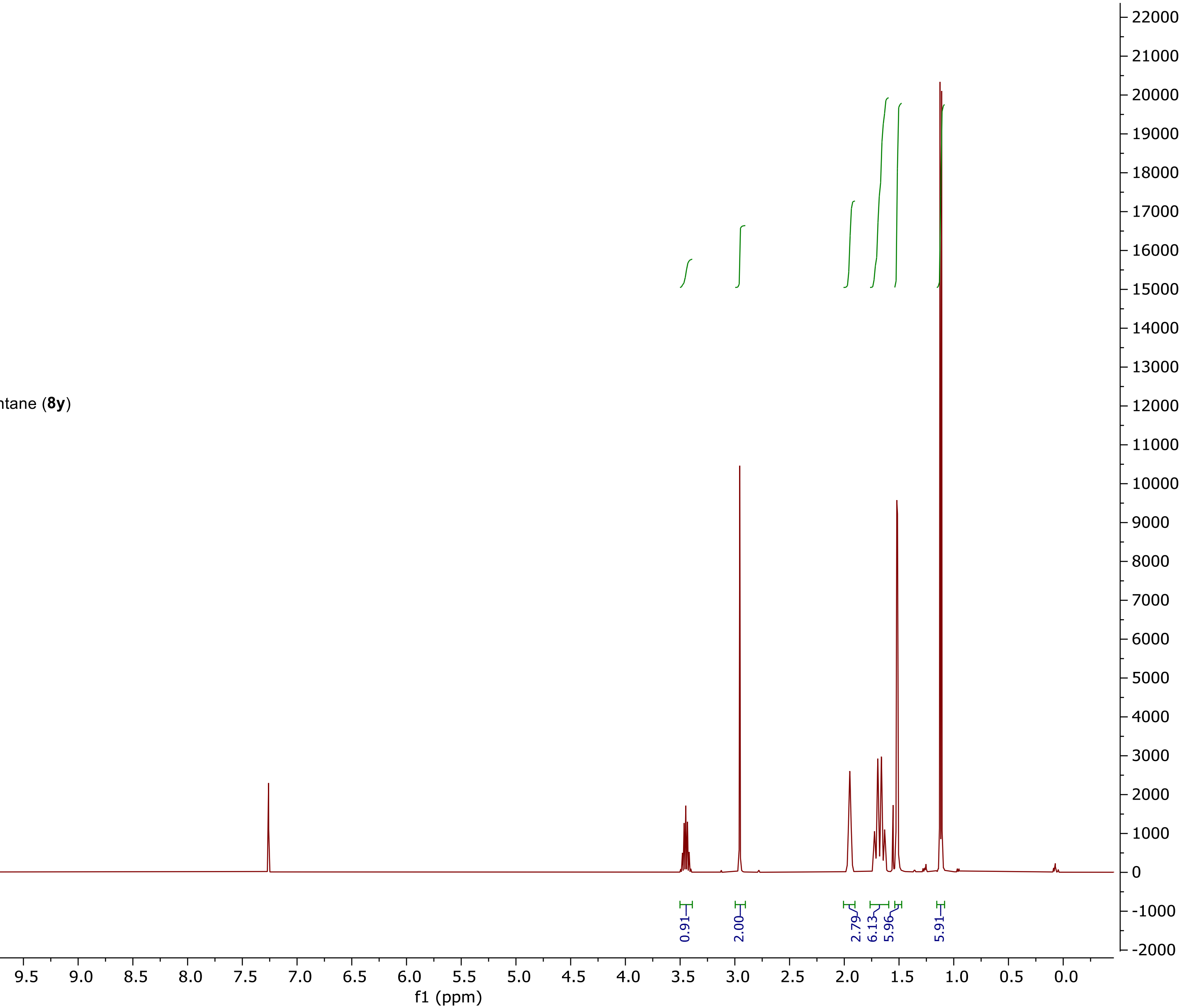

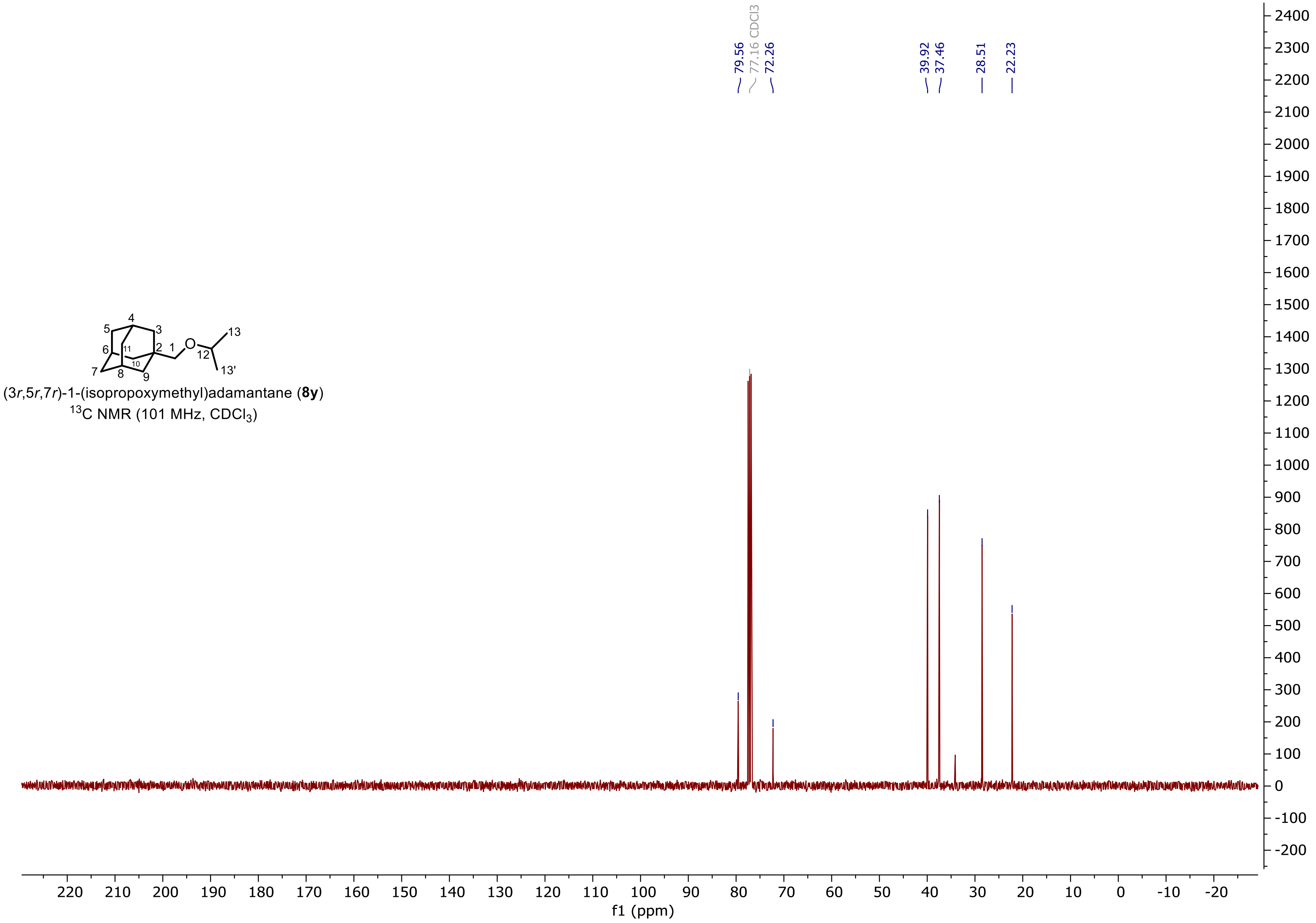
79.56
77.16 CDCl3
72.26
39.92
37.46
28.51
22.23
(3r,5r,7r)-1-(isopropoxymethyl)adamantane (8y)
13C NMR (101 MHz, CDCl3)
f1 (ppm)

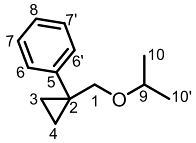

(1-(isopropoxymethyl)cyclopropyl)benzene (**8z**)

^{1}H NMR (400 MHz, $CDCl_3$)

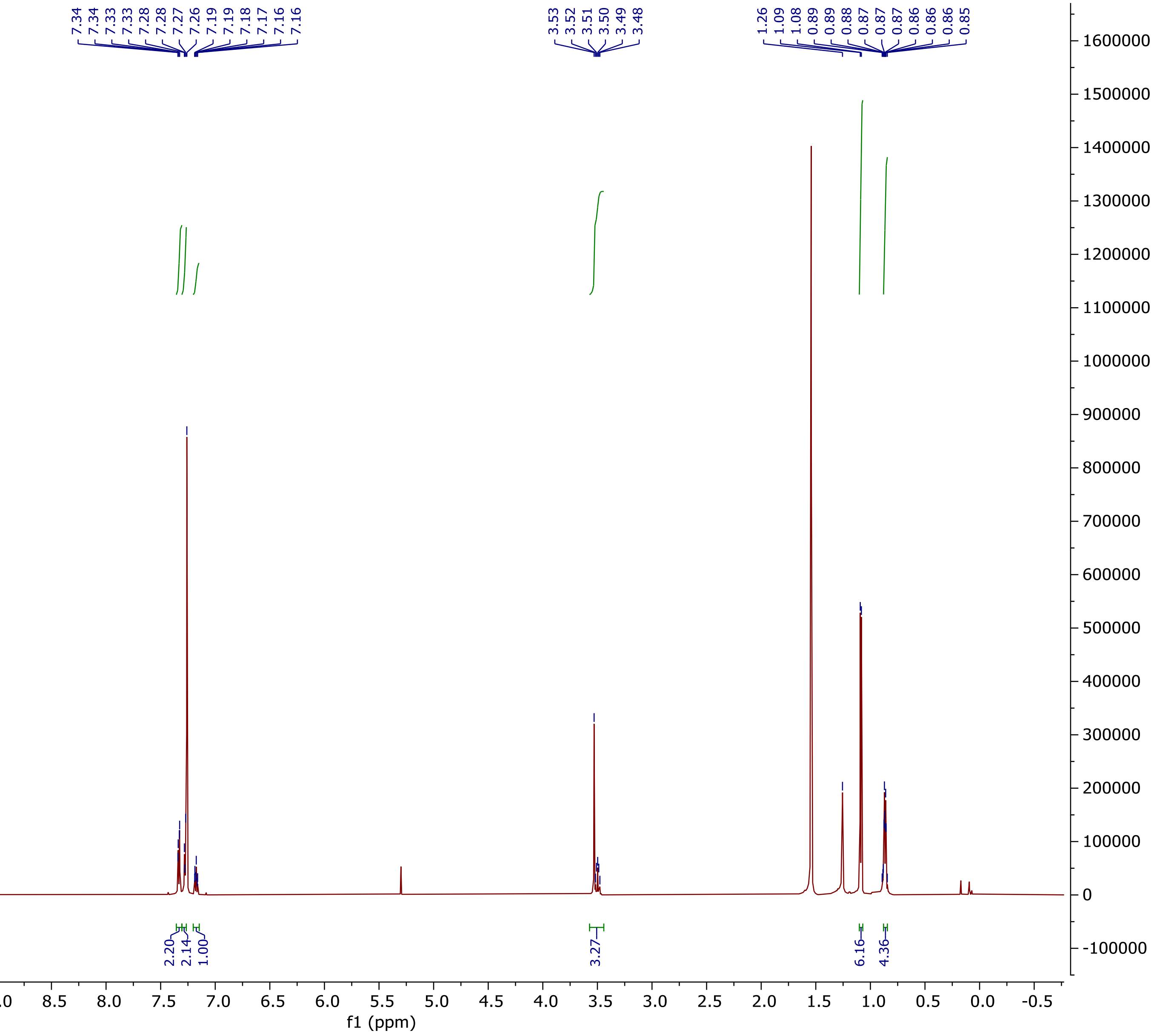

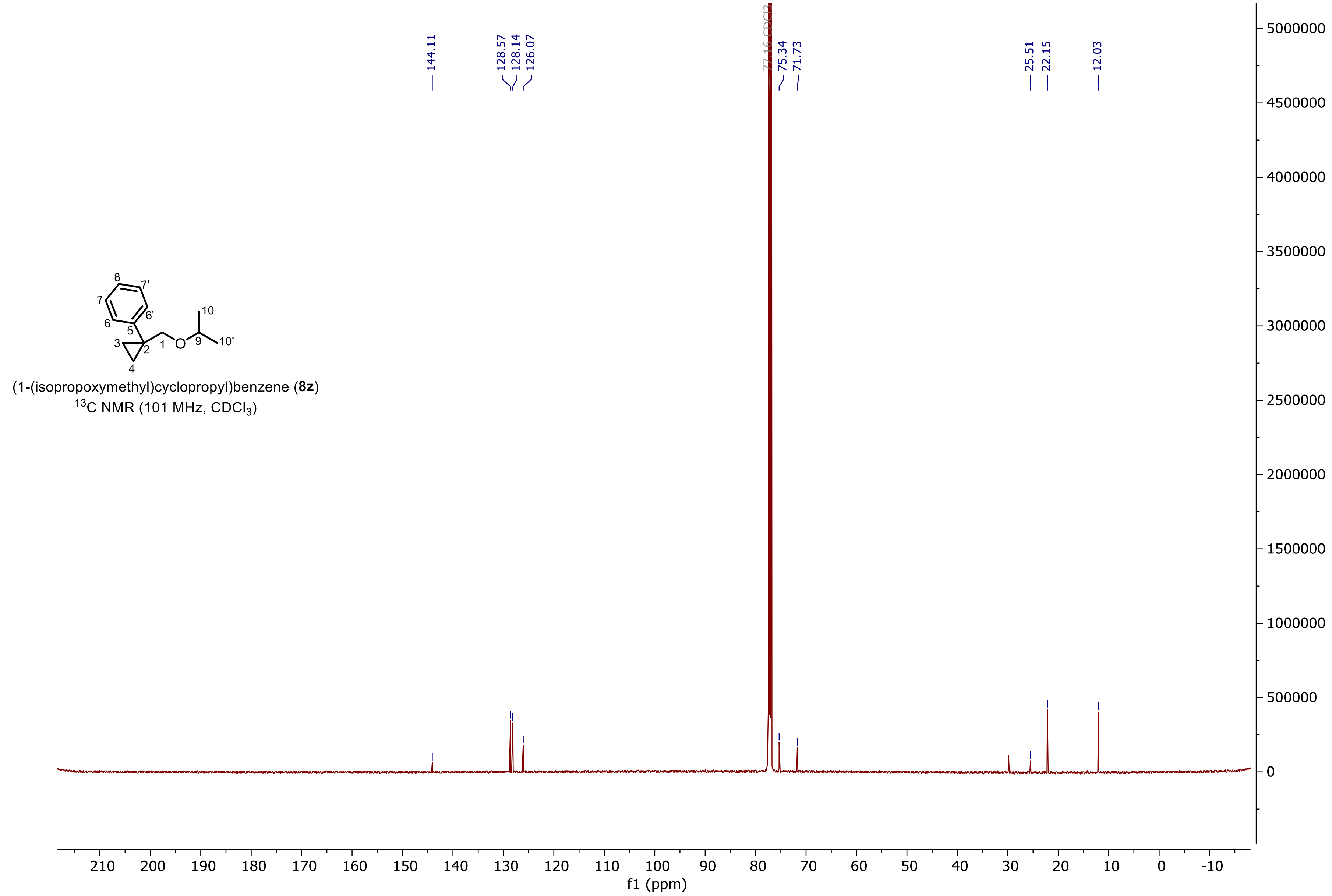

(1-(isopropoxymethyl)cyclopropyl)benzene (**8z**)

^{13}C NMR (101 MHz, $CDCl_3$)

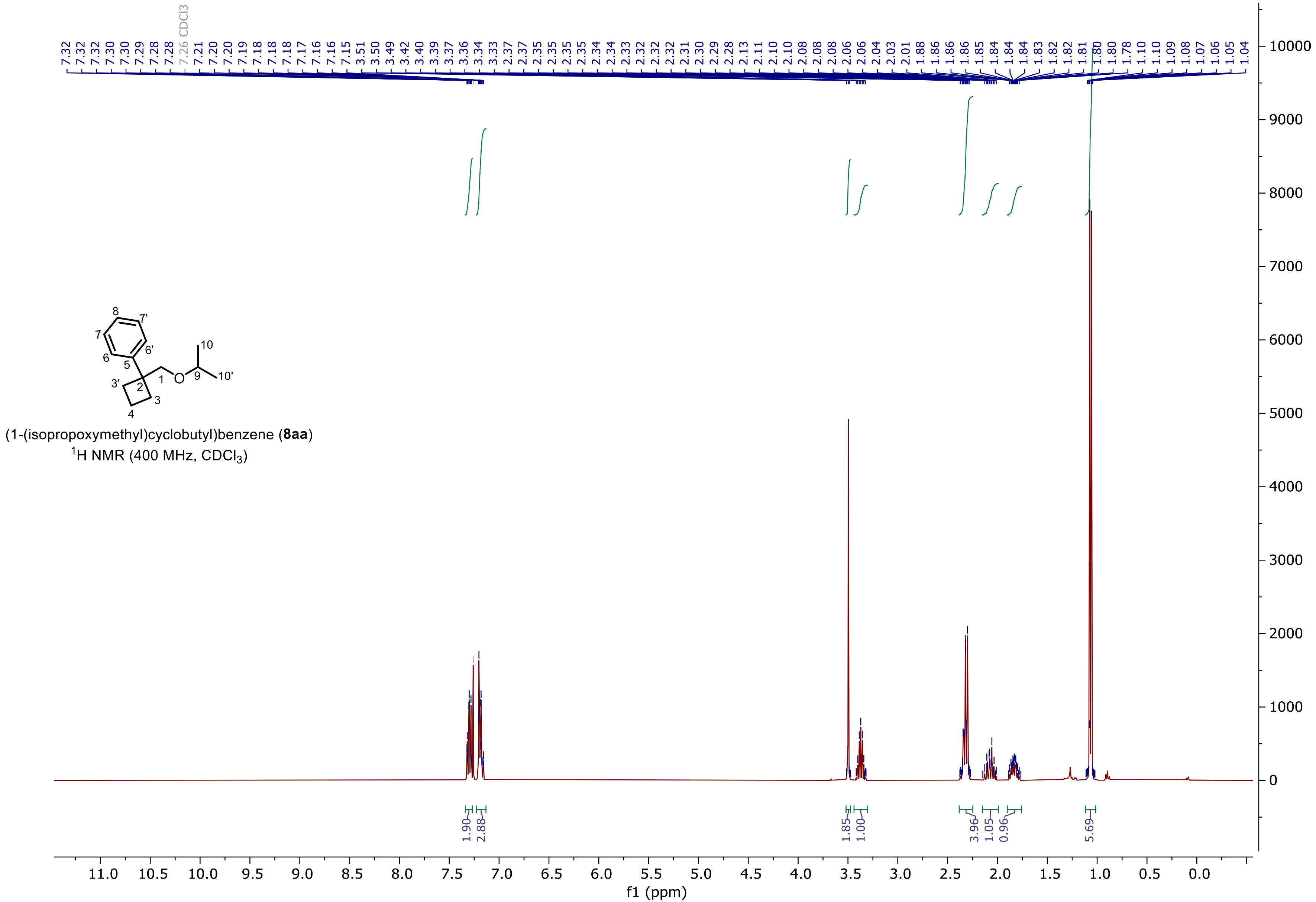

(1-(isopropoxymethyl)cyclobutyl)benzene (**8aa**)
^{1}H NMR (400 MHz, $CDCl_3$)

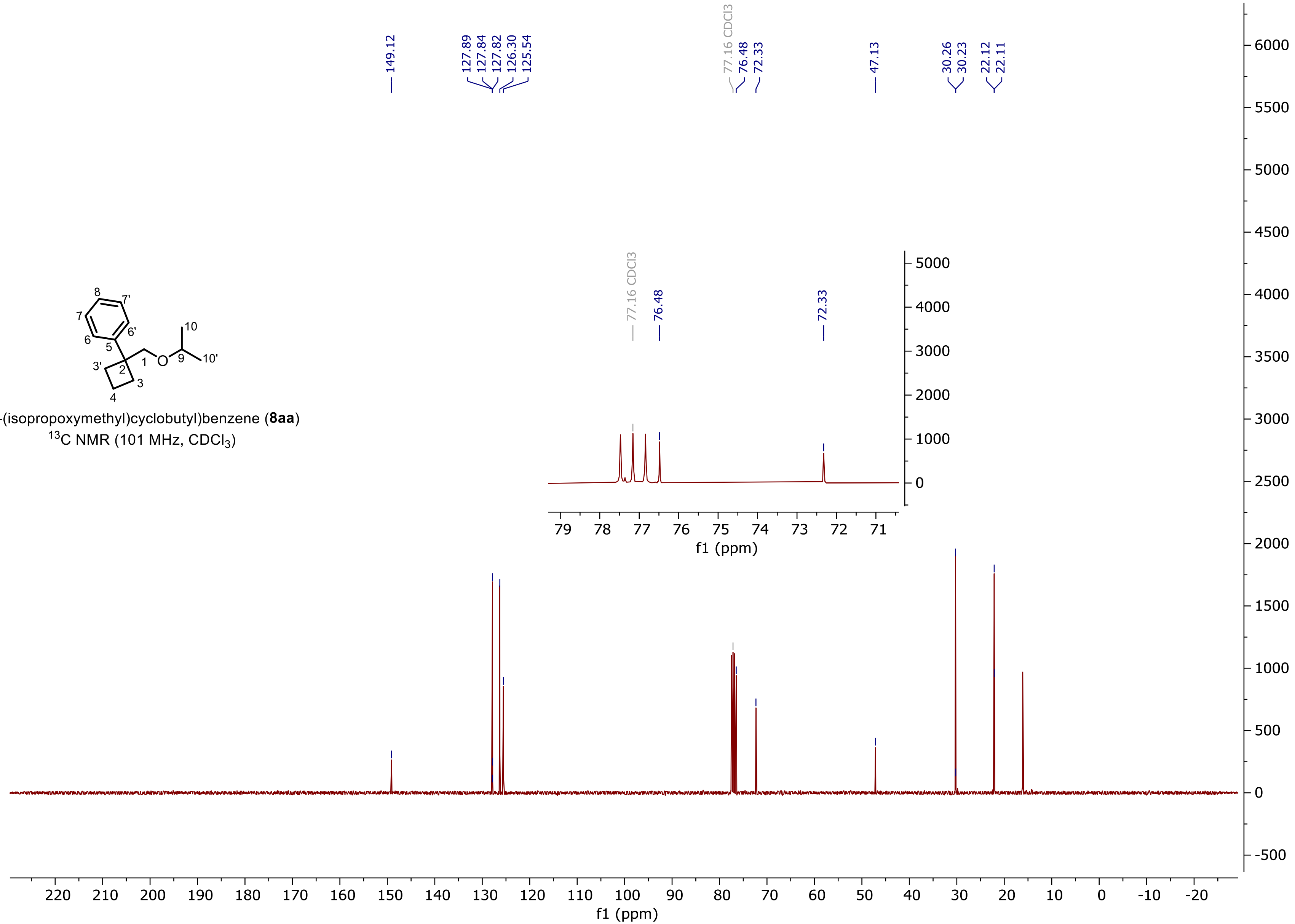

(1-(isopropoxymethyl)cyclobutyl)benzene (**8aa**)
^{13}C NMR (101 MHz, $CDCl_3$)

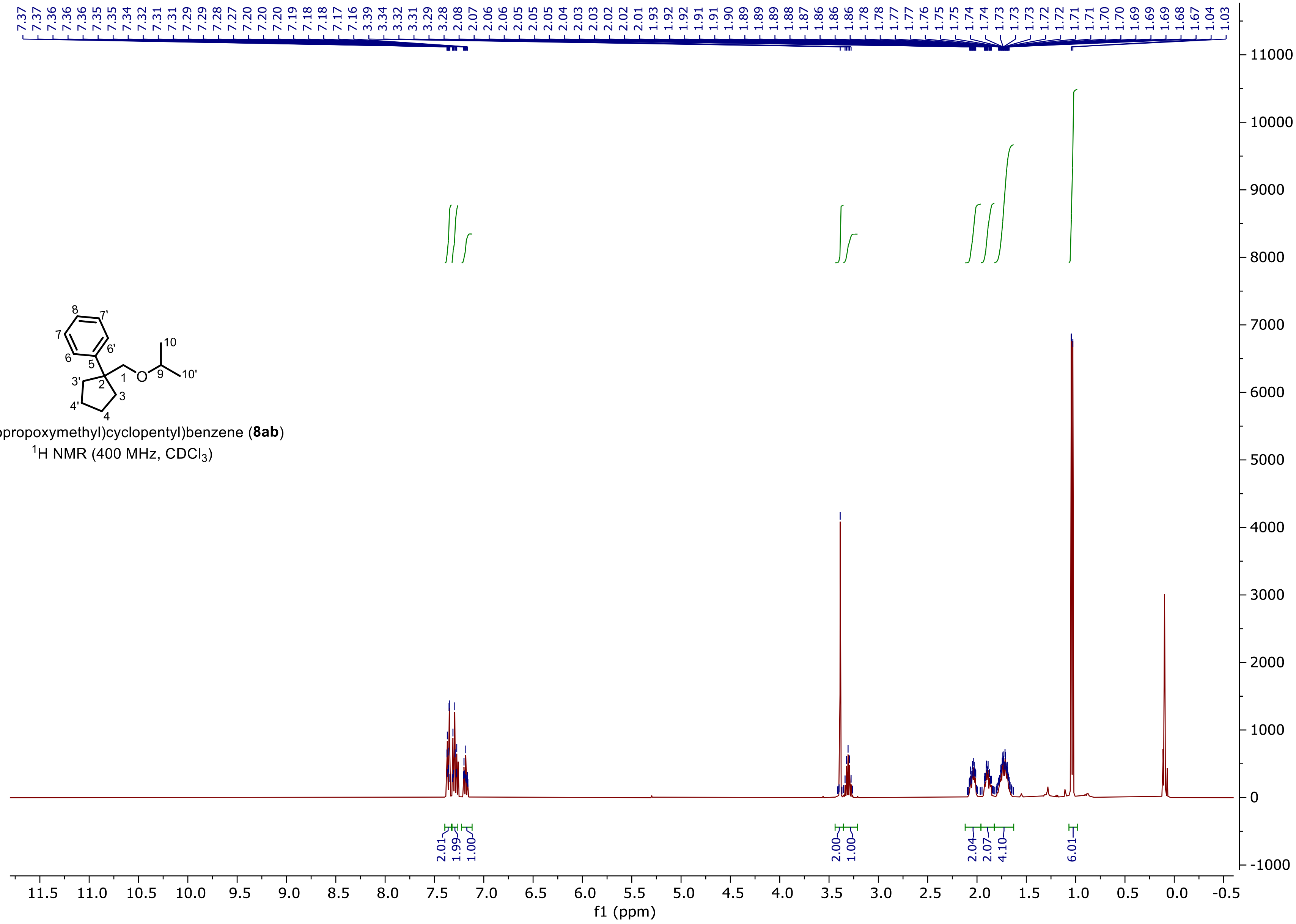

(1-(isopropoxymethyl)cyclopentyl)benzene (**8ab**)

^{1}H NMR (400 MHz, $CDCl_3$)

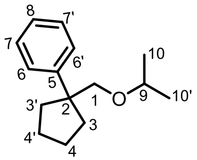

(1-(isopropoxymethyl)cyclopentyl)benzene (**8ab**)

^{13}C NMR (101 MHz, $CDCl_3$)

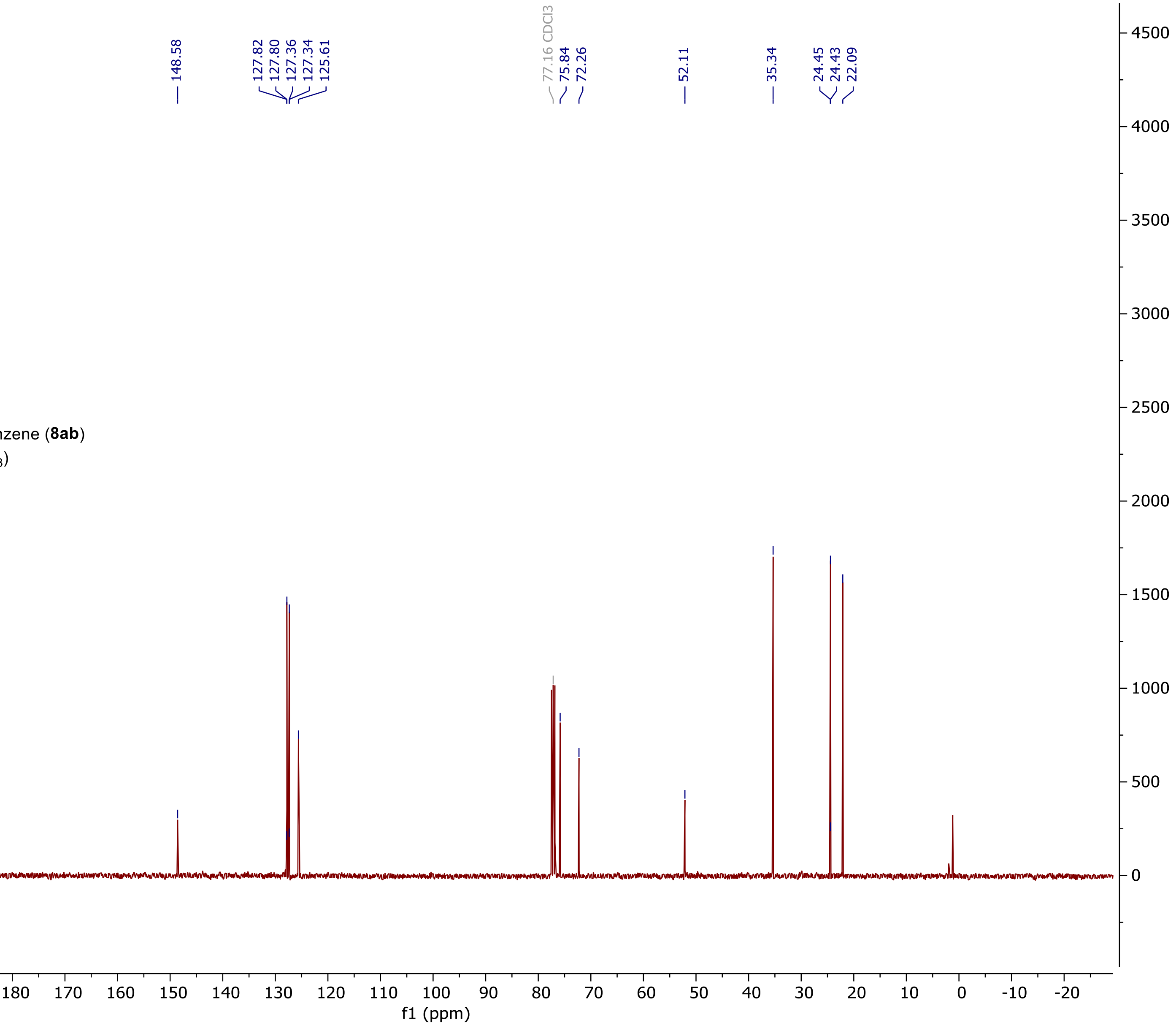

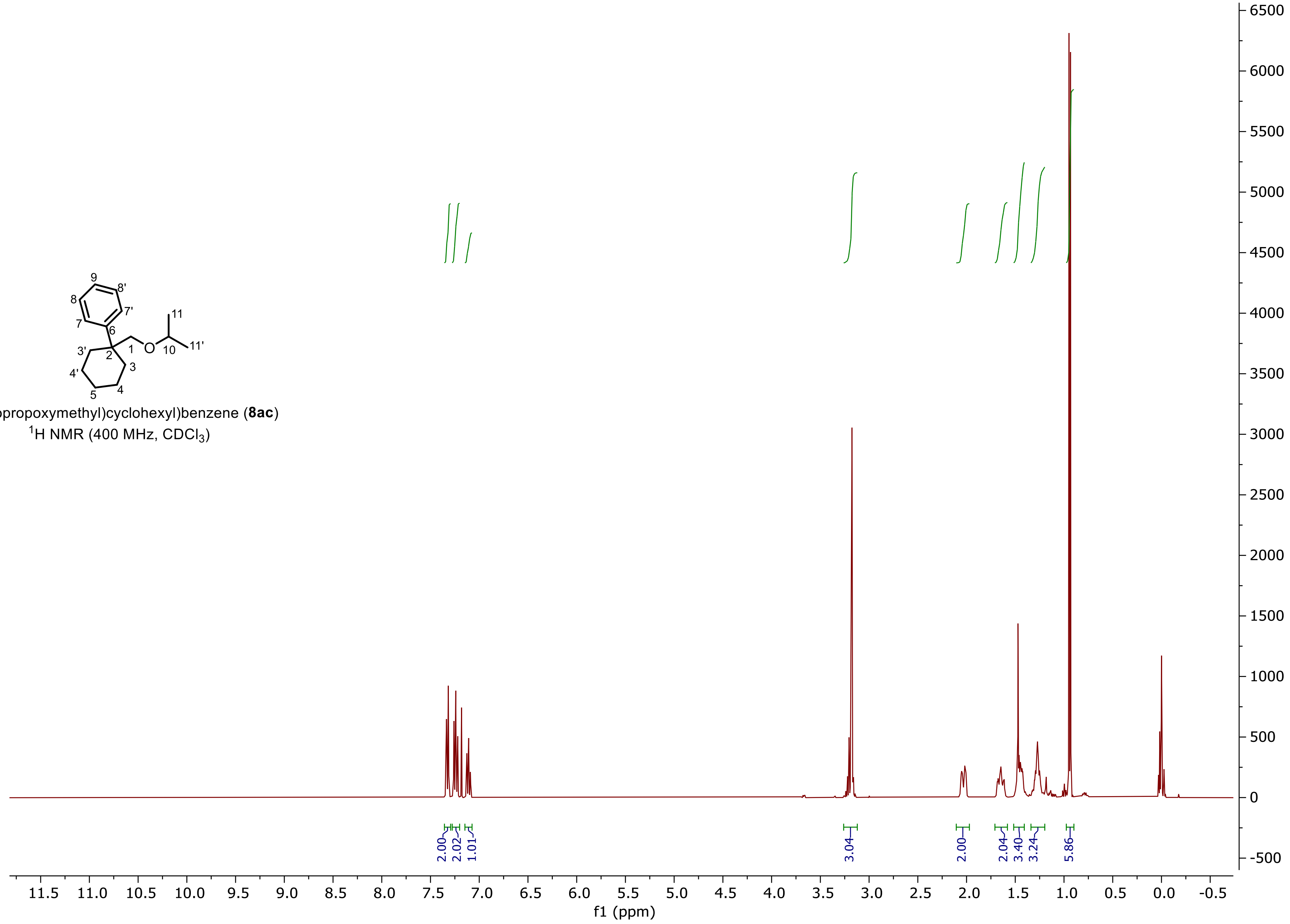

(1-(isopropoxymethyl)cyclohexyl)benzene (**8ac**)
^{1}H NMR (400 MHz, $CDCl_3$)

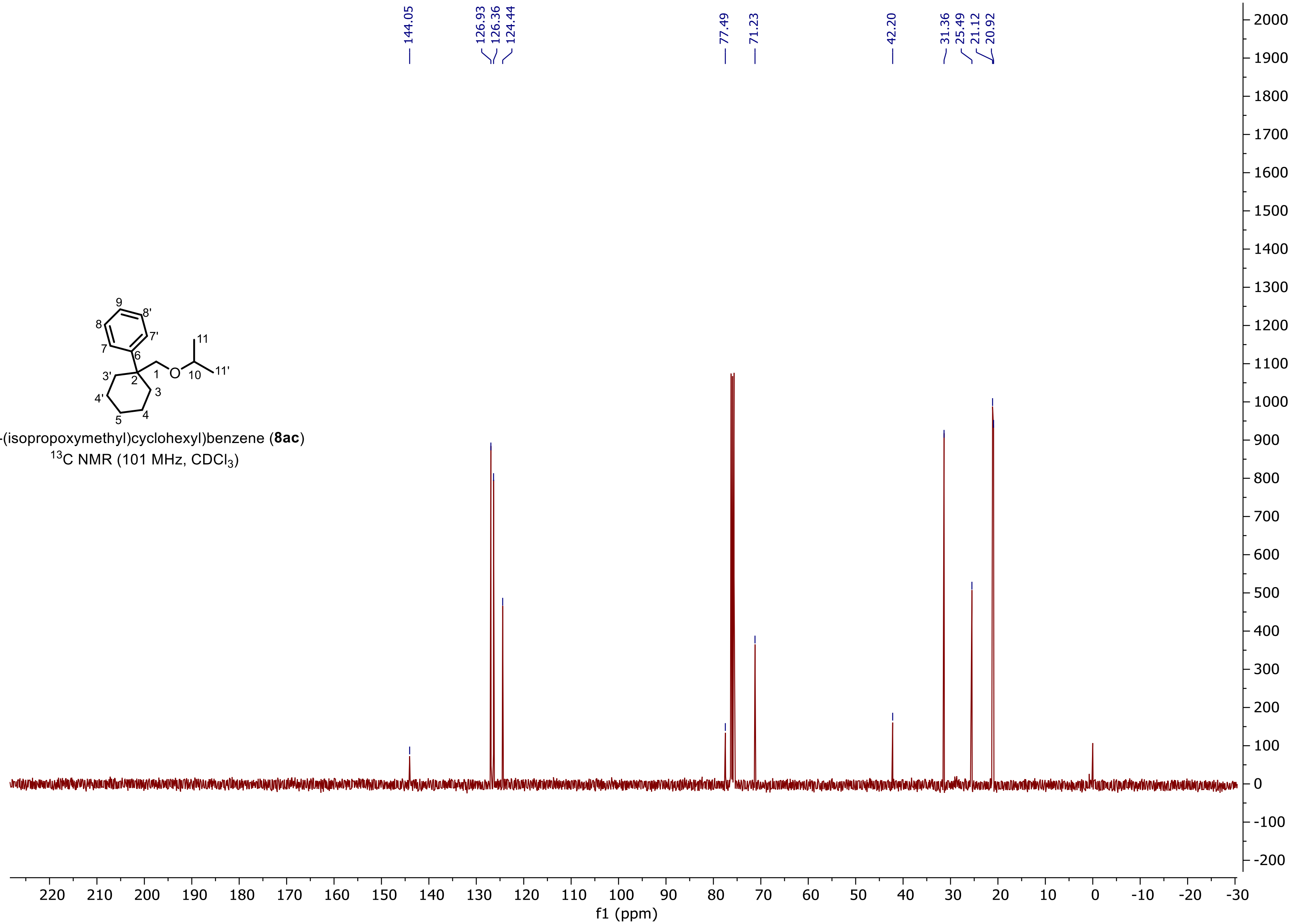

(1-(isopropoxymethyl)cyclohexyl)benzene (**8ac**)

^{13}C NMR (101 MHz, $CDCl_3$)

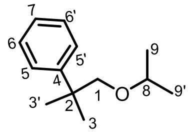

(1-isopropoxy-2-methylpropan-2-yl)benzene (**8ad**)
^{1}H NMR (400 MHz, $CDCl_3$)

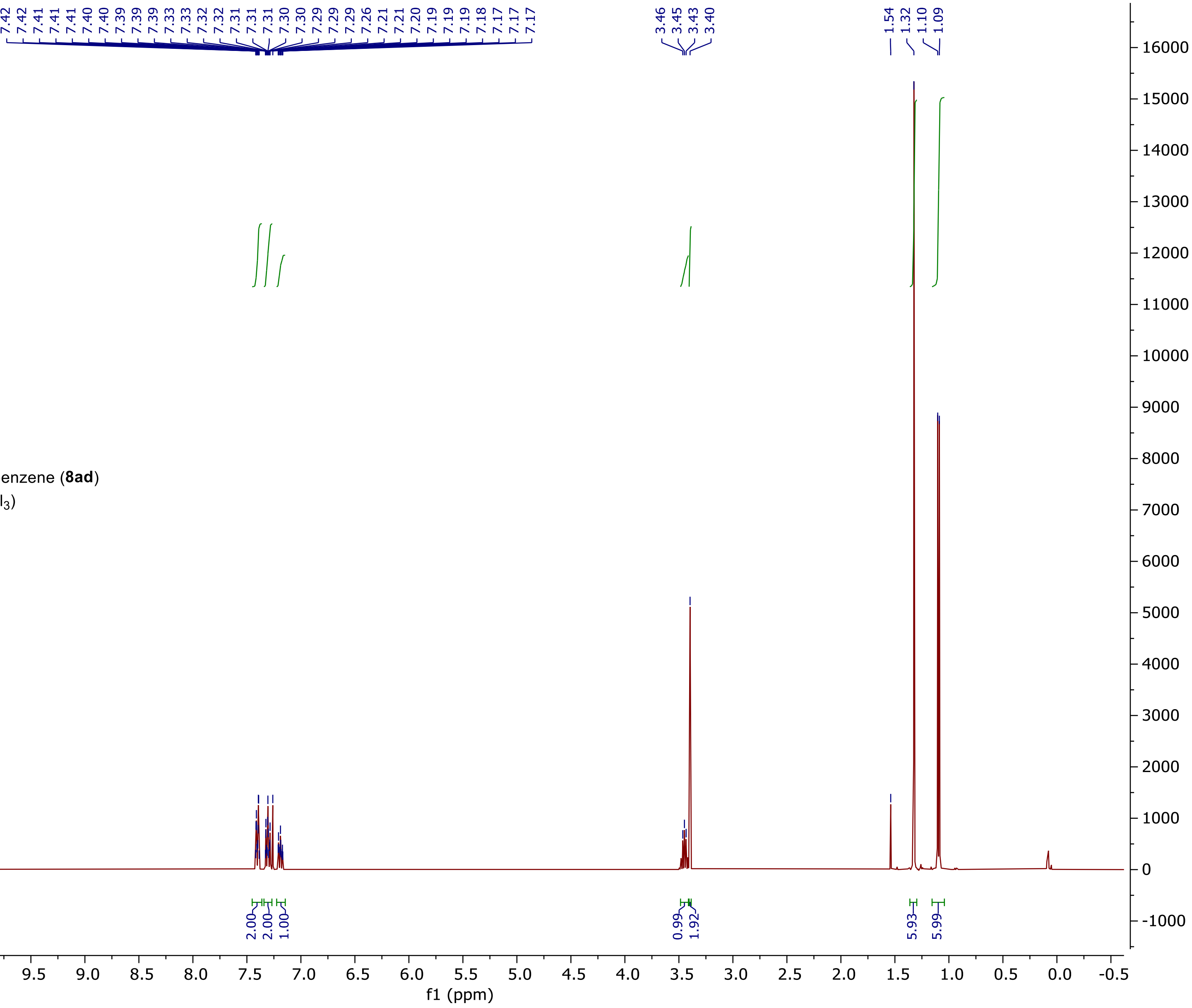

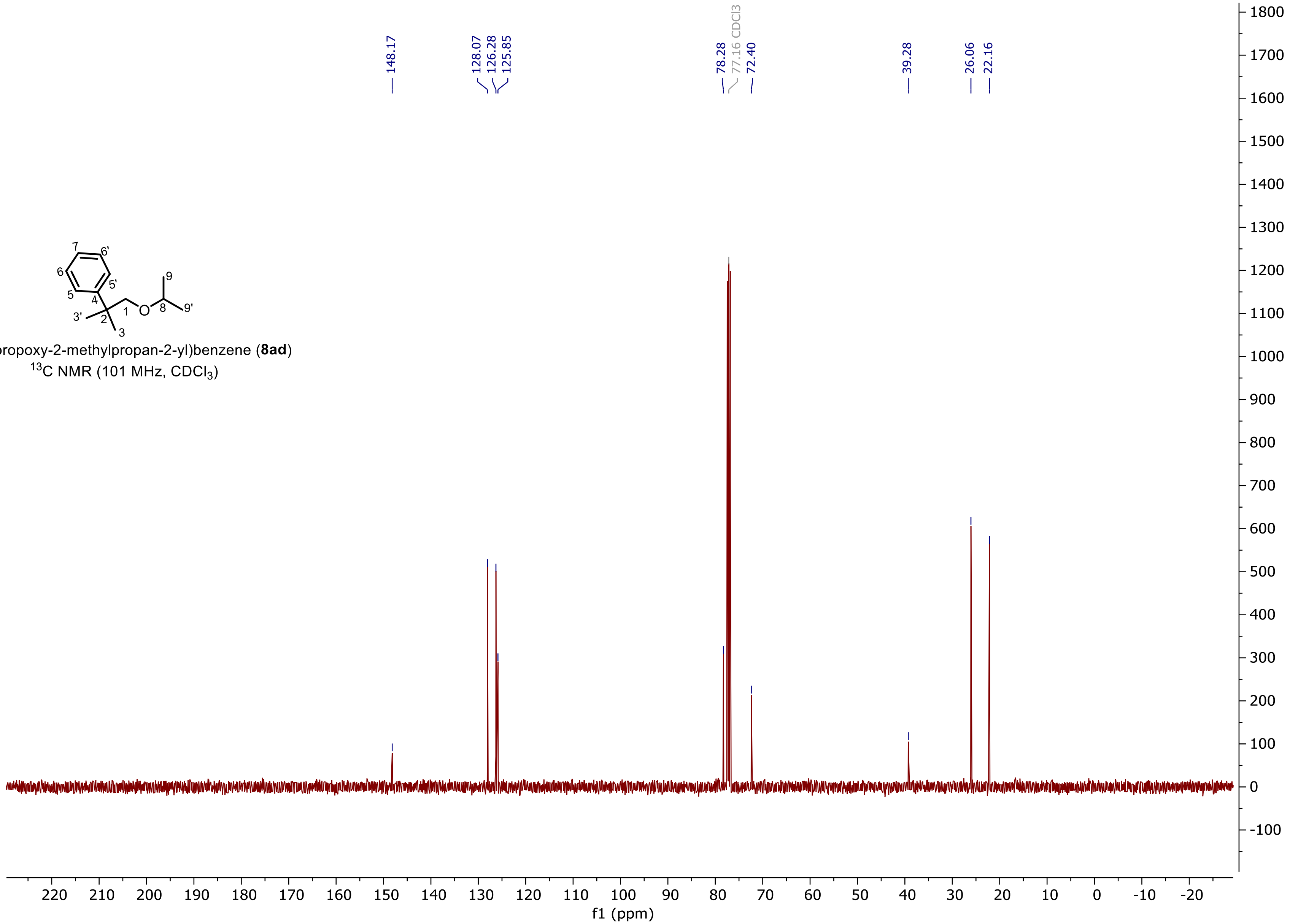

(1-isopropoxy-2-methylpropan-2-yl)benzene (**8ad**)
^{13}C NMR (101 MHz, $CDCl_3$)

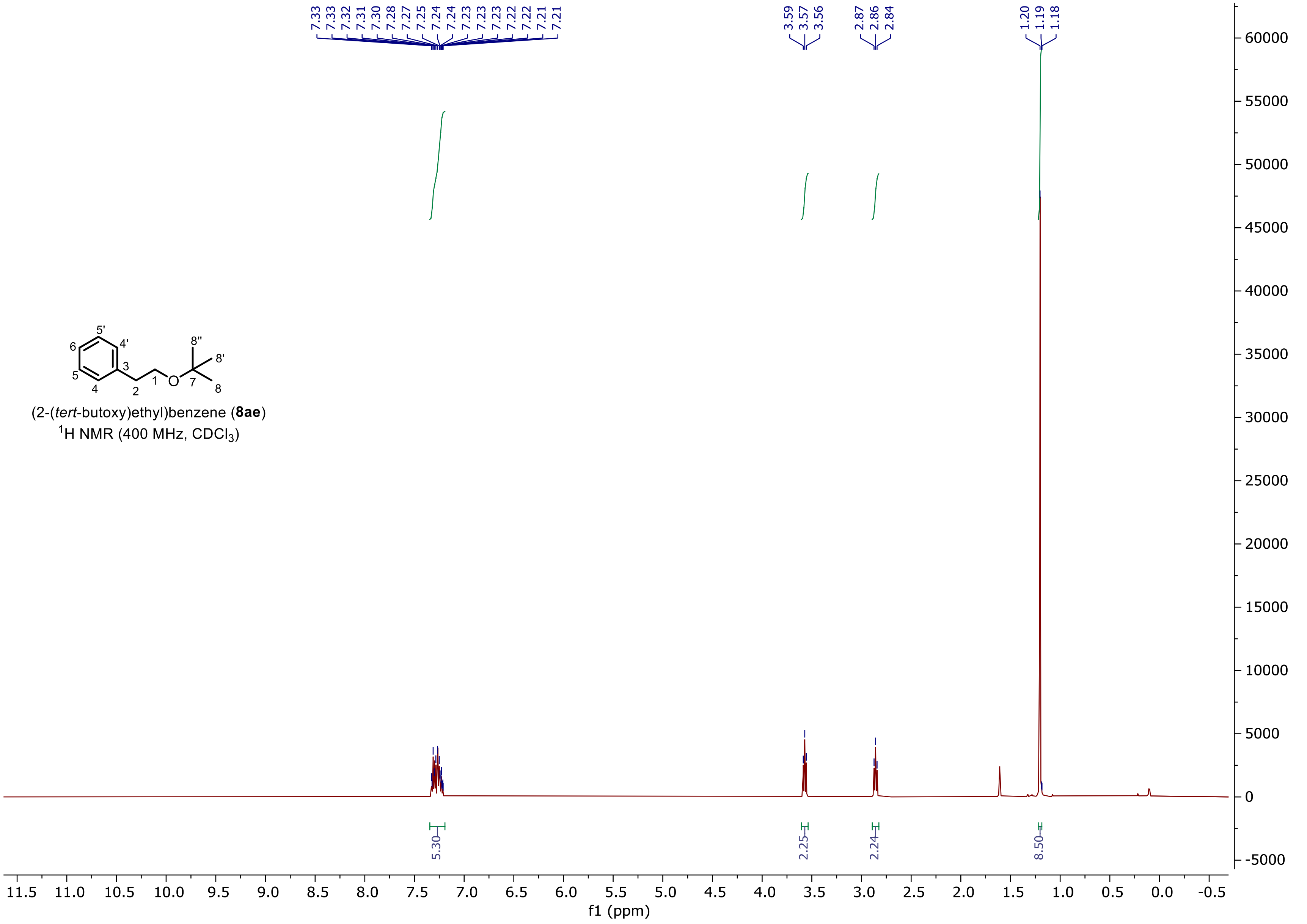

(2-(*tert*-butoxy)ethyl)benzene (**8ae**)
^{1}H NMR (400 MHz, $CDCl_3$)

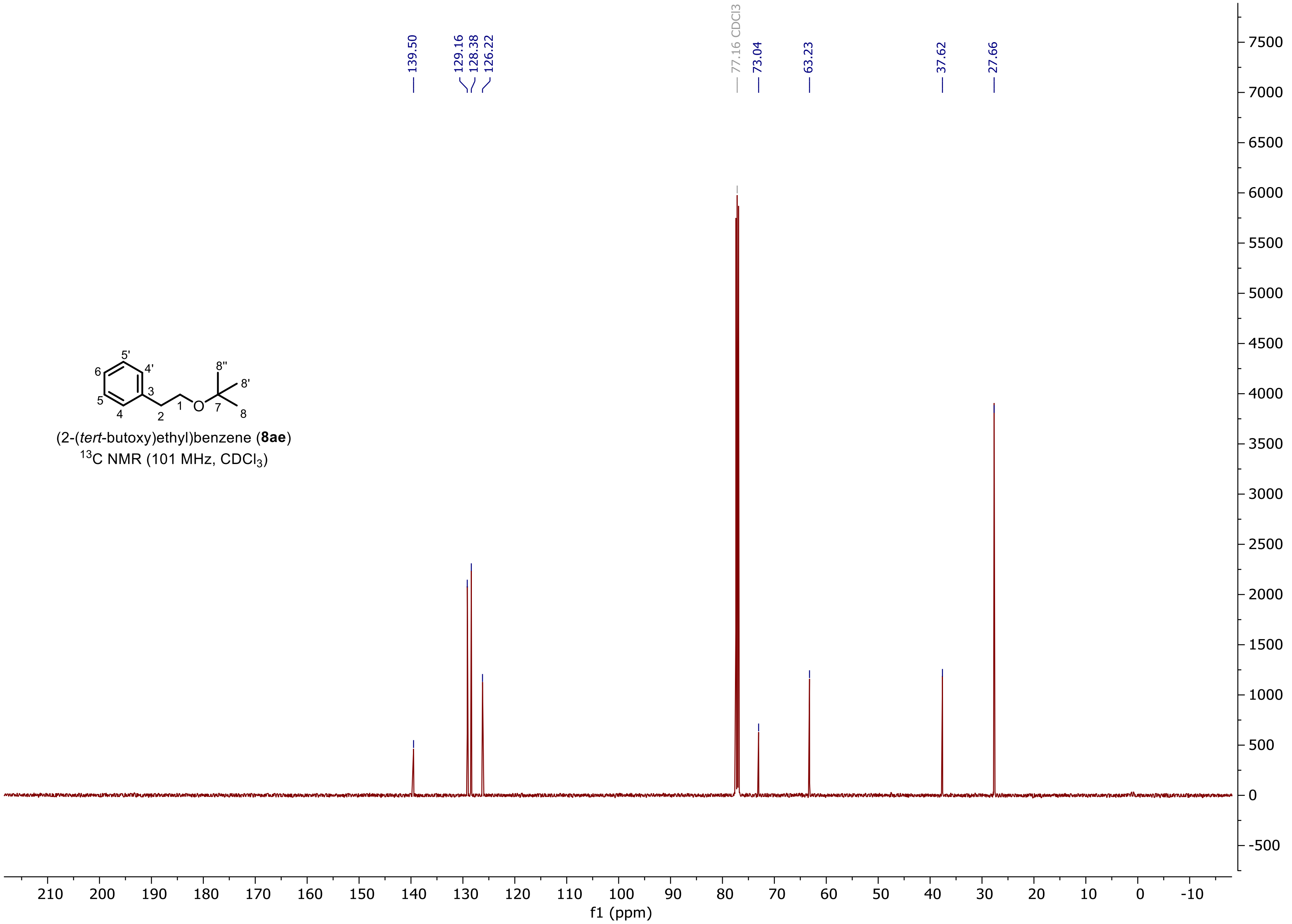

(2-(*tert*-butoxy)ethyl)benzene (**8ae**)
^{13}C NMR (101 MHz, $CDCl_3$)

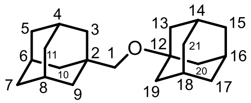

1-adamantan-1-yl)methoxy)adamantane (**8af**)
^{1}H NMR (400 MHz, $CDCl_3$)

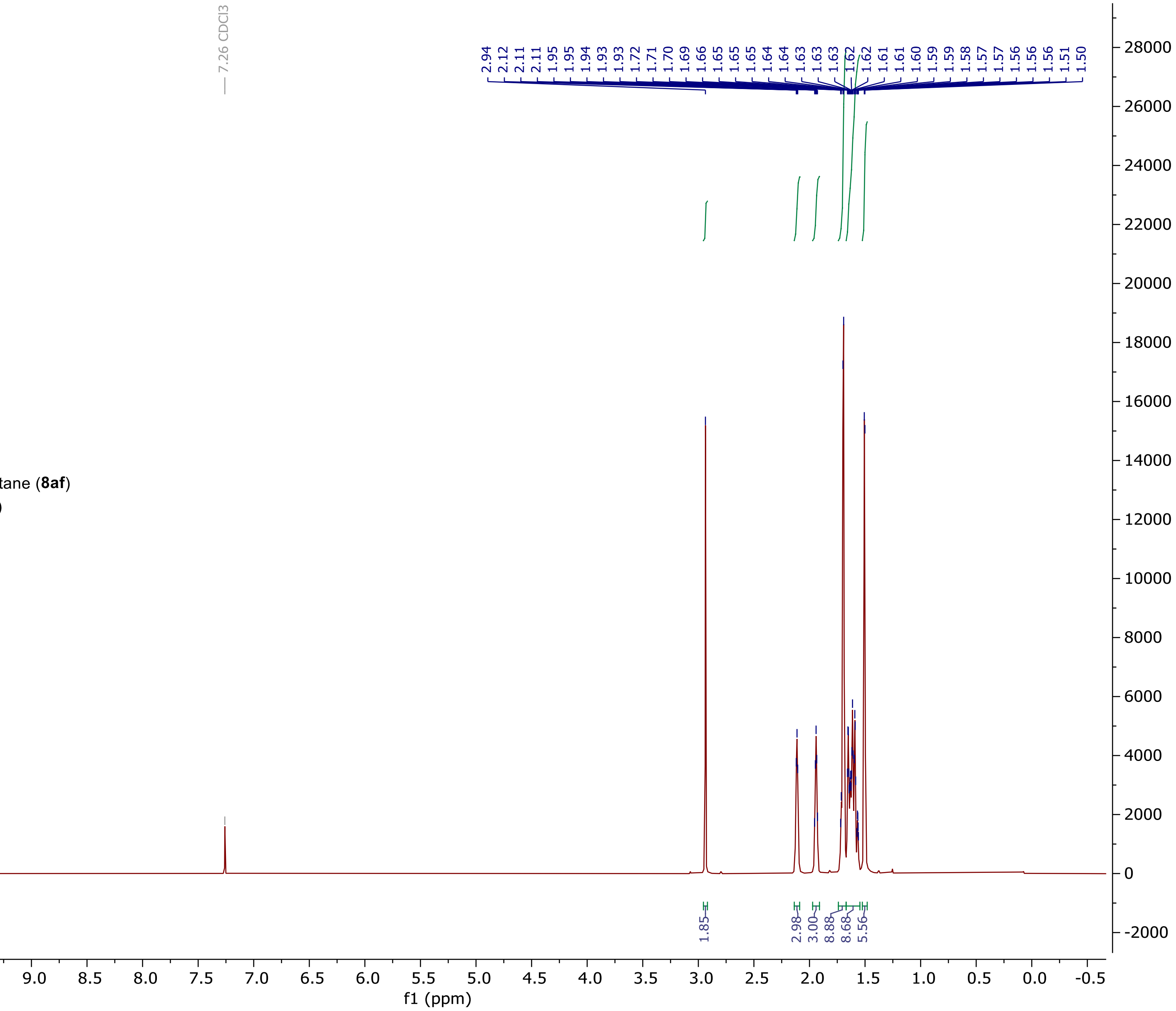

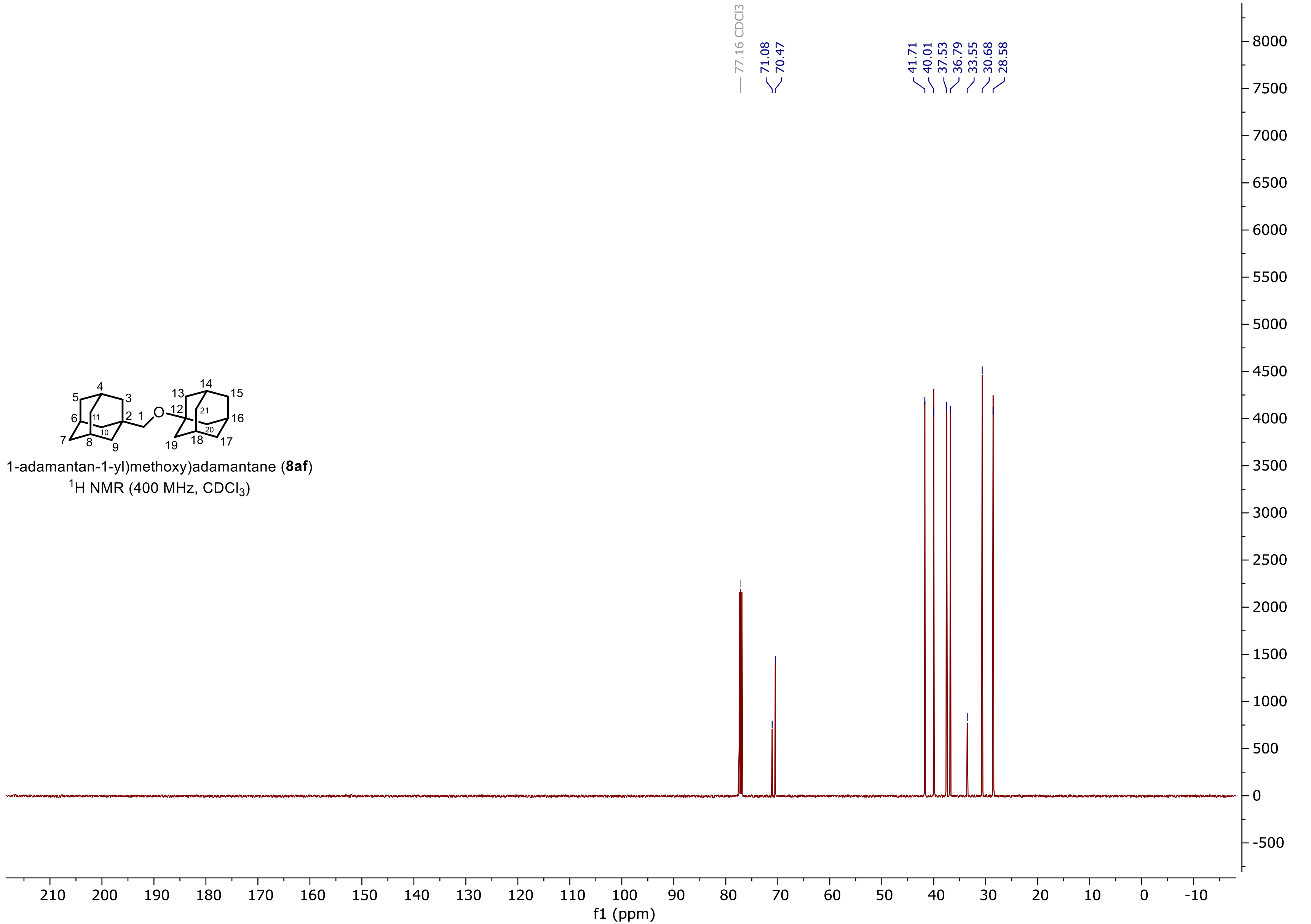

1-adamantan-1-yl)methoxy)adamantane (**8af**)
^{1}H NMR (400 MHz, $CDCl_3$)

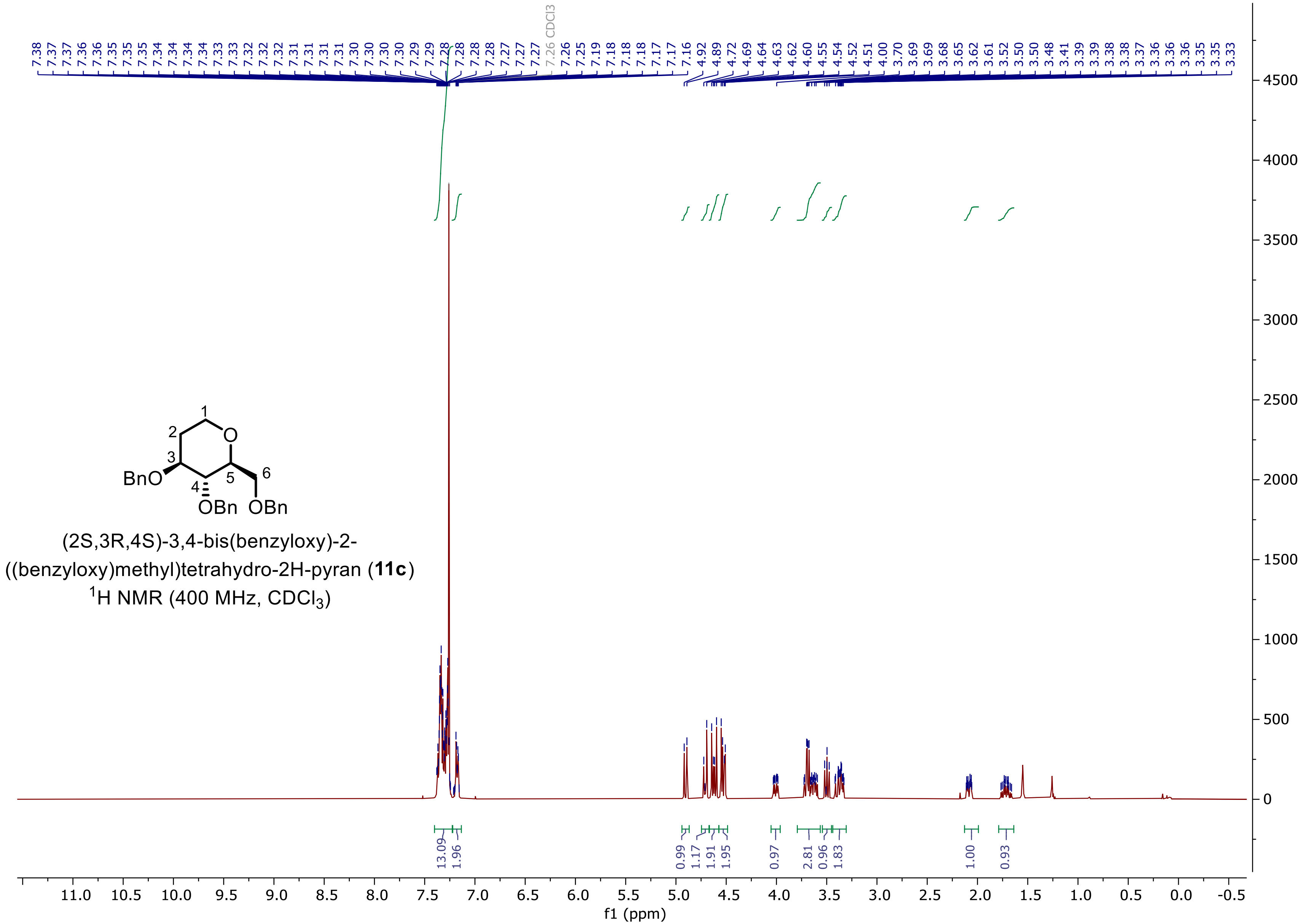
BnO
OBn
OBn
(2S,3R,4S)-3,4-bis(benzyloxy)-2-((benzyloxy)methyl)tetrahydro-2H-pyran (**11c**)
^{1}H NMR (400 MHz, $CDCl_3$)
f1 (ppm)

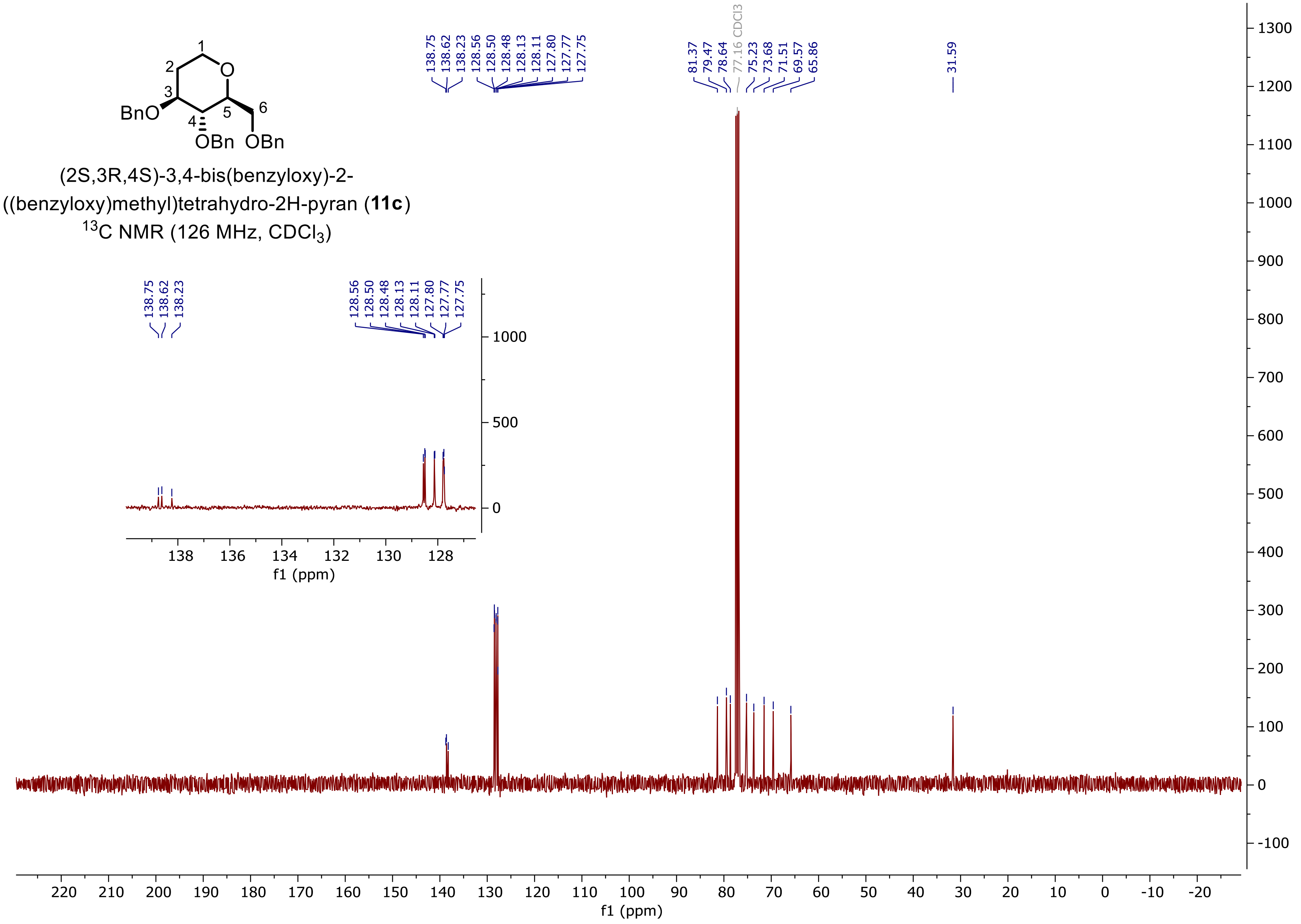

(2S,3R,4S)-3,4-bis(benzyloxy)-2-((benzyloxy)methyl)tetrahydro-2H-pyran (**11c**)

^{13}C NMR (126 MHz, $CDCl_3$)

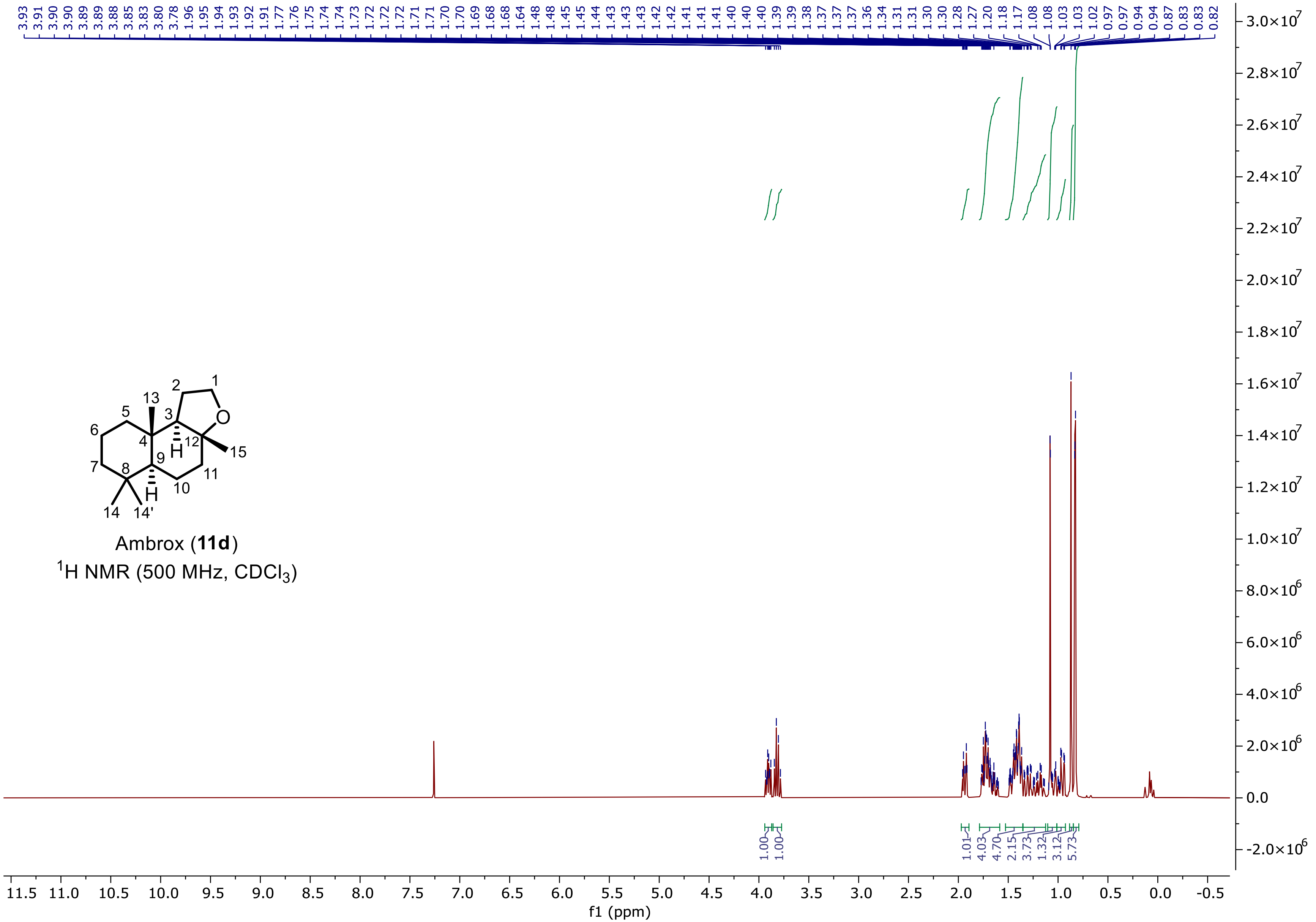

Ambrox (**11d**)
^{1}H NMR (500 MHz, $CDCl_3$)

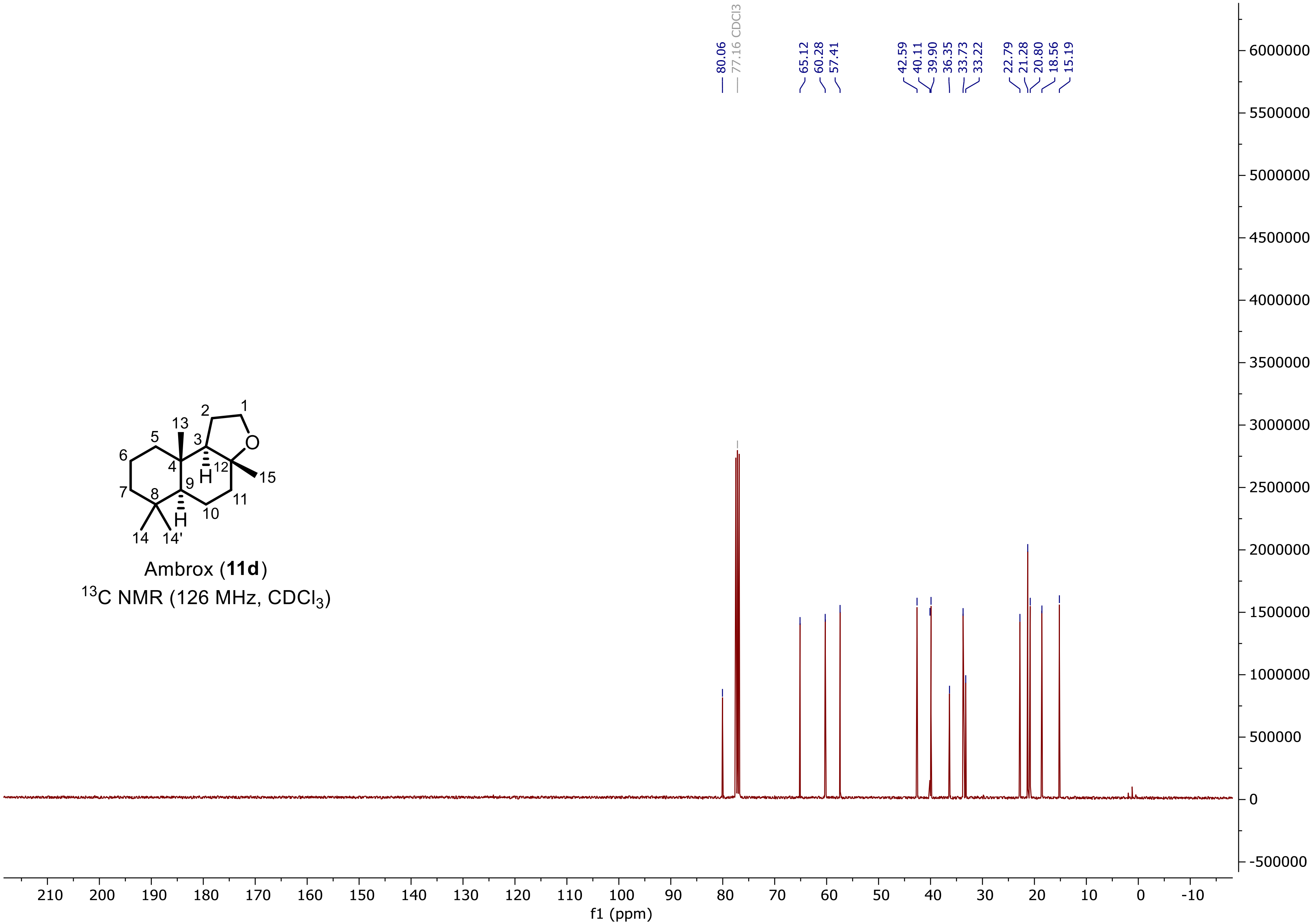

80.06
77.16 CDCl3
65.12
60.28
57.41
42.59
40.11
39.90
36.35
33.73
33.22
22.79
21.28
20.80
18.56
15.19
Ambrox (**11d**)
^{13}C NMR (126 MHz, $CDCl_3$)
f1 (ppm)

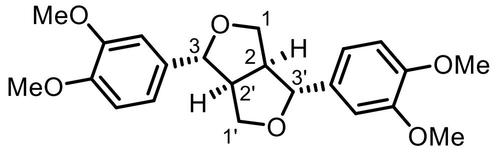

Eudesmin (**11e'**)
^{1}H NMR (500 MHz, $CDCl_3$)

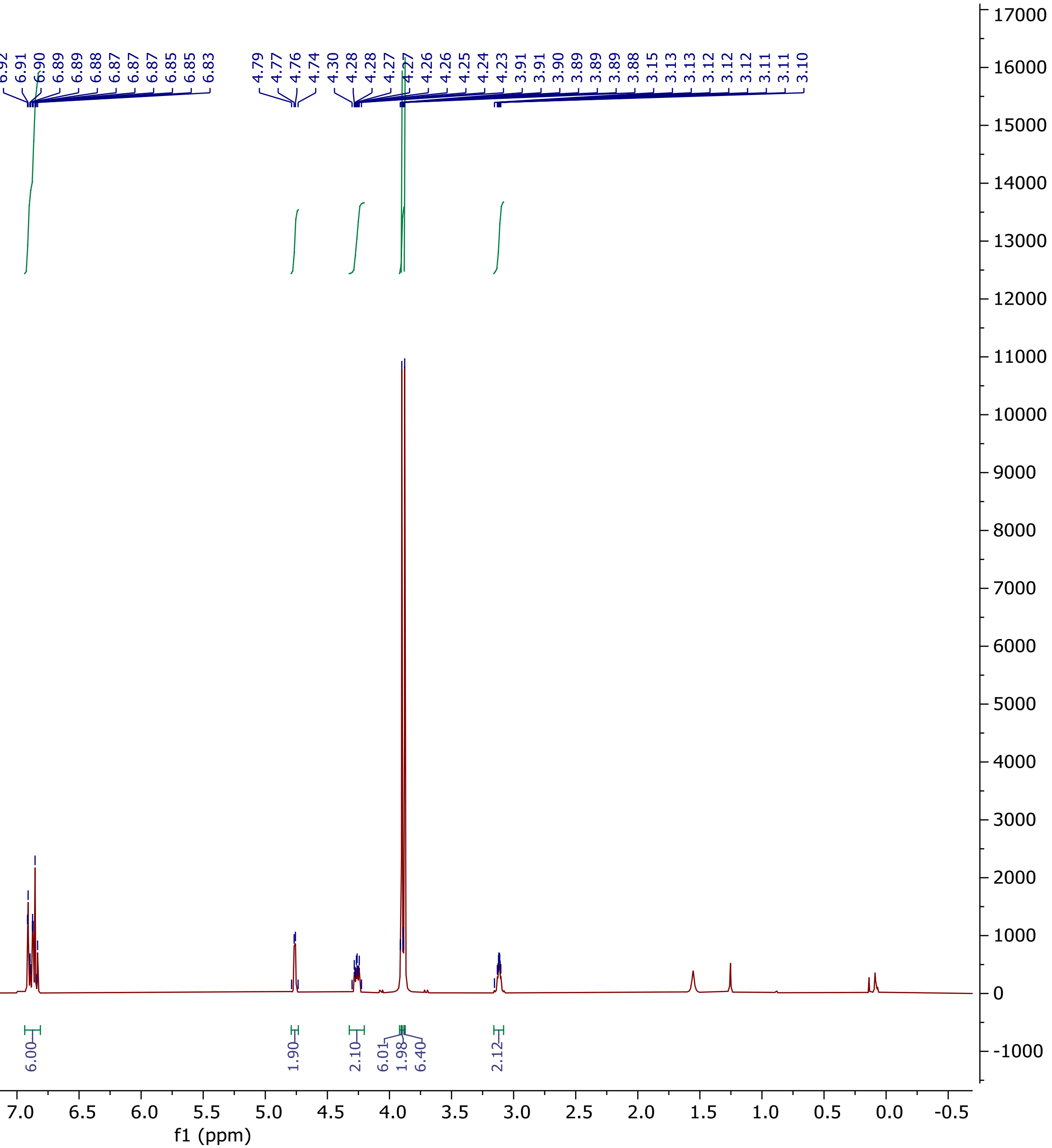

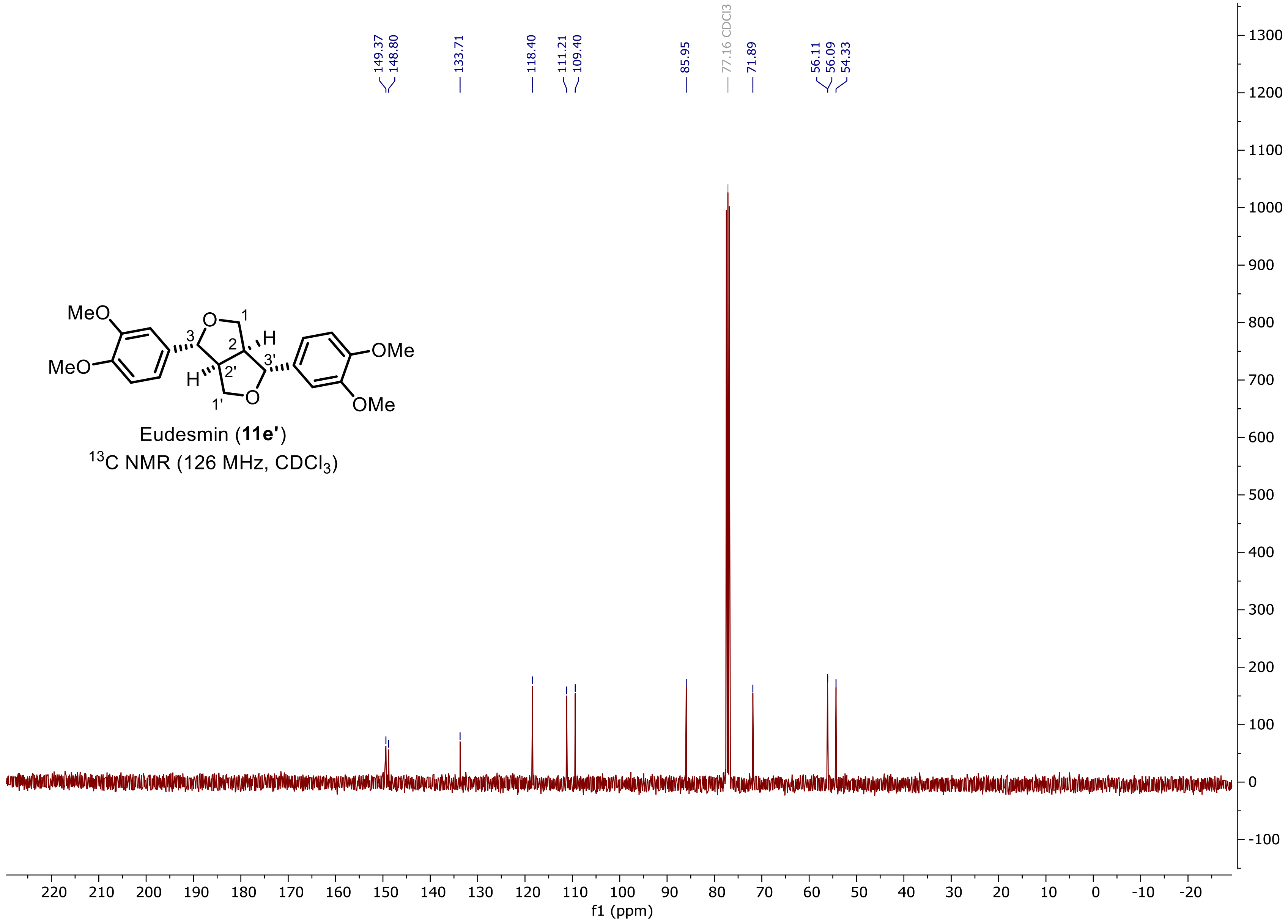

149.37
148.80
133.71
118.40
111.21
109.40
85.95
77.16 CDCl3
71.89
56.11
56.09
54.33
MeO
MeO
OMe
OMe
Eudesmin (**11e'**)
^{13}C NMR (126 MHz, $CDCl_3$)
f1 (ppm)

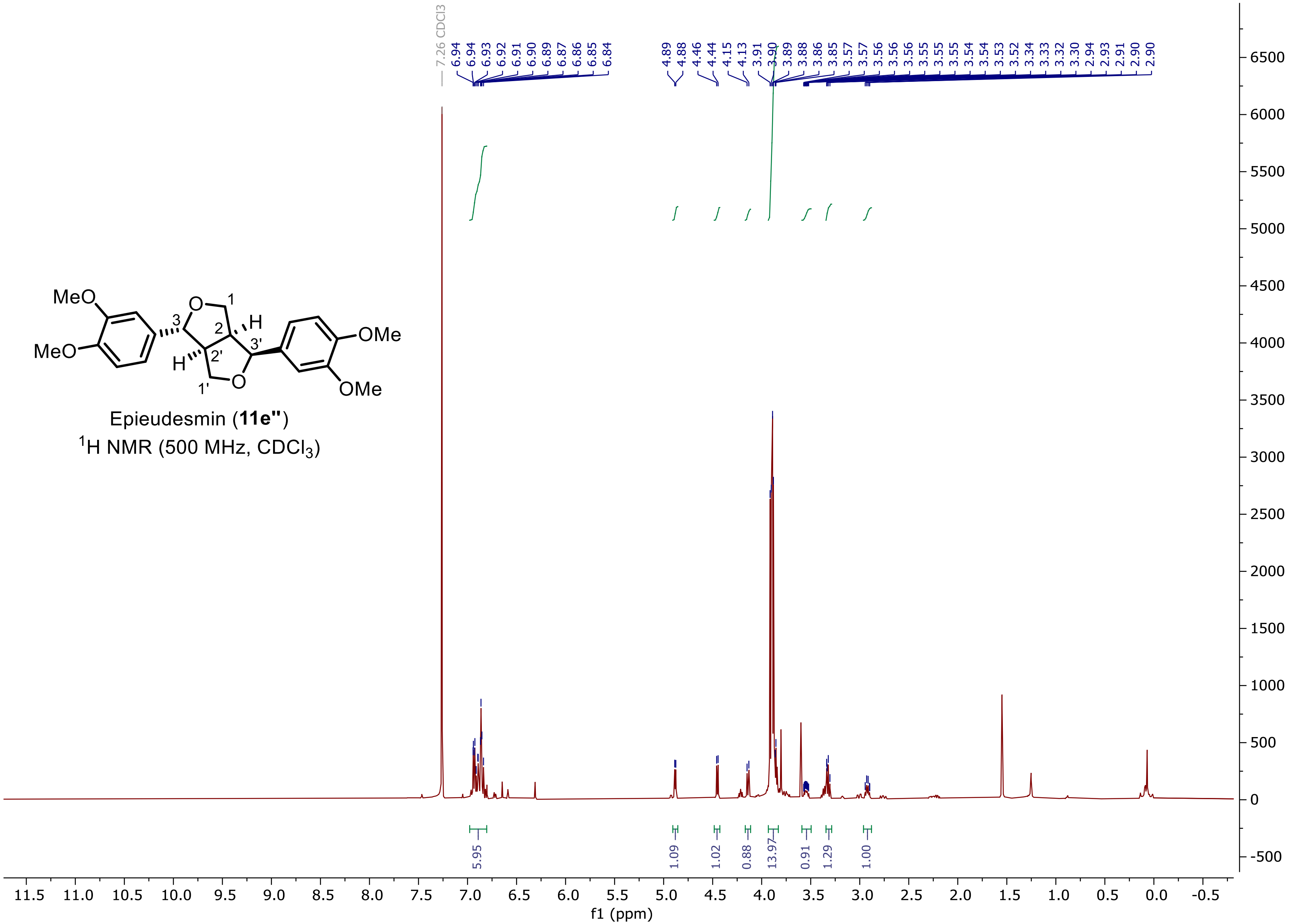

Epieudesmin (**11e''**)
^{1}H NMR (500 MHz, $CDCl_3$)
f1 (ppm)

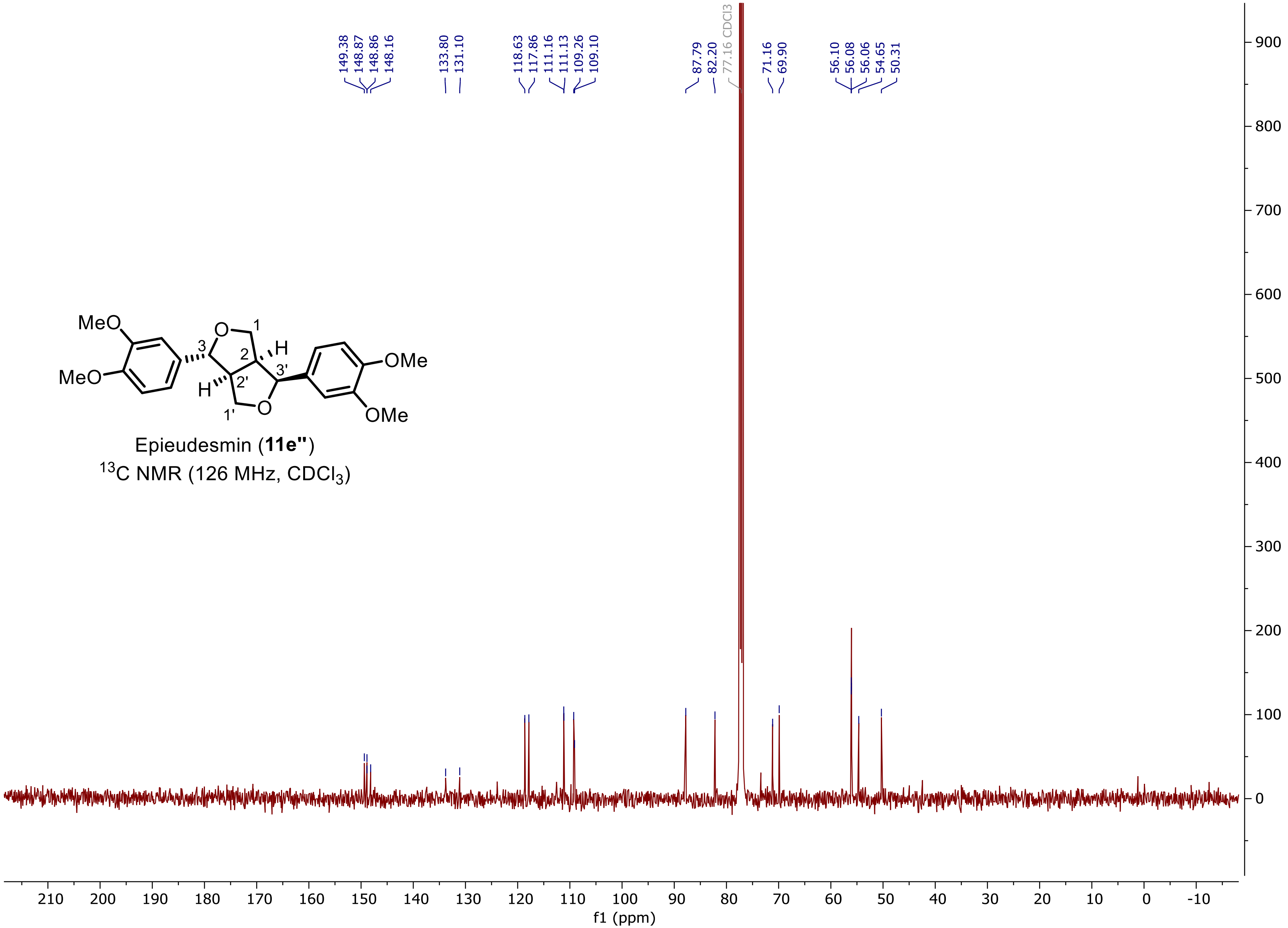
149.38
148.87
148.86
148.16
133.80
131.10
118.63
117.86
111.16
111.13
109.26
109.10
87.79
82.20
77.16 CDCl3
71.16
69.90
56.10
56.08
56.06
54.65
50.31
MeO
MeO
OMe
OMe
Epieudesmin (11e'')
^{13}C NMR (126 MHz, $CDCl_3$)
f1 (ppm)
